# Thermally Distinguishable Polyhedral Shapes in Chemistry: 6- and 7‑Coordination

**DOI:** 10.1021/acsomega.5c05878

**Published:** 2025-09-29

**Authors:** Gabriel H. L. Munguba, Mateus F. da Silva, Frederico T. Silva, Gabriel A. Urquiza-Carvalho, Alfredo M. Simas

**Affiliations:** † Departamento de Química Fundamental, 28116 Universidade Federal de Pernambuco, 50590-470 Recife, Pernambuco, Brazil; § Departamento de Matemática, 28116 Universidade Federal de Pernambuco, 50740-560 Recife, Pernambuco, Brazil

## Abstract

Coordination polyhedra in metal complexes occasionally exhibit marked deviations from ideal geometric shapes, complicating their accurate characterization and symmetry assignment. We introduce a rigorous mathematical approach to establish a complete yet chemically relevant set of thermally distinguishable polyhedral shapes, TDPSs, suitable for describing coordination geometries, specifically focusing on coordination numbers 6 and 7. Anchored in Steinitz’s theorem, we constructed all combinatorially distinct convex polyhedra and then optimized their spatial arrangements using a novel repulsive-type crowding potential, respecting maximum symmetry and minimal repulsion. Owing to thermal smearing, several of these polyhedral forms became experimentally indistinguishable within crystallographic uncertainties. Therefore, we classified these geometries into subsets of thermally interconvertible polyhedra, from which we established a concise set of thermally distinguishable polyhedral shapes. While some of these are already well-established, others represent previously unrecognized shapes in the coordination chemistry literature. Our comprehensive analysis of over 42,000 structures deposited in the Cambridge Structural Database confirmed the not infrequent occurrence of these new shapes. Remarkably, among the five thermally distinguishable shapes identified for hexacoordinated complexes, we identified the digonal anticupola (DAC-6) as a prevalent structure previously overlooked in the coordination chemistry literature. For heptacoordinated complexes, 14 thermally distinguishable polyhedral shapes emerged, including chiral and previously unrecognized shapes that we predominantly identified in lanthanide and alkali-metal complexes. This study thus establishes a robust theoretical foundation, enabling refined structural characterization, more precise symmetry assignments, and facilitating quantitative links to lattice-dynamical and thermal-transport properties (e.g., heat capacity and thermal conductivity) in molecular coordination solids.

## Graphical abstract

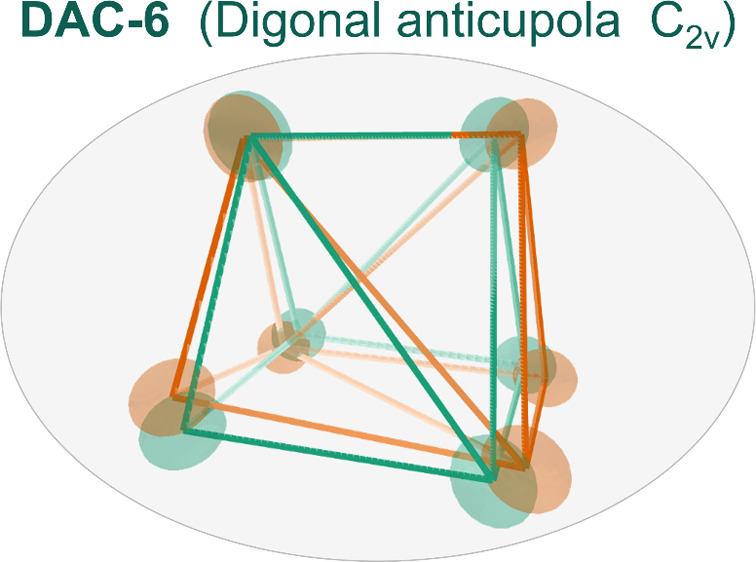

## Introduction

Polyhedral models have long been essential in inorganic chemistry,^1,2^ beginning with Alfred Werner’s octahedral representation of metal complexes over a century ago.^3^ These models help chemists understand and predict the stereochemistry^4–6^ and physicochemical properties of coordination compounds, including metal-centered chirality,^7–9^ structural rearrangements,^10^ and crystal packing.^11,12^ Furthermore, polyhedral shapes serve as templates in computational methods for systematically generating and optimizing various possible stereoisomers.^13–17^

Both theoretical molecular design and experimental data interpretation of metal complexes typically rely on a limited set of reference coordination polyhedra (CPs), primarily regular or semiregular shapes such as Platonic, Archimedean, Catalan solids, prisms, antiprisms, pyramids, bipyramids, and their geometric derivatives.^18–20^ These polyhedral shapes, along with others predicted by Coulomb-based repulsion models,^21–25^ have been used to classify most experimentally observed metal-complex structures, although occasional efforts have also been made to expand this set, exploring less symmetrical polyhedral architectures in the fields of Supramolecular Chemistry^26–30^ and Materials Science.^31,32^

Nevertheless, polyhedra lying outside these boundaries have not been considered in previous studies under the assumption that they might be unstable due to excessive ligand–ligand repulsion,^21,33,34^ or be symmetry-forbidden given the set of atomic orbitals considered,^35,36^ leading them to be prematurely dismissed as unlikely to occur in coordination complexes. Still, in lanthanide complexes, the metal valence orbitals are internal to the ion’s shell structure, resulting in minimal covalent character in coordination bonds and essentially spherical charge densities.^37,38^ Alkali and alkaline earth ions behave similarly.^39^ Truly, when the orbital directionality is weaker than the steric constraints imposed by ligands or the surrounding environment, unconventional coordination geometries can be stabilized.

Minimal-deviation metrics^20,40–45^ are frequently employed to assign an ideal CP to an empirical structure. The most useful approach is the Continuous Shape Measures (CShM) method,^46^ which quantifies the degree of distortion of a given structure relative to a reference polyhedron. CShM calculations can be easily performed by using the SHAPE software, which employs a database of predefined polyhedra to be taken as reference geometries, most belonging to the families of the aforementioned convex solids.

However, CPs in metal complexes do not infrequently exhibit significant deviations from the idealized geometric shapes traditionally regarded as definitive references, blurring their accurate characterization and symmetry assignment and thereby impairing the utility of the resulting structural representations. In such cases, chemists might tend to depict the coordination complex as having a “severely distorted” version of an ideal polyhedron.^47–52^

In this article, we address this pending issue, by means of Graph Theory, as it provides a complete description of all possible combinatorially distinct convex polyhedra in terms of their topologies.

A convex polyhedron is a solid shape with flat faces, straight edges, and vertices, where any line joining two interior points remains inside the shape. Its essential structure can be captured by its wireframe skeleton, which is a 3D graph. A graph, in simple terms, is just a group of points called vertices linked together by lines known as edges. Steinitz’s theorem shows that this 3D polyhedral graph can be drawn on a flat 2D surface without overlaps (in a drawing known as a Schlegel diagram^53^), without loops or parallel edges, and remaining connected even when any two vertices are removed.^54^ Such a graph then exactly represents the polyhedron’s structure. In other words, the flat “shadow” of the polyhedron (Figure 1) contains all the information needed to rebuild its three-dimensional form.

**Figure 1:**
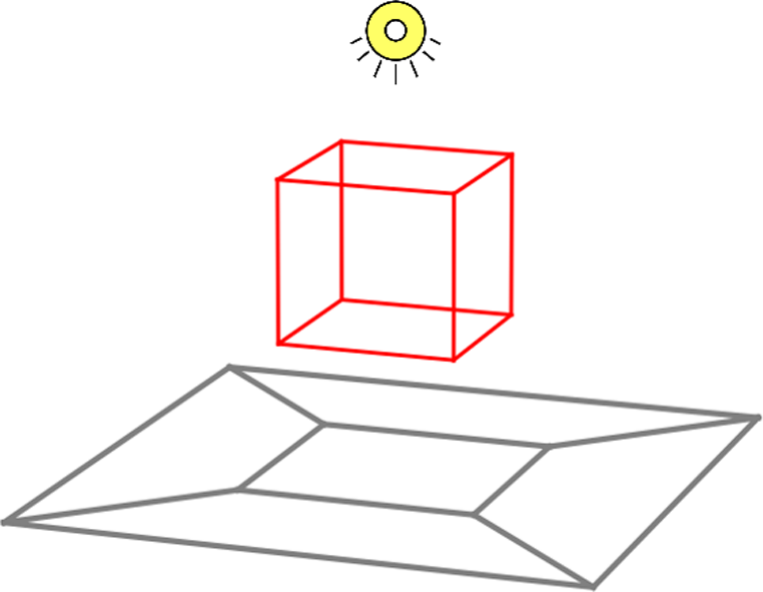
Illustration of the perspective drawing of a 3-connected planar graph *G* as the shadow projection (a Schlegel diagram) of a convex polyhedron *P*, exemplified by a rectangular prism.

Computer algorithms have been used to determine, using graphs, how many distinct convex polyhedra can be formed for a given number of vertices. These studies^55–58^ showed that, for each specific number of vertices, only a finite number of different polyhedra exist. Currently, the exact numbers of these polyhedra are mostly known for up to 18 vertices.^59^ Table 1 provides these numbers^59–64^ for polyhedra with 4 to 12 vertices, which are especially relevant in coordination chemistry.

**Table 1:** Number of All Nonisomorphic Convex Polyhedra by Coordination Number (CN)^a^

CN	n^o^ of convex polyhedra
4	1
5	2
6	7
7	34
8	257
9	2,606
10	32,300
11	440,564
12	6,384,634

a The numbers in this table have been extracted from the web page ref 65.

The values indicate that the number of distinct convex polyhedra increases factorially with the coordination number (CN), ranging from a single polyhedron for tetracoordination—whose geometrical realization of maximum symmetry corresponds to the platonic tetrahedral geometry—up to more than six million topologically nonequivalent distinct polyhedra for CN-12. As CN increases, the number of combinatorially distinct polyhedra grows significantly, and the distinctions among individual polyhedra become progressively less pronounced, reducing the relevance of identifying each polyhedron individually based on its uniqueness.

In this article, we introduce the concept of thermally distinguishable polyhedral shapes to better reflect the physical reality. We group together polyhedra that readily interconvert due to thermal smearing, thereby shifting the focus from individual polyhedra to these clusters. We then represent each cluster by a single characteristic polyhedron. For CN-4 and 5, all possible nonisomorphic polyhedra are already well-established in coordination chemistry: the tetrahedron for CN-4, and the trigonal bipyramid and square pyramid for CN-5. However, for higher coordination numbers, additional polyhedral architectures exist beyond those conventionally recognized. In this initial study, we focus specifically on the CN-6 and CN-7 cases. We systematically constructed all possible 6- and 7-vertex CPs from their corresponding skeleton graphs, grouped them into clusters of thermally distinguishable polyhedral shapes, and identified representative polyhedra for each cluster. By presenting the idealized structures of these representative polyhedra, we provide a more comprehensive set of reference shapes and structural templates. Our findings enable a more accurate characterization of metal complex geometries and facilitate improved structural assembly for molecular modeling.

## Methodology

### On the Choice of the Polyhedral Representations

In the study of CPs, it is useful to emphasize their intrinsic connectivity and combinatorial arrangement rather than the precise spatial configuration or metric details of their constituent elements. This abstraction allows us to classify them based on combinatorial properties and symmetry, facilitating easier comparison and theoretical analysis, especially in contexts where the exact dimensions are less critical than the overall arrangement and topology. Thus, we capture the combinatorial type of a convex polyhedron by its 1–skeleton graph *G* = (*V,E*), where *V* is the set of vertices and *E* is the set of edges connecting them. By Steinitz’s theorem, a finite graph arises as the edge-vertex graph of some convex polyhedron exactly when it is planar and remains connected after removal of any two vertices (i.e., it is 3-connected).^66,67^ We say two convex polyhedra *P* and *Q* in $R^{3}$ are combinatorially equivalent precisely when their 1-skeleton graphs are isomorphic.

We define shape in this work as the entire class of convex polyhedra sharing the same 1-skeleton graph *G*, regardless of their specific edge lengths or dihedral angles.

We constructed a 3D representation of a convex polyhedron by first embedding its graph on a plane and then “lifting” this planar drawing into three dimensions. In this process, we began with Tutte’s barycentric method,^68^ in which the edges of the graph act as constraints that balance the positions of neighboring vertices. One face is selected as the fixed outer boundary, drawn as a convex polygon with predetermined Cartesian coordinates, while all other vertices are positioned within this frame such that each interior vertex is placed at the weighted average, or barycenter, of its neighbors. This equilibrium is achieved by solving a system of linear equations, which can also be interpreted as minimizing an energy function analogous to that of a spring system.

Once the graph is embedded in the plane, we employed the Maxwell-Cremona correspondence,^69,70^ which uses dual graphs to assign heights, ensuring faces remain planar when lifted, providing a systematic way to lift the framework into three-dimensional space. A specific face—preferably a triangle—is chosen to lie in the xy plane, while the remaining faces are raised in the positive z-coordinate. The heights assigned to the vertices are derived through a construction that involves the dual graph, where each face of the original graph corresponds to a vertex and adjacent faces (sharing an edge) are connected. By calculating contributions from these dual relationships, we assigned a vertical displacement to every vertex, yielding new coordinates (*x*,*y*,height) and thus formed a convex polyhedron.

Moreover, every graph possesses an automorphism group, ***Aut*** (*G*), which consists of all permutations of its vertices that preserve the connectivity of the graph, thereby capturing its inherent symmetries. According to Mani’s theorem,^71^ for polyhedral graphs, these abstract symmetries correspond to actual geometric symmetries (e.g., rotations, reflections) in at least one 3D realization. In effect, there exists at least one convex polyhedron whose Euclidean symmetries reflect every combinatorial symmetry of its underlying graph.

Despite Steinitz’s theorem associating a polyhedral realization for a 3-connected planar graph, infinitely many convex representations satisfying the graph topology are possible.^18^ For example, let us consider the Johnson solids, i.e., convex polyhedra whose faces are regular polygons.^72^ They are built so all their edges measure the same length.^1,23,24^ This representation seems appropriate for metallic clusters or supramolecular assemblies.^73^ A spherical representation, more suitable for metal complexes, in which the distances from each vertex to the center of mass of the polyhedron are identical, can also be constructed for this same set of shapes, as will be comprehensively demonstrated in the present article. These two representations do not strictly overlap. Indeed, the enveloping shell of an equal-edge-length polyhedron might be an ellipsoid rather than a perfect sphere.

Numerical algorithms^74–78^ often aim for a canonical form^66^—a standard representation in which every edge is tangent to a fixed sphere and the centroid of the contact points is the origin. This canonical form is unique up to Möbius transformations of the sphere and ensures that all abstract symmetries are visibly realized as geometric isometries.

We employed the iterative procedure proposed by Hart^78^ to refine the polyhedral structure into a symmetry-optimized canonical form, taking the representation calculated using Maxwell-Cremona correspondence as input. In this approach, each edge’s proximity to the origin is evaluated, and its endpoints are adjusted perpendicularly to balance distances across the structure. Vertices are globally recentered, and corrections are applied to preserve face planarity, ensuring no vertex strays too far from the ideal plane of its face. Through repeated iterations, the polyhedron converges to a unique, symmetric configuration in which all combinatorial symmetries of the graph become geometrically visible as Euclidean isometries.

Albeit a canonical form of *P* with edge-tangency to $S^{2}$ might be particularly of interest for displaying its maximum symmetry, for metal complexes, the best representation is not edge-circumscribed, but vertex-inscribed, with the vertex-to-center distances being approximately equal.^21–23,79,80^

Inscribability, nevertheless, does not guarantee a unique representation for *P* satisfying both the maximum symmetry and origin-centered centroid constraints. Hence, an additional condition is required to ensure that the algorithm outputs good representations of each CP suitable for a real coordination sphere situation. If the ligand–ligand interactions are treated as mainly electrostatic and both attractive forces and geometrical restrictions on the ligand are neglected, the spatial arrangement of the vertices can be optimized via a repulsive potential of the form +1/*r*.^80,81^ This function must be applied to disperse the charge-like points over a spherical shell under three strict conditions: the points must lie on the spherical surface, the polyhedron’s topology must remain intact, and every automorphism of its polyhedral graph must appear in the optimized form as a spatial symmetry operation. We call the resulting configuration MSMR—maximum symmetry and minimum repulsion.

A schematic of the main tasks performed by our algorithm to calculate good MSMR representations of CPs is shown in Figure 2.

**Figure 2:**
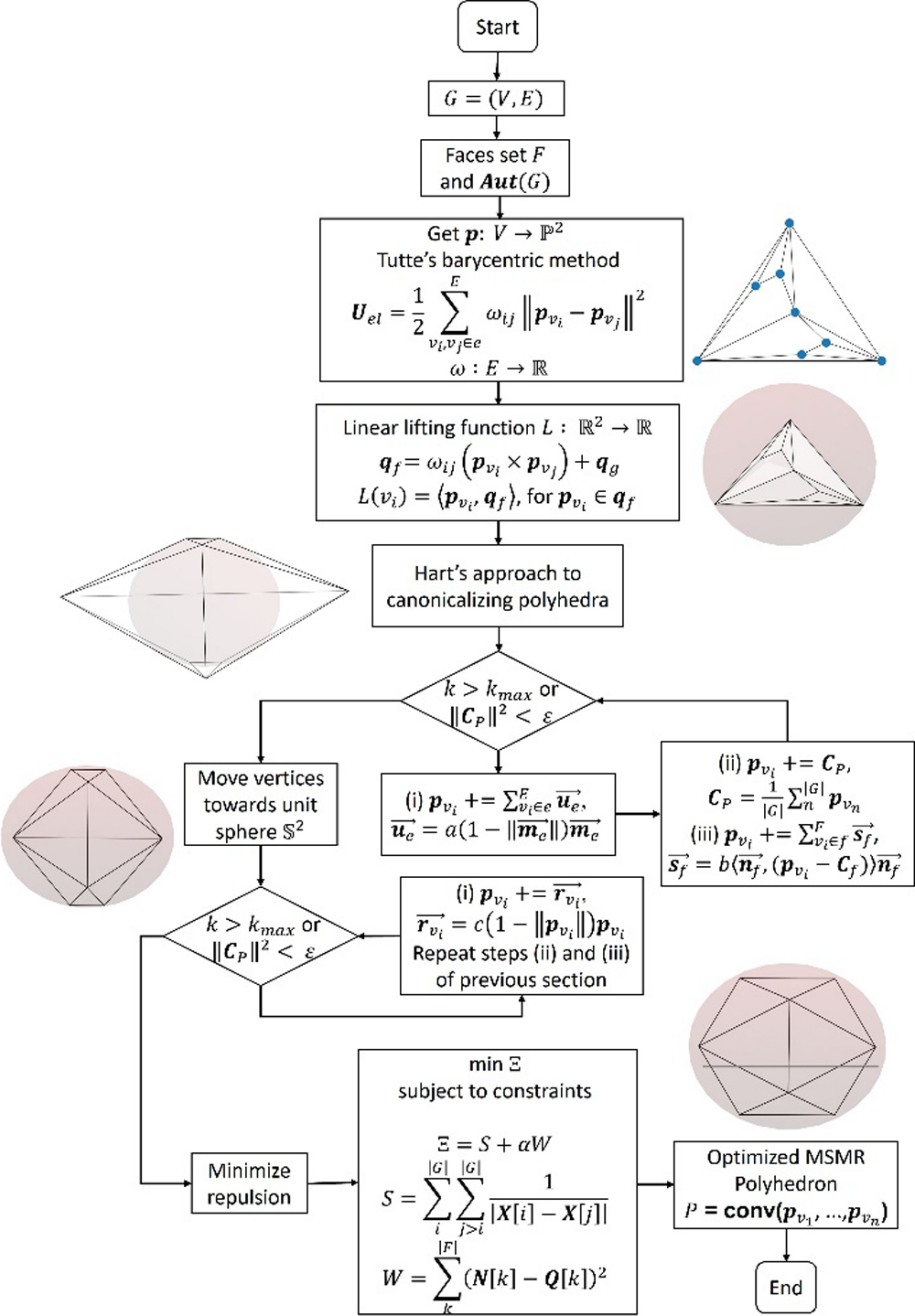
Flowchart outlining the hierarchy and interconnections of subtasks involved in generating optimal 3D drawings of maximum symmetry and minimum repulsion (MSMR) coordination polyhedra from their skeleton-graphs.

### Objective Function

In this work, we chose an adapted version of our Crowding function Ξ, first introduced in our Complex Build algorithm,^13^ as objective-function to spread the vertices along $S^{2}$ and minimize the repulsion.

Originally, this function Ξ is defined as the sum of a steric congestion term *S* of form +1/*r* plus a Hooke-like coordination warp *W* penalty term of type (*x* – *x*_0_)^2^.

Ξ=S+αW 1

$$S=\sum_{n}^{\left|A\right|}\sum_{m>n}^{\left|A\right|}\frac{1}{\left|X[n]-X[m]\right|}$$ 1a

$$W=\sum_{n}^{\left|\Pi \right|}(X[n]-Z[n]{)}^{2}$$ 1b

where in *S* the summations run over all atoms of the coordination complex, while in *W* the summation runs over the coordinating atoms only, and α is a positive scalar parameter. The coordination warp term ensures the position of the *n*th-tooth **X**[*n*] in the pre-optimized stereoisomer structure to be close to the position vector of the n^th^ vertex of an ideal polyhedral shape matrix **Z** given as reference geometry, scaled to the coordination bond reference length.

We adapted the Crowding function, henceforth called Potential, to obtain optimal MSMR representations of all nonisomorphic CPs studied in this work. Here, the penalty term was modified so that the summation goes over the normal vectors of the faces. The summations in the steric congestion term run over all vertex pairs of *P*. Here, we assume that all vertices represent equal charged points. With these modifications, the two terms *S* and *W* become

$$S=\sum_{i}^{\left|G\right|}\sum_{j>i}^{\left|G\right|}\frac{1}{\left|X[i]-X[j]\right|}$$ 2

$$W=\sum_{f}^{\left|F\right|}(N[f]-Q[f]{)}^{2}$$ 3

where **X** is the position vector matrix of the vertices of *P*, **N** is the normal vector matrix of the polyhedron being optimized in the current *k*th iteration, and **Q** is the analogous matrix calculated in the previous (*k* – 1)­th step, given as a reference structure for the algorithm. This penalty term allows small modifications on the vertices’ positions to minimize the overall repulsion while also trying to preserve the topology of *P*, guiding the algorithm along the high-dimensional potential surface. During the geometry optimization, the value of the scalar parameter α can vary within the interval (0, 100].

As minimization techniques, we employed the Sequential Least Square Programming algorithm^82^ (SLSQP) coupled with the Basin-hopping approach to induce global minima^83^ search to the minimizer. Symmetry relations between the edges of *P*, as indicated by ***Aut*** (*G*), inscribability, planarity of faces, and centrality at the origin, are imposed as constraints to the algorithm in the last step.

This minimization procedure, applied to polyhedral shapes that admit an inscribed representation^84,85^ may output good, optimized forms of CPs with all vertices strongly touching the sphere. Noninscribable polyhedra^86,87^ may have optimized representations with their vertices as close as possible to the unit sphere. For CN-6 and CN-7, however, all combinatorially distinct polyhedra are inscribable.

### Modeling Atomic Displacements

Along with optimizing the spatial arrangement of charged points representing ligands around a metal center through minimization of the Coulomb-like potential, we also account for intrinsic atomic and molecular motions that contribute to uncertainties in atomic positions. In fact, atoms within a molecule are inherently dynamic, even in crystalline systems. Their displacements can alter the local geometry^88^ and impact the interpretation of CPs, depending on their magnitudes. Therefore, they must be considered when constructing sets of reference geometrical shapes.

In this work, we considered the types of atomic motions that can occur in a crystal. In crystallography, temperature-dependent vibrations and types of disorders (static and dynamic) may occur, causing displacements in the atomic positions.^89^ These effects yield diffuse scattering and weakening or alteration of the Bragg intensities.^90^ The resulting variations in the atomic positions can be quantitatively modeled by the atomic displacement parameters (ADPs), which estimate the mean square displacement of atoms around their average positions within a crystal lattice. These displacements are typically anisotropic in crystalline systems, meaning that they vary in magnitude in different spatial directions. Indeed, ADPs not only provide insights into the dynamics of atomic and molecular motions^91^ but also serve for interpreting crystal structures, including spectroscopic, optical,^92^ and thermodynamic properties.^89^

During structure refinement, combinations of two crystallographic axes lead to the construction of a second-order tensor **U**, a symmetric 3 × 3 matrix with *U*_*ij*_ ≡ *U*_*ji*_, which represents the probability distribution of the electron density location when thermal vibrations and disorder contributions are both considered.^93^

$$U=\left(\begin{array}{ccc} U_{11} & U_{12} & U_{13}\\ U_{21} & U_{22} & U_{23}\\ U_{31} & U_{32} & U_{33} \end{array}\right)$$ 4

Let $X=\left[\begin{array}{c} x\\ y\\ z \end{array}\right]$ be the position vector of an atom and **U**_**cart**_ the anisotropic displacement tensor, both on a Cartesian basis. The equation **X** ^T^ **U**_**cart**_^**–1**^ **X** = *c* ^2^ describes a quadric surface, commonly called a thermal ellipsoid, with constant probability density.^94^ The percentage of probability density this surface covers depends on the value ascribed for *c*. The probability distribution of this variable, *P*(*c*), has a Gaussian form and is proportional to the dimensionality *n* of the quadric (here, 3):

$$P(c)=\frac{2^{1-n/2}c^{n-1}}{\Gamma \left(\frac{n}{2}\right)}\exp\left(-\frac{1}{2}c^{2}\right)$$ 5

where Γ­(*x*) is the Gamma function. The most frequent value for *c* is 1.5382, at which the ellipsoid encompasses 50% of the probability density.^95–97^

The lengths of the semiaxes of the thermal ellipsoid are directly proportional to the square root of the eigenvalues {λ_*i*_} of **U**_**cart**_. If the differences between the magnitudes of anisotropic displacements in each direction are considered to be nonsignificant, the parameters can be approximated to a single quantity that describes the displacement in an isotropic situation.^96,98,99^ The isotropic displacement parameter, **U**_**iso**_ (Å^2^), is calculated from the trace of the **U**_**cart**_ matrix as

$$U_{\mathrm{iso}}=\frac{1}{3}\mathrm{trace}(U_{\mathrm{cart}})$$ 6

In this work, besides computing MSMR representations of all 6- and 7-vertices combinatorially distinct CPs, we aimed to determine the extent to which these polyhedra remain geometrically distinguishable when considering smearing effects in crystals, i.e., phenomena that perturb the atomic positions in a crystal lattice. That is, given a reference value for the spatial displacement amplitude representative of atomic motion in the solid state, we investigated whether some of these geometries could be considered thermally indistinguishable, in the sense that they can be easily transformed into one another with small modifications on their vertices, as illustrated in Figure 3.

**Figure 3:**
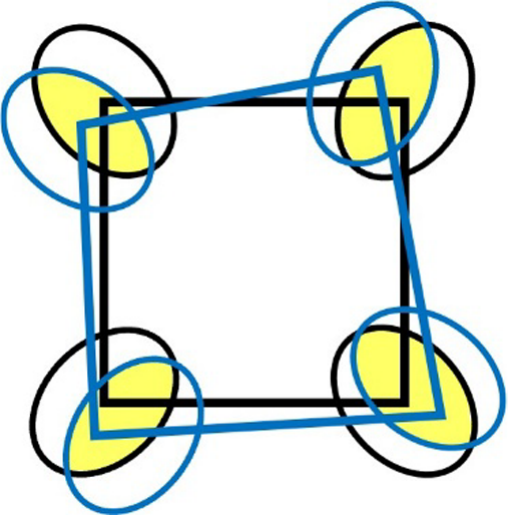
Planar projection of a representative pair of equivalent faces (outlined in black and blue) from two coordination polyhedra that have been superimposed by using an RMSD algorithm. Despite the slight displacement, all four pairs of equivalent thermal ellipses not only overlap but also have their centers positioned inside the area of the corresponding ellipse from the other polyhedron. This indicates that one can be transformed into the other through thermal smearing, confirming their thermal equivalence. Yellow highlights mark the overlapping regions for clarity. We will show later in the article that we will choose the more symmetrical one (the black) to represent the set.

To accomplish this, we selected a set of over 42,000 high-quality crystallographic structures of 6- and 7-coordinate complexes from the Cambridge Structural Database (CSD),^100^ considering all metallic elements of the Periodic Table. Only metal complex structures, either neutral or ionic, with an *R*-factor ≤ 5.0, no structural disorder in the coordinating atoms, and no overlap of distinct stereoisomers resulting from nonresolvable structures were included.

We extracted **U**_iso_ values for all crystallographically independent coordinating atoms from the pre-existing CIF files selected from the CSD. The associated radii of their quadric surfaces (a sphere) were calculated by taking the square root of **U**_iso_. Each value was then scaled to the unit sphere in proportion to the coordination bond lengths specified in the crystallographic file, obtaining a dimensionless variable r. Following this protocol, over 100,000 displacement radius data were computed and stored. The isotropic displacement values were calculated considering a 50% coverage of the probability density.

We performed statistical analyses on these data to estimate a reference value for the maximum displacement of the vertices of an inscribed polyhedron under isotropic motion. For each pair of polyhedral forms, *P*_1_ and *P*_2_, we assessed the minimum root-mean-square deviation (RMSD) between the shapes using Marques et al. algorithm.^101^

$$d^{2}=\sum_{i=1}^{N}(x_{1}^{i}-x_{2}^{i}{)}^{2}+(y_{1}^{i}-y_{2}^{i}{)}^{2}+(z_{1}^{i}-z_{2}^{i}{)}^{2}$$ 7

In this approach, during the alignment of the polyhedra, their position vector matrices are reordered according to the spatial proximity of their vertices. Once the best superpositions were found, we computed the separation distances for each vertex pair {***v***_1_^*i*^, ***v***_2_^*i*^}, where ***v***_1_^*i*^ is the *i*th vertex of *P*_1_ and ***v***_2_^*i*^ the *i*th vertex of *P*_2_, and the closest to ***v***_1_^*i*^. Two polyhedral shapes are thermally indistinguishable if each vertex of one polyhedron can shift its position to the nearest vertex of the other geometry without exceeding the limit value for vertex motion, calculated from ADPs.

## Results and Discussion

As indicated in Table 1, for CN-6 and 7, there are, respectively, 7 and 34 nonisomorphic polyhedral graphs.

Let us first consider the case of hexacoordination. Out of the seven graphs, one corresponds to a chiral tetragonal antiwedge (TAW-6) geometry of the C_2_ maximum symmetry point group. Thus, there are two possible representations in $R^{3}$ for this polyhedron that is nonsuperimposable through isometries of the first kind (proper rotations). For the 34 polyhedra of the heptacoordination case, 11 are chiral, out of which 4 have a maximum C_2_ symmetry, and 7 are completely asymmetric, of the C_1_ point group symmetry.

In this work, we considered all chiral 6- and 7-coordinate polyhedra in both enantiomorphic configurations. They are distinguished by the prefixes Δ and Λ, which denote right-handed and left-handed twists, respectively, extending the convention commonly used for octahedral geometries in coordination chemistry.

In Table 2, we indicate for each CP calculated by our algorithm its maximum symmetry point group (PG), its corresponding number of symmetry elements (*h*), its number of edges (E) and faces (F), the number of n-gonal faces with 3 ≤ *n* ≤ 7, its calculated minimum potential value Ξ, and the average vertex-pair distance η in the final MSMR optimized form together with its standard deviation κ. The CPs are labeled by an index number and, in some cases, by a name abbreviation.

**Table 2:**
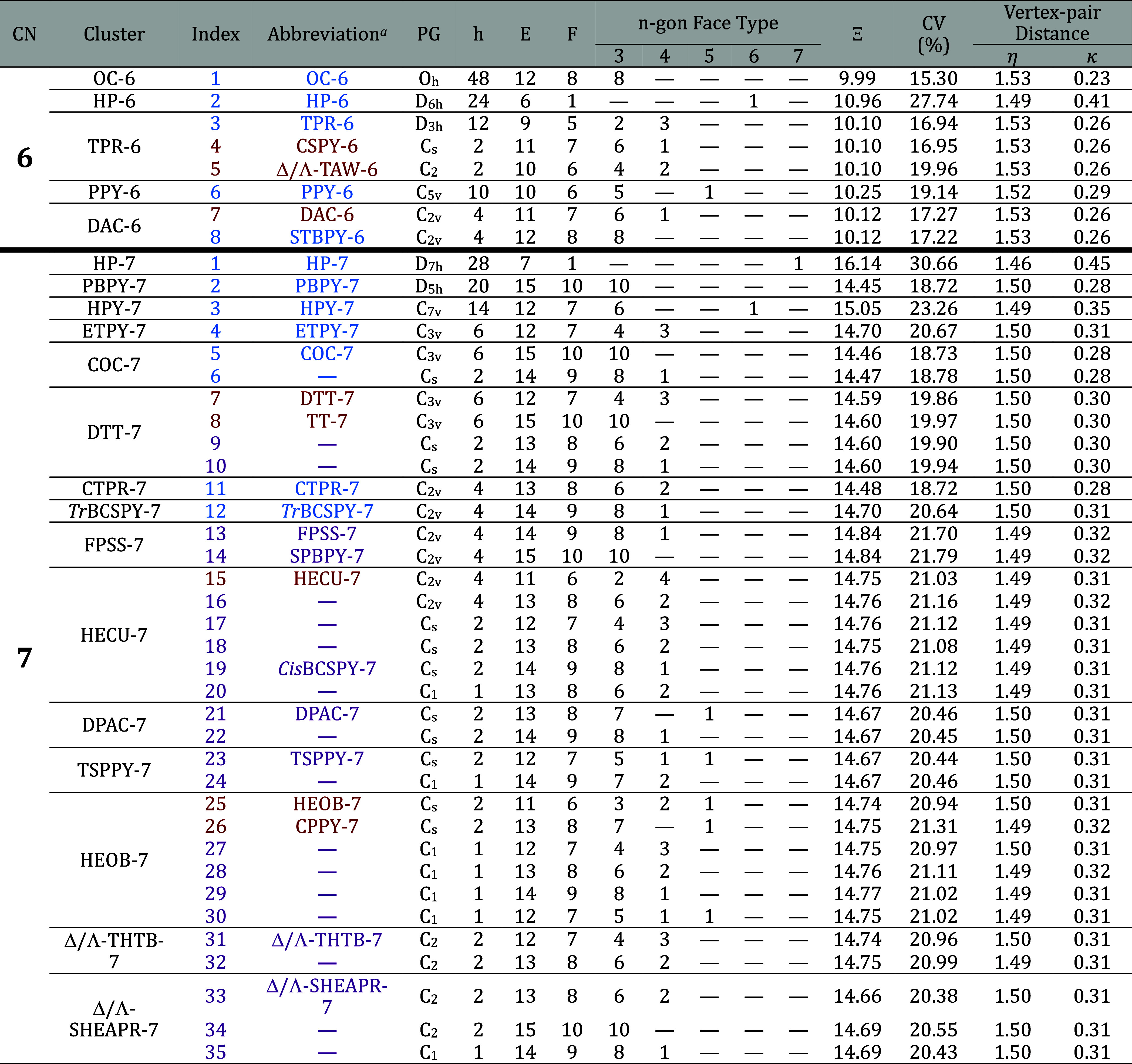
Description of All Combinatorially Distinct 6- and 7-Vertex Shapes Based on Their Features: Coordination Number (CN); Thermally Distinguishable Cluster Symbol; Index Number; Abbreviation; Point Group (PG); *h*, The Number of Symmetry Elements of the Polyhedron’s Point Group, as Defined in Group Theory; Number of Edges E; Number of Faces F; n-Gon Face Types: Triangular (3), Quadrilateral (4), Pentagonal (5), Hexagonal (6) and Heptagonal (7); Final Crowding Potential Value **Ξ** of Their Maximum Symmetry and Minimum Repulsion Representations, Inscribed in a Unity Radius Sphere (arbitrary Unit); the Coefficient of Variation CV(%), defined as the ratio of the standard deviation to the average; and the Average Vertex-pair Distance η, and its Standard Deviation κ, Both on the Same Arbitrary Unit^b^

a OC-6: octahedron, TPR-6: trigonal prism, PPY-6: pentagonal pyramid, STBPY-6: skew trapezoidal bipyramid (or bicapped tetrahedron), DAC-6: digonal anticupola, CSPY-6: capped square pyramid, Δ/Λ-TAW-6: tetragonal antiwedge, PBPY-7: pentagonal bipyramid, CTPR-7: capped trigonal prism, ETPY-7: elongated trigonal pyramid, HPY-7: hexagonal pyramid, TrBCSPY: *trans*-bicapped square pyramid, COC-7: capped octahedron, HECU-7: hemicube, *Cis*BCSPY: *cis*-bicapped square pyramid, HEOB-7: hemiobelisk, CPPY-7: capped pentagonal pyramid, DPAC-7: digonal pseudoanticupola, TSPPY-7: tetragon-substituted pentagonal pyramid, FPSS-7: five-pointed scallop shell, SPBPY-7: skew pentagonal bipyramid, DTT-7: diminished trigonal trapezohedron, TT-7: tricapped tetrahedron, Δ/Λ-THTB-7: tetragonal helicoid with tetragonal base, Δ/Λ-SHEAPR-7: square hemiantiprism.

b Chemically established shape abbreviations are depicted in blue, while those in deep red-orange correspond to polyhedra recognized in other fields. In contrast, the dark magenta shapes denote shapes that are unique to our study; for some, we propose fresh symbolic abbreviations.

For polyhedral shapes such as the octahedron (OC-6), the trigonal prism (TPR-6), the capped octahedron (COC-7), the pentagonal bipyramid (PBPY-7), and the capped trigonal prism (CTPR-7), our model found the same minimum potential values reported in the literature.^21,102,103^ For other geometries, such as the tetragonal antiwedge (TAW-6), the digonal anticupola (DAC-6), the elongated trigonal pyramid (ETPY-7), and a 7-vertex geometry, belonging to a “tetragonal base–trigonal base” ^18,104^ family of polyhedral structures, our optimized MSMR representations present lower potential values than those calculated in previous works using similar functions.^21^ Unlike King^21,34^ who minimized the force-weighted criterion ∑1/*r_ij_* ^2^, we minimized the inverse-distance (Coulomb) potential ∑1/*r_ij_* because it has a clear physical interpretation as an energy and weights all pair separations rather than disproportionately emphasizing the nearest contacts, making it well suited to generating symmetry-maximizing reference geometries with a transparent physical analogy.

Information on the minimum of repulsion achieved for the remaining shapes was not found in the literature, likely because these were so far unknown in the chemistry literature for coordination compounds.

### Atomic Displacements and Thermal Smearing Clusters

As previously mentioned, the challenge in studying all combinatorially distinct convex polyhedra in $R^{3}$ for a given coordination number is 2-fold: (1) as the number of vertices increases, the number of conceivable nonisomorphic polyhedral architectures grows factorially, and (2) these geometries tend to be of low symmetry; most being chiral, belonging to the C_1_ point group. For illustration, in CN-12, there are over 6 million distinct polyhedral graphs (Table 1). However, more than 99% of these skeletons correspond to chiral polyhedra.^61,105^ Consequently, constructing the MSMR representations of all these polyhedral shapes would yield about 12 million geometric configurations on a spherical shell, all nonequivalent by proper rotations. Indeed, implementing all of these representations, for instance, as reference shapes for algorithms to assign the CPs of metal complexes would be futile.

In our work, midsphere representations of each CP are calculated first, prior to inscribing them onto the unit sphere. Following this protocol, we noticed that, although the canonical representation of each polyhedron exhibits significant structural differences (Figure 4), some of their MSMR representations demonstrate a great level of geometric similarity concerning the relative displacement of their vertices.

**Figure 4:**
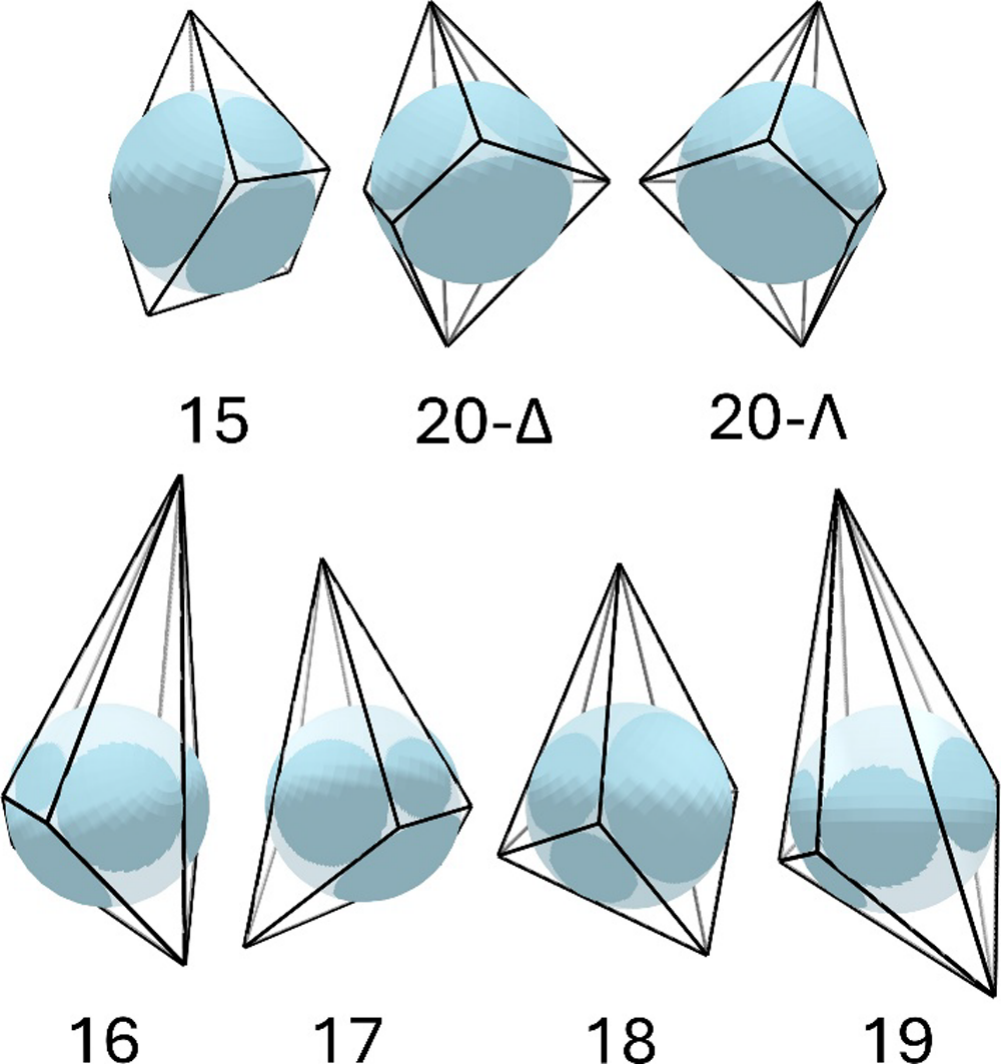
Edge-tangent midsphere representations of seven 7-vertex polyhedra. The spherical caps are shaded darker to distinguish them from the portions of the sphere that lie inside the polyhedron.

This is verified when the geometries are aligned via RMSD minimization, as shown in Figure 5. As observed, although the 7-vertex polyhedra shown as illustrations have distinct topologies, their alignments are nearly perfect. This suggests that their minimum repulsions on the high-dimensional potential surface position them in a close alignment. Because of this, later in this article, we will show that they effectively become indistinguishable from one another due to thermal smearing.

**Figure 5:**
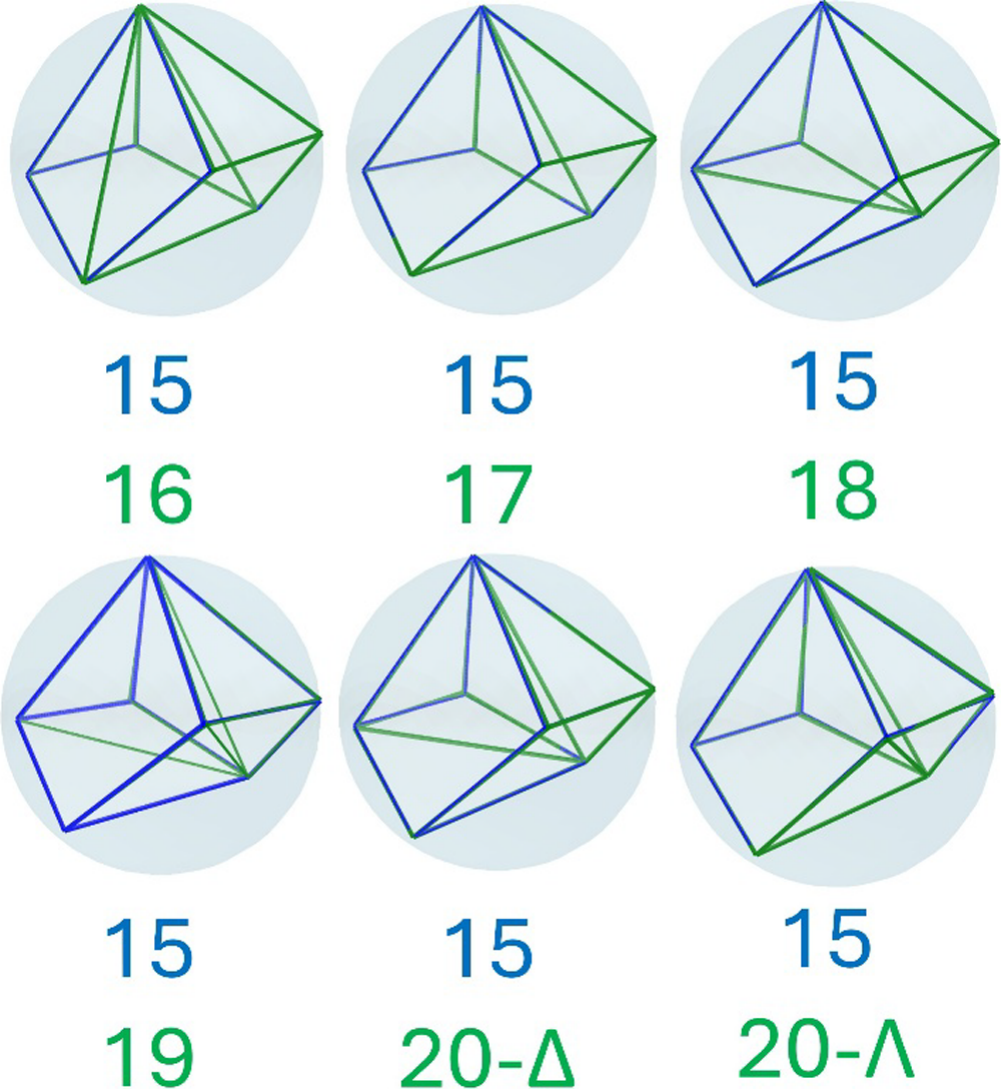
RMSD alignment, in pairs (blue and green) of the inscribed MSMR (maximum symmetry minimum repulsion), forms a few pairs of four 7-vertex polyhedra, highlighting their geometric similarities.

If these shapes are sufficiently similar in terms of geometric parameters, one might select a single polyhedral form, such as the one of index 15, as representative of this subset of polyhedra that share great geometrical similarities.

Let us now proceed to examine to what extent such an approximation would still be valid. In minimum-deviation metrics, guidelines are provided to evaluate the validity of representing a distorted polyhedron found on an empirical structure by a reference geometry of maximum symmetry. For example, Alvarez points out that, for the CShM metric distortion, values greater than 3 of its units from a reference polyhedron indicate that the ideal shape characterizes the metal complex structure quite rudimentarily.^1^ However, this threshold value is based on their computational experiments and does not necessarily include rigorous mathematical or chemical criteria at its core.

In this work, we aim to define a cutoff parameter with physicochemical significance, based on experimental ADP data from crystallography. This parameter provides a reference value that represents the magnitude of atomic displacement in the solid state. When the MSMR geometries of an [ML_n_] system, calculated using a 1/r potential, exceed this threshold, they can be considered distinguishable in terms of thermal smearing. This includes mainly harmonic thermal vibrations, zero-point vibrational motion, unresolved static and dynamic disorder, and the anisotropic nature of atomic displacements.

Conversely, when the displacements applied to the polyhedral vertices to simulate atomic crystallographic motion remain below this reference value, the geometric shapes can be regarded as “thermally equivalent.” In this case, thermal smearing allows these MSMR geometric shapes to transition between one another. To achieve this, we approximate the anisotropic motions typical of crystalline systems to isotropic ones using mean-square displacement amplitude values averaged over all directions (**U**_**iso**_).

We split our ADP data into two sets, one for each coordination number. Given the predominance of 6-coordinate complexes in crystallographic databases, the first set contains a significantly larger amount of collected data (over 94,000 inputs). In Figure 6, histograms of the displacement dimensionless radii values, r, that had been adjusted to a unit coordination bond length are plotted for each CN.

**Figure 6:**
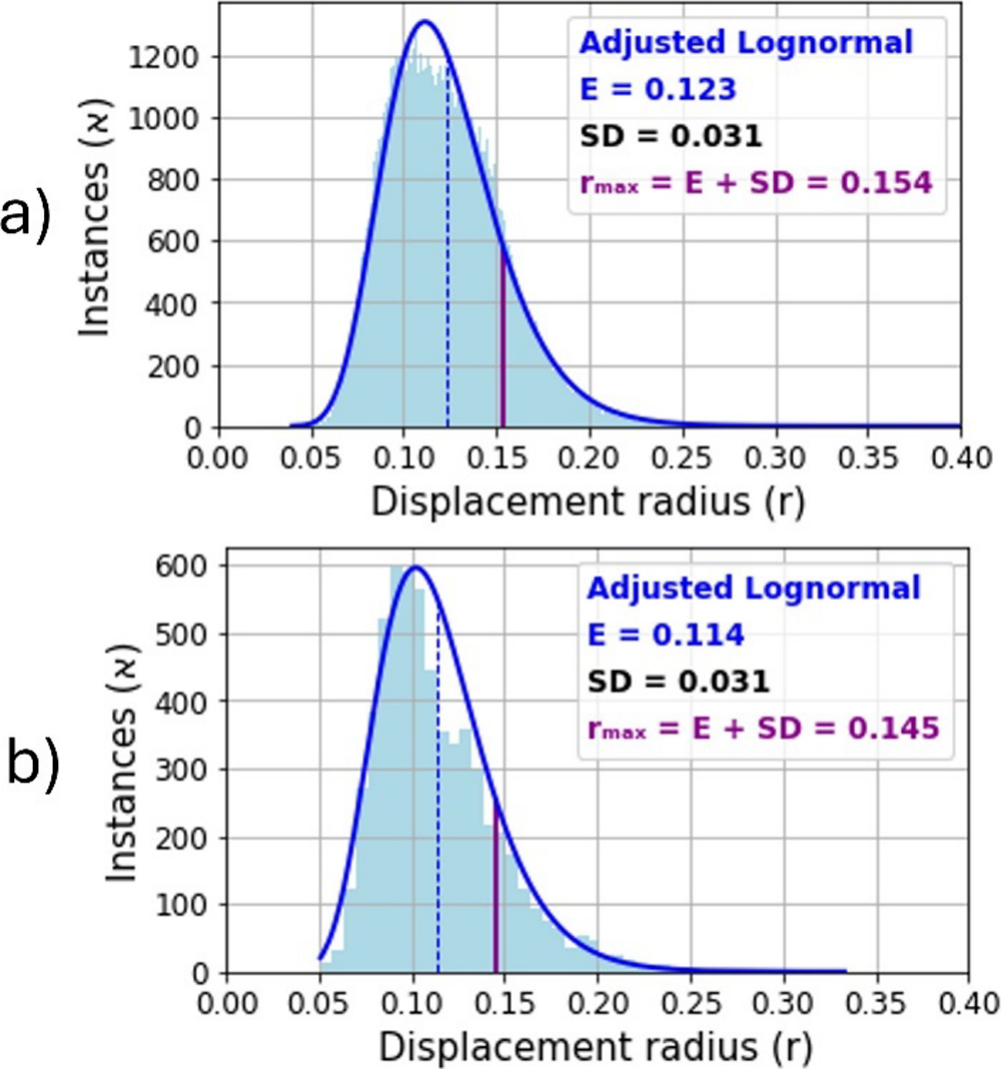
Histograms of isotropic displacement data for CN-6 (a) and CN-7 (b) with a log-normal distribution fit, whose parameters are μ = −2.128 and σ = 0.251 for CN-6 and μ = −2.208 and σ = 0.269 for CN-7. E and SD are the arithmetic means and standard deviations of the respective distributions.

Both graphs exhibit an asymmetric distribution of frequencies, with the greater part of the data concentrated in the interval [0.05, 0.20]. In both cases, statistical analysis indicates that the data can be fitted to a log-normal distribution of the form 

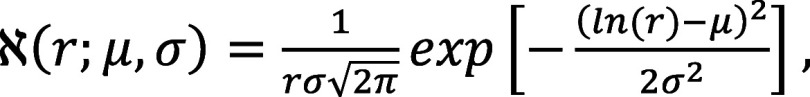

8
where 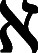 is the probability density, μ and σ are the parameters of the log-normal distribution. The arithmetic mean *E* and standard deviation SD of the log-normal distribution are calculated from μ and σ by the formulas

$$E≡E(\mu ,\sigma )=\exp\left(\mu +\frac{\sigma^{2}}{2}\right)$$ 9

$$\mathrm{SD}≡\mathrm{SD}(\mu ,\sigma )=E(\mu ,\sigma )\times \sqrt{\exp(\sigma^{2})-1}$$ 10

We take the reference value for the maximum isotropic displacement *r*_max_ as the sum of *E* + SD, calculated from the parameters of each adjusted distribution curve. This threshold corresponds to a high percentile of the cumulative distribution function of the isotropic displacements under the log-normal model, specifically ∼85% for the observed σ values in our study, and therefore serves as a conservative bound by excluding a portion of the upper tail, which reduces the sensitivity to measurement uncertainty and outliers. Furthermore, it retains interpretability even if the log-normal model is imperfect in the extreme tail. Consequently, the interval [0,*r*_max_] is predicted by the fitted log-normal model to contain ∼85% of the displacements, with values beyond *r*_max_ treated as extremes.

For hexacoordination, we found the values *E* = 0.123 and SD = 0.031. Thus, for CN-6, we set a maximum displacement value of *r*_max_ = 0.154. For heptacoordination, we found values *E* = 0.114 and SD = 0.031. We thus set the cutoff value for CN-7 to *r*_max_ = 0.145.

With these reference values, we determined which MSMR representations of the 6- and 7-coordinate geometries form groups of thermally indistinguishable polyhedral shapes according to their best pairwise alignments. In our calculations, we considered not only the three-dimensional solids optimized by our algorithm but also the hexa- and heptagonal planar shapes. The lowest RMSD values found for each pair of CP, and the Euclidean separation distance of each aligned vertex pair are shown in Tables S18 and S19 of the Compendium within the Supporting Information.

The indistinguishability relationships of these shapes, as defined in our work, can be visualized by using a graph representation. In this representation, shapes serve as nodes, and each pair, which can be classified as thermally indistinguishable, is connected by an edge. In Figures 7 and 8, we show the graphs we found for CN-6 and 7, respectively.

**Figure 7:**
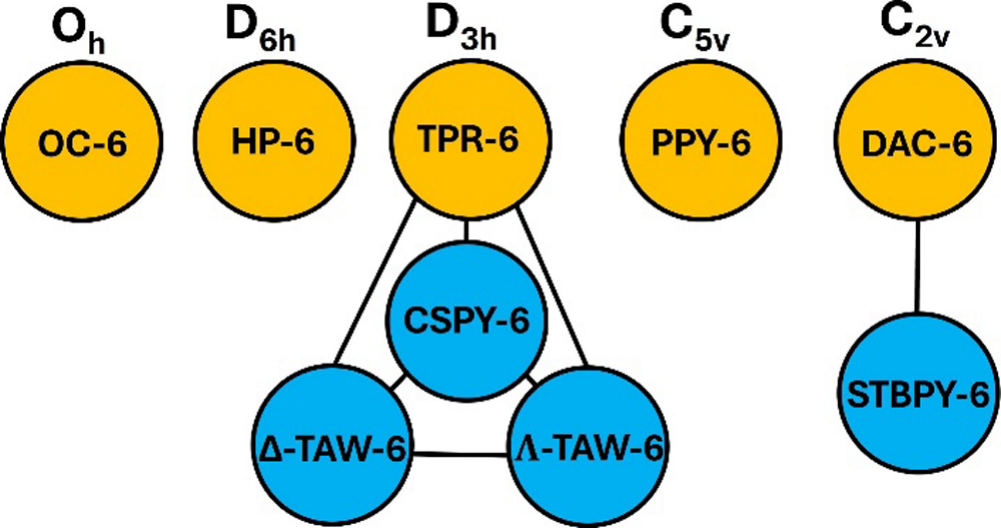
Polyhedral networks for 6-vertex coordination polyhedra.

**Figure 8:**
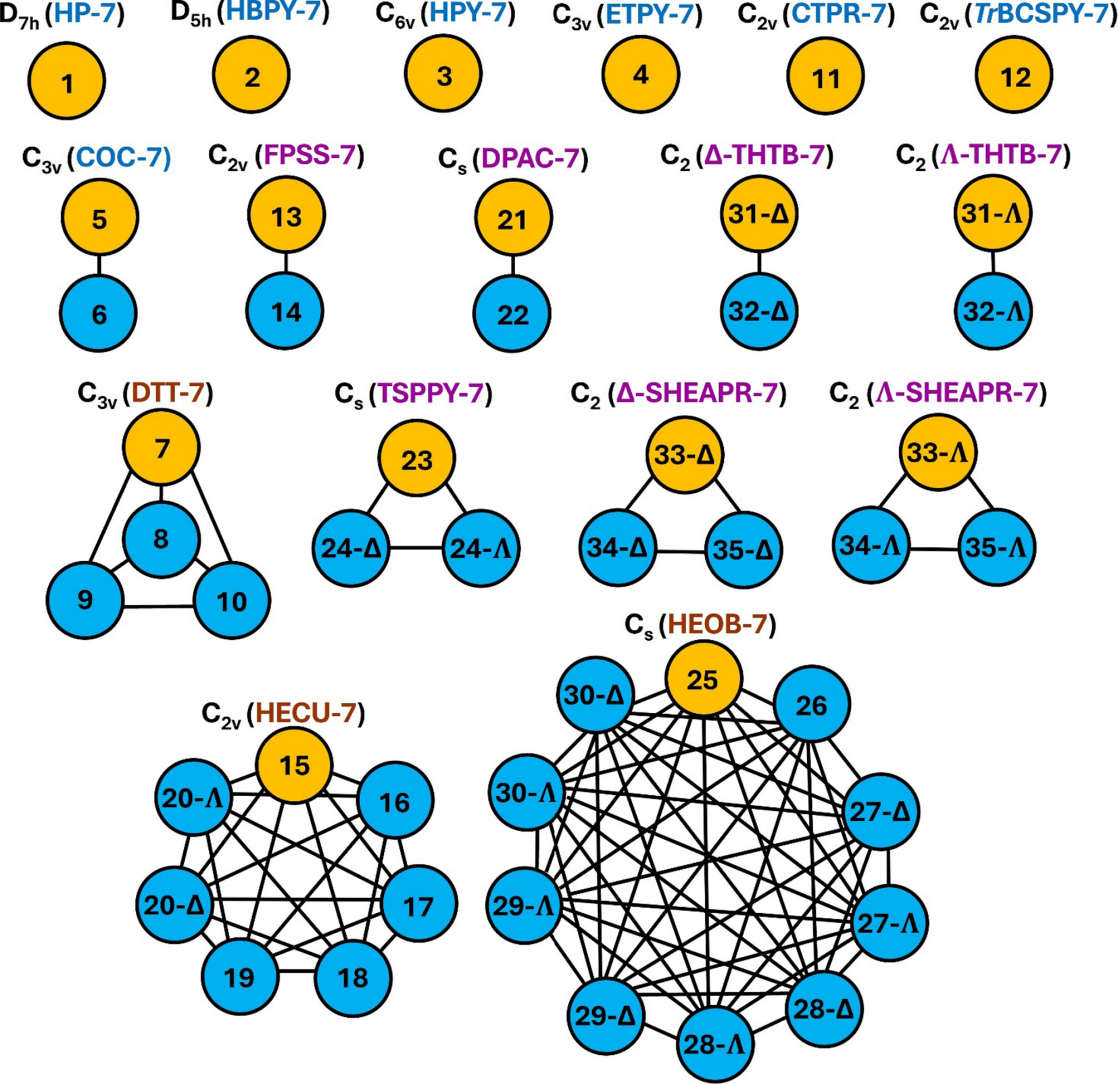
Polyhedral networks for 7-vertex coordination polyhedra.

The patterns indicate that, while some polyhedral shapes are not connected by thermal smearing to any other combinatorially nonequivalent geometry, some polyhedra form thermal smearing clusters. In all cases, these polyhedral clusters form complete graphs *K*_*n*_, where each node represents a polyhedron and every node (with *n* being the total number of nodes) is connected to all others. Thus, completeness implies that any geometry in the cluster can be directly transformed into any other geometry without passing through an intermediate geometry, which is a core criterion in our definition of thermally distinguishable polyhedral shapes. Ergo, all polyhedra within each thermal smearing cluster are all thermally indistinguishable from one another. Therefore, these complete graphs satisfy the requirement of maximal pairwise connectivity. They are perfect, highly symmetric, and exceptionally robust, providing a solid foundation for us to define the complete sets of thermally distinguishable polyhedral shapes, TDPSs, as a novel concept in coordination chemistry.

Molecular vibrations can be regarded as excursions in shape space about symmetry-maximizing minima. Consistent with this view, our crowding-potential landscape exhibited discrete basins rather than a continuum, yielding clustered rather than continuously drifting geometries. When the thermal amplitude and exchange rate of a low-barrier mode carry the structure across the geodesic distance between neighboring minima on the crystallographic time scale, the shapes become experimentally indistinguishable and are assigned to the same TDPS. Thus, dynamics do not erode TDPS distinctions; they provide the mechanism by which some shapes coalesce under experimental conditions, whereas others remain resolvably distinct.

In hexacoordination, the shapes trigonal prism (TPR-6), capped square pyramid (CSPY-6), and the two chiral configurations of the tetragonal antiwedge (Δ/Λ-TAW-6) form a complete graph *K*_4_. Likewise, the skew trapezoidal bipyramid (STBPY-6, also known in the literature as bicapped tetrahedron^106^) and the digonal anticupola (DAC-6) shapes form a thermally indistinguishable pair of shapes.

For heptacoordination, more complex patterns are observed. Five isolated pairs of thermally indistinguishable polyhedral shapes are identified. Three clusters are of the *K*_3_ type. In two of them, the same chiral polyhedral shapes are found, but their enantiomorphic configurations are distinct in each cluster. This indicates that the clustering process can also occur based on the absolute configuration of the polyhedra (Δ or Λ). Complete graphs *K*_4_ and *K*_7_ are also present. A *K*_10_ cluster is the largest found for CN-7.

Based on the patterns observed, we can now define a complete set of TDPSs for a given coordination number, out of its entire topological space of nonisomorphic convex polyhedra.

Thus, we present the following protocol for selecting the representative shape within each of the clusters of thermally indistinguishable shapes:

1. Start by selecting the polyhedral shape with the highest connectivity, that is, the one whose corresponding node has the highest degree, *d*_P_;
2. If two or more shapes have the same degree, *d*_P_, choose the one whose polyhedral graph has the highest number of symmetry elements *h*.
3. If two or more shapes have the same *d*_*P*_ and *h*, choose the one displaying the lowest repulsion Ξ as calculated by the Crowding potential;

The shapes selected by this protocol are those whose nodes have their backgrounds colored in Figures 7 and 8. Thus, for hexacoordination, we obtain 5 TDPSs, while for heptacoordination, we obtain 17, considering the polygonal forms and counting chiral shapes twice, accounting for both Δ and Λ configurations.

The complete sets of TDPSs for coordination numbers 6 and 7 are shown in Figures 9 and 10.

**Figure 9:**
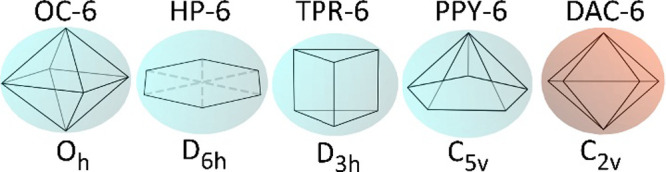
Complete set of 5 thermally distinguishable polyhedral shapes, TDPSs, for coordination number 6. Chemically established shapes are depicted with a blue background, while the one with a deep red-orange background corresponds to a polyhedron recognized in other fields.

**Figure 10:**
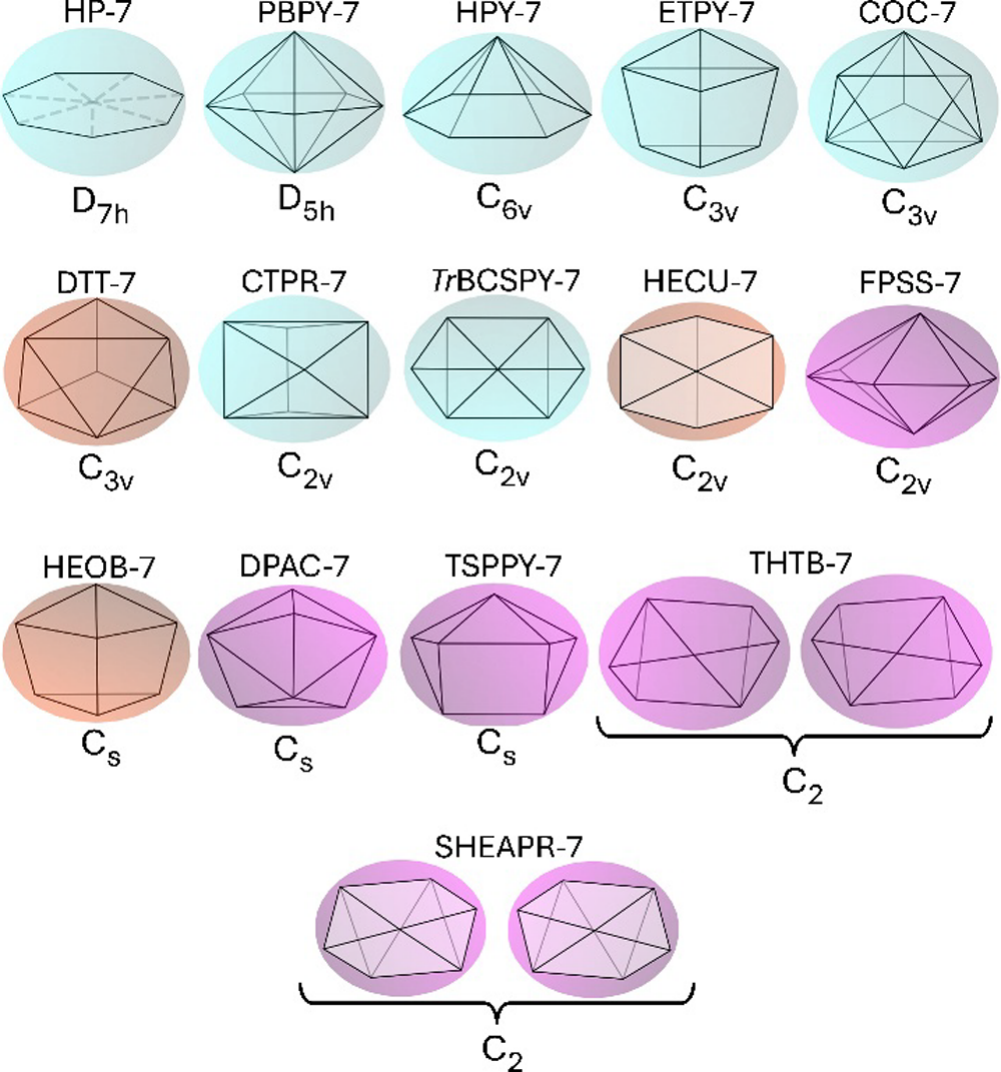
Complete set of 17 thermally distinguishable polyhedral shapes, TDPSs, for coordination number 7. Chemically established shapes are depicted with a blue background, while those with a deep red-orange background correspond to polyhedra recognized in other fields. In contrast, the shapes with a dark magenta background denote polyhedra that are unique to our study, for which we are proposing fresh symbolic abbreviations.

Naturally, as illustrated in Figures 9 and 10, the CP shapes commonly used in the chemical literature and in computational chemistry software are already included as elements in these complete sets of TDPSs. For CN-6, these are the octahedron, the trigonal prism, the pentagonal pyramid, and the planar hexagon. For CN-7, these are the pentagonal bipyramid, the capped octahedron, the capped trigonal prism, the elongated trigonal pyramid, the hexagonal pyramid, and the planar heptagon.^107^ They are all depicted in light blue backgrounds in Figures 9 and 10.

However, the additional forms defined in this work may also occur in nature, as indicated by crystallographic structures, yet they have remained unrecognized as such—a point we will demonstrate later in this article. As a result, these are frequently forced into one of these common shapes and are often labeled as distorted variants. Such an approach, when examined through the lens of the rigor of our work, proves to be incomplete. and leads to incorrect symmetry assignments. In this work, we argue that our newly identified forms, alongside the commonly used ones, constitute a comprehensive set of thermally distinguishable coordination polyhedral shapes. This expanded framework is essential for the accurate description of coordination compounds, particularly those with higher coordination numbers such as lanthanide complexes.

Thus, besides these commonly used polyhedra, our findings therefore require that other shapes also be considered. For hexacoordination, Figure 9, the digonal anticupola (DAC-6, C_2v_). For heptacoordination, Figures 10 and 11 new polyhedra: a diminished trigonal trapezohedron (DTT-7, C_3v_), a *trans*-bicapped square pyramid (*Tr*BCSPY-7, C_2v_), a hemicube (HECU-7, C_2v_), a five-pointed scallop shell (FPSS-7, C_2v_) shape, the hemiobelisk (HEOB-7, C_s_), a digonal pseudoanticupola (DPAC-7, C_s_), a tetragon-substituted pentagonal pyramid (TSPPY-7, C_s_), the tetragonal helicoid tetragonal-base (THTB-7, C_2_), and square hemiantiprismatic geometries (SHEAPR-7, C_2_).

**Figure 11:**
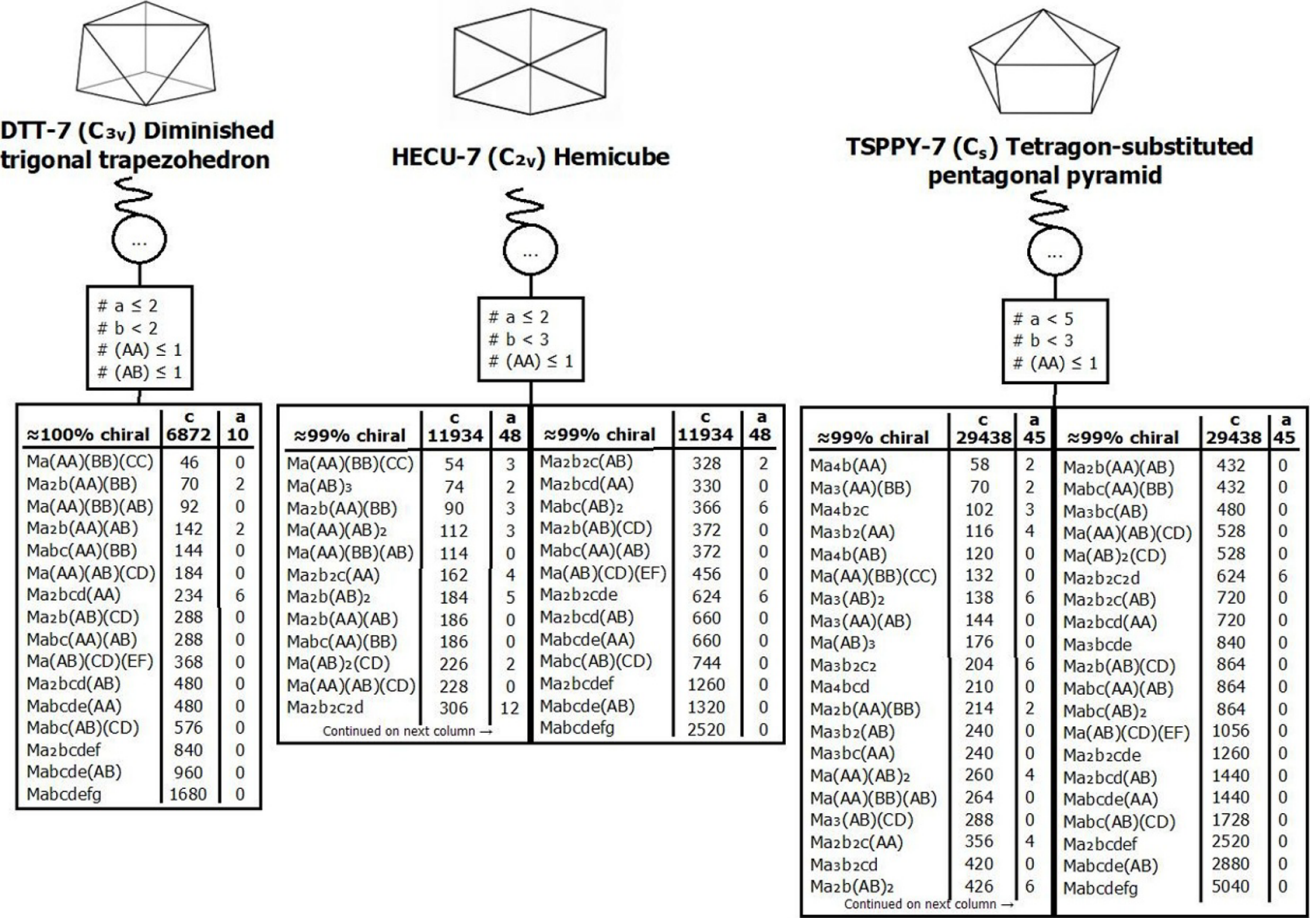
Largest clusters found on the final leaves of the decision trees of the diminished trigonal trapezohedron, DTT-7, hemicube, HECU-7, and tetragon-substituted pentagonal pyramid, TSPPY-7, polyhedral shapes.

Noteworthy, for CN-7, there are chiral polyhedral shapes in the final set of representative CPs: the THTB-7 and SHEAPR-7, both of the C_2_ point group. No asymmetric polyhedron of C_1_ symmetry is found in the CN-6 and CN-7 subsets, indicating all their MSMR representations are thermally indistinguishable to polyhedral shapes containing a symmetry element besides the identity E_1_.

Some of these polyhedra could not be taken as possible shapes for metal complexes if we had followed previous selection criteria presented in the literature that impose minimum symmetry, such as C_2v_,^34^ or limit the configurational search to polyhedra with triangular or quadrilateral faces under the premise of being of lower repulsion.^21,33,34^ In fact, five of the eleven 7-vertex polyhedra added to the subset of common CPs have a lower symmetry than C_2v_: three C_s_ polyhedra and two C_2_ shapes. Besides this, three of these polyhedra have pentagonal faces as polygonal bases. A careful inspection of Table 2 reveals that these shapes have lower potential values than CPs constituted exclusively of triangular faces. For instance, the hemiobelisk (HEOB-7) and the digonal pseudoanticupola (DPAC-7) both have a 5-vertex face, and their potentials are 14.74 and 14.67, respectively, while the skew pentagonal bipyramidal shape (SPBPY-7) has only triangular faces and exhibits a value of 14.84 for the potential.

### Stereoisomer Enumeration in Coordination Complexes

Previously, we have addressed the proliferation of stereoisomers^14^ in high coordination complexes by means of a rigorous combinatorial approach, using Pólya’s Enumeration Theorem.^108^ This theorem offers a powerful tool to tally the total number of stereoisomers of a mononuclear coordination compound^109–111^ by considering each symmetry operation of a given shape of a CP. Accordingly, a cycle index polynomial is constructed that is representative of the symmetry elements of the geometric shape. Upon variable substitutions and further expansion, the resulting polynomial yields coefficients that directly indicate the number of distinct stereoisomers for each possible generic formula. Furthermore, we have shown that this approach allows for the determination of the total number of chiral-at-metal and achiral stereoisomers.^14^

We had also advanced in this previous work^14^ the concept of random coordination ratio (RCR), which represents the relative probability of obtaining each subset of stereoisomers when coordination occurs in a uniformly random manner, that is, in the absence of differential energetic effects. The framework we had previously introduced assigns a unique code to each stereoisomer, as illustrated below for a specific case: {[Ma2(AA)2] DAC-6 C2v a 2 C [3 1 4 5 6 2]}, which indicates, in order, the generic formula of the metal complex, Ma_2_(AA)_2_, a short abbreviation of its shape, DAC-6, the point group of the stereoisomer, C_2v_, a string for the occurrence of coordination chirality (“c” for chiral-at-metal, “a” for achiral), the symmetry number σ of the arrangement, 2, the subset of the stereoisomer, C, which is based on the RCRs, and a permutational vector, [3 1 4 5 6 2], which is unique for each distinct stereoisomer.

Our approach enabled the construction of tables of stereoisomers that allow an easy visualization of the diversity of stereoisomers for each possible composition of ligands in a given polyhedral shape, their diversity of symmetry point groups, and the relative probability of random assembly determined exclusively by combinatorial factors, in accordance with the RCRs.

This protocol was previously applied to well-established shapes in Coordination Chemistry.^14^ In this work, we applied the same approach to further investigate the permutational space of stereoisomers corresponding to the newly defined and introduced coordination polyhedral shapes. Accordingly, for these cases, we addressed the cases of all generic formulae involving mono- and/or bidentate ligands. As such, we identified the respective counts of chiral and achiral arrangements, their associated symmetry point groups, RCRs, and related characteristics. As an example, Table 3 presents an excerpt from the complete stereoisomer table for the hexacoordinate digonal anticupola (DAC-6) geometry. This selection includes cases corresponding to generic formulae with only mono- and/or bidentate ligands identified in the CSD for this shape, as discussed in further detail later in this article.

**Table 3:** Number of Stereoisomers for the Digonal Anticupola (DAC-6) Geometry Corresponding to the Generic Formulae Identified in the CSD as Occurring for This Shape (Subset of the Complete Table Available in the Supplementary Information)­^a^

		set			**RCR**	subsets											
# CSD cases	generic formula	total	c	a		subset	group	χ	σ	RCW	#	subset	group	χ	σ	RCW	#
3	Ma6	1	0	1	1	A	C2v	a	2	360	1						
6	Ma4(AA)	6	4	2	8:2:1	A	C1	c	1	48	4	B	Cs	a	1	48	1
						C	C2v	a	2	24	1						
2	Ma3b3	10	4	6	3:2	A	Cs	a	1	36	6	B	C1	c	1	36	4
2	Ma3b2c	30	20	10	2:1	A	C1	c	1	12	20	B	Cs	a	1	12	10
5	Ma3b(AB)	44	38	6	1:1	A	C1	c	1	6	38	B	Cs	a	1	6	6
1	Ma2b2c2	48	36	12	2:2:1	A	C1	c	1	8	36	B	Cs	a	1	8	6
						C	C2v	a	2	4	6						
4	Ma2b2(AA)	34	30	4	30:2:1	A	C1	c	1	8	30	B	Cs	a	1	8	2
						C	C2v	a	2	4	2						
2	Ma2b2(AB)	66	60	6	1:1	A	C1	c	1	4	60	B	Cs	a	1	4	6
1	Ma2bc(AA)	66	62	4	15.5:1	A	C1	c	1	4	62	B	Cs	a	1	4	4
46	Ma2(AA)2	15	14	1	20:4:1	A	C1	c	1	16	10	B	C2	c	2	8	4
						C	C2v	a	2	8	1						
2	Ma2(AA)(AB)	50	48	2	24:1	A	C1	c	1	4	48	B	Cs	a	1	4	2
95	Ma2(AB)2	55	52	3	44:4:1:1	A	C1	c	1	4	44	B	C2	c	2	2	8
						C’	Cs	a	1	4	1	C″	C2v	a	2	2	2
18	Mab(AA)2	25	24	1	24:1	A	C1	c	1	8	24	B	Cs	a	1	8	1
4	Mab(AA)(AB)	100	100	0	1	A	C1	c	1	2	100						
3	Mab(AB)2	100	98	2	49:1	A	C1	c	1	2	98	B	Cs	a	1	2	2
2	Mab(AB)(CD)	200	200	0	1	A	C1	c	1	1	200						
12	M(AA)3	4	4	0	2:1	A	C1	c	1	48	2	B	C2	c	2	24	2
2	M(AA)2(BB)	10	10	0	8:1	A	C1	c	1	16	8	B	C2	c	2	8	2
10	M(AA)(AB)2	38	38	0	17:1	A	C1	c	1	4	34	B	C2	c	2	2	4
6	M(AB)3	24	24	0	1	A	C1	c	1	6	24						
4	M(AB)2(CD)	72	72	0	1	A	C1	c	1	2	72						

a # CSD cases refers to the number of actual CSD geometries found, whose CSD codes are listed in Table S14 of the Compendium within the Supporting Information; total is the number of stereoisomers for the DAC-6 shape and generic formula, of which c is the number of chiral and a is the number of achiral stereoisomers. RCR is the random coordination ratio. All stereoisomers are classified into subsets according to their point groups and ordered in terms of the product of the number # of distinct stereoisomers by their corresponding random coordination weights, RCWs.^14^ χ identifies chirality of the CPs of all stereoisomers of the subset, with ‘a’ meaning that they are achiral, and ‘c’ meaning that they are chiral; and σ is the rotational symmetry number.^14^

Table 3 indicates that the total number of stereoisomers varies significantly across different ligand compositions, ranging from 1 for Ma_6_ to 200 for Mab­(AB)­(CD). This variability is primarily influenced by both the diversity of ligands and their intrinsic structural variability, with more heterogeneous ligand combinations, e.g., Mab­(AB)­(CD), leading to a higher number of stereoisomers. Nevertheless, the number of chiral stereoisomers ‘c’ shows larger variability than the number of achiral stereoisomers ‘a’. While the number of achiral stereoisomers ranges from 0 to 12, depending on the ligand composition and symmetry constraints, the number of chiral stereoisomers ranges from 0 to 200. Notably, certain formulae, such as Mab­(AA)­(BB), yield exclusively chiral-at-metal stereoisomers. Thus, disregarding energetic effects, a random combinatorial assembly for this shape is more likely to yield chiral arrangements rather than achiral arrangements, as indicated by the RCRs. For instance, for the Mab­(AB)_2_ formula, a chiral C_1_ stereoisomer is 49 times more probable than an achiral C_s_ stereoisomer.

Complete tables for all newly introduced polyhedral shapes are available in the Supporting Information of this article.

### Metal-Centered Chirality and Decision Trees

Recently, we introduced decision tree methodologies to rapidly discern trends in coordination chirality for monometallic complexes,^7^ based solely on polyhedral shape and generic ligand composition. Building upon the enumeration and chirality identification results detailed in the preceding section, we systematically analyzed all possible generic formulae for n-coordinate complexes with mono- and/or bidentate ligands. This comprehensive analysis involved calculating the total number of chiral-at-metal and achiral stereoisomers corresponding to each distinct combination of polyhedral shape and generic formula. Subsequently, we employed decision tree algorithms to establish optimal predictive rules for metal-centered chirality probabilities, explicitly informed by the polyhedral shape and generic ligand combinations. This approach allowed us to pinpoint specific shapes and ligand combinations that significantly influence the presence or absence of chirality at the metal center.

Unlike the well-known case of sp^3^ tetrahedral carbon atoms in organic chemistry, higher coordination numbers commonly found in metal complexes lead to significantly more complex decision-tree structures. Accurately classifying generic formulae into clusters of similar likelihoods of forming either chiral, or achiral metal-centered structures, necessitates multiple classification rules to accommodate this increased complexity.^7^

A comparison between theoretical predictions and the occurrence of coordination chirality in crystallographic structures reveals that, overall, the tendency of higher coordination number complexes to exhibit metal-centered chirality is indeed observed experimentally.^7^

Generic formulae with lower coordination numbers (CNs) of 4 and 5 predominantly lead to achiral metal-centered structures, whereas those with higher CNs (8 and above) tend to favor chiral configurations.^14^ Consequently, the coordination numbers examined in this study, CN-6 and CN-7, represent a transition regime between these two tendencies.

For example, our statistical analysis^7^ of the CSD crystallographic database indicates a shift in the preferred arrangement (achiral or chiral-at-metal) in CN-7 complexes, depending on the CP adopted. In the common pentagonal bipyramidal geometry, achiral structures predominate, whereas in the more frequently observed capped trigonal prismatic and capped octahedral geometries, chiral-at-metal structures prevail.^7^

Similarly to our previous work,^7^ we have constructed decision trees for the new polyhedral shapes introduced in this article, adhering to the same criteria. We note that, in general, the polyhedra of these new shapes display lower symmetries than the polyhedra traditionally known in coordination chemistry. For instance, the familiar 6-vertex ideal octahedral and trigonal prismatic geometries have point groups of O_h_ and D_3h_, respectively, and their corresponding number of symmetry elements (*h*) are 48 and 12, half of which are chirality-reversing (reflections, inversion, and improper rotations). On the other hand, the new hexacoordinate polyhedron added, the digonal anticupola, has C_2v_ symmetry, thus *h* = 4, and only two isometries of the second kind (two perpendicular mirror planes). For heptacoordination, the majority of the new polyhedra have symmetry point groups with *h* = 2; therefore, these geometries have only one other isometry besides the identity operator (E). In two cases (square hemiantiprism and tetragonal helicoid with tetragonal base geometry), this symmetry operation is of the first type: a 2-fold rotational axis. Consequently, the corresponding polyhedra are intrinsically chiral with the C_2_ point group. Otherwise, the isometry is a mirror plane, and the corresponding polyhedra display the point group C_s_. The remaining new 7-vertex polyhedra are more symmetric and display C_nv_ point groups (n = 2 or 3, with *h* = 4 and 6, respectively), but their numbers of symmetry elements are far lower than the one for the predominant D_5h_ pentagonal bipyramid (*h* = 20).

Chiral-at-metal arrangements of ligands are formed in achiral polyhedra by breaking their handedness-inverting symmetry elements, which can be achieved either by increasing the diversity of ligands or by coordinating bidentate ligands, thereby conveying helicoidal-like layouts. The lower symmetry of these new polyhedral shapes makes coordination chirality easier to reach. In the decision trees, this is reflected in the formation of clusters where all generic formulae exhibit p­(**c**)—the probability of randomly assembling a chiral-at-metal stereoisomer in a CP—very close to or exactly 100%. Usually, the lower the symmetry of the polyhedron, the larger its final decision tree leaves can be. As examples, the diminished trigonal trapezohedron (DTT-7), the hemicube (HECU-7), and the tetragon-substituted pentagonal pyramid (TSPPY-7) 7-vertex polyhedra have, in sequence, C_3v_, C_2v_, and C_s_ point groups, and their largest clusters contain, respectively, 16, 25, and 40 generic formulae (all gathered on a terminal node) out of 54 possible compositions of ligands, as shown in Figure 11. On the other hand, the largest final leaf in the pentagonal pyramid decision tree contains 13 generic formulae.^123^ Therefore, we expect these new polyhedral shapes to be more likely to lead to chiral-at-metal stereoisomers for a given generic formula than their more symmetric and already known CN-7 shapes.

### Evidence of the New Polyhedral Shapes in Metal Complexes

Now that we have a comprehensive set of thermally distinguishable polyhedra for 6- and 7-coordinate compounds, we can verify the occurrence of these shapes, including the newly introduced ones, in structures reported in crystallographic databases. For this purpose, we applied these reference shapes to the previously downloaded set of 42,000 crystallographic structures to identify the best-fitting shape for each metal complex geometry determined crystallographically. Further, for coordination complexes having only mono- and/or bidentate ligands, we identified the stereoisomer code that characterizes their overall stereochemistry. As previously mentioned, these codes represent the ideal point-group symmetry of the stereoisomer, describe the CP, indicate possible metal-centered chirality, and specify the rotational symmetry number, among other properties. Accordingly, our results for CN-6 and 7 are shown in Table 4.

**Table 4:**
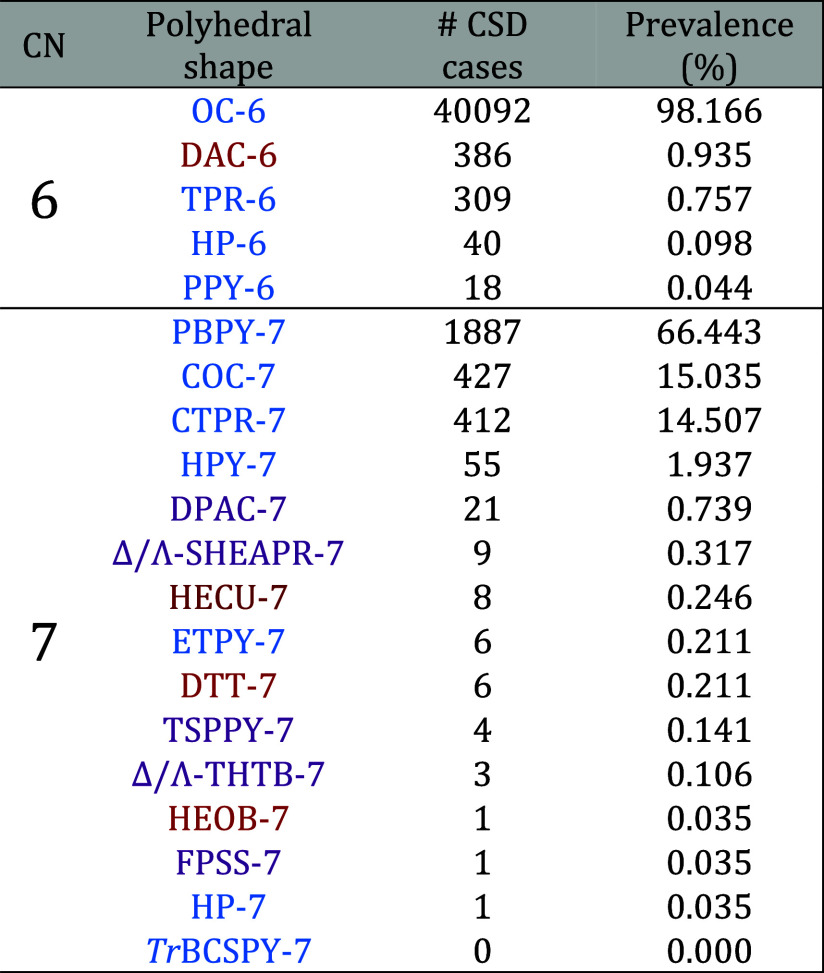
Prevalence of 6- and 7-Vertex Coordination Polyhedra Among Metal Coordination Compounds in the CSD, Including Those with Polydentate Ligands and Polymetallic Complexes

The data show that, so far, all 6- and 7-vertex CPs that constitute our complete reference sets of shapes can be found in coordination complexes deposited in crystallographic databases, except for the *trans*-bicapped square pyramidal geometry (TrBCSPY-7). In 6-coordinate complexes, the predominant shape, even after the addition of the new CP DAC-6, is still the octahedron (OC-6), with more of 98% of these structures exhibiting that shape. Remarkably, we found that the DAC-6 digonal anticupola shape occurs more frequently (386 cases) than the well-known TPR-6 trigonal prism (309), the HP-6 planar hexagonal (40), and the PPY-6 pentagonal pyramid (18) shapes.

Further support for the validity and necessity of the newly defined TDPSs comes from an independent comparison with established shape analysis tools. We evaluated the CPs of the metal complexes listed in Table 4—excluding, for the hexacoordinate set, those that are not polymetallic and possess polydentate ligands, while including all heptacoordinate cases—using both CShM (via SHAPE^46^) and the Continuous Symmetry Operations Method (CSOM).^112^ For the CShM analysis, our new TDPSs were implemented in SHAPE 2.1 (see Supporting Information), which then consistently identified DAC-6, FPSS-7, DPAC-7, and others as the lowest-CShM fits (<3), fully supporting their structural distinctiveness (see Tables S14 and S15). The CSOM evaluation, performed on trimmed CPs containing only the central metal atom and its directly coordinating atoms, likewise showed strong agreement with our assignments: DAC-6 cases were predominantly classified within the expected C_2v_ point group (see Table S16)—a symmetry absent from established hexacoordinate shapes—whereas the new 7-coordinate TDPSs were generally assigned to their corresponding point groups (see Table S17), with the few lower-symmetry classifications being readily reconcilable with our RMSD assignments through thermal smearing. This significant agreement among the RMSD,^101^ SHAPE,^46^ and CSOM^112^ studies supports the validity and relevance of identifying the novel TDPSs presented in this study.

As these findings illustrate, expanding the set of reference CPs to include the thermally complete set of TDPSs proposed here offers a powerful means to refine the structural analysis of coordination complexes, enabling more accurate symmetry assignments and a comprehensive description of their geometrical diversity.

For instance, Pedrick et al. synthesized a homoleptic thorium anionic complex of formula [Th­(C_6_H_5_)_6_]^2–^, where C_6_H_5_^–^ is the phenyl ligand.^47^ Two lithium compounds were separately employed as countercations: [Li­(DME)_3_]^2+^, where DME stands for dimethyl ether, and [Li­(THF)­(12-crown-4)]^2+^, where THF is the tetrahydrofuran and 12-crown-4 is a crown-ether ligand. In the first compound, with [Li­(DME)_3_]^2+^ working as countercation (CSD refcode HABLEE), the thorium complex geometry is a “near perfect octahedron”,^47^ with its CShM for this polyhedron measuring 0.03.^47^ However, the authors point that the characterization of the geometry of the second thorium compound, with [Li­(THF)­(12-crown-4)]^2+^ as countercation (CSD refcode HABLII), is challenging, since its structure exhibits high values of distortion to both the common octahedral and trigonal prismatic shapes, according to the CShM approach: their distortion values measure 4.87 and 6.17 to their reference shapes, respectively.^47^ Both values are bigger than the limit value of 3 units mentioned by Alvarez. Lacking any other better reference shape, the authors describe this second thorium complex, shown in Figure 12a, as having a “severely distorted octahedral geometry”, lying in some intermediate point between the two shapes and out of the Bailar trigonal twist pathway.^47^ Nonetheless, the application of our 6-vertex polyhedral set indicates that our reference digonal anticupola (DAC-6) geometry can promptly describe this coordination complex, with a CShM distortion measure of 1.351, below the maximum threshold value of 3 units.^1^ The RMSD value measured for this same complex using the Marques et al. algorithm^101^ was 0.121, below the value of 0.154 established in an earlier section of this paper.

**Figure 12:**
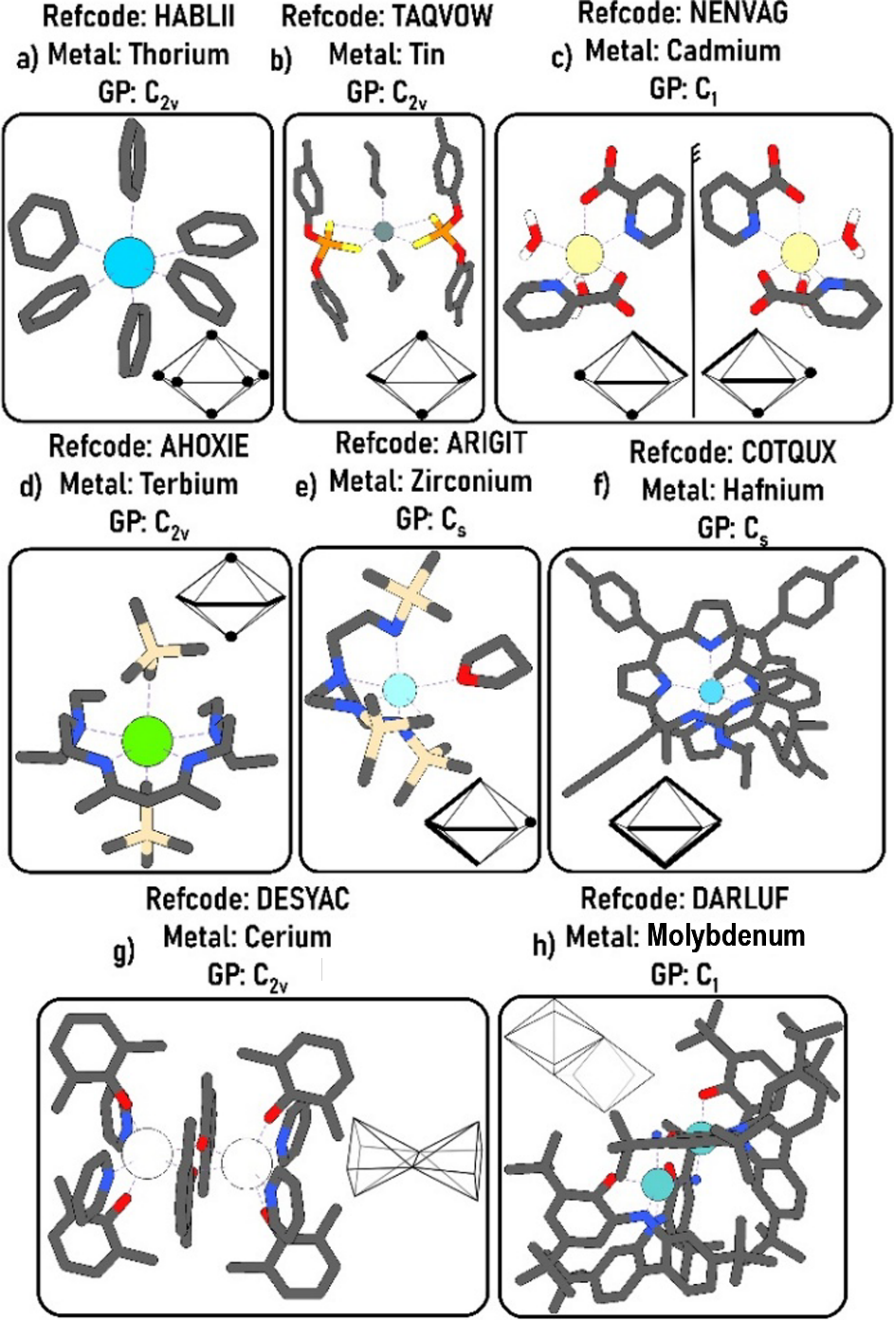
Case examples of digonal anticupola geometry in 6-coordinate metal complexes with various kinds of ligands.

The DAC-6 geometry is common on tin complexes of the generic formula Ma_2_(AA)_2_. In this composition, the two monodentate ligands usually occupy opposite vertices of the square base of the shape, whereas the bidentate ones, often of small four-membered chelate rings, span the edges connecting the square polygonal base to the top digonal base of the anticupola, as shown in Figure 12b, whose arrangement has an ideal C_2v_ symmetry. The remarkable feature regarding the predominance of the stereochemical arrangement in Figure 12b is that, for this shape and ligand composition, this is the only possible achiral stereoisomer out of 15 conceivable ones. The remaining 14 theoretically possible isomers have either C_1_ or C_2_ point groups. Considering the RCRs for this shape and generic formula, a chiral-at-metal stereoisomer would be expected to be significantly more likely. In fact, the RCRs indicate that a chiral arrangement in this case is 24 times more probable than an achiral C_2__v_ isomer in this geometry. Hence, this deviation from our RCRs suggests that other factors, most likely energetic ones, also play a role in determining this predominant stereoisomer formed during the self-assembly process of these coordination complexes.

This stereochemistry of C_2v_ symmetry is frequently described in the literature of tin compounds by means of the skew trapezoidal bipyramidal geometry (STBPY-6),^113–115^ which is a polyhedral shape thermally indistinguishable from the digonal anticupola shape, according to our results.

We point out, however, that aside from this metal and ligand composition, this polyhedron has also been identified in metal complexes with ligands of varying symmetries and denticities (mono-, bi-, and polidentates of diverse types; see Figure 12a–f) and employing different metallic elements from all blocks of the Periodic Table (s, p, d, and f). We have also found that this shape occurs more frequently in polynuclear metal compounds (Figure 12g,h). In this case, unlike the Platonic octahedral geometry, the low symmetry of the polyhedron and the irregularity of its faces allow the junctions of the coordination polyhedral units—each representing a metal complex nucleus—to form different edge-stacked polyhedral patterns. Furthermore, for some cases of this shape, the compound is chiral-at-metal, as in Figure 12c.

Because the DAC-6 TDPS has largely gone unrecognized in metal complexes, explicitly identifying it corrects site-symmetry assignments and yields more reliable spectroscopic interpretations. As an illustrative case, the anticlastic isomer of [M­(ndt)_3_]^−^ (M = Nb, Ta) reported by Tatsumi et al.^116^ is classified within our TDPS framework as DAC-6, whose ideal polyhedral symmetry is C_2v_. Coordination by three bidentate ligands lowers the molecular site symmetry to that of C_2_, which is consistent with the crystallographically observed enantiomeric pair. This C_2v_ → C_2_ reduction removes mirror planes and lifts stretching-mode degeneracies, accounting for the six distinct IR-active M–S stretching fundamentals in the far-IR and crystallographic inequivalences without invoking octahedral distortions,^116^ thereby providing a more accurate basis for spectroscopic interpretation.

Let us now turn to consider heptacoordination. The pentagonal bipyramid (PBPY-7), the capped octahedron (COC-7), and the capped trigonal prism (CTPR-7) are the most prevalent CP shapes across the set of analyzed CSD structures, according to Table 4. A qualitative inspection of the unit sphere coordinates with CSD frequencies (Table 4) indicates that, for largely isotropic metal sites (typical of Ln and alkali ions), the most prevalent CN-7 geometries are those that distribute ligand directions as evenly as possible—maximizing the smallest pairwise angle—and that avoid large “empty caps” on the sphere, which would otherwise invite an eighth donor. Shapes such as PBPY-7 satisfy both criteria and therefore dominate the database. On the other hand, topologies with uneven coverage or pronounced voids are intrinsically fragile for CN-7 and may either relax toward better-packed TDPSs or invite an eighth ligand. Absence of TrBCSPY-7 in metal coordination compounds (even when we increase the threshold *R*-factor to ≤10) is thus unsurprising, as it packs many directions into a single hemisphere, features that increase same-side crowding, while leaving a comparatively large unoccupied spherical patch. That makes distortion toward a neighboring TDPS, and even the addition of a further ligand, more favorable than stabilizing the ideal TrBCSPY-7 arrangement. Nevertheless, although examples of structures adopting other shapes are considerably less common, we identified some—particularly the new polyhedra added to our reference database—in compounds where interactions with the metal center are predominantly electrostatic, such as those involving lanthanide and s-block metals (Figure 13).

**Figure 13:**
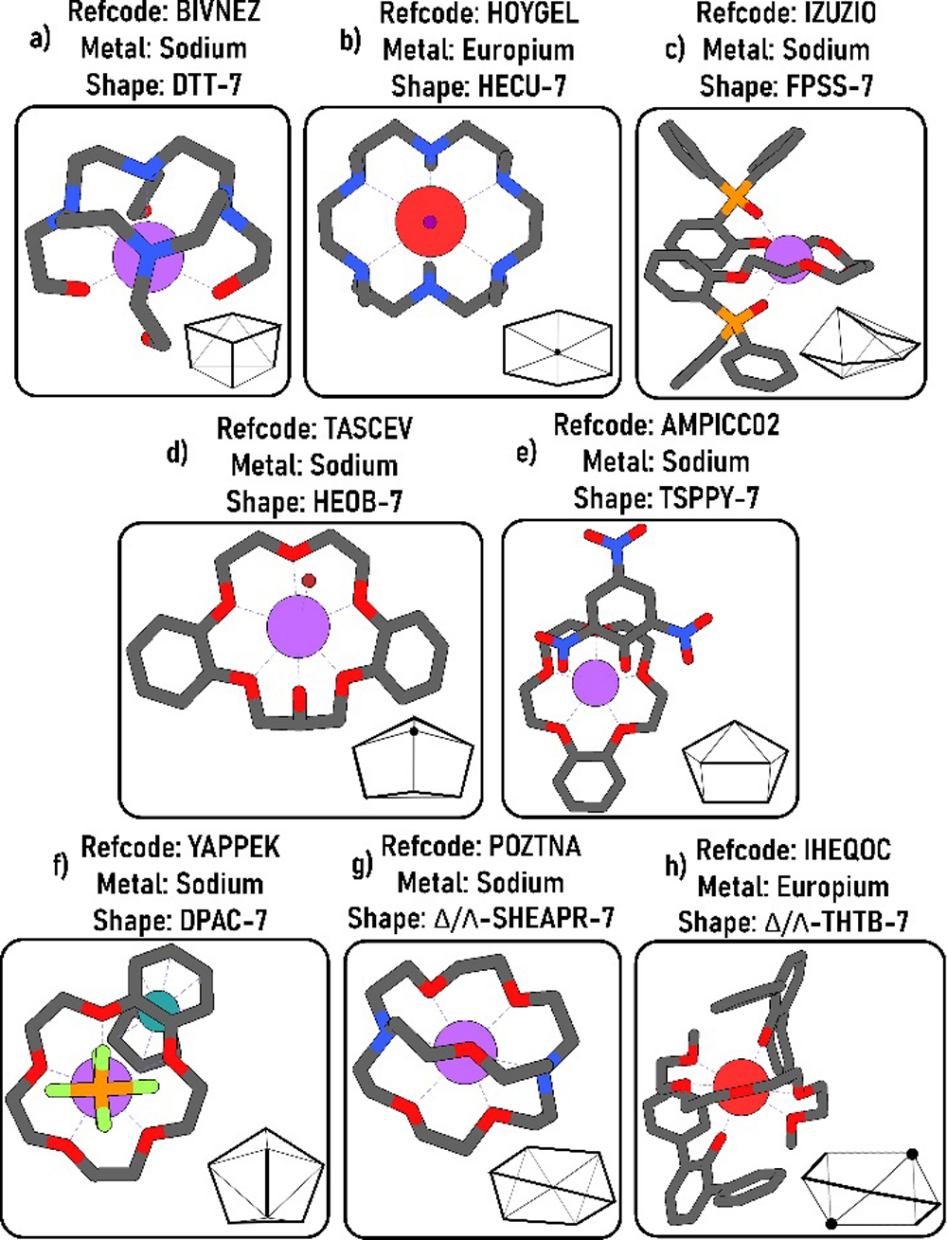
Examples of other 7-vertex coordination polyhedral shapes in metal compounds.

The coordination chemistry of Group 1 and Group 2 metals is less studied due to their lack of paramagnetic properties, colorlessness, strong ionic character, and low stability in aqueous media.^39,117^ Moreover, the high reactivity and hard acid character of these metals require careful ligand design to prevent competition with hard bases. The most effective strategy to address this challenge is the use of polydentate ligands that encapsulate the metal center,^117,118^ as in crown-ethers, cryptands, and their derivatives.

As a result, s-block metals behave similarly to lanthanides on acting like a charged sphere^37,38^ with minimal orbital interaction,^39^ resulting in a lack of directionality. Consequently, the stereochemistry of their compounds is largely influenced by electrostatic and steric factors as well as metal ion size, opening up possibilities of other shapes. In fact, we noticed that in almost all cases, these shapes are found, the corresponding complexes contain polydentate ligands. In other words, when a 7-coordinate complex has only mono- and/or bidentate ligands, its CP shape is in general either the PBPY-7 pentagonal bipyramid, the COC-7 capped octahedron, or the CTPR-7 capped trigonal prism. These shapes have the lowest potential, as calculated by our model for a [ML_n_] complex. The inclusion of polydentates might lead to various geometrical restraints, facilitating the occurrence of the less prevalent shapes.

To illustrate this, let us consider crown-ether ligands that are commonly coordinated to s-block metals. Their highly flexible structures make them adaptable to various CPs on metal complexes.^118^ As an example, the 15-crown-5 ligand has a good size fit to the lithium ion, forming 6-coordinate monocationic compounds of type [Li­(15-crown-5)­(monodentate)]^+^. In these complexes, the structure can adopt either a DAC-6 digonal anticupola or a PPY-6 pentagonal pyramid geometry, as shown in Figure 14a,b. When the shape is a PPY-6 pentagonal pyramid (Figure 14a), the crown-ether lies on the pentagonal base of the polyhedron, with the oxygen donor atoms being all approximately coplanar and the monodentate ligand at the axial axis. Otherwise, when the digonal anticupola occurs (Figure 14b), the macrocycle structure is bent, with the different oxygen atoms lying on distinct planes.

**Figure 14:**
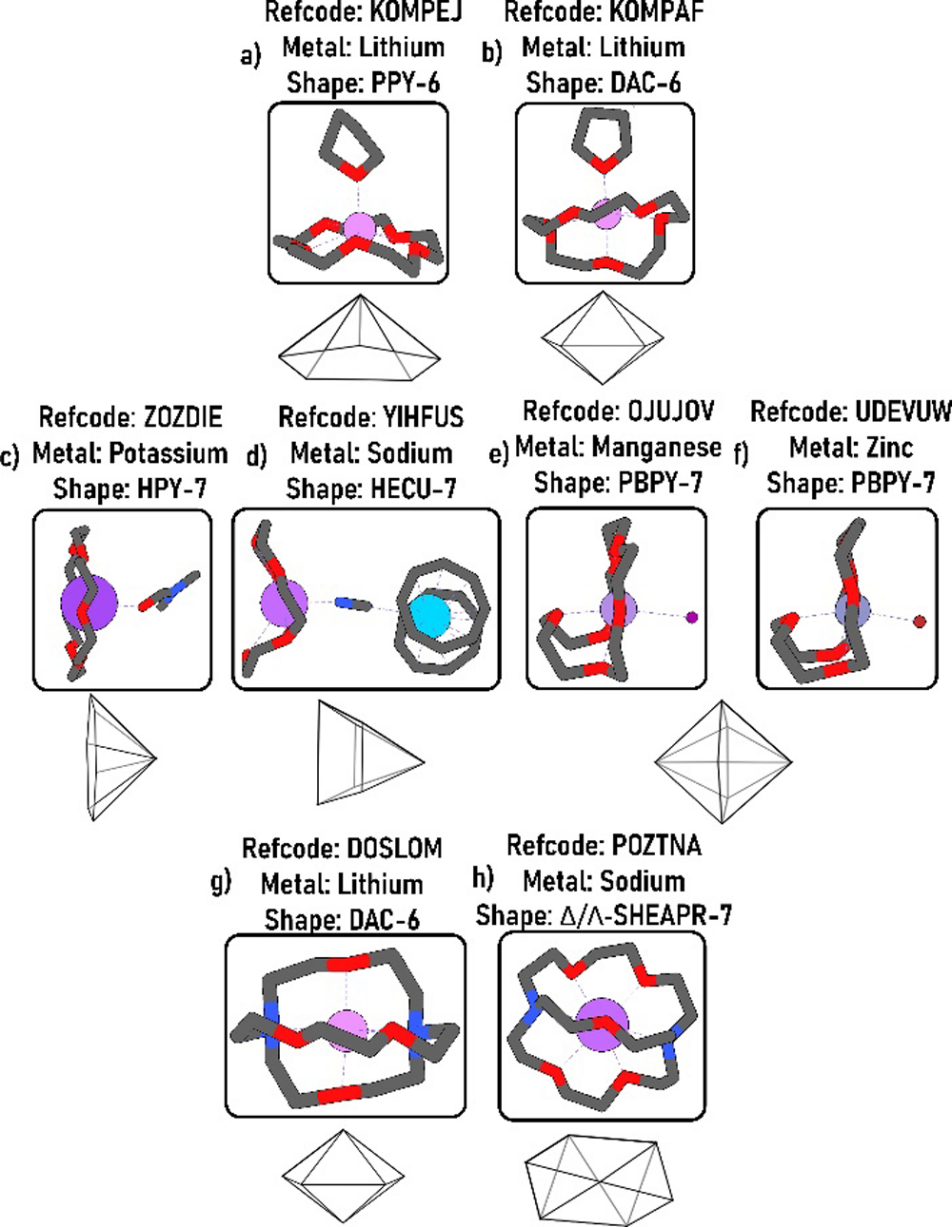
Diversity of coordination polyhedra on crown-ether and cryptand metal complexes.

Let us now turn to the 18-crown-6 ligand. This macrocycle is often coordinated to potassium and sodium to form 7-coordinate complexes whose set of ligands consists of the hexadentate crown-ether plus a monodentate ligand. In these compounds, the CP appears to be influenced by the size of the metal ion. Potassium has a larger radius than sodium, which allows the 6 oxygen-coordinating atoms of the polydentate crown-ether ligand to lie roughly on the same plane. Thus, a 7-coordinate complex of type [K­(18-crown-6)­(monodentate)]^+^ exhibits a hexagonal pyramidal geometry (Figure 14c). On the other hand, an analogous complex with a smaller sodium ion as the metal center exhibits a distorted crown-ether conformation. The macrocycle is bent, the donor atoms are out of the plane, and the shape identified is a hemicubic geometry HECU-7 (Figure 14d). Curiously, if the metal ion is a d-block one with a radius shorter than those of potassium or sodium ions, such as manganese or zinc ions, then the CP is the common PBPY-7 pentagonal pyramid (Figure 14e,f). In this geometry, five oxygen atoms are placed on the vertices of the pentagonal base, while the sixth occupies one of the axial sites of the geometry.

Let us now turn to macrocycle ligands like the cryptands that are capable of encapsulating metal ions in a three-dimensional manner, more intricate than the simpler ring structure of crown ethers. Cryptands consist of nitrogen atoms as anchor points connected by three oxygen-containing ether chains, forming a cage-like structure. The nomenclature of the cryptands reflects the number of oxygen atoms in each of the three distinct bond pathways between the nitrogen atoms. For instance, in a [2.2.1]-cryptand, two pathways contain two oxygen atoms each, while the third pathway contains only one oxygen atom.

In the case of cryptands, the number of coordinating atoms might also dictate the CP shape assumed by the structure. As an example, when the [2.1.1]-cryptand is bound to a lithium ion, the resulting six-coordinate complex exhibits a DAC-6 digonal anticupola geometry, whereas a [2.2.1]-cryptand coordinated to sodium yields a seven-coordinated compound that exhibits a chiral Δ/Λ-SHEAPR-7 square hemiantiprismatic shape.

As in CN-6, identification of CPs on 7-coordinate complexes using an expanded polyhedral database also helps to clarify the overall arrangement of the ligands around the metal center. Recently, Bokouende and co-workers (2024)^52^ reported a series of divalent europium and samarium compounds of varying coordination numbers and ligands, including complexes of type Ln­(**L3**)­X_2_ and [Ln­(**L3**)­X]­X, where **L3** is the hexadentate macrocyclic hexamethylhexacyclen, an analogue of the 18-crown-6 but with nitrogen as coordinating atoms, and X is a halide (either bromide, Br^–^, or iodide, I^–^). The authors investigated the effects of the steric bulkiness of the macrocycle and the halide ion size on the molecular structures of the coordination complexes. Divalent lanthanide complexes with 18-crown-6-like ligands and halides are usually octacoordinated,^119–123^ with 2 halogen ions bound to the metal center, and the commonest CP found for these molecules is the hexagonal bipyramid (HBPY-8). In this polyhedron, the macrocyclic ligand occupies the vertices of the 6-vertex base, while the halides span the axial sites. Let us now focus on the Eu­(**L3**)­X_2_ and [Eu­(**L3**)­X]­X complexes reported by the authors, with europium as the metal center.^52^ When the halide is a bromide, of a smaller ionic radius size, the compound has CN-8 (CSD refcode HOYKUF), its formula is Eu­(**L3**)­Br_2_, and the authors describe its geometry as a “distorted hexagonal bipyramid”.^52^ On the other hand, when the halide is the iodide ion, of a larger ionic radius, the authors point out that the corresponding complex (CSD refcode HOYGEL) has a folded conformation structure, with the 6 nitrogen donor atoms of the macrocyclic ligand not all occupying the same plane^52^ (see Figure 13d) The conformation becomes similar to the 18-crown-6 deformation found on [Na­(18-crown-6)­(monodentate)]^+^ compounds (see Figure 14d), where the structure of the crown-ether is distorted. This folding process prevents the europium atom from being coordinated by a second halide ion on the same side that the polydentate ligand structure is bent. As a result, the complex is 7-coordinate with formula [Eu­(**L3**)­I]^−^ instead of being 8-coordinated with a HBPY-8 hexagonal bipyramidal geometry. Consequently, only one iodide ion coordinates to the lanthanide metal center, but on the opposite side, where the polydentate ligand is found, the other iodide working as a counterion.

CShM calculations performed by the authors for common 7-vertex polyhedra in Coordination Chemistry reveal that the structure of this compound of CN-7 has a high degree of distortion for their ideal reference shapes (a 6.44 value for the capped octahedron, 8.05 for the capped trigonal prism, 8.99 for the hexagonal pyramid, 12.46 for the pentagonal bipyramid, 18.22 for the elongated trigonal pyramid, and 33.73 for the hexagonal planar geometry).^52^ The structure is thus described by the authors as having a “distorted capped octahedron”^52^ geometry. On the other hand, our calculations using our set of TDPSs reveal the [Eu­(**L3**)­I]^−^ ion is better described by a HECU-7 hemicubic shape, with a CShM distortion value of 0.607 as we measured, which indicates that the geometry of this complex has “significant but small distortions” to the reference polyhedron, according to Alvarez guidelines to interpret CShM values.^1^ The RMSD value measured using the Marques et al. algorithm^101^ was only 0.055 for the HECU-7 shape, well below our previously defined cutoff value of 0.145, indicating compliance with our pre-established criteria as presented in this article.

## Conclusions

In the chemical literature, it is acknowledged that CPs derived from crystallographic structures sometimes do not fit precisely into the standard polyhedral shapes traditionally used for their description. As a result, they are commonly referred to as severely distorted versions of the usual ones. Consequently, these structures are inadequately characterized, which typically leads to improper symmetry assignments and thus to potentially incomplete descriptions of coordination chemistry phenomena.

To solve this issue, we first explored the mathematical literature to establish a complete set of polyhedra forming a basis that could span the entire space of convex polyhedra relevant to coordination chemistry. Convex polyhedra are solids bounded by flat surfaces, containing neither ’holes’ nor ’inward folds’, whose skeletons correspond to a specific class of graphs, according to Steinitz′s theorem, with a finite number of graphs for each number of vertices. Steinitz’s theorem thus provides the mathematical foundations of our work.

Accordingly, we retrieved from the House of Graphs the topologies corresponding to this specific class of graphs and first constructed two-dimensional drawings of each of the polyhedron’s graphs. These drawings were then lifted into three dimensions and transformed into a representation in which the edges are tangent to a sphere, using Hart’s algorithm. Subsequently, the vertices were scaled radially to lie on the unit sphere. Finally, the points were redistributed across the sphere using a version of our previously defined crowding potential to ensure a more uniform distribution. We then arrived at the geometrical structures of all such possible polyhedra for coordination numbers 6 and 7, always respecting the topologies and symmetries of the original graphs.

However, the number of topologically distinct polyhedra in the mathematically complete set, although small for the lower coordination numbers (4–5), increases rapidly with higher coordination numbers, reaching over six million at coordination number 12—a figure clearly unrealistic for meaningful stereochemical analysis. Thus, these very large numbers, which constitute a mathematically complete set, had to be substantially reduced to yield a subset justified in the context of coordination chemistry. For this reduction to be principled, it must be firmly grounded in the chemical and physical realities of the complexes and preserve the notion of a minimally representative complete set, even though we deliberately pruned it according to criteria consistent with crystallographic principles.

As one might intuitively expect, as the number of polyhedra grows with increasing coordination numbers, the geometric differences among many of them become progressively smaller, eventually becoming indistinguishable within experimental error—likely as a consequence of their being inscribed, as constructed, in the unit sphere to exhibit maximum symmetry and minimal repulsion.

Since the crystallographically determined vertex coordinates of these polyhedra always lie within thermal ellipsoids, we observed that many apparently distinct polyhedra can easily be interconverted by slight shifts of their vertex positions within these ellipsoids. Accordingly, we found that these large sets of polyhedra can be grouped into subsets whose members are effectively equivalent within the thermal uncertainty. We then established a set of criteria to select a single geometry from each subset as its representative. Finally, we refer to this collection of representative polyhedra as a complete set of thermally distinguishable polyhedra.

In this article, we demonstrate that a complete geometric description of coordination compounds with CN-6 requires five thermally distinguishable shapes: four polyhedra and one polygonal. Among these four polyhedral shapes, one—the digonal anticupola (DAC-6)—to our knowledge, has not been previously described in coordination chemistry, at least not in any widely recognized manner. We then scanned the CSD and unexpectedly found that DAC-6 is indeed very prevalent in coordination compounds, present in six-coordinated complexes of essentially all metals considered. In fact, we identified more cases of DAC-6 shapes (386 in total) than the combined number of cases found for the other well-recognized CN-6 shapes: TPR-6, PPY-6, and HP-6. Definitely, DAC-6 is truly a very important shape in the chemistry of hexacoordinated compounds.

Likewise, for the description of coordination compounds of CN-7, we could reduce the mathematically complete set of 34 polyhedra to 14 thermally distinguishable polyhedra, of which two are chiral polyhedra and therefore have, each, a pair of nonsuperimposable geometries: one Δ and one Λ. Together with the HP-7 heptagon polygon, these are all the shapes that are needed for a complete description of the 7-coordinated compounds.

We also searched the CSD for the nine new CN-7 shapes and, with a single exception, identified representative compounds for each. These were predominantly found among alkaline and lanthanide metal ion complexes containing crown ethers, cryptands, and similar ligands, whose coordination bonds are essentially electrostatic and thus nondirectional. These unconventional geometries are largely determined by stereochemical interactions between ligands.

In conclusion, by laying out a complete set of thermally distinguishable polyhedral shapes, we aimed to provide chemists with a solid, easy-to-use framework for rigorously assigning and comparing the geometries of metal complexes. These results are intended to contribute to a deeper understanding of the intrinsic properties. Each TDPS category represents a set of thermally interconvertible geometries that are experimentally resolvable and characterized by a distinct symmetry profile. These distinct TDPS classes provide robust symmetry templates, replacing the conventional practice of describing them merely as distorted variations of the traditional ideal shapes. These profiles serve as fundamental inputs for ab initio or point-charge crystal-field parameter calculations, which can then be integrated into angular overlap or ligand-field models. Such integration facilitates the prediction and interpretation of transition-metal d–d splitting patterns and the associated electronic structures. For quantitative links in molecular coordination solids, the TDPS classification introduces clearly defined reference polyhedral shapes, including newly identified geometries, such as the digonal anticupola (DAC-6) for hexacoordination. The characteristic symmetry profiles associated with each TDPS category offer precise geometric parameters that are essential, for example, for accurately refining the Judd–Ofelt Ω_λ_ parameters for lanthanide luminescence. Consequently, spectroscopists may reliably interpret critical spectral features, such as line intensities and crystal-field energy splittings, based on the true intrinsic symmetry of the complex rather than constraining them to fit legacy shape classifications.

By providing experiment-ready geometry labels, this refined framework may help tie measured spectra directly to the actual coordination TDPS, and, for example, in areas where lattice dynamics is relevant, it supports quantitative links to properties such as heat capacity and thermal conductivity in molecular coordination solids. Ultimately, we expect this methodology to help open new avenues for the targeted design of metal-coordinated compounds with tailored and optimized functionalities.

The Supporting Information contains instructions on how to include all of these new CN-6 and CN-7 shapes in the SHAPE software so that crystallographic structures can now be classified more adequately by the CShM formalism into any one of these thermally distinguishable shapes that, as we contend, make a complete set for the characterization of metal complexes in coordination chemistry.

The Compendium within the Supporting Information also includes chirality decision trees and tables of RCR by generic chemical formulae for each of the new thermally distinguishable shapes introduced in this article, thereby completing—specifically for CN-6 and CN-7—the previously published work of our research group.

Ongoing research in our laboratories is focused on determining complete sets of thermally distinguishable shapes for higher coordination numbers.

## Supplementary Material



## Notes

Instructions detailing how to integrate the new CN-6 and CN-7 shapes into the SHAPE software; .ref files corresponding to each of these ideal geometries; a supplementary stereochemistry compendium file with tables with the algebraic countings of stereoisomers, RCRs, subsets, chirality, symmetry numbers and RCWs for all new 6- and 7- coordination number shapes presented in this work, per generic molecular formulae with mono- and/or bidentate ligands; decision trees for the discrimination of metal-centered chirality; Cartesian coordinates of their optimized maximum symmetry minimum repulsion representations as calculated by our algorithm; their cycle index polynomials for counting their stereoisomers using Polya’s enumeration theorem; stereoisomer codes of all identified crystallographic structures of hexacoordinate complexes with the digonal anticupola (DAC-6) shape with only mono- and/or bidentate ligands together with CShM values; a table listing all crystallographic structures of heptacoordinate complexes whose shapes are neither the pentagonal bipyramid (PBPY-7), the capped octahedron (COC-7) or the capped trigonal prism (CTPR-7) together with CShM values; tables, showing CSOM values for the hexacoordinate complexes identified with the TDPS DAC-6, and the heptacoordinate complexes listed in the previous item; and tables showing the euclidean distances d­(*v*_*i*_^1^, *v*_*i*_^2^) of every pair of vertices (*v*_*i*_^1^, *v*_*i*_^2^) between two 6- or two 7-vertex shapes P_1_ and P_2_ after their alignment via RMSD minimization; and CSV files for coordination numbers 6 and 7 containing, for each of the 43,686 coordination complexes examined in this study (each identified by its CSD reference code), the RMSD values calculated for each shape within the complete set of thermally distinguishable polyhedral shapes (TDPSs) relevant to the respective coordination number (either 6 or 7, depending on the complex) (ZIP)
