# Supplementary material for: Thermally Distinguishable Polyhedral Shapes in Chemistry: 6- and 7‑Coordination
Source: ACS Omega. 2025 Sep 29;10(40):47189–209. doi: 10.1021/acsomega.5c05878 (PMC12529146; doi:10.1021/acsomega.5c05878)
Supplement: Supplementary file 1 [file ao5c05878_si_001.zip › iii - Supplementary_Stereochemistry_Compendium.pdf]

Thermally Distinguishable Polyhedral Shapes in Chemistry: 6- and 7-Coordination

Gabriel H. L. Munguba[a], Mateus F. da Silva[b], Frederico T. Silva[a], Gabriel A. Urquiza-Carvalho[a], Alfredo M. Simas[a]

[a]Departamento de Química Fundamental, Universidade Federal de Pernambuco, 50.740-560 Recife, Pernambuco, Brazil

[b]Departamento de Matemática, Universidade Federal de Pernambuco, 50.740-560 Recife, Pernambuco, Brazil

Summary

Abbreviations and Names of Coordination Polyhedra 7
Representations of 6- and 7-Vertex Polyhedral Graphs with Optimized 3D Geometries of Maximum Symmetry and Minimum Repulsion (MSMR) 8
New Geometric Families from MSMR-Optimized 6- and 7-Vertex Polyhedra 12
Stereoisomer Counting, Random Coordination Ratios, Weights, Chirality Indicators, and Symmetry Numbers for the New Coordination Polyhedra 15
Decision Trees for Predicting Coordination Chirality in the New Polyhedral Shapes 45
Ideal Geometries for the Various Shapes and Coordination Numbers. 57
Algebraic Counting of the Number of Stereoisomers for the Selected Coordination Polyhedra 62
Occurrence of the Digonal Anticupola (DAC-6) Coordination Geometry in Reported Crystallographic Structures of Metal Complexes 65
Occurrence of the New 7-Coordinate Geometries Defined in This Study in Known Crystallographic Structures of Metal Complexes 72
Continuous Symmetry Operations Measurements (CSOM) Analyses 77
Rmsd Alignment and Displacement Data for Thermally Indistinguishable Polyhedral Shape Classification Of 6- and 7-Vertex Polyhedra 80
Mathematical Essentials 106
References........ 108

List of Tables

Table S1. Definition of some families of convex polyhedra, including information on number of vertices (V), edges (E) and faces (F), common features as number of n-gons, and maximum symmetry point group (PG). 14

Table S2. Digonal anticupola 16

Table S3. Diminished trigonal trapezohedron. 18

Table S4. Trans-bicapped square pyramid. 21

Table S5. Five-pointed scallop seashell. 24

Table S6. Hemicube. 27

Table S7. Digonal pseudo-anticupola. 30

Table S8. Tetragon-substituted square pyramid. 33

Table S9. Hemiobelisk. 36

Table S10. Tetragonal helicoid with tetragonal base. 39

Table S11. Square hemiantiprism. 42

Table S12. Cartesian coordinates of the maximum symmetry minimum repulsion inscribed forms of the 6-vertex polyhedral shapes studied in this work. The tetragonal antiwedge (TAW-6) has two enantiomorphic representations, distinguished by the prefixes Δ- and Λ-. The hexagon planar geometry (HP-6) is also depicted. 57

Table S13. Cartesian coordinates of the maximum symmetry minimum repulsion inscribed forms of the shapes of coordination number 7 studied in this work. Chiral polyhedra have two enantiomorphic representations, distinguished by the prefixes Δ- and Λ-. The heptagon planar geometry (HP-7) was also depicted. 58

Table S14. List of all crystallographic structures analyzed in this study that feature 6-coordinate metal complexes with mono- and/or bidentate ligands, for which the coordination environment is best described by a digonal anticupola (DAC-6) shape. The table includes the following details for each entry: the CSD reference code (refcode) of the structure, the identity of the metal center, the crystallographic space group, the root-mean-square deviation (RMSD) of the actual coordination environment from the ideal DAC-6 geometry, the CShM values for the DAC-6 shape, and a stereoisomer code describing the compound's stereochemistry. This code encodes the generic formula of the complex, its idealized point group symmetry, the presence or absence of metal-centered chirality, and a permutation vector, among other stereochemical features. 66

Table S15. A table listing all 7-coordinate complexes considered in this work retrieved from crystallographic structures whose coordination polyhedron is not the capped octahedron (COC-7), the capped trigonal prism (CTPR-7) and the pentagonal bipyramid (PBPY-7). The list informs the reference CSD code (refcode) of the crystallographic structure, the metal center of the coordination complex, the spatial group of the crystal structure, the coordination polyhedron of the metal complex, and RMSD and CShM values. 73

Table S16. List of crystallographic structures analyzed in this study that contain hexacoordinate metal complexes with mono- and/or bidentate ligands, whose coordination environments are best described by the digonal anticupola (DAC-6) geometry. For each entry, the table reports the Cambridge Structural Database (CSD) reference code (refcode), the identity of the metal center, the CSOM deviation value (σ_{sym}), and the corresponding CSOM-assigned point group. In a small number of cases, CSOM assigned a D_{3h} point group; these assignments likely reflect borderline cases, as indicated by their relatively larger σ_{sym} values, consistent with geometries positioned near a symmetry boundary. 77

Table S17. List of crystallographic structures analyzed in this study that contain heptacoordinate metal complexes whose coordination environments are best described by the new thermally distinguishable polyhedral shapes (TDPSs) introduced in this work. For each entry, the table reports the Cambridge Structural Database (CSD) reference code (refcode), the identity of the metal center, the CSOM deviation value (σ_{sym}), and the corresponding CSOM-assigned point group. In a few cases, CSOM identified point groups of slightly lower symmetry, which may reflect structural variations encompassed by thermal smearing, yet still broadly consistent with our TDPS classifications 79

Table S18. Minimum values of root mean-square deviation (RMSD) and Euclidean distances of the aligned vertices d($vi1, vi2$) calculated for all possible combination of pair of 6-vertex geometries, P_1 and P_2. These include coordination polyhedra and the hexagonal planar geometry. 81

Table S19. Minimum values of root mean-square deviation (RMSD) and euclidean distances of the aligned vertices d($vi1, vi2$) calculated for all possible combination of pair of 7-vertex geometries, P_1 and P_2. Geometries without known or given symbols are represented by their index numbers. These include coordination polyhedra and the heptagonal planar geometry. 82

List of Figures

Figure S1. A drawing of all seven possible 6-vertex polyhedral graphs and the corresponding inscribed maximum symmetry minimum repulsion representations. Symmetry point groups are indicated, and chiral polyhedra are shown in both enantiomorphic forms. 9

Figure S2. A drawing of all 34 possible 7-vertex polyhedral graphs plus the graph corresponding to the heptagon.. 10

Figure S3. Inscribed maximum symmetry minimum repulsion representations of all 34 convex polyhedra calculated plus the heptagonal planar geometry, with corresponding symmetry point groups indicated. Chiral polyhedra are shown in both enantiomorphic forms. 11

Figure S4. Case examples of convex polyhedra belonging to the family of (n-2)-pointed seashell scallop polyhedra, for n = 6, 7, 8 and 9, respectively. 13

Figure S5. Case examples of polyhedra that belong to different families of polyhedra: cupolae, anticupolae, pseudo-cupolae and pseudo-anticupolae. On the top row, cupolae (n = 6 and 9) are intercalated with pseudo-cupolae (n = 7 and 10). On the bottom row, anticupolae (n = 6 and 9) are pseudo-anticupolae (n = 7 and 10) are shown alternately. 13

Figure S6 Chiral polyhedra of the $(n+1)2$-gonal helicoid family with tetragonal base for n =7, 9 and 11, and the limit figure when n tends to infinity. 14

Figure S7. A bidimensional F vs. V plot of all convex polyhedra, with their upper and lower bound limits indicated... 107

List of Decision Trees by Coordination Number, CN, Thermally Distinguishable Polyhedral Shape and Point Group

CN-6 Digonal anticupola (C_{2v}).. 46

CN-7 Diminished trigonal trapezohedron (C_{3v}).. 47

CN-7 *Trans*-bicapped square pyramid (C_{2v}).. 49

CN-7 Five-pointed scallop seashell (C_{2v}).. 50

CN-7 Hemicube (C_{2v}).. 52

CN-7 Digonal pseudo-anticupola (C_s).. 54

CN-7 Tetragon-substituted pentagonal pyramid (C_s).. 55

CN-7 Hemiobelisk (C_s).. 56

Abbreviations and Names of Coordination Polyhedra

Chemically established shape abbreviations are depicted in **blue**, while those in **deep red-orange** correspond to polyhedra recognized in other fields. In contrast, the dark magenta ones denote shapes that are unique to our study, for which we propose new names and symbolic abbreviations.

Coordination number 6

OC-6	Octahedron
TPR-6	Trigonal prism
PPY-6	Pentagonal pyramid
HP-6	Hexagonal planar
DAC-6	Digonal anticupola
STBPY-6	Skew-trapezoidal bipyramid
CSPY-6	Capped square pyramid
Δ/Λ-TAW-6	Tetragonal antiwedge

Coordination number 7

PBPY-7	Pentagonal bipyramid
COC-7	Capped octahedron
CTPR-7	Capped trigonal prism
HPY-7	Hexagonal pyramid
ETPY-7	Elongated trigonal pyramid
HP-7	Heptagonal planar
DTT-7	Diminished trigonal trapezohedron
TT-7	Tricapped tetrahedron
HECU-7	Hemicube
FPSS-7	Five-pointed scallop seashell
SPBPY-7	Skew pentagonal bipyramid
*Tr*BCSPY-7	*Trans*-bicapped square pyramid
*Cis*BCSPY-7	*Cis*-bicapped square pyramid
CPPY-7	Capped pentagonal pyramid
HEOB-7	Hemiobelisk
DPAC-7	Digonal pseudo-anticupola
TSPPY-7	Tetragon-substituted pentagonal pyramid
Δ/Λ-THTB-7	Tretagonal helicoid with tetragonal base
Δ/Λ-SHEAPR-7	Square hemiantiprism

Representations of 6- and 7-Vertex Polyhedral Graphs with Optimized 3D Geometries of Maximum Symmetry and Minimum Repulsion (MSMR)

By Steinitz's theorem, every 3-connected planar graph corresponds to the 'skeleton' of a three-dimensional convex polyhedron. That is, given such a graph, G, with its corresponding adjacency relations between vertices V connected by edges E, a polyhedral realization P of G in the Euclidean Space is possible. These graphs are commonly referred to as polyhedral graphs.

For each specific number of vertices, there exists a finite number of distinct, non-isomorphic polyhedral graphs. Specifically, for four vertices, exactly one polyhedral graph corresponds to the tetrahedron. For the five vertices, there are exactly two distinct polyhedral graphs: trigonal bipyramid and square pyramid graphs. When considering the six vertices, there are exactly seven distinct polyhedral graphs. For seven vertices, there are exactly thirty-four distinct polyhedral graphs.

Every polyhedral graph possesses an automorphism group, which is defined as the set of all vertex permutations that leaves their adjacency relationships unchanged. In the three-dimensional (3D) realization of such a graph, each automorphism corresponds to a spatial symmetry operation that preserves the structure of the polyhedron. According to Mani's theorem, for every polyhedral graph, there exists at least one realization of its corresponding polyhedron in 3D, where each automorphism is represented as spatial isometry. Such a polyhedron is said to possess the maximum possible symmetry.

In this work, we constructed geometrical representations in 3D of all possible 6- and 7-vertex convex polyhedra from their corresponding graphs, optimized considering a coordination complex situation. Our representations exhibit the following characteristics: they are inscribed in a unit sphere (all vertices lie on the surface of the sphere), possess the maximum possible symmetry, and the total repulsion is minimized. These representations are designated as the Maximum Symmetry Minimum Repulsion (MSMR). In Figure S1, we present two-dimensional (2D) planar drawings of all seven polyhedral graphs with six vertices, with their corresponding MSMR 3D representations optimized by our algorithm. Similarly, the 2D drawings of all thirty-four 7-vertex polyhedral graphs and their MSMR representations are shown in Figures S2 and S3, respectively.

Figure S1. A drawing of all seven possible 6-vertex polyhedral graphs and the corresponding inscribed maximum symmetry minimum repulsion representations. Symmetry point groups are indicated, and chiral polyhedra are shown in both enantiomorphic forms.

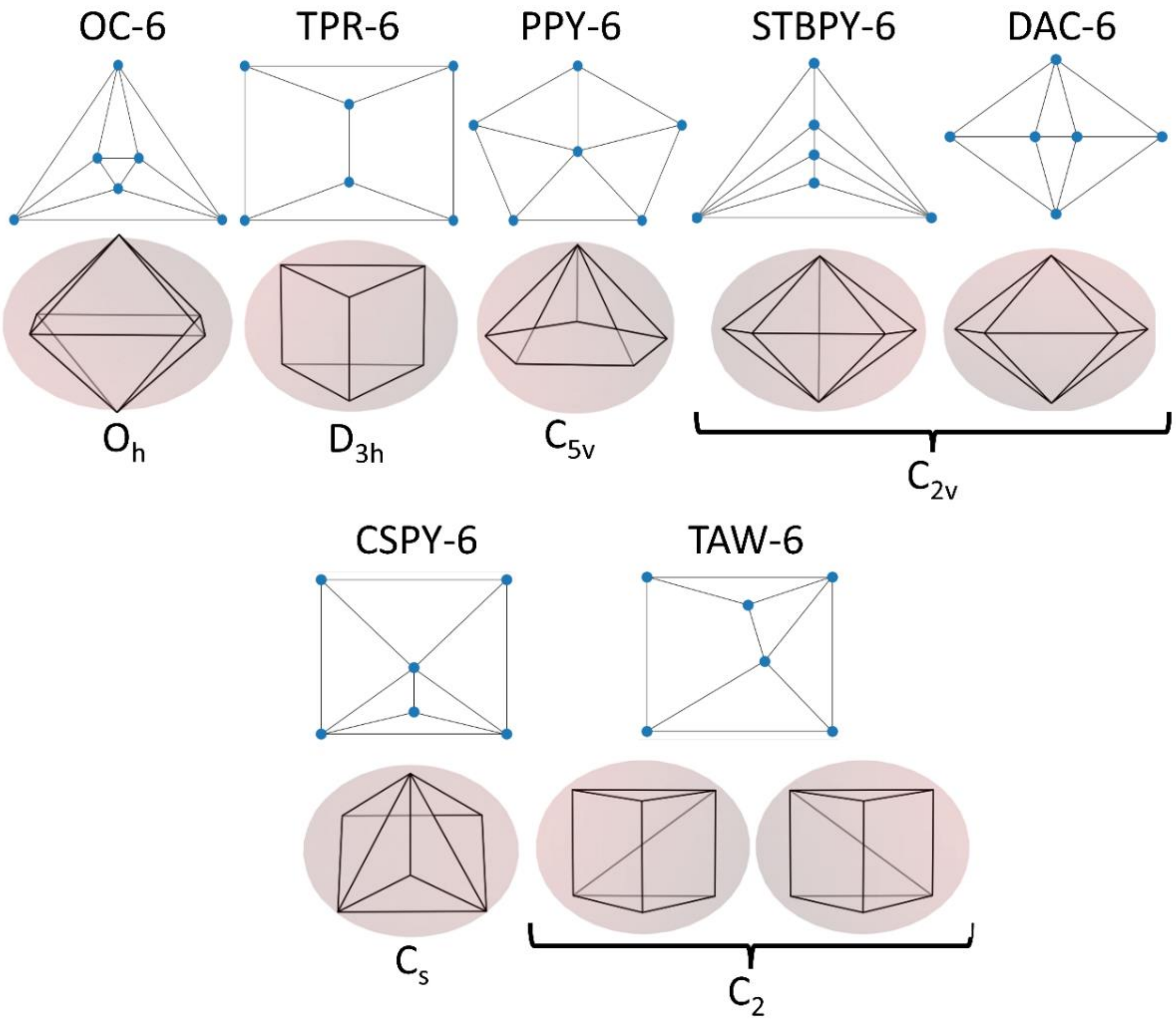

Figure S2. A drawing of all 34 possible 7-vertex polyhedral graphs plus the graph corresponding to the heptagon.

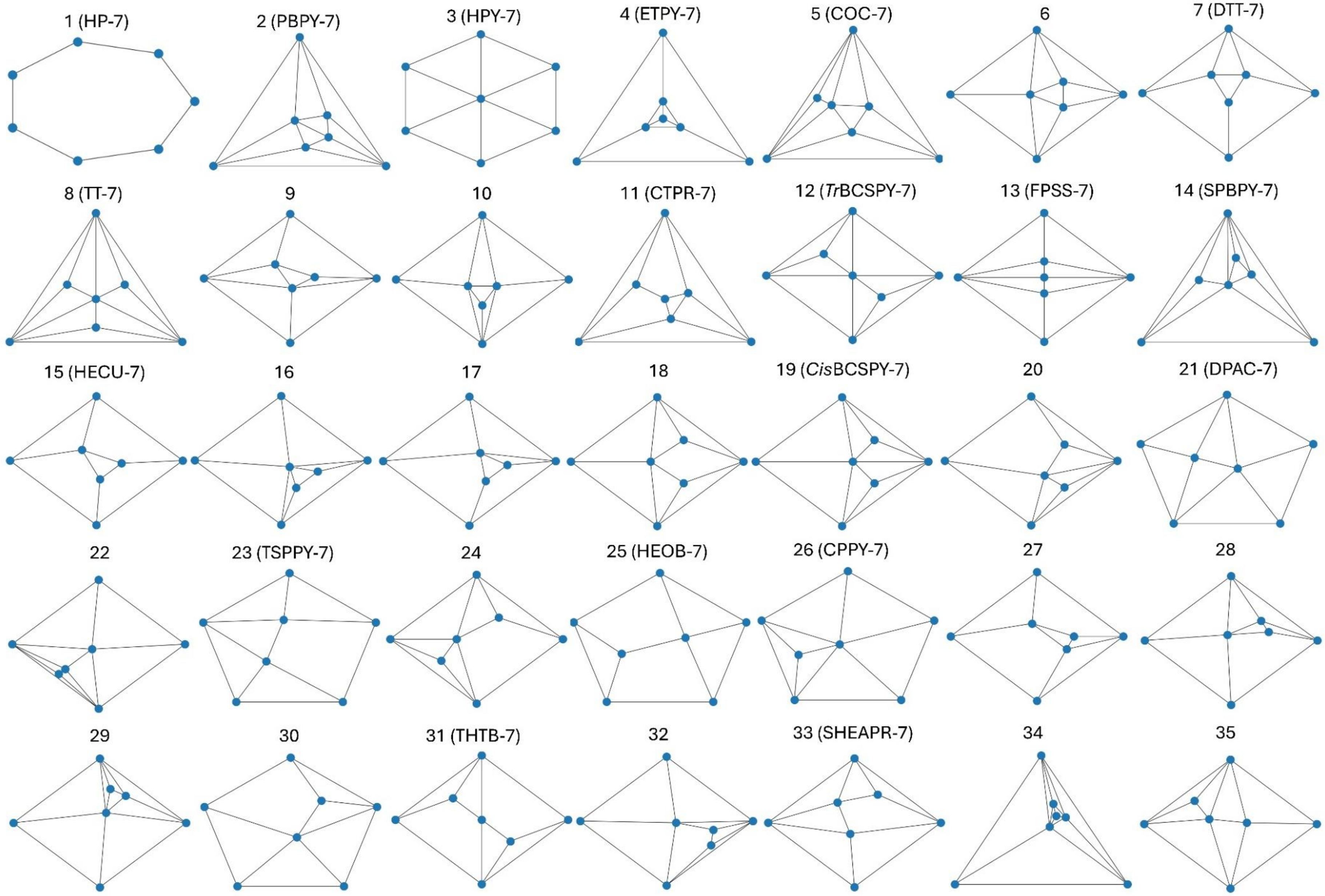

Figure S3. Inscribed maximum symmetry minimum repulsion representations of all 34 convex polyhedra calculated plus the heptagonal planar geometry, with corresponding symmetry point groups indicated. Chiral polyhedra are shown in both enantiomorphic forms.

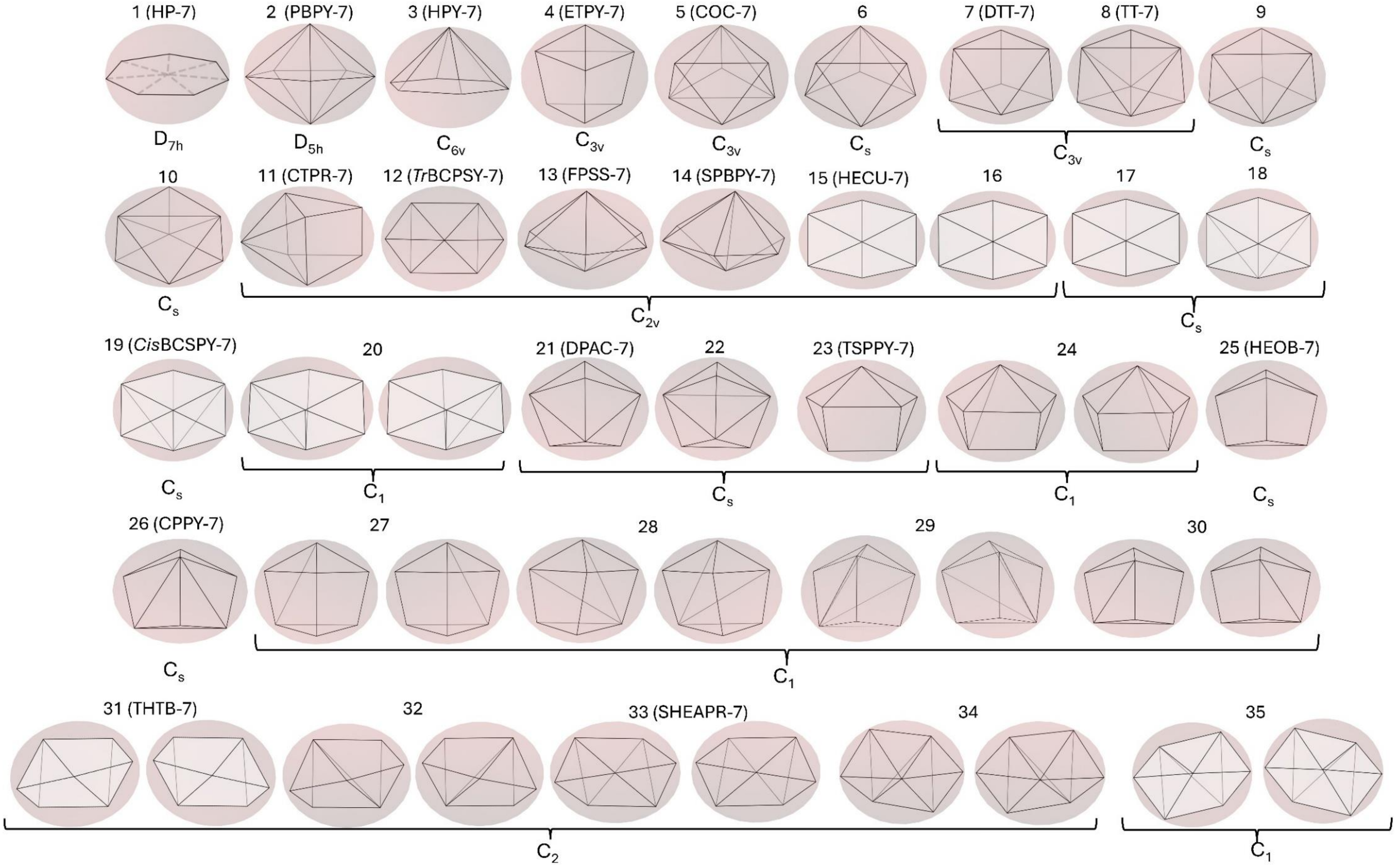

New Geometric Families from MSMR-Optimized 6- and 7-Vertex Polyhedra

Coordination Chemistry recognizes several convex polyhedra as possible molecular geometries for coordination polyhedra of metal complexes. These polyhedra traditionally belong to well-established families of historical significance: Platonic solids, Archimedean solids, Catalan solids, prisms, antiprisms, pyramids, and bipyramids. Additionally, derivatives of these basic forms can be obtained through geometric operations, such as capping, diminishment, truncation, snubification, and torsion. However, as demonstrated in this study, polyhedral combinatorics enables the construction of mathematically complete sets of polyhedra that contain numerous other polyhedra beyond these conventional ones. These polyhedra may not necessarily belong to any of the aforementioned traditional families and thus exhibit unique structural characteristics.

We constructed inscribed representations of all distinct 6- and 7-vertex convex polyhedra from their corresponding graphs, optimized for maximum symmetry and minimal repulsion (MSMR configurations). These include both familiar polyhedra, previously documented in the Coordination Chemistry literature, and unrecognized forms. By considering the thermal smearing effects that are commonly observed in crystal structures, we have demonstrated that some of these novel polyhedra are effectively thermally indistinguishable from known forms and can therefore be adequately represented by well-established geometries. However, other unfamiliar polyhedra exhibit significant geometric distinctions from conventional shapes and are thus thermally distinguishable from these, constituting unique geometries that must be added to the set of reference coordination polyhedra as valid geometries for metal complexes. While some of these polyhedra, although possibly unfamiliar within Coordination Chemistry, have been previously mathematically documented and classified into established polyhedral families, others appear to represent entirely novel geometric structures. Consequently, these new structures necessitate the introduction of suitable names for both individual polyhedra and their corresponding families. We have proposed a systematic nomenclature for these polyhedra based on their structural characteristics and potential grouping into families. Representative examples of newly identified polyhedral families are shown in Figures S4–S6. Table S1 provides an extensive analysis of various polyhedral families, including known forms and those newly introduced here. Specifically, the table details the relationships among the numbers of vertices (V), edges (E), and faces (F) relative to any foundational n-gonal base, along with associated face types and symmetry point groups. For clarity and ease of reference, families previously recognized within Coordination Chemistry are highlighted in light blue, those known from other fields are marked in orange red, and the new families introduced in this study are indicated in violet.

Figure S4. Case examples of convex polyhedra belonging to the family of (n-2)-pointed seashell scallop polyhedra, for n = 6, 7, 8 and 9, respectively.

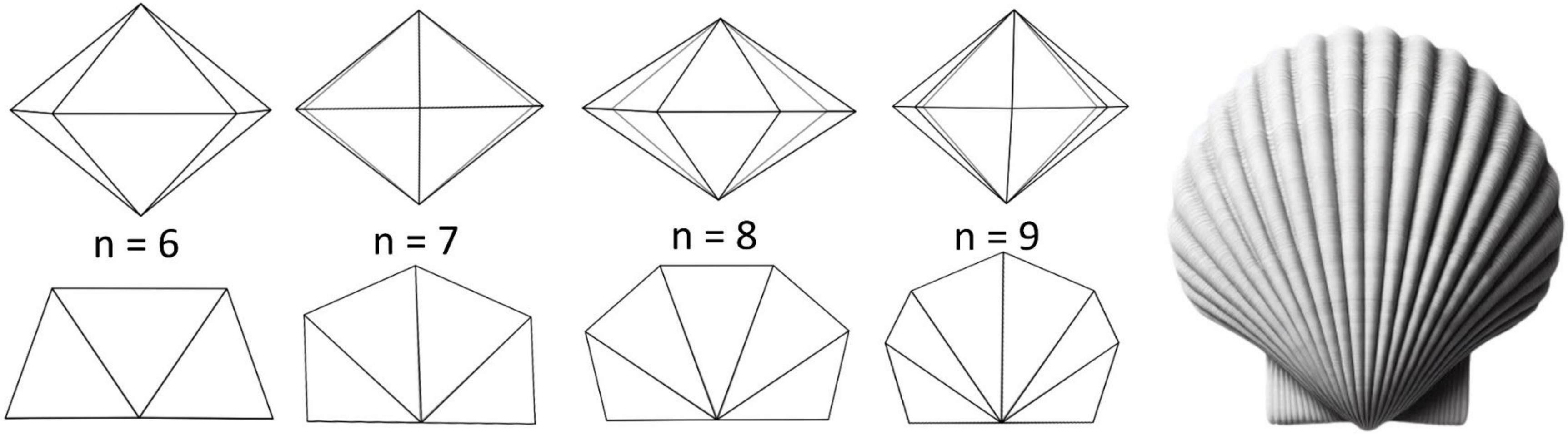

Figure S5. Case examples of polyhedra that belong to different families of polyhedra: cupolae, anticupolae, pseudo-cupolae and pseudo-anticupolae. On the top row, cupolae (n = 6 and 9) are intercalated with pseudo-cupolae (n = 7 and 10). On the bottom row, anticupolae (n = 6 and 9) are pseudo-anticupolae (n = 7 and 10) are shown alternately.

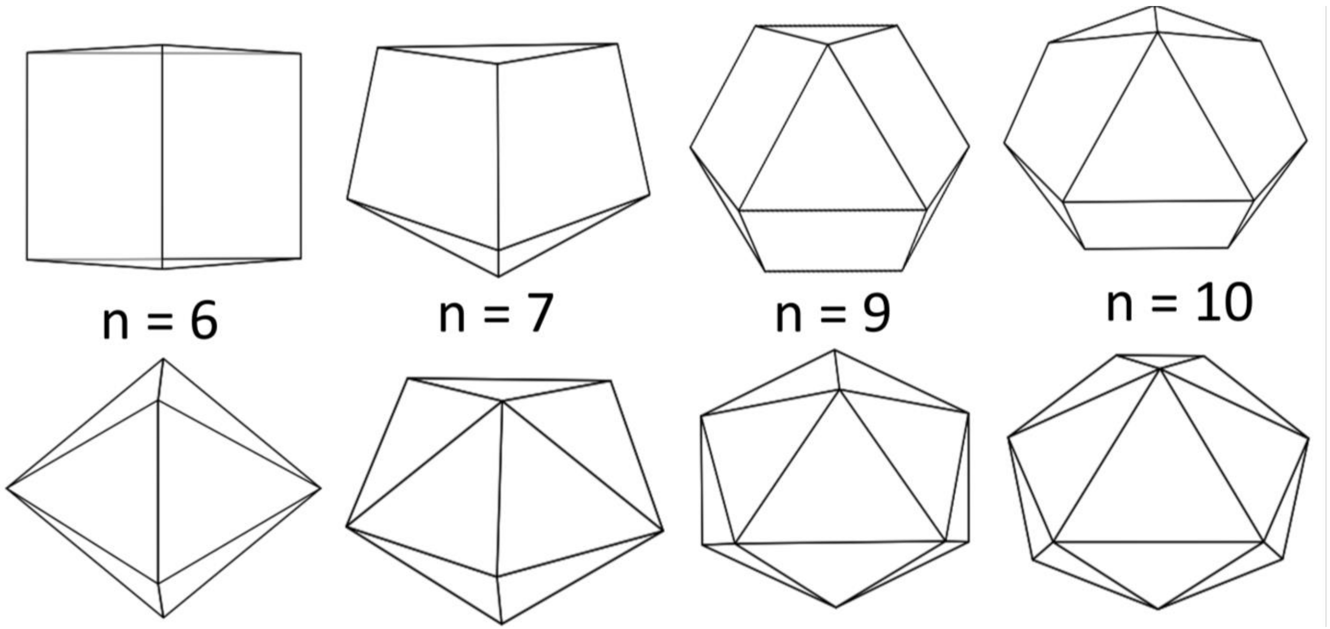

Figure S6 Chiral polyhedra of the $\frac{(n+1)}{2}$-gonal helicoid family with tetragonal base for n =7, 9 and 11, and the limit figure when n tends to infinity.

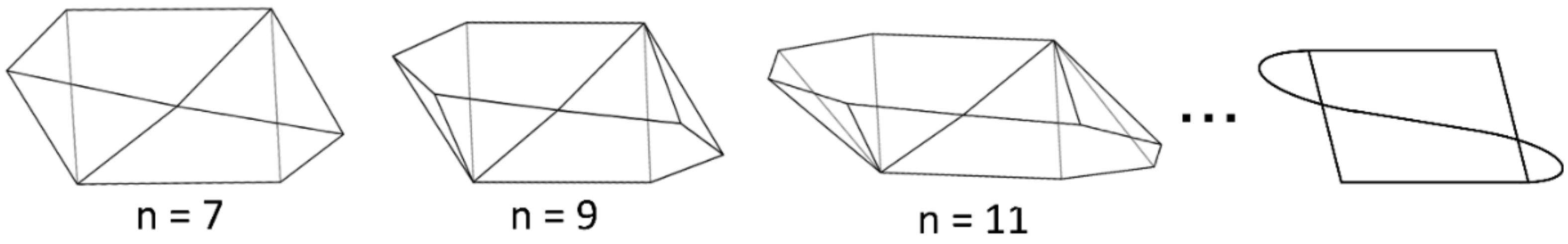

Table S1. Definition of some families of convex polyhedra, including information on number of vertices (V), edges (E) and faces (F), common features as number of n-gons, and maximum symmetry point group (PG).

Name	V	E	F	Features	PG
Pyramid	n+1	2n	n+1	One n-gon, n triangles	C_{nv}
Bipyramid	n+2	3n	2n	2n triangles	D_{nh}
Prism	2n	3n	n+2	Two n-gonos, n quadrilaterals	D_{nh}
Antiprism	2n	4n	2n+2	Two n-gons, 2n triangles	D_{nd}
Trapezohedron	2n+2	4n	2n	2n quadrilaterals	D_{nd}
Cupola	3n	3n	2n+2	One 2n-gon base, one n-gon top, n quadrilaterals alternately with n triangles	C_{nv}
Anticupola	3n	6n	3n+2	One 2n-gon base, one n-gon top, 3n triangles	C_{nv}
Pseudo-cupola	3n+1	3n+2	2n+3	One (2n+1)-gon base, one n-gon top, n quadriaterals intercalated with (n+1) triangles except for 2 triangles that are adjacent	C_s
Pseudo-anticupola	3n+1	6n+2	3n+3	One (2n+1)-gon base, one n-gon top, (3n+1) triangles	C_s
Hemiprism	2n-1	3n-1	n+2	Two n-gons (sharing a vertex), two triangles, (n-2) quadrilaterals	C_{2v}
Wedge	2n-2	3n-3	n+1	Two n-gons (sharing an edges), two triangles, (n-3) quadrilaterals	C_{2v}
Antiwedge (Δ/Λ)	2n-2	4n-6	2n-2	Two n-gons, 2n-4 triangles, four vertices of degree 3	C_n
Helicoid with tetragonal base	n+2	4(n -1)	2n-1	Two n-gons (sharing a vertex), 2(n-2) triangles, a quadrilateral	C_2
Scallop seashell	n+2	3n - 1	2n - 1	One quadrilateral, 2n triangles	C_{2v}

Stereoisomer counting, random coordination ratios, weights, chirality indicators, and symmetry numbers for the new coordination polyhedra

In the following, we present comprehensive tables detailing the stereoisomerism of various coordination polyhedra. These tables provide detailed analyses, including stereoisomer counting, symmetry-based subset classifications according to point groups, and corresponding quantitative parameters such as Random Coordination Ratios (RCRs), Random Coordination Weights (RCWs), chirality indicators, and symmetry numbers introduced in our group's previous publication (DOI: https://doi.org/10.1021/acs.inorgchem.8b01133). Our presentation focuses exclusively on polyhedra that have not been previously analyzed. The current tables maintain consistency with the format and methodology established in that previous study (Silva et al., 2018); therefore, the interpretative guidelines provided on page 3 of its Supporting Information (Hyperlink: ic8b01133_si_001.pdf) remain applicable to the present data tables.

Table S2. Digonal anticupola. Total is the number of stereoisomers for the corresponding shape and formula, of which c is the number of chiral and a is the number of achiral stereoisomers. RCR is the random coordination ratio. All stereoisomers are classified into subsets according to their point groups and ordered in terms of the product of the number # of distinct stereoisomers by their corresponding random coordination weights, RCWs. χ identifies chirality of the coordination polyhedra of all stereoisomers of the subset, with a meaning that they are achiral, and c meaning that they are chiral; and σ is the rotational symmetry number.

DAC-6 Digonal anticupola

Formula	Set			RCR	Subsets											
	Total	c	a		Subset	Group	χ	σ	RCW	#	Subset	Group	χ	σ	RCW	#
Ma6	1	0	1	1	A	C2v	a	2	360	1						
Ma5b	3	0	3	1	A	Cs	a	1	120	3						
Ma4b2	9	4	5	2.7:1.3:1	A	C1	c	1	48	4	B	Cs	a	1	48	2
					C	C2v	a	2	24	3						
Ma4bc	15	8	7	1.1:1	A	C1	c	1	24	8	B	Cs	a	1	24	7
Ma4(AA)	6	4	2	8:2:1	A	C1	c	1	48	4	B	Cs	a	1	48	1
					C	C2v	a	2	24	1						
Ma4(AB)	11	8	3	2.7:1	A	C1	c	1	24	8	B	Cs	a	1	24	3
Ma3b3	10	4	6	1.5:1	A	Cs	a	1	36	6	B	C1	c	1	36	4
Ma3b2c	30	20	10	2:1	A	C1	c	1	12	20	B	Cs	a	1	12	10
Ma3bcd	60	48	12	4:1	A	C1	c	1	6	48	B	Cs	a	1	6	12
Ma3b(AA)	22	18	4	4.5:1	A	C1	c	1	12	18	B	Cs	a	1	12	4
Ma3b(AB)	44	38	6	6.3:1	A	C1	c	1	6	38	B	Cs	a	1	6	6
Ma2b2c2	48	36	12	12:2:1	A	C1	c	1	8	36	B	Cs	a	1	8	6
					C	C2v	a	2	4	6						
Ma2b2cd	90	76	14	5.4:1	A	C1	c	1	4	76	B	Cs	a	1	4	14
Ma2b2(AA)	34	30	4	30:2:1	A	C1	c	1	8	30	B	Cs	a	1	8	2
					C	C2v	a	2	4	2						
Ma2b2(AB)	66	60	6	10:1	A	C1	c	1	4	60	B	Cs	a	1	4	6
Ma2bcde	180	168	12	14:1	A	C1	c	1	2	168	B	Cs	a	1	2	12
Ma2bc(AA)	66	62	4	15.5:1	A	C1	c	1	4	62	B	Cs	a	1	4	4
Ma2bc(AB)	132	126	6	21:1	A	C1	c	1	2	126	B	Cs	a	1	2	6
Ma2(AA)2	15	14	1	20:4:1	A	C1	c	1	16	10	B	C2	c	2	8	4
					C	C2v	a	2	8	1						

Table S2 (continuation).

DAC-6 Digonal anticupola

Formula*	Set			**RCR**	Subsets											
	Total	c	a		Subset	Group	χ	σ	RCW	#	Subset	Group	χ	σ	RCW	#
Ma2(AA)(BB)	25	24	1	24:1	A	C1	c	1	8	24	B	Cs	a	1	8	1
Ma2(AA)(AB)	50	48	2	24:1	A	C1	c	1	4	48	B	Cs	a	1	4	2
Ma2(AB)2	55	52	3	44:4:1:1	A	C1	c	1	4	44	B	C2	c	2	2	8
					C'	Cs	a	1	4	1	C''	C2v	a	2	2	2
Ma2(AB)(CD)	100	96	4	24:1	A	C1	c	1	2	96	B	Cs	a	1	2	4
Mabcdef	360	360	0	1	A	C1	c	1	1	360						
Mabcd(AA)	132	132	0	1	A	C1	c	1	2	132						
Mabcd(AB)	264	264	0	1	A	C1	c	1	1	264						
Mab(AA)2	25	24	1	24:1	A	C1	c	1	8	24	B	Cs	a	1	8	1
Mab(AA)(BB)	50	50	0	1	A	C1	c	1	4	50						
Mab(AA)(AB)	100	100	0	1	A	C1	c	1	2	100						
Mab(AB)2	100	98	2	49:1	A	C1	c	1	2	98	B	Cs	a	1	2	2
Mab(AB)(CD)	200	200	0	1	A	C1	c	1	1	200						
M(AA)3	4	4	0	2:1	A	C1	c	1	48	2	B	C2	c	2	24	2
M(AA)2(BB)	10	10	0	8:1	A	C1	c	1	16	8	B	C2	c	2	8	2
M(AA)2(AB)	18	18	0	1	A	C1	c	1	8	18						
M(AA)(BB)(CC)	18	18	0	1	A	C1	c	1	8	18						
M(AA)(BB)(AB)	36	36	0	1	A	C1	c	1	4	36						
M(AA)(AB)2	38	38	0	17:1	A	C1	c	1	4	34	B	C2	c	2	2	4
M(AA)(AB)(CD)	72	72	0	1	A	C1	c	1	2	72						
M(AB)3	24	24	0	1	A	C1	c	1	6	24						
M(AB)2(CD)	72	72	0	1	A	C1	c	1	2	72						
M(AB)(CD)(EF)	144	144	0	1	A	C1	c	1	1	144						

*a, b, c, d, refer to monodentate ligands; (AA) and (AB) refer to bidentate ligands, (AA) being symmetrical, and (AB) non-symmetrical, with A and B representing non-equivalent coordinating atoms.

Table S3. Diminished trigonal trapezohedron. Total is the number of stereoisomers for the corresponding shape and formula, of which c is the number of chiral and a is the number of achiral stereoisomers. RCR is the random coordination ratio. All stereoisomers are classified into subsets according to their point groups and ordered in terms of the product of the number # of distinct stereoisomers by their corresponding random coordination weights, RCWs. χ identifies chirality of the coordination polyhedra of all stereoisomers of the subset, with a meaning that they are achiral, and c meaning that they are chiral; and σ is the rotational symmetry number.

DTT-7 Diminished trigonal trapezohedron

Formula	Set			**RCR**	Subsets											
	Total	c	a		Subset	Group	χ	σ	RCW	#	Subset	Group	χ	σ	RCW	#
Ma7	1	0	1	1	A	C3v	a	3	1680	1						
Ma6b	3	0	3	6:1	A	Cs	a	1	720	2	B	C3v	a	3	240	1
Ma5b2	7	2	5	2.5:1	A	Cs	a	1	240	5	B	C1	c	1	240	2
Ma5bc	14	8	6	1.3:1	A	C1	c	1	120	8	B	Cs	a	1	120	6
Ma5(AA)	4	2	2	1:1	A'	C1	c	1	240	2	A''	Cs	a	1	240	2
Ma5(AB)	8	6	2	3:1	A	C1	c	1	120	6	B	Cs	a	1	120	2
Ma4b3	13	6	7	9:7.5:1	A	C1	c	1	144	6	B	Cs	a	1	144	5
					C	C3v	a	3	48	2						
Ma4b2c	35	26	9	2.9:1	A	C1	c	1	48	26	B	Cs	a	1	48	9
Ma4bcd	70	64	6	10.7:1	A	C1	c	1	24	64	B	Cs	a	1	24	6
Ma4b(AA)	20	16	4	4:1	A	C1	c	1	48	16	B	Cs	a	1	48	4
Ma4b(AB)	40	38	2	19:1	A	C1	c	1	24	38	B	Cs	a	1	24	2
Ma3b3c	48	36	12	54:15:1	A	C1	c	1	36	36	B	Cs	a	1	36	10
					C	C3v	a	3	12	2						
Ma3b2c2	70	56	14	4:1	A	C1	c	1	24	56	B	Cs	a	1	24	14
Ma3b2cd	140	128	12	10.7:1	A	C1	c	1	12	128	B	Cs	a	1	12	12
Ma3b2(AA)	40	34	6	5.7:1	A	C1	c	1	24	34	B	Cs	a	1	24	6
Ma3b2(AB)	80	76	4	19:1	A	C1	c	1	12	76	B	Cs	a	1	12	4
Ma3bcde	280	280	0	1	A	C1	c	1	6	280						
Ma3bc(AA)	80	74	6	12.3:1	A	C1	c	1	12	74	B	Cs	a	1	12	6
Ma3bc(AB)	160	160	0	1	A	C1	c	1	6	160						
Ma3(AA)2	12	10	2	5:1	A	C1	c	1	48	10	B	Cs	a	1	48	2

Table S3 (continuation).

DTT-7 Diminished trigonal trapezohedron

Formula*	Set			RCR	Subsets											
	Total	c	a		Subset	Group	χ	σ	RCW	#	Subset	Group	χ	σ	RCW	#
Ma3(AA)(BB)	24	22	2	11:1	A	C1	c	1	24	22	B	Cs	a	1	24	2
Ma3(AA)(AB)	48	46	2	23:1	A	C1	c	1	12	46	B	Cs	a	1	12	2
Ma3(AB)2	48	46	2	23:1	A	C1	c	1	12	46	B	Cs	a	1	12	2
Ma3(AB)(CD)	96	96	0	1	A	C1	c	1	6	96						
Ma2b2c2d	210	192	18	10.7:1	A	C1	c	1	8	192	B	Cs	a	1	8	18
Ma2b2cde	420	408	12	34:1	A	C1	c	1	4	408	B	Cs	a	1	4	12
Ma2b2c(AA)	120	112	8	14:1	A	C1	c	1	8	112	B	Cs	a	1	8	8
Ma2b2c(AB)	240	236	4	59:1	A	C1	c	1	4	236	B	Cs	a	1	4	4
Ma2bcdef	840	840	0	1	A	C1	c	1	2	840						
Ma2bcd(AA)	240	234	6	39:1	A	C1	c	1	4	234	B	Cs	a	1	4	6
Ma2bcd(AB)	480	480	0	1	A	C1	c	1	2	480						
Ma2b(AA)2	36	32	4	8:1	A	C1	c	1	16	32	B	Cs	a	1	16	4
Ma2b(AA)(BB)	72	70	2	35:1	A	C1	c	1	8	70	B	Cs	a	1	8	2
Ma2b(AA)(AB)	144	142	2	71:1	A	C1	c	1	4	142	B	Cs	a	1	4	2
Ma2b(AB)2	144	138	6	23:1	A	C1	c	1	4	138	B	Cs	a	1	4	6
Ma2b(AB)(CD)	288	288	0	1	A	C1	c	1	2	288						
Mabcdefg	1680	1680	0	1	A	C1	c	1	1	1680						
Mabcde(AA)	480	480	0	1	A	C1	c	1	2	480						
Mabcde(AB)	960	960	0	1	A	C1	c	1	1	960						
Mabc(AA)2	72	66	6	11:1	A	C1	c	1	8	66	B	Cs	a	1	8	6
Mabc(AA)(BB)	144	144	0	1	A	C1	c	1	4	144						
Mabc(AA)(AB)	288	288	0	1	A	C1	c	1	2	288						
Mabc(AB)2	288	276	12	23:1	A	C1	c	1	2	276	B	Cs	a	1	2	12
Mabc(AB)(CD)	576	576	0	1	A	C1	c	1	1	576						
Ma(AA)3	9	8	1	9:1.5:1	A	C1	c	1	48	6	B	Cs	a	1	48	1
					C	C3	c	3	16	2						
Ma(AA)2(BB)	23	22	1	22:1	A	C1	c	1	16	22	B	Cs	a	1	16	1

Table S3 (continuation).

DTT-7 Diminished trigonal trapezohedron

Formula*	Set			RCR	Subsets											
	Total	c	a		Subset	Group	χ	σ	RCW	#	Subset	Group	χ	σ	RCW	#
Ma(AA)2(AB)	46	44	2	22:1	A	C1	c	1	8	44	B	Cs	a	1	8	2
Ma(AA)(BB)(CC)	46	46	0	1	A	C1	c	1	8	46						
Ma(AA)(BB)(AB)	92	92	0	1	A	C1	c	1	4	92						
Ma(AA)(AB)2	92	90	2	45:1	A	C1	c	1	4	90	B	Cs	a	1	4	2
Ma(AA)(AB)(CD)	184	184	0	1	A	C1	c	1	2	184						
Ma(AB)3	64	60	4	42:3:1	A	C1	c	1	6	56	B	Cs	a	1	6	4
					C	C3	c	3	2	4						
Ma(AB)2(CD)	184	180	4	45:1	A	C1	c	1	2	180	B	Cs	a	1	2	4
Ma(AB)(CD)(EF)	368	368	0	1	A	C1	c	1	1	368						

*a, b, c, d, refer to monodentate ligands; (AA) and (AB) refer to bidentate ligands, (AA) being symmetrical, and (AB) non-symmetrical, with A and B representing non-equivalent coordinating atoms.

Table S4. *Trans*-bicapped square pyramid. Total is the number of stereoisomers for the corresponding shape and formula, of which c is the number of chiral and a is the number of achiral stereoisomers. RCR is the random coordination ratio. All stereoisomers are classified into subsets according to their point groups and ordered in terms of the product of the number # of distinct stereoisomers by their corresponding random coordination weights, RCWs. χ identifies chirality of the coordination polyhedra of all stereoisomers of the subset, with a meaning that they are achiral, and c meaning that they are chiral; and σ is the rotational symmetry number.

_Tr_BCSPY-7 _Trans_-bicapped square pyramid

Formula	Set			RCR	Subsets											
	Total	c	a		Subset	Group	χ	σ	RCW	#	Subset	Group	χ	σ	RCW	#
Ma7	1	0	1	1	A	C2v	a	2	2520	1						
Ma6b	4	2	2	4:2:1	A	C1	c	1	720	2	B	Cs	a	1	720	1
					C	C2v	a	2	360	1						
Ma5b2	12	8	4	12:6:2:1	A	C1	c	1	240	6	B	Cs	a	1	240	3
					C	C2	c	2	120	2	D	C2v	a	2	120	1
Ma5bc	21	18	3	6:1	A	C1	c	1	120	18	B	Cs	a	1	120	3
Ma5(AA)	7	4	3	4:3	A	C1	c	1	240	4	B	Cs	a	1	240	3
Ma5(AB)	14	12	2	6:1	A	C1	c	1	120	12	B	Cs	a	1	120	2
Ma4b3	19	14	5	24:8:2:1	A	C1	c	1	144	12	B	Cs	a	1	144	4
					C	C2	c	2	72	2	D	C2v	a	2	72	1
Ma4b2c	54	48	6	92:10:2:1	A	C1	c	1	48	46	B	Cs	a	1	48	5
					C	C2	c	2	24	2	D	C2v	a	2	24	1
Ma4bcd	105	102	3	34:1	A	C1	c	1	24	102	B	Cs	a	1	24	3
Ma4b(AA)	35	30	5	6:1	A	C1	c	1	48	30	B	Cs	a	1	48	5
Ma4b(AB)	70	68	2	34:1	A	C1	c	1	24	68	B	Cs	a	1	24	2
Ma3b3c	70	64	6	10.7:1	A	C1	c	1	36	64	B	Cs	a	1	36	6
Ma3b2c2	108	98	10	30.7:3.3:1	A	C1	c	1	24	92	B	Cs	a	1	24	10
					C	C2	c	2	12	6						
Ma3b2cd	210	204	6	34:1	A	C1	c	1	12	204	B	Cs	a	1	12	6
Ma3b2(AA)	70	62	8	7.75:1	A	C1	c	1	24	62	B	Cs	a	1	24	8
Ma3b2(AB)	140	136	4	34:1	A	C1	c	1	12	136	B	Cs	a	1	12	4
Ma3bcde	420	420	0	1	A	C1	c	1	6	420						

Table S4 (continuation).

_Tr_BCSPY-7 _Trans_-bicapped square pyramid

Formula*	Set			RCR	Subsets											
	Total	c	a		Subset	Group	χ	σ	RCW	#	Subset	Group	χ	σ	RCW	#
Ma3bc(AA)	140	134	6	22.3:1	A	C1	c	1	12	134	B	Cs	a	1	12	6
Ma3bc(AB)	280	280	0	1	A	C1	c	1	6	280						
Ma3(AA)2	25	20	5	18:3:1:1	A	C1	c	1	48	18	B	Cs	a	1	48	3
					C'	C2	c	2	24	2	C''	C2v	a	2	24	2
Ma3(AA)(BB)	46	40	6	6.7:1	A	C1	c	1	24	40	B	Cs	a	1	24	6
Ma3(AA)(AB)	92	88	4	22:1	A	C1	c	1	12	88	B	Cs	a	1	12	4
Ma3(AB)2	96	92	4	21:1:1	A	C1	c	1	12	84	B'	Cs	a	1	12	4
					B''	C2	c	2	6	8						
Ma3(AB)(CD)	184	184	0	1	A	C1	c	1	6	184						
Ma2b2c2d	318	306	12	100:4:1	A	C1	c	1	8	300	B	Cs	a	1	8	12
					C	C2	c	2	4	6						
Ma2b2cde	630	624	6	104:1	A	C1	c	1	4	624	B	Cs	a	1	4	6
Ma2b2c(AA)	210	200	10	20:1	A	C1	c	1	8	200	B	Cs	a	1	8	10
Ma2b2c(AB)	420	416	4	104:1	A	C1	c	1	4	416	B	Cs	a	1	4	4
Ma2bcdef	1260	1260	0	1	A	C1	c	1	2	1260						
Ma2bcd(AA)	420	414	6	69:1	A	C1	c	1	4	414	B	Cs	a	1	4	6
Ma2bcd(AB)	840	840	0	1	A	C1	c	1	2	840						
Ma2b(AA)2	71	64	7	62:5:1:1	A	C1	c	1	16	62	B	Cs	a	1	16	5
					C'	C2	c	2	8	2	C''	C2v	a	2	8	2
Ma2b(AA)(BB)	138	130	8	16.25:1	A	C1	c	1	8	130	B	Cs	a	1	8	8
Ma2b(AA)(AB)	276	272	4	68:1	A	C1	c	1	4	272	B	Cs	a	1	4	4
Ma2b(AB)2	280	274	6	66.5:1.5:1	A	C1	c	1	4	266	B	Cs	a	1	4	6
					C	C2	c	2	2	8						
Ma2b(AB)(CD)	552	552	0	1	A	C1	c	1	2	552						
Mabcdefg	2520	2520	0	1	A	C1	c	1	1	2520						
Mabcde(AA)	840	840	0	1	A	C1	c	1	2	840						
Mabcde(AB)	1680	1680	0	1	A	C1	c	1	1	1680						

Table S4 (continuation).

TrBCSPY-7 *Trans*-bicapped square pyramid

Formula*	Set			RCR	Subsets											
	Total	c	a		Subset	Group	χ	σ	RCW	#	Subset	Group	χ	σ	RCW	#
Mabc(AA)2	138	132	6	22:1	A	C1	c	1	8	132	B	Cs	a	1	8	6
Mabc(AA)(BB)	276	270	6	45:1	A	C1	c	1	4	270	B	Cs	a	1	4	6
Mabc(AA)(AB)	552	552	0	1	A	C1	c	1	2	552						
Mabc(AB)2	552	546	6	91:1	A	C1	c	1	2	546	B	Cs	a	1	2	6
Mabc(AB)(CD)	1104	1104	0	1	A	C1	c	1	1	1104						
Ma(AA)3	15	12	3	4:1	A	C1	c	1	48	12	B	Cs	a	1	48	3
Ma(AA)2(BB)	45	40	5	8:1	A	C1	c	1	16	40	B	Cs	a	1	16	5
Ma(AA)2(AB)	90	86	4	21.5:1	A	C1	c	1	8	86	B	Cs	a	1	8	4
Ma(AA)(BB)(CC)	90	84	6	14:1	A	C1	c	1	8	84	B	Cs	a	1	8	6
Ma(AA)(BB)(AB)	180	176	4	44:1	A	C1	c	1	4	176	B	Cs	a	1	4	4
Ma(AA)(AB)2	180	176	4	44:1	A	C1	c	1	4	176	B	Cs	a	1	4	4
Ma(AA)(AB)(CD)	360	360	0	1	A	C1	c	1	2	360						
Ma(AB)3	120	116	4	29:1	A	C1	c	1	6	116	B	Cs	a	1	6	4
Ma(AB)2(CD)	360	356	4	89:1	A	C1	c	1	2	356	B	Cs	a	1	2	4
Ma(AB)(CD)(EF)	720	720	0	1	A	C1	c	1	1	720						

*a, b, c, d, refer to monodentate ligands; (AA) and (AB) refer to bidentate ligands, (AA) being symmetrical, and (AB) non-symmetrical, with A and B representing non-equivalent coordinating atoms.

Table S5. Five-pointed scallop seashell. Total is the number of stereoisomers for the corresponding shape and formula, of which c is the number of chiral and a is the number of achiral stereoisomers. RCR is the random coordination ratio. All stereoisomers are classified into subsets according to their point groups and ordered in terms of the product of the number # of distinct stereoisomers by their corresponding random coordination weights, RCWs. χ identifies chirality of the coordination polyhedra of all stereoisomers of the subset, with a meaning that they are achiral, and c meaning that they are chiral; and σ is the rotational symmetry number.

FPSS-7 Five-pointed scallop seashell

Formula	Set			**RCR**	Subsets											
	Total	c	a		Subset	Group	χ	σ	RCW	#	Subset	Group	χ	σ	RCW	#
Ma7	1	0	1	1	A	C2v	a	2	2520	1						
Ma6b	4	0	4	6:1	A	Cs	a	1	720	3	B	C2v	a	2	360	1
Ma5b2	12	4	8	3.3:2.7:1	A	Cs	a	1	240	5	B	C1	c	1	240	4
					C	C2v	a	2	120	3						
Ma5bc	21	8	13	1.625:1	A	Cs	a	1	120	13	B	C1	c	1	120	8
Ma5(AA)	7	4	3	1.3:1	A	C1	c	1	240	4	B	Cs	a	1	240	3
Ma5(AB)	14	8	6	1.3:1	A	C1	c	1	120	8	B	Cs	a	1	120	6
Ma4b3	19	8	11	5.3:5.3:1	A'	Cs	a	1	144	8	A''	C1	c	1	144	8
					B	C2v	a	2	72	3						
Ma4b2c	54	32	22	21.3:12.7:1	A	C1	c	1	48	32	B	Cs	a	1	48	19
					C	C2v	a	2	24	3						
Ma4bcd	105	72	33	2.18:1	A	C1	c	1	24	72	B	Cs	a	1	24	33
Ma4b(AA)	35	28	7	4:1	A	C1	c	1	48	28	B	Cs	a	1	48	7
Ma4b(AB)	70	56	14	4:1	A	C1	c	1	24	56	B	Cs	a	1	24	14
Ma3b3c	70	44	26	1.7:1	A	C1	c	1	36	44	B	Cs	a	1	36	26
Ma3b2c2	108	76	32	25.3:8.7:1	A	C1	c	1	24	76	B	Cs	a	1	24	26
					C	C2v	a	1	12	6						
Ma3b2cd	210	164	46	3.57:1	A	C1	c	1	12	164	B	Cs	a	1	12	46
Ma3b2(AA)	70	60	10	6:1	A	C1	c	1	24	60	B	Cs	a	1	24	10
Ma3b2(AB)	140	120	20	6:1	A	C1	c	1	12	120	B	Cs	a	1	12	20
Ma3bcde	420	360	60	6:1	A	C1	c	1	6	360	B	Cs	a	1	6	60
Ma3bc(AA)	140	128	12	10.7:1	A	C1	c	1	12	128	B	Cs	a	1	12	12

Table S5 (continuation).

FPSS-7 Five-pointed scallop seashell

Formula*	Set			RCR	Subsets											
	Total	c	a		Subset	Group	χ	σ	RCW	#	Subset	Group	χ	σ	RCW	#
Ma3bc(AB)	280	256	24	10.7:1	A	C1	c	1	6	256	B	Cs	a	1	6	24
Ma3(AA)2	26	24	2	40:4:2:1	A	C1	c	1	48	20	B	C2	c	2	24	4
					C	Cs	a	1	48	1	D	C2v	a	2	24	1
Ma3(AA)(BB)	47	44	3	14.7:1	A	C1	c	1	24	44	B	Cs	a	1	24	3
Ma3(AA)(AB)	94	88	6	14.7:1	A	C1	c	1	12	88	B	Cs	a	1	12	6
Ma3(AB)2	99	92	7	84:5:4:1	A	C1	c	1	12	84	B	Cs	a	1	12	5
					C	C2	c	2	6	8	D	C2v	a	2	6	2
Ma3(AB)(CD)	188	176	12	14.7:1	A	C1	c	1	6	176	B	Cs	a	1	6	12
Ma2b2c2d	318	264	54	88:16:1	A	C1	c	1	8	264	B	Cs	a	1	8	48
					C	C2v	a	2	4	6						
Ma2b2cde	630	564	66	8.55:1	A	C1	c	1	4	564	B	Cs	a	1	4	66
Ma2b2c(AA)	210	196	14	14:1	A	C1	c	1	8	196	B	Cs	a	1	8	14
Ma2b2c(AB)	420	392	28	14:1	A	C1	c	1	4	392	B	Cs	a	1	4	28
Ma2bcdef	1260	1200	60	20:1	A	C1	c	1	2	1200	B	Cs	a	1	2	60
Ma2bcd(AA)	420	408	12	34:1	A	C1	c	1	4	408	B	Cs	a	1	4	12
Ma2bcd(AB)	840	816	24	34:1	A	C1	c	1	2	816	B	Cs	a	1	2	24
Ma2b(AA)2	73	70	3	132:4:4:1	A	C1	c	1	16	66	B'	Cs	a	1	16	2
					B''	C2	c	2	8	4	C	C2v	a	2	8	1
Ma2b(AA)(BB)	141	138	3	46:1	A	C1	c	1	8	138	B	Cs	a	1	8	3
Ma2b(AA)(AB)	282	276	6	46:1	A	C1	c	1	4	276	B	Cs	a	1	4	6
Ma2b(AB)2	287	278	9	270:7:4:1	A	C1	c	1	4	270	B	Cs	a	1	4	7
					C	C2	c	2	2	8	D	C2v	a	2	2	2
Ma2b(AB)(CD)	564	552	12	46:1	A	C1	c	1	2	552	B	Cs	a	1	2	12
Mabcdefg	2520	2520	0	1	A	C1	c	1	1	2520						
Mabcde(AA)	840	840	0	1	A	C1	c	1	2	840						
Mabcde(AB)	1680	1680	0	1	A	C1	c	1	1	1680						
Mabc(AA)2	141	138	3	46:1	A	C1	c	1	8	138	B	Cs	a	1	8	3

Table S5 (continuation).

FPSS-7 Five-pointed scallop seashell

Formula*	Set			**RCR**	Subsets											
	Total	c	a		Subset	Group	χ	σ	RCW	#	Subset	Group	χ	σ	RCW	#
Mabc(AA)(BB)	282	282	0	1	A	C1	c	1	4	282						
Mabc(AA)(AB)	564	564	0	1	A	C1	c	1	2	564						
Mabc(AB)2	564	558	6	93:1	A	C1	c	1	2	558	B	Cs	a	1	2	6
Mabc(AB)(CD)	1128	1128	0	1	A	C1	c	1	1	1128						
Ma(AA)3	15	14	1	14:1	A	C1	c	1	48	14	B	Cs	a	1	48	1
Ma(AA)2(BB)	45	44	1	44:1	A	C1	c	1	16	44	B	Cs	a	1	16	1
Ma(AA)2(AB)	90	88	2	44:1	A	C1	c	1	8	88	B	Cs	a	1	8	2
Ma(AA)(BB)(CC)	90	90	0	1	A	C1	c	1	8	90						
Ma(AA)(BB)(AB)	180	180	0	1	A	C1	c	1	4	180						
Ma(AA)(AB)2	180	178	2	89:1	A	C1	c	1	4	178	B	Cs	a	1	4	2
Ma(AA)(AB)(CD)	360	360	0	1	A	C1	c	1	2	360						
Ma(AB)3	120	116	4	29:1	A	C1	c	1	6	116	B	Cs	a	1	6	4
Ma(AB)2(CD)	360	356	4	89:1	A	C1	c	1	2	356	B	Cs	a	1	2	4
Ma(AB)(CD)(EF)	720	720	0	1	A	C1	c	1	1	720						

*a, b, c, d, refer to monodentate ligands; (AA) and (AB) refer to bidentate ligands, (AA) being symmetrical, and (AB) non-symmetrical, with A and B representing non-equivalent coordinating atoms.

Table S6. Hemicube. Total is the number of stereoisomers for the corresponding shape and formula, of which c is the number of chiral and a is the number of achiral stereoisomers. RCR is the random coordination ratio. All stereoisomers are classified into subsets according to their point groups and ordered in terms of the product of the number # of distinct stereoisomers by their corresponding random coordination weights, RCWs. χ identifies chirality of the coordination polyhedra of all stereoisomers of the subset, with a meaning that they are achiral, and c meaning that they are chiral; and σ is the rotational symmetry number.

HECU-7 Hemicube

Formula	Set			**RCR**	Subsets											
	Total	c	a		Subset	Group	χ	σ	RCW	#	Subset	Group	χ	σ	RCW	#
Ma7	1	0	1	1	A	C2v	a	2	2520	1						
Ma6b	4	2	2	4:2:1	A	C1	c	1	720	2	B	Cs	a	1	720	1
					C	C2v	a	2	360	1						
Ma5b2	12	8	4	12:6:2:1	A	C1	c	1	240	6	B	Cs	a	1	240	3
					C	C2	c	2	120	2	D	C2v	a	2	120	1
Ma5bc	21	18	3	6:1	A	C1	c	1	120	18	B	Cs	a	1	120	3
Ma5(AA)	6	4	2	8:2:1	A	C1	c	1	240	4	B	Cs	a	1	240	1
					C	C2v	a	2	120	1						
Ma5(AB)	11	10	1	10:1	A	C1	c	1	120	10	B	Cs	a	1	120	1
Ma4b3	19	14	5	24:8:2:1	A	C1	c	1	144	12	B	Cs	a	1	144	4
					C	C2	c	2	72	2	D	C2v	a	2	72	1
Ma4b2c	54	48	6	92:10:2:1	A	C1	c	1	48	46	B	Cs	a	1	48	5
					C	C2	c	2	24	2	D	C2v	a	2	24	1
Ma4bcd	105	102	3	34:1	A	C1	c	1	24	102	B	Cs	a	1	24	3
Ma4b(AA)	28	26	2	52:2:1	A	C1	c	1	48	26	B	Cs	a	1	48	1
					C	C2v	a	2	24	1						
Ma4b(AB)	55	54	1	54:1	A	C1	c	1	24	54	B	Cs	a	1	24	1
Ma3b3c	70	64	6	10.7:1	A	C1	c	1	36	64	B	Cs	a	1	36	6
Ma3b2c2	108	98	10	30.7:3.3:1	A	C1	c	1	24	92	B	Cs	a	1	24	10
					C	C2	c	2	12	6						
Ma3b2cd	210	204	6	34:1	A	C1	c	1	12	204	B	Cs	a	1	12	6
Ma3b2(AA)	56	52	4	50:4:1	A	C1	c	1	24	50	B	Cs	a	1	24	4
					C	C2	c	2	12	2						
Ma3b2(AB)	110	108	2	54:1	A	C1	c	1	12	108	B	Cs	a	1	12	2

Table S6 (continuation).

HECU-7 Hemicube

Formula*	Set			RCR	Subsets											
	Total	c	a		Subset	Group	χ	σ	RCW	#	Subset	Group	χ	σ	RCW	#
Ma3bcde	420	420	0	1	A	C1	c	1	6	420						
Ma3bc(AA)	110	110	0	1	A	C1	c	1	12	110						
Ma3bc(AB)	220	220	0	1	A	C1	c	1	6	220						
Ma3(AA)2	17	14	3	24:4:2:1	A	C1	c	1	48	12	B	Cs	a	1	48	2
					C	C2	c	2	24	2	D	C2v	a	2	24	1
Ma3(AA)(BB)	31	28	3	9.3:1	A	C1	c	1	24	28	B	Cs	a	1	24	3
Ma3(AA)(AB)	62	62	0	1	A	C1	c	1	12	62						
Ma3(AB)2	65	62	3	18.7:1:1	A	C1	c	1	12	56	B'	C2	c	2	6	6
					B''	Cs	a	1	12	3						
Ma3(AB)(CD)	124	124	0	1	A	C1	c	1	6	124						
Ma2b2c2d	318	306	12	100:4:1	A	C1	c	1	8	300	B	Cs	a	1	8	12
					C	C2	c	2	4	6						
Ma2b2cde	630	624	6	104:1	A	C1	c	1	4	624	B	Cs	a	1	4	6
Ma2b2c(AA)	166	162	4	160:4:1	A	C1	c	1	8	160	B	Cs	a	1	8	4
					C	C2	c	2	4	2						
Ma2b2c(AB)	330	328	2	164:1	A	C1	c	1	4	328	B	Cs	a	1	4	2
Ma2bcdef	1260	1260	0	1	A	C1	c	1	2	1260						
Ma2bcd(AA)	330	330	0	1	A	C1	c	1	4	330						
Ma2bcd(AB)	660	660	0	1	A	C1	c	1	2	660						
Ma2b(AA)2	48	44	4	84:6:2:1	A	C1	c	1	16	42	B	Cs	a	1	16	3
					C	C2	c	2	8	2	D	C2v	a	2	8	1
Ma2b(AA)(BB)	93	90	3	30:1	A	C1	c	1	8	90	B	Cs	a	1	8	3
Ma2b(AA)(AB)	186	186	0	1	A	C1	c	1	4	186						
Ma2b(AB)2	189	184	5	59.3:1.7:1	A	C1	c	1	4	178	B	Cs	a	1	4	5
					C	C2	c	2	2	6						
Ma2b(AB)(CD)	372	372	0	1	A	C1	c	1	2	372						
Mabcdefg	2520	2520	0	1	A	C1	c	1	1	2520						
Mabcde(AA)	660	660	0	1	A	C1	c	1	2	660						

Table S6 (continuation).

HECU-7 Hemicube

Formula*	Set			**RCR**	Subsets											
	Total	c	A		Subset	Group	χ	σ	RCW	#	Subset	Group	χ	σ	RCW	#
Mabcde(AB)	1320	1320	0	1	A	C1	c	1	1	1320						
Mabc(AA)2	93	90	3	30:1	A	C1	c	1	8	90	B	Cs	a	1	8	3
Mabc(AA)(BB)	186	186	0	1	A	C1	c	1	4	186						
Mabc(AA)(AB)	372	372	0	1	A	C1	c	1	2	372						
Mabc(AB)2	372	366	6	61:1	A	C1	c	1	2	366	B	Cs	a	1	2	6
Mabc(AB)(CD)	744	744	0	1	A	C1	c	1	1	744						
Ma(AA)3	10	8	2	16:2:1	A	C1	c	1	48	8	B	Cs	a	1	48	1
					C	C2v	a	2	24	1						
Ma(AA)2(BB)	29	26	3	52:4:1	A	C1	c	1	16	26	B	Cs	a	1	16	2
					C	C2v	a	2	8	1						
Ma(AA)2(AB)	57	56	1	56:1	A	C1	c	1	8	56	B	Cs	a	1	8	1
Ma(AA)(BB)(CC)	57	54	3	18:1	A	C1	c	1	8	54	B	Cs	a	1	8	3
Ma(AA)(BB)(AB)	114	114	0	1	A	C1	c	1	4	114						
Ma(AA)(AB)2	115	112	3	110:3:1	A	C1	c	1	4	110	B	Cs	a	1	4	3
					C	C2	c	2	2	2						
Ma(AA)(AB)(CD)	228	228	0	1	A	C1	c	1	2	228						
Ma(AB)3	76	74	2	37:1	A	C1	c	1	6	74	B	Cs	a	1	6	2
Ma(AB)2(CD)	228	226	2	113:1	A	C1	c	1	2	226	B	Cs	a	1	2	2
Ma(AB)(CD)(EF)	456	456	0	1	A	C1	c	1	1	456						

*a, b, c, d, refer to monodentate ligands; (AA) and (AB) refer to bidentate ligands, (AA) being symmetrical, and (AB) non-symmetrical, with A and B representing non-equivalent coordinating atoms.

Table S7. Digonal pseudo-anticupola. Total is the number of stereoisomers for the corresponding shape and formula, of which c is the number of chiral and a is the number of achiral stereoisomers. RCR is the random coordination ratio. All stereoisomers are classified into subsets according to their point groups and ordered in terms of the product of the number # of distinct stereoisomers by their corresponding random coordination weights, RCWs. χ identifies chirality of the coordination polyhedra of all stereoisomers of the subset, with a meaning that they are achiral, and c meaning that they are chiral; and σ is the rotational symmetry number.

DPAC-7 Digonal pseudo-anticupola

Formula	Set			RCR	Subsets											
	Total	c	a		Subset	Group	χ	σ	RCW	#	Subset	Group	χ	σ	RCW	#
Ma7	1	0	1	1	A	Cs	a	1	5040	1						
Ma6b	7	4	3	1.3:1	A	C1	c	1	720	4	B	Cs	a	1	720	3
Ma5b2	21	16	5	3.2:1	A	C1	c	1	240	16	B	Cs	a	1	240	5
Ma5bc	42	36	6	6:1	A	C1	c	1	120	36	B	Cs	a	1	120	6
Ma5(AA)	13	10	3	3.3:1	A	C1	c	1	240	10	B	Cs	a	1	240	3
Ma5(AB)	26	22	4	5.5:1	A	C1	c	1	120	22	B	Cs	a	1	120	4
Ma4b3	35	28	7	4:1	A	C1	c	1	144	28	B	Cs	a	1	144	7
Ma4b2c	105	96	9	10.7:1	A	C1	c	1	48	96	B	Cs	a	1	48	9
Ma4bcd	210	204	6	34:1	A	C1	c	1	24	204	B	Cs	a	1	24	6
Ma4b(AA)	65	60	5	12:1	A	C1	c	1	48	60	B	Cs	a	1	48	5
Ma4b(AB)	130	126	4	31.5:1	A	C1	c	1	24	126	B	Cs	a	1	24	4
Ma3b3c	140	128	12	10.7:1	A	C1	c	1	36	128	B	Cs	a	1	36	12
Ma3b2c2	210	196	14	14:1	A	C1	c	1	24	196	B	Cs	a	1	24	14
Ma3b2cd	420	408	12	34:1	A	C1	c	1	12	408	B	Cs	a	1	12	12
Ma3b2(AA)	130	122	8	15.25:1	A	C1	c	1	24	122	B	Cs	a	1	24	8
Ma3b2(AB)	260	252	8	31.5:1	A	C1	c	1	12	252	B	Cs	a	1	12	8
Ma3bcde	840	840	0	1	A	C1	c	1	6	840						
Ma3bc(AA)	260	254	6	42.3:1	A	C1	c	1	12	254	B	Cs	a	1	12	6
Ma3bc(AB)	520	520	0	1	A	C1	c	1	6	520						
Ma3(AA)2	41	38	3	12.7:1	A	C1	c	1	48	38	B	Cs	a	1	48	3
Ma3(AA)(BB)	82	78	4	19.5:1	A	C1	c	1	24	78	B	Cs	a	1	24	4
Ma3(AA)(AB)	164	160	4	40:1	A	C1	c	1	12	160	B	Cs	a	1	12	4
Ma3(AB)2	164	162	2	81:1	A	C1	c	1	12	162	B	Cs	a	1	12	2
Ma3(AB)(CD)	328	328	0	1	A	C1	c	1	6	328						

Table S7 (continuation).

DPAC-7 Digonal pseudo-anticupola

Formula*	Set			RCR	Subsets											
	Total	c	a		Subset	Group	χ	σ	RCW	#	Subset	Group	χ	σ	RCW	#
Ma2b2c2d	630	612	18	34:1	A	C1	c	1	8	612	B	Cs	a	1	8	18
Ma2b2cde	1260	1248	12	104:1	A	C1	c	1	4	1248	B	Cs	a	1	4	12
Ma2b2c(AA)	390	380	10	38:1	A	C1	c	1	8	380	B	Cs	a	1	8	10
Ma2b2c(AB)	780	772	8	96.5:1	A	C1	c	1	4	772	B	Cs	a	1	4	8
Ma2bcdef	2520	2520	0	1	A	C1	c	1	2	2520						
Ma2bcd(AA)	780	774	6	129:1	A	C1	c	1	4	774	B	Cs	a	1	4	6
Ma2bcd(AB)	1560	1560	0	1	A	C1	c	1	2	1560						
Ma2b(AA)2	123	118	5	23.6:1	A	C1	c	1	16	118	B	Cs	a	1	16	5
Ma2b(AA)(BB)	246	242	4	60.5:1	A	C1	c	1	8	242	B	Cs	a	1	8	4
Ma2b(AA)(AB)	492	488	4	122:1	A	C1	c	1	4	488	B	Cs	a	1	4	4
Ma2b(AB)2	492	486	6	81:1	A	C1	c	1	4	486	B	Cs	a	1	4	6
Ma2b(AB)(CD)	984	984	0	1	A	C1	c	1	2	984						
Mabcdefg	5040	5040	0	1	A	C1	c	1	1	5040						
Mabcde(AA)	1560	1560	0	1	A	C1	c	1	2	1560						
Mabcde(AB)	3120	3120	0	1	A	C1	c	1	1	3120						
Mabc(AA)2	246	240	6	40:1	A	C1	c	1	8	240	B	Cs	a	1	8	6
Mabc(AA)(BB)	492	492	0	1	A	C1	c	1	4	492						
Mabc(AA)(AB)	984	984	0	1	A	C1	c	1	2	984						
Mabc(AB)2	984	972	12	81:1	A	C1	c	1	2	972	B	Cs	a	1	2	12
Mabc(AB)(CD)	1968	1968	0	1	A	C1	c	1	1	1968						
Ma(AA)3	26	24	2	12:1	A	C1	c	1	48	24	B	Cs	a	1	48	2
Ma(AA)2(BB)	78	76	2	38:1	A	C1	c	1	16	76	B	Cs	a	1	16	2
Ma(AA)2(AB)	156	152	4	38:1	A	C1	c	1	8	152	B	Cs	a	1	8	4
Ma(AA)(BB)(CC)	156	156	0	1	A	C1	c	1	8	156						
Ma(AA)(BB)(AB)	312	312	0	1	A	C1	c	1	4	312						
Ma(AA)(AB)2	312	308	4	77:1	A	C1	c	1	4	308	B	Cs	a	1	4	4
Ma(AA)(AB)(CD)	624	624	0	1	A	C1	c	1	2	624						

Table S7 (continuation).

DPAC-7 Digonal pseudo-anticupola

Formula*	Set			RCR	Subsets											
	Total	c	a		Subset	Group	χ	σ	RCW	#	Subset	Group	χ	σ	RCW	#
Ma(AB)3	208	200	8	25:1	A	C1	c	1	6	200	B	Cs	a	1	6	8
Ma(AB)2(CD)	624	616	8	77:1	A	C1	c	1	2	616	B	Cs	a	1	2	8
Ma(AB)(CD)(EF)	1248	1248	0	1	A	C1	c	1	1	1248						

*a, b, c, d, refer to monodentate ligands; (AA) and (AB) refer to bidentate ligands, (AA) being symmetrical, and (AB) non-symmetrical, with A and B representing non-equivalent coordinating atoms.

Table S8. Tetragon-substituted square pyramid. Total is the number of stereoisomers for the corresponding shape and formula, of which c is the number of chiral and a is the number of achiral stereoisomers. RCR is the random coordination ratio. All stereoisomers are classified into subsets according to their point groups and ordered in terms of the product of the number # of distinct stereoisomers by their corresponding random coordination weights, RCWs. χ identifies chirality of the coordination polyhedra of all stereoisomers of the subset, with a meaning that they are achiral, and c meaning that they are chiral; and σ is the rotational symmetry number.

TSPPY-7 Tetragon-substituted pentagonal pyramid

Formula	Set			**RCR**	Subsets											
	Total	c	a		Subset	Group	χ	σ	RCW	#	Subset	Group	χ	σ	RCW	#
Ma7	1	0	1	1	A	Cs	a	1	5040	1						
Ma6b	7	6	1	6:1	A	C1	c	1	720	6	B	Cs	a	1	720	1
Ma5b2	21	18	3	6:1	A	C1	c	1	240	18	B	Cs	a	1	240	3
Ma5bc	42	42	0	1	A	C1	c	1	120	42						
Ma5(AA)	12	10	2	5:1	A	C1	c	1	240	10	B	Cs	a	1	240	2
Ma5(AB)	24	24	0	1	A	C1	c	1	120	24						
Ma4b3	35	32	3	10.7:1	A	C1	c	1	144	32	B	Cs	a	1	144	3
Ma4b2c	105	102	3	34:1	A	C1	c	1	48	102	B	Cs	a	1	48	3
Ma4bcd	210	210	0	1	A	C1	c	1	24	210						
Ma4b(AA)	60	58	2	29:1	A	C1	c	1	48	58	B	Cs	a	1	48	2
Ma4b(AB)	120	120	0	1	A	C1	c	1	24	120						
Ma3b3c	140	140	0	1	A	C1	c	1	36	140						
Ma3b2c2	210	204	6	34:1	A	C1	c	1	24	204	B	Cs	a	1	24	6
Ma3b2cd	420	420	0	1	A	C1	c	1	12	420						
Ma3b2(AA)	120	116	4	29:1	A	C1	c	1	24	116	B	Cs	a	1	24	4
Ma3b2(AB)	240	240	0	1	A	C1	c	1	12	240						
Ma3bcde	840	840	0	1	A	C1	c	1	6	840						
Ma3bc(AA)	240	240	0	1	A	C1	c	1	12	240						
Ma3bc(AB)	480	480	0	1	A	C1	c	1	6	480						
Ma3(AA)2	36	32	4	8:1	A	C1	c	1	48	32	B	Cs	a	1	48	4
Ma3(AA)(BB)	72	70	2	35:1	A	C1	c	1	24	70	B	Cs	a	1	24	2
Ma3(AA)(AB)	144	144	0	1	A	C1	c	1	12	144						
Ma3(AB)2	144	138	6	23:1	A	C1	c	1	12	138	B	Cs	a	1	12	6
Ma3(AB)(CD)	288	288	0	1	A	C1	c	1	6	288						

Table S8 (continuation).

TSPPY-7 Tetragon-substituted pentagonal pyramid

Formula*	Set			RCR	Subsets											
	Total	c	a		Subset	Group	χ	σ	RCW	#	Subset	Group	χ	σ	RCW	#
Ma2b2c2d	630	624	6	104:1	A	C1	c	1	8	624	B	Cs	a	1	8	6
Ma2b2cde	1260	1260	0	1	A	C1	c	1	4	1260						
Ma2b2c(AA)	360	356	4	89:1	A	C1	c	1	8	356	B	Cs	a	1	8	4
Ma2b2c(AB)	720	720	0	1	A	C1	c	1	4	720						
Ma2bcdef	2520	2520	0	1	A	C1	c	1	2	2520						
Ma2bcd(AA)	720	720	0	1	A	C1	c	1	4	720						
Ma2bcd(AB)	1440	1440	0	1	A	C1	c	1	2	1440						
Ma2b(AA)2	108	104	4	26:1	A	C1	c	1	16	104	B	Cs	a	1	16	4
Ma2b(AA)(BB)	216	214	2	107:1	A	C1	c	1	8	214	B	Cs	a	1	8	2
Ma2b(AA)(AB)	432	432	0	1	A	C1	c	1	4	432						
Ma2b(AB)2	432	426	6	71:1	A	C1	c	1	4	426	B	Cs	a	1	4	6
Ma2b(AB)(CD)	864	864	0	1	A	C1	c	1	2	864						
Mabcdefg	5040	5040	0	1	A	C1	c	1	1	5040						
Mabcde(AA)	1440	1440	0	1	A	C1	c	1	2	1440						
Mabcde(AB)	2880	2880	0	1	A	C1	c	1	1	2880						
Mabc(AA)2	216	216	0	1	A	C1	c	1	8	216						
Mabc(AA)(BB)	432	432	0	1	A	C1	c	1	4	432						
Mabc(AA)(AB)	864	864	0	1	A	C1	c	1	2	864						
Mabc(AB)2	864	864	0	1	A	C1	c	1	2	864						
Mabc(AB)(CD)	1728	1728	0	1	A	C1	c	1	1	1728						
Ma(AA)3	22	20	2	10:1	A	C1	c	1	48	20	B	Cs	a	1	48	2
Ma(AA)2(BB)	66	64	2	32:1	A	C1	c	1	16	64	B	Cs	a	1	16	2
Ma(AA)2(AB)	132	132	0	1	A	C1	c	1	8	132						
Ma(AA)(BB)(CC)	132	132	0	1	A	C1	c	1	8	132						
Ma(AA)(BB)(AB)	264	264	0	1	A	C1	c	1	4	264						
Ma(AA)(AB)2	264	260	4	65:1	A	C1	c	1	4	260	B	Cs	a	1	4	4
Ma(AA)(AB)(CD)	528	528	0	1	A	C1	c	1	2	528						

Table S8 (continuation).

TSPPY-7 Tetragon-substituted pentagonal pyramid

Formula*	Set			RCR	Subsets											
	Total	c	a		Subset	Group	χ	σ	RCW	#	Subset	Group	χ	σ	RCW	#
Ma(AB)3	176	176	0	1	A	C1	c	1	6	176						
Ma(AB)2(CD)	528	528	0	1	A	C1	c	1	2	528						
Ma(AB)(CD)(EF)	1056	1056	0	1	A	C1	c	1	1	1056						

*a, b, c, d, refer to monodentate ligands; (AA) and (AB) refer to bidentate ligands, (AA) being symmetrical, and (AB) non-symmetrical, with A and B representing non-equivalent coordinating atoms.

Table S9. Hemiobelisk. Total is the number of stereoisomers for the corresponding shape and formula, of which c is the number of chiral and a is the number of achiral stereoisomers. RCR is the random coordination ratio. All stereoisomers are classified into subsets according to their point groups and ordered in terms of the product of the number # of distinct stereoisomers by their corresponding random coordination weights, RCWs. χ identifies chirality of the coordination polyhedra of all stereoisomers of the subset, with a meaning that they are achiral, and c meaning that they are chiral; and σ is the rotational symmetry number.

HEOB-7 Hemiobelisk

Formula	Set			RCR	Subsets											
	Total	c	a		Subset	Group	χ	σ	RCW	#	Subset	Group	χ	σ	RCW	#
Ma7	1	0	1	1	A	Cs	a	1	5040	1						
Ma6b	7	4	3	1.3:1	A	C1	c	1	720	4	B	Cs	a	1	720	3
Ma5b2	21	16	5	3.2:1	A	C1	c	1	240	16	B	Cs	a	1	240	5
Ma5bc	42	36	6	6:1	A	C1	c	1	120	36	B	Cs	a	1	120	6
Ma5(AA)	11	8	3	2.7:1	A	C1	c	1	240	8	B	Cs	a	1	240	3
Ma5(AB)	22	18	4	4.5:1	A	C1	c	1	120	18	B	Cs	a	1	120	4
Ma4b3	35	28	7	4:1	A	C1	c	1	144	28	B	Cs	a	1	144	7
Ma4b2c	105	96	9	10.7:1	A	C1	c	1	48	96	B	Cs	a	1	48	9
Ma4bcd	210	204	6	34:1	A	C1	c	1	24	204	B	Cs	a	1	24	6
Ma4b(AA)	55	50	5	10:1	A	C1	c	1	48	50	B	Cs	a	1	48	5
Ma4b(AB)	110	106	4	26.5:1	A	C1	c	1	24	106	B	Cs	a	1	24	4
Ma3b3c	140	128	12	10.7:1	A	C1	c	1	36	128	B	Cs	a	1	36	12
Ma3b2c2	210	196	14	14:1	A	C1	c	1	24	196	B	Cs	a	1	24	14
Ma3b2cd	420	408	12	34:1	A	C1	c	1	12	408	B	Cs	a	1	12	12
Ma3b2(AA)	110	102	8	12.75:1	A	C1	c	1	24	102	B	Cs	a	1	24	8
Ma3b2(AB)	220	212	8	26.5:1	A	C1	c	1	12	212	B	Cs	a	1	12	8
Ma3bcde	840	840	0	1	A	C1	c	1	6	840						
Ma3bc(AA)	220	214	6	35.7:1	A	C1	c	1	12	214	B	Cs	a	1	12	6
Ma3bc(AB)	440	440	0	1	A	C1	c	1	6	440						
Ma3(AA)2	31	28	3	9.3:1	A	C1	c	1	48	28	B	Cs	a	1	48	3
Ma3(AA)(BB)	62	58	4	14.5:1	A	C1	c	1	24	58	B	Cs	a	1	24	4
Ma3(AA)(AB)	124	120	4	30:1	A	C1	c	1	12	120	B	Cs	a	1	12	4
Ma3(AB)2	124	122	2	61:1	A	C1	c	1	12	122	B	Cs	a	1	12	2
Ma3(AB)(CD)	248	248	0	1	A	C1	c	1	6	248						

Table S9 (continuation).

HEOB-7 Hemiobelisk

Formula*	Set			RCR	Subsets											
	Total	c	a		Subset	Group	χ	σ	RCW	#	Subset	Group	χ	σ	RCW	#
Ma2b2c2d	630	612	18	34:1	A	C1	c	1	8	612	B	Cs	a	1	8	18
Ma2b2cde	1260	1248	12	104:1	A	C1	c	1	4	1248	B	Cs	a	1	4	12
Ma2b2c(AA)	330	320	10	32:1	A	C1	c	1	8	320	B	Cs	a	1	8	10
Ma2b2c(AB)	660	652	8	81.5:1	A	C1	c	1	4	652	B	Cs	a	1	4	8
Ma2bcdef	2520	2520	0	1	A	C1	c	1	2	2520						
Ma2bcd(AA)	660	654	6	109:1	A	C1	c	1	4	654	B	Cs	a	1	4	6
Ma2bcd(AB)	1320	1320	0	1	A	C1	c	1	2	1320						
Ma2b(AA)2	93	88	5	17.6:1	A	C1	c	1	16	88	B	Cs	a	1	16	5
Ma2b(AA)(BB)	186	182	4	45.5:1	A	C1	c	1	8	182	B	Cs	a	1	8	4
Ma2b(AA)(AB)	372	368	4	92:1	A	C1	c	1	4	368	B	Cs	a	1	4	4
Ma2b(AB)2	372	366	6	61:1	A	C1	c	1	4	366	B	Cs	a	1	4	6
Ma2b(AB)(CD)	744	744	0	1	A	C1	c	1	2	744						
Mabcdefg	5040	5040	0	1	A	C1	c	1	1	5040						
Mabcde(AA)	1320	1320	0	1	A	C1	c	1	2	1320						
Mabcde(AB)	2640	2640	0	1	A	C1	c	1	1	2640						
Mabc(AA)2	186	180	6	30:1	A	C1	c	1	8	180	B	Cs	a	1	8	6
Mabc(AA)(BB)	372	372	0	1	A	C1	c	1	4	372						
Mabc(AA)(AB)	744	744	0	1	A	C1	c	1	2	744						
Mabc(AB)2	744	732	12	61:1	A	C1	c	1	2	732	B	Cs	a	1	2	12
Mabc(AB)(CD)	1488	1488	0	1	A	C1	c	1	1	1488						
Ma(AA)3	18	16	2	8:1	A	C1	c	1	48	16	B	Cs	a	1	48	2
Ma(AA)2(BB)	54	52	2	26:1	A	C1	c	1	16	52	B	Cs	a	1	16	2
Ma(AA)2(AB)	108	104	4	26:1	A	C1	c	1	8	104	B	Cs	a	1	8	4
Ma(AA)(BB)(CC)	108	108	0	1	A	C1	c	1	8	108						
Ma(AA)(BB)(AB)	216	216	0	1	A	C1	c	1	4	216						
Ma(AA)(AB)2	216	212	4	53:1	A	C1	c	1	4	212	B	Cs	a	1	4	4
Ma(AA)(AB)(CD)	432	432	0	1	A	C1	c	1	2	432						

Table S9 (continuation).

HEOB-7 Hemiobelisk

Formula*	Set			**RCR**	Subsets											
	Total	c	a		Subset	Group	χ	σ	RCW	#	Subset	Group	χ	σ	RCW	#
Ma(AB)3	144	136	8	17:1	A	C1	c	1	6	136	B	Cs	a	1	6	8
Ma(AB)2(CD)	432	424	8	53:1	A	C1	c	1	2	424	B	Cs	a	1	2	8
Ma(AB)(CD)(EF)	864	864	0	1	A	C1	c	1	1	864						

*a, b, c, d, refer to monodentate ligands; (AA) and (AB) refer to bidentate ligands, (AA) being symmetrical, and (AB) non-symmetrical, with A and B representing non-equivalent coordinating atoms.

Table S10. Tetragonal helicoid with tetragonal base. Total is the number of stereoisomers for the corresponding shape and formula, of which c is the number of chiral and a is the number of achiral stereoisomers. RCR is the random coordination ratio. All stereoisomers are classified into subsets according to their point groups and ordered in terms of the product of the number # of distinct stereoisomers by their corresponding random coordination weights, RCWs. χ identifies chirality of the coordination polyhedra of all stereoisomers of the subset, with a meaning that they are achiral, and c meaning that they are chiral; and σ is the rotational symmetry number.

Δ/Λ THTB-7 Tetragonal helicoid with tetragonal base

Formula	Set			**RCR**	Subsets											
	Total	c	a		Subset	Group	χ	σ	RCW	#	Subset	Group	χ	σ	RCW	#
Ma7	1	1	0	1	A	C2	c	2	2520	1						
Ma6b	4	4	0	6:1	A	C1	c	1	720	3	B	C2	c	2	360	1
Ma5b2	12	12	0	6:1	A	C1	c	1	240	9	B	C2	c	2	120	3
Ma5bc	21	21	0	1	A	C1	c	1	120	21						
Ma5(AA)	6	6	0	1	A	C1	c	1	240	6						
Ma5(AB)	12	12	0	1	A	C1	c	1	120	12						
Ma4b3	19	19	0	10.7:1	A	C1	c	1	144	16	B	C2	c	2	72	3
Ma4b2c	54	54	0	34:1	A	C1	c	1	48	51	B	C2	c	2	24	3
Ma4bcd	105	105	0	1	A	C1	c	1	24	105						
Ma4b(AA)	30	30	0	1	A	C1	c	1	48	30						
Ma4b(AB)	60	60	0	1	A	C1	c	1	24	60						
Ma3b3c	70	70	0	1	A	C1	c	1	36	70						
Ma3b2c2	108	108	0	34:1	A	C1	c	1	24	102	B	C2	c	2	12	6
Ma3b2cd	210	210	0	1	A	C1	c	1	12	210						
Ma3b2(AA)	60	60	0	1	A	C1	c	1	24	60						
Ma3b2(AB)	120	120	0	1	A	C1	c	1	12	120						
Ma3bcde	420	420	0	1	A	C1	c	1	6	420						
Ma3bc(AA)	120	120	0	1	A	C1	c	1	12	120						
Ma3bc(AB)	240	240	0	1	A	C1	c	1	6	240						
Ma3(AA)2	20	20	0	8:1	A	C1	c	1	48	16	B	C2	c	2	24	4
Ma3(AA)(BB)	36	36	0	1	A	C1	c	1	24	36						
Ma3(AA)(AB)	72	72	0	1	A	C1	c	1	12	72						
Ma3(AB)2	76	76	0	17:1	A	C1	c	1	12	68	B	C2	c	2	6	8
Ma3(AB)(CD)	144	144	0	1	A	C1	c	1	6	144						

Table S10 (continuation).

Δ/Λ THTB-7 Tetragonal helicoid with tetragonal base

Formula*	Set			RCR	Subsets											
	Total	c	a		Subset	Group	χ	σ	RCW	#	Subset	Group	χ	σ	RCW	#
Ma2b2c2d	318	318	0	104:1	A	C1	c	1	8	312	B	C2	c	2	4	6
Ma2b2cde	630	630	0	1	A	C1	c	1	4	630						
Ma2b2c(AA)	180	180	0	1	A	C1	c	1	8	180						
Ma2b2c(AB)	360	360	0	1	A	C1	c	1	4	360						
Ma2bcdef	1260	1260	0	1	A	C1	c	1	2	1260						
Ma2bcd(AA)	360	360	0	1	A	C1	c	1	4	360						
Ma2bcd(AB)	720	720	0	1	A	C1	c	1	2	720						
Ma2b(AA)2	56	56	0	26:1	A	C1	c	1	16	52	B	C2	c	2	8	4
Ma2b(AA)(BB)	108	108	0	1	A	C1	c	1	8	108						
Ma2b(AA)(AB)	216	216	0	1	A	C1	c	1	4	216						
Ma2b(AB)2	220	220	0	53:1	A	C1	c	1	4	212	B	C2	c	2	2	8
Ma2b(AB)(CD)	432	432	0	1	A	C1	c	1	2	432						
Mabcdefg	2520	2520	0	1	A	C1	c	1	1	2520						
Mabcde(AA)	720	720	0	1	A	C1	c	1	2	720						
Mabcde(AB)	1440	1440	0	1	A	C1	c	1	1	1440						
Mabc(AA)2	108	108	0	1	A	C1	c	1	8	108						
Mabc(AA)(BB)	216	216	0	1	A	C1	c	1	4	216						
Mabc(AA)(AB)	432	432	0	1	A	C1	c	1	2	432						
Mabc(AB)2	432	432	0	1	A	C1	c	1	2	432						
Mabc(AB)(CD)	864	864	0	1	A	C1	c	1	1	864						
Ma(AA)3	11	11	0	1	A	C1	c	1	48	11						
Ma(AA)2(BB)	33	33	0	1	A	C1	c	1	16	33						
Ma(AA)2(AB)	66	66	0	1	A	C1	c	1	8	66						
Ma(AA)(BB)(CC)	66	66	0	1	A	C1	c	1	8	66						
Ma(AA)(BB)(AB)	132	132	0	1	A	C1	c	1	4	132						
Ma(AA)(AB)2	132	132	0	1	A	C1	c	1	4	132						
Ma(AA)(AB)(CD)	264	264	0	1	A	C1	c	1	2	264						

Table S10 (continuation).

Δ/Λ THTB-7 Tetragonal helicoid with tetragonal base

Formula*	Set			RCR	Subsets											
	Total	c	a		Subset	Group	χ	σ	RCW	#	Subset	Group	χ	σ	RCW	#
Ma(AB)3	88	88	0	1	A	C1	c	1	6	88						
Ma(AB)2(CD)	264	264	0	1	A	C1	c	1	2	264						
Ma(AB)(CD)(EF)	528	528	0	1	A	C1	c	1	1	528						

*a, b, c, d, refer to monodentate ligands; (AA) and (AB) refer to bidentate ligands, (AA) being symmetrical, and (AB) non-symmetrical, with A and B representing non-equivalent coordinating atoms.

Table S11. Square hemiantiprism. Total is the number of stereoisomers for the corresponding shape and formula, of which c is the number of chiral and a is the number of achiral stereoisomers. RCR is the random coordination ratio. All stereoisomers are classified into subsets according to their point groups and ordered in terms of the product of the number # of distinct stereoisomers by their corresponding random coordination weights, RCWs. χ identifies chirality of the coordination polyhedra of all stereoisomers of the subset, with a meaning that they are achiral, and c meaning that they are chiral; and σ is the rotational symmetry number.

Δ/Λ SHEAPR-7 Square hemiantiprism

Formula	Set			RCR	Subsets											
	Total	c	a		Subset	Group	χ	σ	RCW	#	Subset	Group	χ	σ	RCW	#
Ma7	1	1	0	1	A	C2	c	2	2520	1						
Ma6b	4	4	0	6:1	A	C1	c	1	720	3	B	C2	c	2	360	1
Ma5b2	12	12	0	6:1	A	C1	c	1	240	9	B	C2	c	2	120	3
Ma5bc	21	21	0	1	A	C1	c	1	120	21						
Ma5(AA)	7	7	0	12:1	A	C1	c	1	240	6	B	C2	c	2	120	1
Ma5(AB)	13	13	0	1	A	C1	c	1	120	13						
Ma4b3	19	19	0	10.7:1	A	C1	c	1	144	16	B	C2	c	2	72	3
Ma4b2c	54	54	0	34:1	A	C1	c	1	48	51	B	C2	c	2	24	3
Ma4bcd	105	105	0	1	A	C1	c	1	24	105						
Ma4b(AA)	33	33	0	64:1	A	C1	c	1	48	32	B	C2	c	2	24	1
Ma4b(AB)	65	65	0	1	A	C1	c	1	24	65						
Ma3b3c	70	70	0	1	A	C1	c	1	36	70						
Ma3b2c2	108	108	0	34:1	A	C1	c	1	24	102	B	C2	c	2	12	6
Ma3b2cd	210	210	0	1	A	C1	c	1	12	210						
Ma3b2(AA)	66	66	0	64:1	A	C1	c	1	24	64	B	C2	c	2	12	2
Ma3b2(AB)	130	130	0	1	A	C1	c	1	12	130						
Ma3bcde	420	420	0	1	A	C1	c	1	6	420						
Ma3bc(AA)	130	130	0	1	A	C1	c	1	12	130						
Ma3bc(AB)	260	260	0	1	A	C1	c	1	6	260						
Ma3(AA)2	23	23	0	9.5:1	A	C1	c	1	48	19	B	C2	c	2	24	4
Ma3(AA)(BB)	42	42	0	1	A	C1	c	1	24	42						
Ma3(AA)(AB)	84	84	0	1	A	C1	c	1	12	84						
Ma3(AB)2	88	88	0	20:1	A	C1	c	1	12	80	B	C2	c	2	6	8
Ma3(AB)(CD)	168	168	0	1	A	C1	c	1	6	168						

Table S11 (continuation).

Δ/Λ SHEAPR-7 Square hemiantiprism

Formula*	Set			RCR	Subsets											
	Total	c	a		Subset	Group	χ	σ	RCW	#	Subset	Group	χ	σ	RCW	#
Ma2b2c2d	318	318	0	104:1	A	C1	c	1	8	312	B	C2	c	2	4	6
Ma2b2cde	630	630	0	1	A	C1	c	1	4	630						
Ma2b2c(AA)	196	196	0	194:1	A	C1	c	1	8	194	B	C2	c	2	4	2
Ma2b2c(AB)	390	390	0	1	A	C1	c	1	4	390						
Ma2bcdef	1260	1260	0	1	A	C1	c	1	2	1260						
Ma2bcd(AA)	390	390	0	1	A	C1	c	1	4	390						
Ma2bcd(AB)	780	780	0	1	A	C1	c	1	2	780						
Ma2b(AA)2	65	65	0	30.5:1	A	C1	c	1	16	61	B	C2	c	2	8	4
Ma2b(AA)(BB)	126	126	0	1	A	C1	c	1	8	126						
Ma2b(AA)(AB)	252	252	0	1	A	C1	c	1	4	252						
Ma2b(AB)2	256	256	0	62:1	A	C1	c	1	4	248	B	C2	c	2	2	8
Ma2b(AB)(CD)	504	504	0	1	A	C1	c	1	2	504						
Mabcdefg	2520	2520	0	1	A	C1	c	1	1	2520						
Mabcde(AA)	780	780	0	1	A	C1	c	1	2	780						
Mabcde(AB)	1560	1560	0	1	A	C1	c	1	1	1560						
Mabc(AA)2	126	126	0	1	A	C1	c	1	8	126						
Mabc(AA)(BB)	252	252	0	1	A	C1	c	1	4	252						
Mabc(AA)(AB)	504	504	0	1	A	C1	c	1	2	504						
Mabc(AB)2	504	504	0	1	A	C1	c	1	2	504						
Mabc(AB)(CD)	1008	1008	0	1	A	C1	c	1	1	1008						
Ma(AA)3	14	14	0	26:1	A	C1	c	1	48	13	B	C2	c	2	24	1
Ma(AA)2(BB)	41	41	0	80:1	A	C1	c	1	16	40	B	C2	c	2	8	1
Ma(AA)2(AB)	81	81	0	1	A	C1	c	1	8	81						
Ma(AA)(BB)(CC)	81	81	0	1	A	C1	c	1	8	81						
Ma(AA)(BB)(AB)	162	162	0	1	A	C1	c	1	4	162						
Ma(AA)(AB)2	163	163	0	161:1	A	C1	c	1	4	161	B	C2	c	2	2	2
Ma(AA)(AB)(CD)	324	324	0	1	A	C1	c	1	2	324						

Table S11 (continuation).

Δ/Λ-SHEAPR-7 Square hemiantiprism

Formula*	Set			RCR	Subsets											
	Total	c	a		Subset	Group	χ	σ	RCW	#	Subset	Group	χ	σ	RCW	#
Ma(AB)3	108	108	0	1	A	C1	c	1	6	108						
Ma(AB)2(CD)	324	324	0	1	A	C1	c	1	2	324						
Ma(AB)(CD)(EF)	648	648	0	1	A	C1	c	1	1	648						

*a, b, c, d, refer to monodentate ligands; (AA) and (AB) refer to bidentate ligands, (AA) being symmetrical, and (AB) non-symmetrical, with A and B representing non-equivalent coordinating atoms.

Decision Trees for Predicting Coordination Chirality in the New Polyhedral Shapes

Next, we present decision trees to analyze the possibility of coordination chirality in the new shapes of coordination polyhedra introduced in this work, considering various ligand types and the complete space of all possible chiral and achiral-at-metal stereoisomers for the corresponding generic formulae within each polyhedral shape. These flowchart structures are a continuation of our recently published study (DOI: https://doi.org/10.1002/jcc.70025). The development of these decision trees addresses a longstanding gap in Coordination Chemistry: the lack of clear systematic guidelines for identifying and predicting the occurrence of coordination chirality. This stands in contrast to the well-established stereochemical rules for tetrahedral molecules in Organic Chemistry. Consistent with our previous work, the decision trees are presented for generic formulae composed exclusively of mono- and/or bidentate ligands. Our decision trees have a top-down-oriented structure and are binary. Each decision node parenting two new branches indicates an optimal local rule by which the probability of a metal complex being chiral-at-metal is most impacted in a subset. These rules systematically partition sets into smaller subsets using specific ligand types and their quantities as parameters. To illustrate, consider the initial decision node in the Digonal Anticupola (DAC-6) decision tree presented on the next page. The splitting rule at this node evaluates the quantity of monodentate ligand 'a'. The right-oriented branch from this node indicates that when a complex contains more than four units of ligand type 'a’, all possible stereoisomers for the corresponding generic formulae (Ma_5b and Ma_6) are exclusively achiral-at-metal, resulting in a metal-centered chirality probability p(**c**) of 0%. Conversely, the left-oriented branch, corresponding to complexes containing four or fewer units of ligand 'a', leads to multiple possible outcomes through subsequent decision nodes with additional splitting rules, where the probability of coordination chirality assumes nonzero values.

For each terminal node (leaf), our decision trees provide information, including the mean probability of metal-centered chirality across all generic formulae within this leaf, the complete string of each formula, and their respective enumeration of chiral-at-metal (**c**) and achiral (**a**) stereoisomers in designated columns.

CN-6 Digonal anticupola (C_{2v})

DAC-6 (C_{2v}) Digonal anticupola

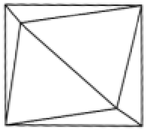

DAC-6 Digonal anticupola

- # a > 4 → Not chiral
- # a ≤ 4 → # a
  - # 2 < a ≤ 4 → # b
    - # b > 1 → # c
      - # c = 1 → 67% chiral
      - # c = 0 → ≈40% chiral
    - # b ≤ 1 → # a
      - # a = 3 → # (AB)
        - # (AB) = 1 → 86% chiral
        - # (AB) = 0 → ≈81% chiral
      - # a = 4 → # b
        - # b = 1 → 53% chiral
        - # b = 0 → # (AB)
          - # (AB) = 1 → 73% chiral
          - # (AB) = 0 → 67% chiral
  - # a ≤ 2 → # b
    - # b = 2 → # c
      - # c = 2 → 75% chiral
      - # c < 2 → # c
        - # c = 1 → 84% chiral
        - # c = 0 → ≈90% chiral
    - # b < 3 → # a
      - # a = 2 → ≈95% chiral
      - # a < 2 → ≈100% chiral

Not chiral	a 4
Ma_6	1
Ma_5b	3

75% chiral	c 36	a 12
$Ma_2b_2c_2$	36	12

84% chiral	c 76	a 14
Ma_2b_2cd	76	14

≈90% chiral	c 90	a 10
$Ma_2b_2(AA)$	30	4
$Ma_2b_2(AB)$	60	6

≈95% chiral	c 590	a 33
$Ma_2(AA)_2$	14	1
$Ma_2(AA)(BB)$	24	1
$Ma_2(AA)(AB)$	48	2
$Ma_2(AB)_2$	52	3
$Ma_2bc(AA)$	62	4
$Ma_2(AB)(CD)$	96	4
$Ma_2bc(AB)$	126	6
Ma_2bcde	168	12

≈100% chiral	c 1664	a 3
$M(AA)_3$	4	0
$M(AA)_2(BB)$	10	0
M(AA)(BB)(CC)	18	0
$M(AA)_2(AB)$	18	0
$Mab(AA)_2$	24	1
$M(AB)_3$	24	0
M(AA)(BB)(AB)	36	0
$M(AA)(AB)_2$	38	0
Mab(AA)(BB)	50	0

Continued on next column →

≈100% chiral	c 1664	a 3
M(AA)(AB)(CD)	72	0
$M(AB)_2(CD)$	72	0
$Mab(AB)_2$	98	2
Mab(AA)(AB)	100	0
Mabcd(AA)	132	0
M(AB)(CD)(EF)	144	0
Mab(AB)(CD)	200	0
Mabcd(AB)	264	0
Mabcdef	360	0

86% chiral	c 38	a 6
$Ma_3b(AB)$	38	6

≈81% chiral	c 66	a 16
$Ma_3b(AA)$	18	4
Ma_3bcd	48	12

67% chiral	c 20	a 10
Ma_3b_2c	20	10

≈40% chiral	c 8	a 11
Ma_3b_3	4	6
Ma_4b_2	4	5

53% chiral	c 8	a 7
Ma_4bc	8	7

73% chiral	c 8	a 3
$Ma_4(AB)$	8	3

67% chiral	c 4	a 2
$Ma_4(AA)$	4	2

DTT-7 (C_{3v}) Diminished trigonal trapezohedron

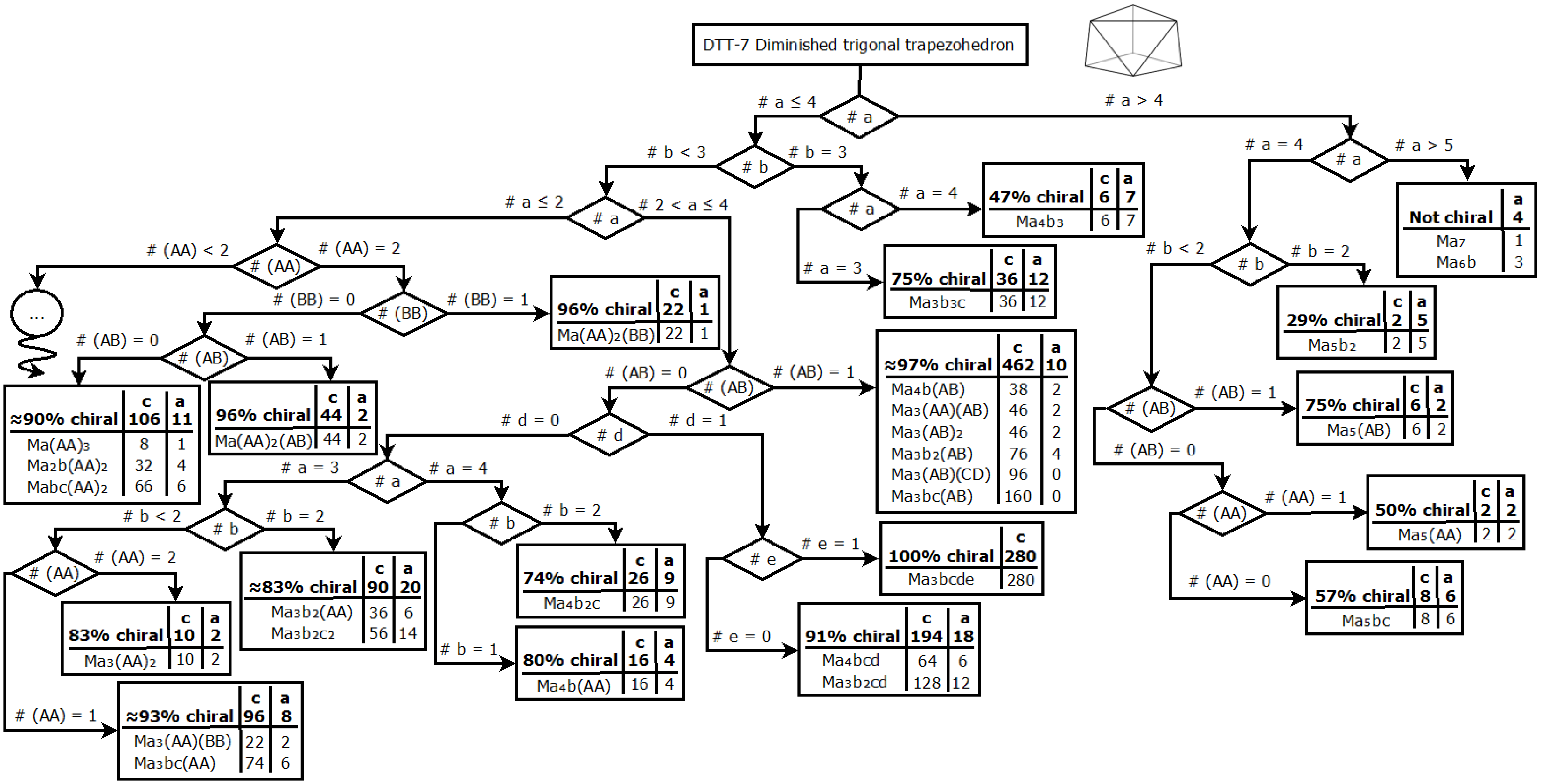

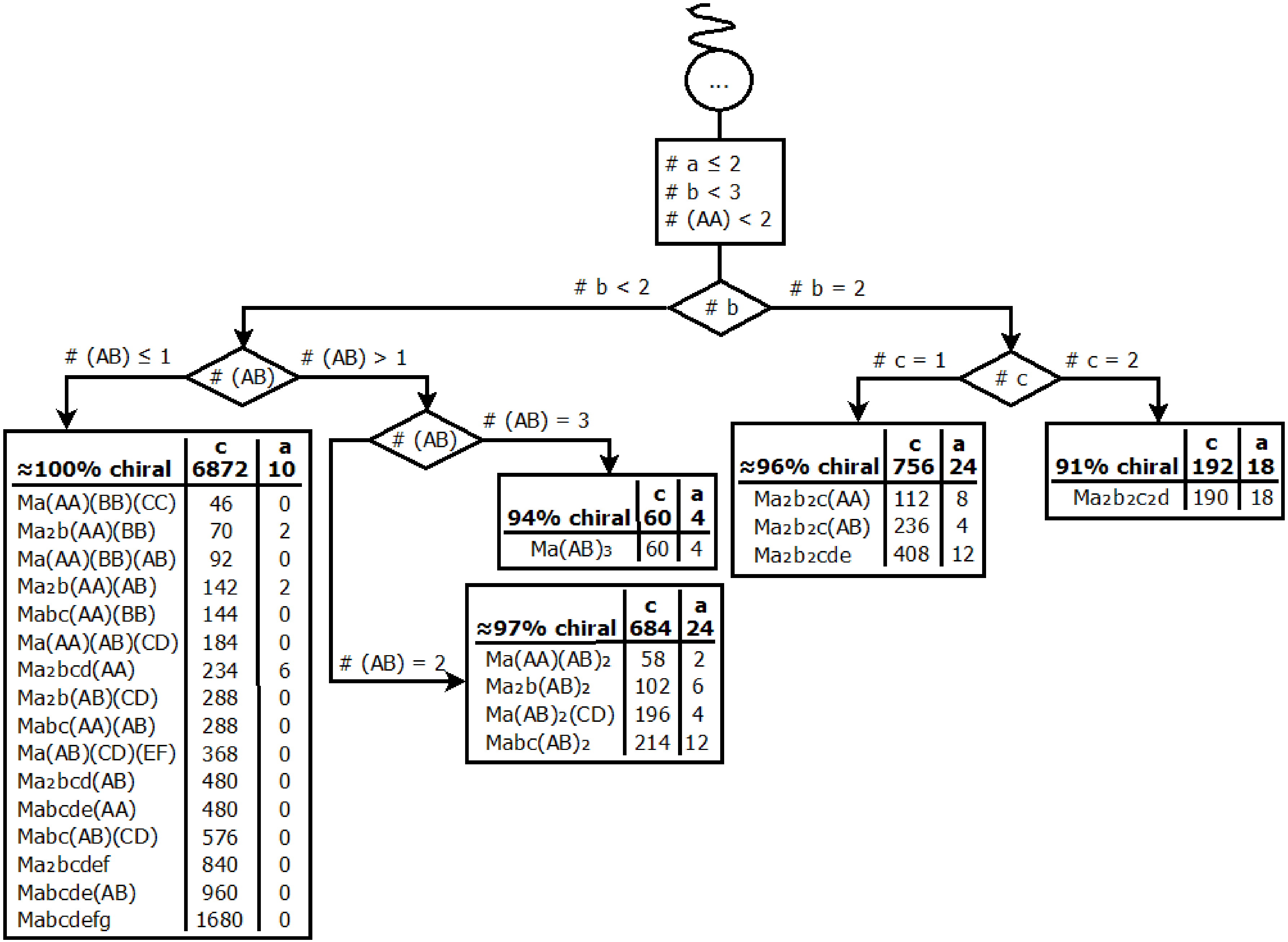
...
a ≤ 2
b < 3
(AA) < 2
b
b < 2
b = 2
(AB)
(AB) ≤ 1
(AB) > 1
(AB)
(AB) = 3
(AB) = 2
c
c = 1
c = 2
≈100% chiral | c 6872 | a 10
Ma(AA)(BB)(CC) | 46 | 0
$Ma_2b(AA)(BB)$ | 70 | 2
Ma(AA)(BB)(AB) | 92 | 0
$Ma_2b(AA)(AB)$ | 142 | 2
Mabc(AA)(BB) | 144 | 0
Ma(AA)(AB)(CD) | 184 | 0
$Ma_2bcd(AA)$ | 234 | 6
$Ma_2b(AB)(CD)$ | 288 | 0
Mabc(AA)(AB) | 288 | 0
Ma(AB)(CD)(EF) | 368 | 0
$Ma_2bcd(AB)$ | 480 | 0
Mabcde(AA) | 480 | 0
Mabc(AB)(CD) | 576 | 0
Ma_2bcdef | 840 | 0
Mabcde(AB) | 960 | 0
Mabcdefg | 1680 | 0
94% chiral | c 60 | a 4
$Ma(AB)_3$ | 60 | 4
≈97% chiral | c 684 | a 24
$Ma(AA)(AB)_2$ | 58 | 2
$Ma_2b(AB)_2$ | 102 | 6
$Ma(AB)_2(CD)$ | 196 | 4
$Mabc(AB)_2$ | 214 | 12
≈96% chiral | c 756 | a 24
$Ma_2b_2c(AA)$ | 112 | 8
$Ma_2b_2c(AB)$ | 236 | 4
Ma_2b_2cde | 408 | 12
91% chiral | c 192 | a 18
$Ma_2b_2c_2d$ | 190 | 18

CN-7 *Trans*-bicapped square pyramid (C_{2v})

TrBCSPY-7 (C_{2v}) Trans-bicapped square pyramid

TrBCSPY-7 Trans-bicapped square pyramid

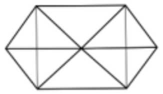

a ≤ 5 → # a

a < 5 → # (AB)

(AB) = 0 → # c

c = 0 → # b

b < 3 → # (BB)

(BB) = 0

≈85% chiral	c 188	a 28
$Ma(AA)_3$	12	3
$Ma_3(AA)_2$	20	5
$Ma_4b(AA)$	30	5
$Ma_3b_2(AA)$	62	8
$Ma_2b(AA)_2$	64	7

(BB) = 1 → # a

a < 3

≈92% chiral	c 274	a 19
$Ma(AA)_2(BB)$	40	5
Ma(AA)(BB)(CC)	84	6
$Ma_2b(AA)(BB)$	130	8

a = 3

87% chiral	c 40	a 6
$Ma_3(AA)(BB)$	40	6

b = 3

74% chiral	c 14	a 5
Ma_4b_3	14	5

c > 0 → # d

d = 1

≈99% chiral	c 6690	a 33
Ma_4bcd	102	3
Ma_3b_2cd	204	6
$Ma_2b_2c_2d$	306	12
$Ma_2bcd(AA)$	414	6
Ma_3bcde	420	0
Ma_2b_2cde	624	6
Mabcde(AA)	840	0
Ma_2bcdef	1260	0
Mabcdefg	2520	0

d = 0 → # (AA)

(AA) = 0

≈90% chiral	c 210	a 22
Ma_4b_2c	48	6
Ma_3b_3c	64	6
$Ma_3b_2c_2$	98	10

(AA) > 0

≈96% chiral	c 736	a 28
$Mabc(AA)_2$	132	6
$Ma_3bc(AA)$	134	6
$Ma_2b_2c(AA)$	200	10
Mabc(AA)(BB)	270	6

(AB) > 0

≈99% chiral	c 9074	a 54
$Ma_4b(AB)$	68	2
$Ma(AA)_2(AB)$	86	4
$Ma_3(AA)(AB)$	88	4
$Ma_3(AB)_2$	92	4
$Ma(AB)_3$	116	4
$Ma_3b_2(AB)$	136	4
$Ma(AA)(AB)_2$	176	4
Ma(AA)(BB)(AB)	176	4
$Ma_3(AB)(CD)$	184	0
$Ma_2b(AA)(AB)$	272	4
$Ma_2b(AB)_2$	274	6

Continued on next column →

≈99% chiral	c 9074	a 54
$Ma_3bc(AB)$	280	0
$Ma(AB)_2(CD)$	356	4
Ma(AA)(AB)(CD)	360	0
$Ma_2b_2c(AB)$	416	4
$Mabc(AB)_2$	546	6
$Ma_2b(AB)(CD)$	552	0
Mabc(AA)(AB)	552	0
Ma(AB)(CD)(EF)	720	0
$Ma_2bcd(AB)$	840	0
Mabc(AB)(CD)	1104	0
Mabcde(AB)	1680	0

a = 5 → # (AA)

(AA) = 1

57% chiral	c 4	a 3
$Ma_5(AA)$	4	3

(AA) = 0 → # b

b = 2

67% chiral	c 8	a 4
Ma_5b_2	8	4

b < 2

86% chiral	c 30	a 5
$Ma_5(AB)$	12	2
Ma_5bc	18	3

a > 5 → # a

a = 7

Not chiral	a 1
Ma_7	1

a = 6

50% chiral	c 2	a 2
Ma_6b	2	2

CN-7 Five-pointed scallop seashell (C_{2v})

FPSS-7 (C_{2v}) Five-pointed scallop seashell

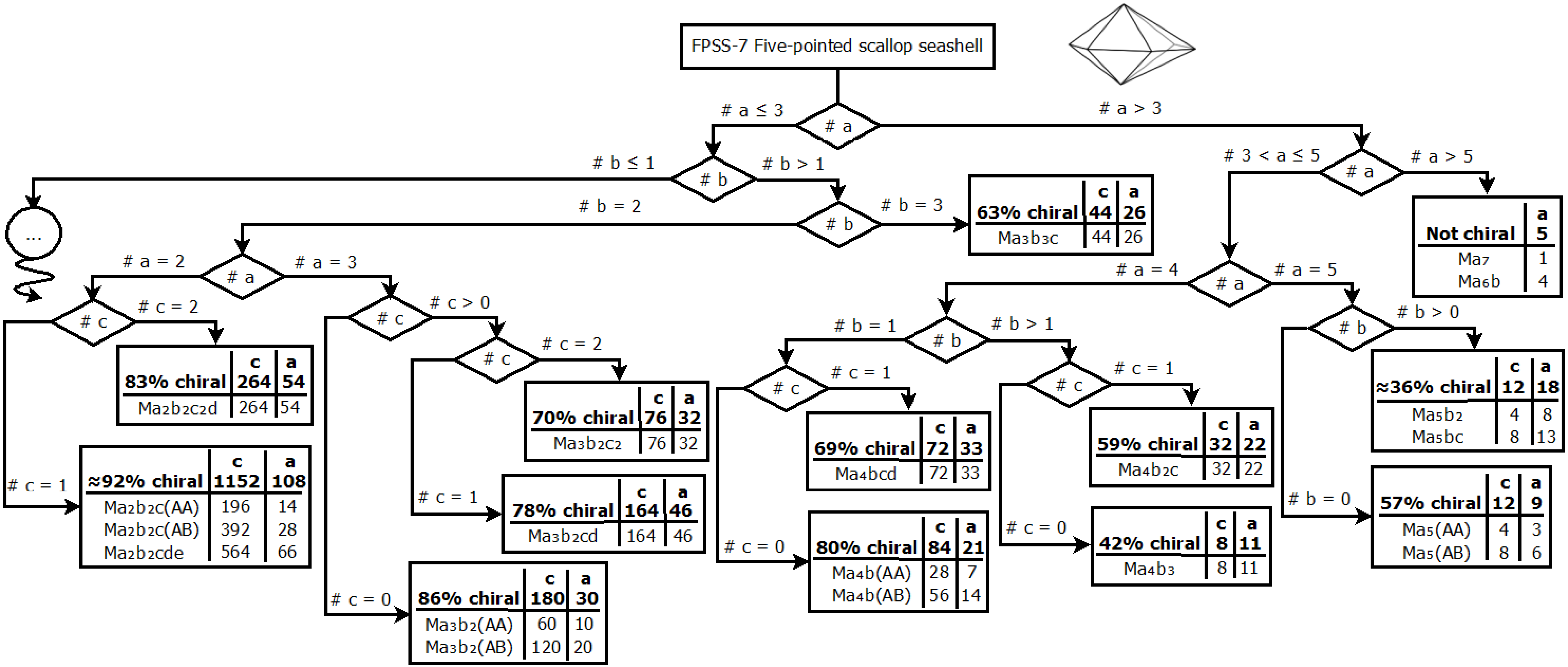

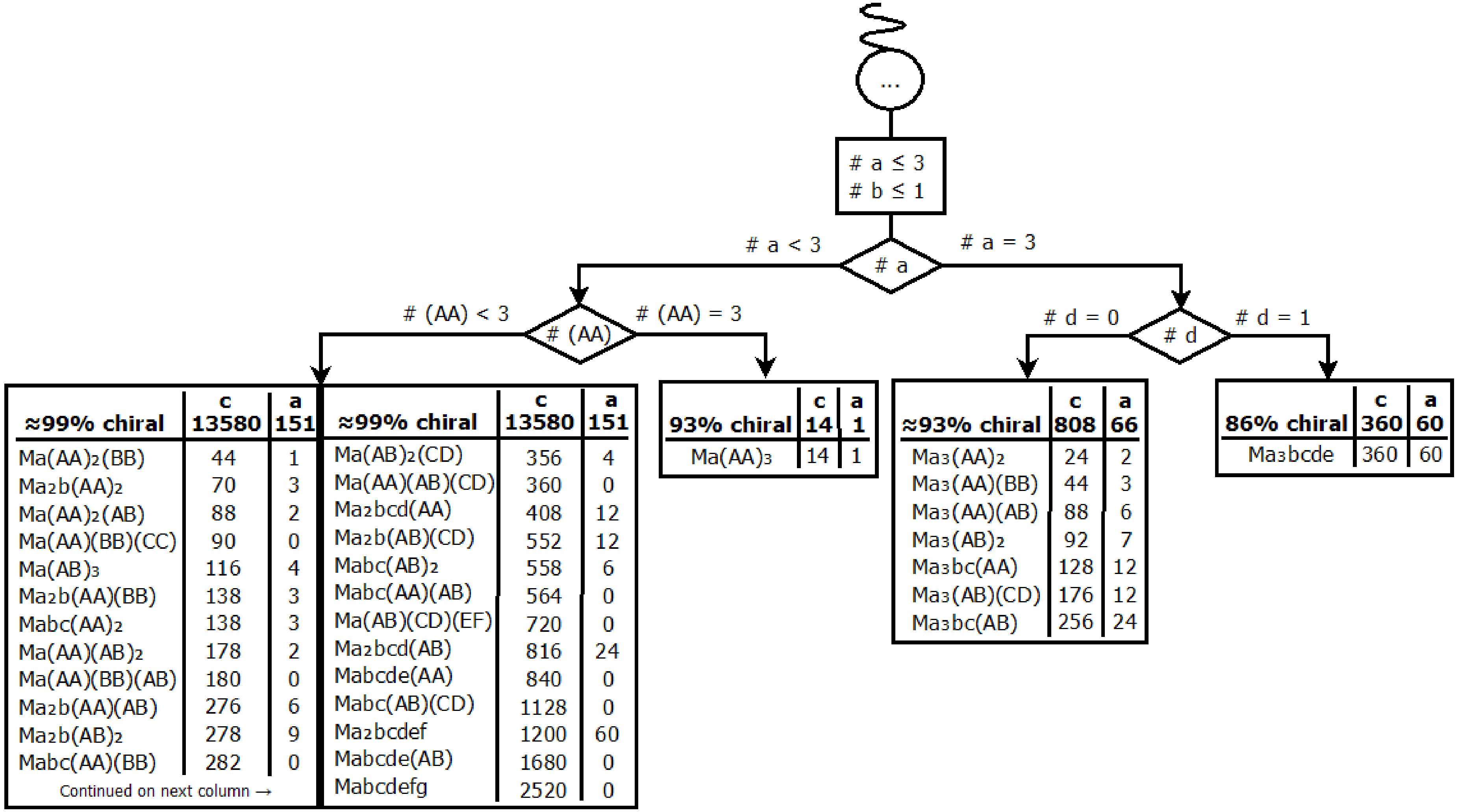

≈99% chiral	c 13580	a 151
$Ma(AA)_2(BB)$	44	1
$Ma_2b(AA)_2$	70	3
$Ma(AA)_2(AB)$	88	2
$Ma(AA)(BB)(CC)$	90	0
$Ma(AB)_3$	116	4
$Ma_2b(AA)(BB)$	138	3
$Mabc(AA)_2$	138	3
$Ma(AA)(AB)_2$	178	2
$Ma(AA)(BB)(AB)$	180	0
$Ma_2b(AA)(AB)$	276	6
$Ma_2b(AB)_2$	278	9
$Mabc(AA)(BB)$	282	0

Continued on next column →

≈99% chiral	c 13580	a 151
$Ma(AB)_2(CD)$	356	4
$Ma(AA)(AB)(CD)$	360	0
$Ma_2bcd(AA)$	408	12
$Ma_2b(AB)(CD)$	552	12
$Mabc(AB)_2$	558	6
$Mabc(AA)(AB)$	564	0
$Ma(AB)(CD)(EF)$	720	0
$Ma_2bcd(AB)$	816	24
$Mabcde(AA)$	840	0
$Mabc(AB)(CD)$	1128	0
Ma_2bcdef	1200	60
$Mabcde(AB)$	1680	0
$Mabcdefg$	2520	0

93% chiral	c 14	a 1
$Ma(AA)_3$	14	1

≈93% chiral	c 808	a 66
$Ma_3(AA)_2$	24	2
$Ma_3(AA)(BB)$	44	3
$Ma_3(AA)(AB)$	88	6
$Ma_3(AB)_2$	92	7
$Ma_3bc(AA)$	128	12
$Ma_3(AB)(CD)$	176	12
$Ma_3bc(AB)$	256	24

86% chiral	c 360	a 60
Ma_3bcde	360	60

HECU-7 (C_{2v}) Hemicube

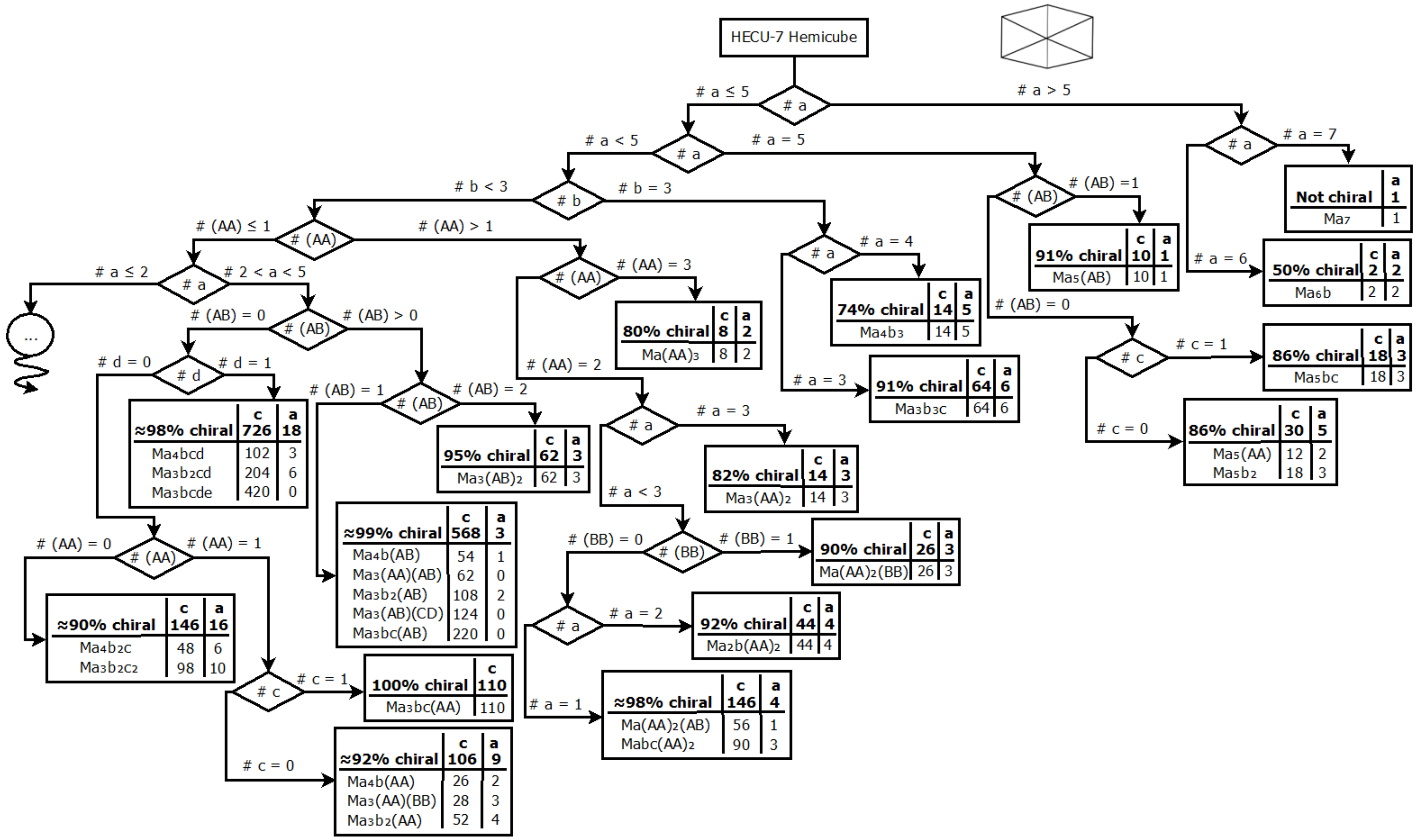

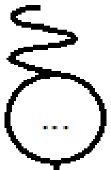

a ≤ 2
b < 3
(AA) ≤ 1

≈99% chiral	c 11934	a 48
Ma(AA)(BB)(CC)	54	3
Ma(AB)$_3$	74	2
Ma$_2$b(AA)(BB)	90	3
Ma(AA)(AB)$_2$	112	3
Ma(AA)(BB)(AB)	114	0
Ma$_2$b$_2$c(AA)	162	4
Ma$_2$b(AB)$_2$	184	5
Ma$_2$b(AA)(AB)	186	0
Mabc(AA)(BB)	186	0
Ma(AB)$_2$(CD)	226	2
Ma(AA)(AB)(CD)	228	0
Ma$_2$b$_2$c$_2$d	306	12

Continued on next column →

≈99% chiral	c 11934	a 48
Ma$_2$b$_2$c(AB)	328	2
Ma$_2$bcd(AA)	330	0
Mabc(AB)$_2$	366	6
Ma$_2$b(AB)(CD)	372	0
Mabc(AA)(AB)	372	0
Ma(AB)(CD)(EF)	456	0
Ma$_2$b$_2$cde	624	6
Ma$_2$bcd(AB)	660	0
Mabcde(AA)	660	0
Mabc(AB)(CD)	744	0
Ma$_2$bcdef	1260	0
Mabcde(AB)	1320	0
Mabcdefg	2520	0

CN-7 Digonal pseudo-anticupola (C_s)

DPAC-7 (C_s) Digonal pseudo-anticupola

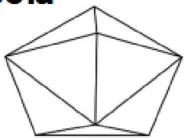

DPAC-7 Digonal pseudo-anticupola

a = 7

Not chiral	a
	1
Ma_7	1

a < 7 → # a: # 5 ≤ a < 7 → # a

a = 6

57% chiral	c	a
	4	3
Ma_6b	4	3

a = 5 → # b

b = 2

76% chiral	c	a
	16	5
Ma_5b_2	16	5

b < 2 → # (AA)

(AA) = 1

77% chiral	c	a
	10	3
$Ma_5(AA)$	10	3

(AA) = 0

≈86% chiral	c	a
	58	10
$Ma_5(AB)$	22	4
Ma_5bc	36	6

a < 5 → # b: # b = 3 → # a

a = 4

80% chiral	c	a
	28	7
Ma_4b_3	28	7

a = 3

91% chiral	c	a
	128	12
Ma_3b_3c	128	12

b < 3 → # a: # 3 ≤ a < 5 → # (AB)

(AB) > 0

≈99% chiral	c	a
	1548	18
$Ma_4b(AB)$	126	4
$Ma_3(AA)(AB)$	160	4
$Ma_3(AB)_2$	162	2
$Ma_3b_2(AB)$	252	8
$Ma_3(AB)(CD)$	328	0
$Ma_3bc(AB)$	520	0

(AB) = 0 → # d

d = 1

≈98% chiral	c	a
	1452	18
Ma_4bcd	204	6
Ma_3b_2cd	408	12
Ma_3bcde	840	0

d = 0 → # a

a = 4

≈92% chiral	c	a
	156	14
$Ma_4b(AA)$	60	5
Ma_4b_2c	96	9

a = 3

≈95% chiral	c	a
	688	35
$Ma_3(AA)_2$	38	3
$Ma_3(AA)(BB)$	78	4
$Ma_3b_2(AA)$	122	8
$Ma_3b_2c_2$	196	14
$Ma_3bc(AA)$	254	6

a < 3 → # (AA)

(AA) = 3

92% chiral	c	a
	24	2
$Ma(AA)_3$	24	2

(AA) < 3

≈99% chiral	c	a
	28252	117
$Ma(AA)_2(BB)$	76	2
$Ma_2b(AA)_2$	118	5
$Ma(AA)_2(AB)$	152	4
$Ma(AA)(BB)(CC)$	156	0
$Ma(AB)_3$	200	8
$Mabc(AA)_2$	240	6
$Ma_2b(AA)(BB)$	242	4
$Ma(AA)(AB)_2$	308	4
$Ma(AA)(BB)(AB)$	312	0
$Ma_2b_2c(AA)$	380	10
$Ma_2b(AB)_2$	486	6
$Ma_2b(AA)(AB)$	488	4
$Mabc(AA)(BB)$	492	0
$Ma_2b_2c_2d$	612	18

Continued on next column →

≈99% chiral	c	a
	28252	117
$Ma(AB)_2(CD)$	616	8
$Ma(AA)(AB)(CD)$	624	0
$Ma_2b_2c(AB)$	772	8
$Ma_2bcd(AA)$	774	6
$Mabc(AB)_2$	972	12
$Ma_2b(AB)(CD)$	984	0
$Mabc(AA)(AB)$	984	0
$Ma(AB)(CD)(EF)$	1248	0
Ma_2b_2cde	1248	12
$Ma_2bcd(AB)$	1560	0
$Mabcde(AA)$	1560	0
$Mabc(AB)(CD)$	1968	0
Ma_2bcdef	2520	0
$Mabcde(AB)$	3120	0
$Mabcdefg$	5040	0

CN-7 Tetragon-substituted pentagonal pyramid (C_s)

TSPPY-7 (C_s) Tetragon-substituted pentagonal pyramid

TSSPY-7 Tetragon-substituted pentagonal pyramid

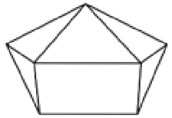

a = 7

Not chiral	a 1
Ma_7	1

a < 7; # 5 ≤ a < 7; # c = 1

100% chiral	c 42
Ma_5bc	42

a < 7; # 5 ≤ a < 7; # c = 0; # (AB) = 1

100% chiral	c 24
$Ma_5(AB)$	24

a < 7; # 5 ≤ a < 7; # c = 0; # (AB) = 0

≈85% chiral	c 34	a 6
Ma_6b	6	1
$Ma_5(AA)$	10	2
Ma_5b_2	18	3

a < 7; # a < 5; # (AA) > 1; # a = 3

89% chiral	c 32	a 4
$Ma_3(AA)_2$	32	4

a < 7; # a < 5; # (AA) > 1; # a < 3; # (AA) = 3

91% chiral	c 20	a 2
$Ma(AA)_3$	20	2

a < 7; # a < 5; # (AA) > 1; # a < 3; # (AA) = 2

≈98% chiral	c 516	a 6
$Ma(AA)_2(BB)$	64	2
$Ma_2b(AA)_2$	104	4
$Ma(AA)_2(AB)$	132	0
$Mabc(AA)_2$	216	0

a < 7; # a < 5; # (AA) ≤ 1; # b = 3; # a = 4

91% chiral	c 32	a 3
Ma_4b_3	32	3

a < 7; # a < 5; # (AA) ≤ 1; # b = 3; # a = 3

100% chiral	c 140
Ma_3b_3c	140

a < 7; # a < 5; # (AA) ≤ 1; # b < 3

≈99% chiral	c 29438	a 45
$Ma_4b(AA)$	58	2
$Ma_3(AA)(BB)$	70	2
Ma_4b_2c	102	3
$Ma_3b_2(AA)$	116	4
$Ma_4b(AB)$	120	0
$Ma(AA)(BB)(CC)$	132	0
$Ma_3(AB)_2$	138	6
$Ma_3(AA)(AB)$	144	0
$Ma(AB)_3$	176	0
$Ma_3b_2c_2$	204	6
Ma_4bcd	210	0
$Ma_2b(AA)(BB)$	214	2
$Ma_3b_2(AB)$	240	0
$Ma_3bc(AA)$	240	0
$Ma(AA)(AB)_2$	260	4
$Ma(AA)(BB)(AB)$	264	0
$Ma_3(AB)(CD)$	288	0
$Ma_2b_2c(AA)$	356	4
Ma_3b_2cd	420	0
$Ma_2b(AB)_2$	426	6

Continued on next column →

≈99% chiral	c 29438	a 45
$Ma_2b(AA)(AB)$	432	0
$Mabc(AA)(BB)$	432	0
$Ma_3bc(AB)$	480	0
$Ma(AA)(AB)(CD)$	528	0
$Ma(AB)_2(CD)$	528	0
$Ma_2b_2c_2d$	624	6
$Ma_2b_2c(AB)$	720	0
$Ma_2bcd(AA)$	720	0
Ma_3bcde	840	0
$Ma_2b(AB)(CD)$	864	0
$Mabc(AA)(AB)$	864	0
$Mabc(AB)_2$	864	0
$Ma(AB)(CD)(EF)$	1056	0
Ma_2b_2cde	1260	0
$Ma_2bcd(AB)$	1440	0
$Mabcde(AA)$	1440	0
$Mabc(AB)(CD)$	1728	0
Ma_2bcdef	2520	0
$Mabcde(AB)$	2880	0
$Mabcdefg$	5040	0

CN-7 Hemiobelisk (C_s)

HEOB-7 (C_s) Hemiobelisk

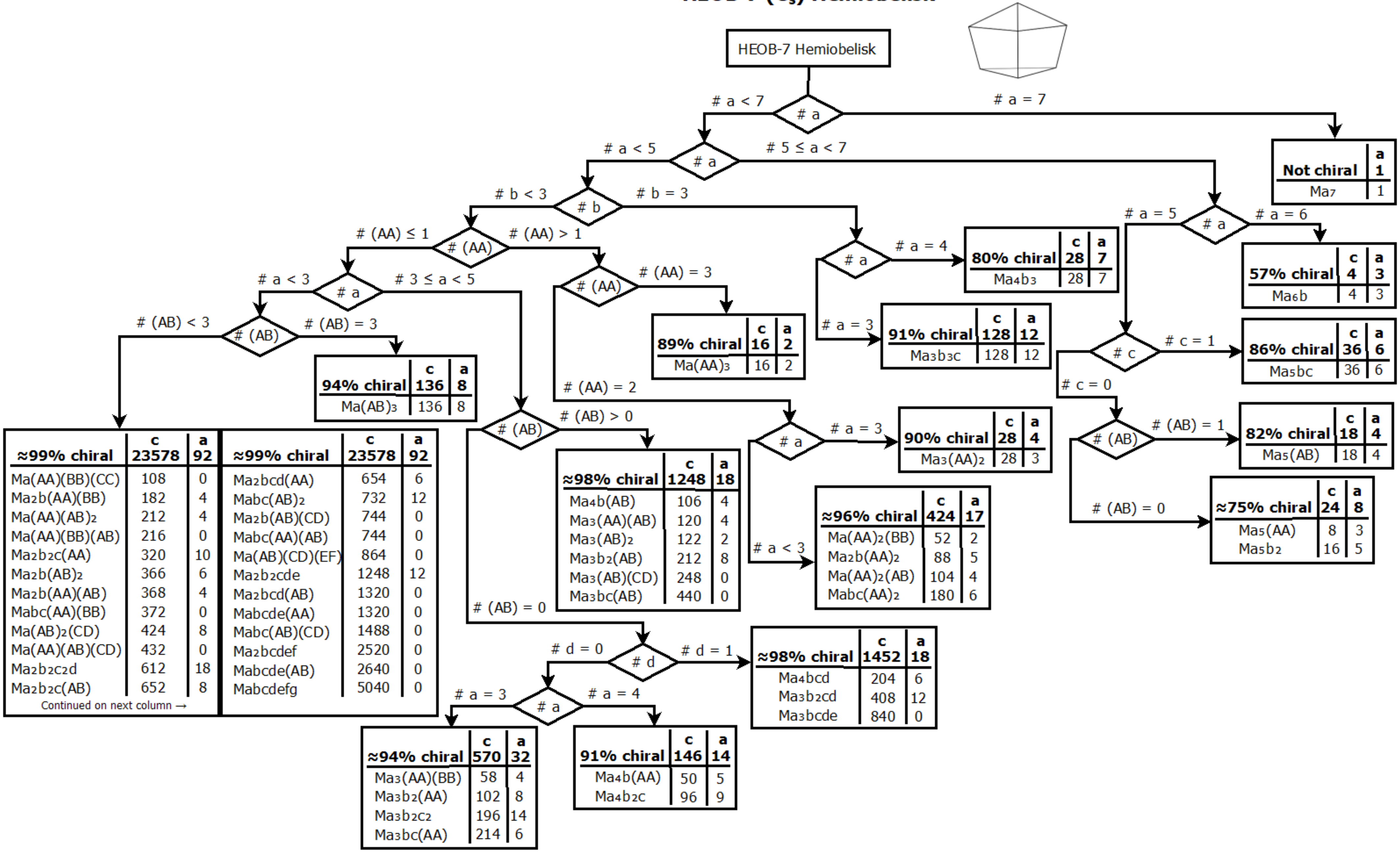

Ideal geometries for the various shapes and coordination numbers.

Tables S12 and S13 present the Cartesian coordinates for the optimized configurations of all 6- and 7-vertex coordination polyhedra, respectively, as calculated by our algorithm. For each coordination number, we included the coordinates of the corresponding polygonal geometry. inscribed in a unit sphere, and have been optimized to achieve maximum symmetry and minimum repulsion.

Table S12. Cartesian coordinates of the maximum symmetry minimum repulsion inscribed forms of the 6-vertex polyhedral shapes studied in this work. The tetragonal antiwedge (TAW-6) has two enantiomorphic representations, distinguished by the prefixes Δ- and Λ-. The hexagon planar geometry (HP-6) is also depicted.

Positions	OC-6			HP-6			TPR-6			CSPY-6			Δ-TAW-6-6		
	x	y	z	x	y	z	x	y	z	x	y	z	x	y	z
M	0.0000	0.0000	0.0000	0.0000	0.0000	0.0000	0.0000	0.0000	0.0000	0.0000	0.0000	0.0000	0.0000	0.0000	0.0000
1	0.0000	0.0000	0.1000	1.0000	0.0000	0.0000	-0.6784	-0.3917	0.6216	0.6848	-0.3953	0.6122	0.6744	-0.3928	-0.6252
2	0.1000	0.0000	0.0000	0.5000	0.8660	0.0000	-0.6784	-0.3916	-0.6216	0.6709	-0.3874	-0.6324	-0.6727	-0.3928	-0.6271
3	0.0000	0.1000	0.0000	-0.5000	0.8660	0.0000	0.6784	-0.3916	0.6216	0.6835	-0.3757	0.6259	-0.6791	-0.3877	0.6233
4	-0.1000	0.0000	0.0000	-1.0000	0.0000	0.0000	0.6784	-0.3917	-0.6216	0.6734	-0.4091	-0.6158	0.6773	-0.3877	0.6253
5	0.0000	-0.1000	0.0000	-0.5000	-0.8660	0.0000	0.0000	0.7833	0.6217	0.0164	0.7798	0.6258	0.0038	0.7790	0.6270
6	0.0000	0.0000	-0.1000	0.5000	-0.8660	0.0000	0.0000	0.7833	-0.6217	0.0176	0.7877	-0.6158	-0.0037	0.7820	-0.6233

Positions	Λ-TAW-6			PPY-6			DAC-6			STBPY-6		
	x	y	Z	x	y	z	x	y	Z	x	y	z
M	0.0000	0.0000	0.0000	0.0000	0.0000	0.0000	0.0000	0.0000	0.0000	0.0000	0.0000	0.0000
1	0.6744	-0.3928	0.6252	-0.5047	0.2900	0.8131	0.1433	0.0000	0.9897	0.2869	-0.2869	0.9140
2	-0.6727	-0.3928	0.6271	0.8131	0.2900	-0.5047	0.9897	0.0000	0.1433	0.9300	0.3675	0.0000
3	-0.6791	-0.3877	-0.6233	0.6051	0.5173	0.6051	-0.2804	0.9180	-0.2804	-0.1473	0.9891	0.0000
4	0.6773	-0.3877	-0.6253	-0.9827	-0.0777	-0.1682	-0.9327	0.0000	0.3606	-0.9891	0.1473	0.0000
5	0.0038	0.7790	-0.6270	0.2374	-0.9420	0.2374	-0.2804	-0.9180	-0.2804	-0.3675	-0.9300	0.0000
6	-0.0037	0.7820	0.6233	-0.1682	-0.0777	-0.9827	0.3606	0.0000	-0.9327	0.2869	-0.2869	-0.9140

Table S13. Cartesian coordinates of the maximum symmetry minimum repulsion inscribed forms of the shapes of coordination number 7 studied in this work. Chiral polyhedra have two enantiomorphic representations, distinguished by the prefixes Δ- and Λ-. The heptagon planar geometry (HP-7) was also depicted.

Positions	1 (HP-7)			2 (PBPY-7)			3 (HPY-7)			4 (ETPY-7)			5 (COC-7)		
	x	y	z	x	y	z	x	y	z	x	y	z	x	y	z
M	0.0000	0.0000	0.0000	0.0000	0.0000	0.0000	0.0000	0.0000	0.0000	0.0000	0.0000	0.0000	0.0000	0.0000	0.0000
1	1.0000	0.0000	0.0000	0.6271	-0.3621	0.6896	0.0000	0.0000	1.0000	0.7703	-0.4467	0.4551	0.6557	-0.3769	0.6542
2	0.6235	0.7818	0.0000	-0.7771	-0.2301	0.5859	0.8539	0.4930	-0.1667	-0.3043	-0.7563	0.5791	-0.5853	-0.6192	0.5234
3	-0.2225	0.9749	0.0000	-0.2910	-0.9302	-0.2238	0.8539	-0.4930	-0.1667	0.6503	-0.3740	-0.6612	0.1332	-0.8569	-0.4979
4	-0.9010	0.4339	0.0000	0.6601	0.7171	-0.2238	0.0000	-0.9860	-0.1667	0.5022	0.6384	0.5833	0.8076	0.3161	-0.4979
5	-0.9009	-0.4339	0.0000	0.5972	-0.3448	-0.7242	-0.8539	-0.4930	-0.1667	-0.9899	-0.1417	-0.0026	0.2406	0.8174	0.5234
6	-0.2225	-0.9749	0.0000	-0.1893	0.7880	0.5859	-0.8539	0.4930	-0.1667	-0.2574	0.1517	-0.9543	-0.8524	0.4901	0.1824
7	0.6235	-0.7818	0.0000	-0.6271	0.3621	-0.6896	0.0000	0.9860	-0.1667	-0.3711	0.9286	0.0006	-0.3993	0.2296	-0.8876

Positions	6			7 (DTT-7)			8 (TT-7)			9			10		
	x	y	z	x	y	z	x	y	z	x	y	z	x	y	Z
M	0.0000	0.0000	0.0000	0.0000	0.0000	0.0000	0.0000	0.0000	0.0000	0.0000	0.0000	0.0000	0.0000	0.0000	0.0000
1	0.7785	0.0215	0.6273	0.8094	0.0034	0.5873	0.6491	-0.3303	0.6853	0.8009	-0.0810	0.5933	0.6680	-0.4684	0.5782
2	-0.0928	0.8776	0.4703	0.0613	0.8559	0.5134	-0.6506	-0.2207	0.7267	-0.2612	-0.8075	0.5288	-0.4521	-0.7054	0.5459
3	-0.9375	-0.0045	0.3480	-0.9083	-0.0032	0.4182	0.5758	-0.7295	-0.3693	-0.2413	-0.7581	-0.6058	-0.5968	-0.5542	-0.5802
4	-0.0720	-0.8607	0.5041	0.0678	-0.8548	0.5145	0.7121	0.6786	0.1803	0.6960	-0.1119	-0.7093	0.6614	-0.2623	-0.7027
5	0.4766	-0.6717	-0.5671	0.2653	-0.7545	-0.6003	-0.3472	0.3700	-0.8617	-0.0292	0.7460	-0.6653	0.2311	0.9187	-0.3204
6	-0.6134	-0.0227	-0.7894	-0.5550	-0.0026	-0.8318	-0.3927	0.9105	0.1295	-0.9044	0.1210	0.4092	-0.8215	0.4975	-0.2785
7	0.4606	0.6604	-0.5930	0.2596	0.7558	-0.6012	-0.5465	-0.6786	-0.4908	-0.0607	0.8915	0.4490	0.3099	0.5742	0.7578

Positions	11 (CTPR-7)			12 (*Tr*BCSPY-7)			13 (FPSS-7)			14 (SPBPY-7)			15 (HECU-7)		
	x	y	z	x	y	Z	x	y	Z	x	y	z	x	y	z
M	0.0000	0.0000	0.0000	0.0000	0.0000	0.0000	0.0000	0.0000	0.0000	0.0000	0.0000	0.0000	0.0000	0.0000	0.0000
1	0.6792	-0.3640	0.6374	0.8809	0.0998	0.4627	0.8586	0.1422	0.4926	0.6658	-0.6514	0.364	0.8225	0.0001	0.5687
2	-0.6172	-0.4613	0.6374	0.0998	0.8808	0.4628	0.0056	0.9721	0.2346	-0.3006	-0.8565	0.4197	0.1650	-0.8501	0.5002
3	0.0590	-0.9338	-0.3529	-0.8810	-0.1000	0.4624	-0.8635	0.1551	0.4800	-0.3021	-0.0555	-0.9517	-0.2183	-0.8500	-0.4794
4	0.1515	0.8210	0.5505	-0.0997	-0.8809	0.4627	-0.0105	-0.6748	0.7380	0.9803	0.1914	-0.048	0.2149	-0.0001	-0.9766
5	0.8277	0.3485	-0.4398	0.6400	-0.6399	-0.4254	0.0104	0.6674	-0.7446	0.3131	0.8531	-0.4173	-0.2185	0.8499	-0.4795
6	-0.8578	0.5098	-0.0656	0.0000	0.0000	-1.0000	0.0048	-0.2923	-0.9563	-0.3788	0.7217	0.5794	-0.9304	-0.0001	0.3666
7	-0.2424	0.0798	-0.9669	-0.6399	0.6401	-0.4253	-0.0055	-0.9697	-0.2442	-0.9777	-0.2029	0.054	0.1647	0.8502	0.5001

Table S13 (continuation).

Positions	16			17			18			19 (*Cis*BCSPY-7)			20-Δ		
	x	y	z	x	y	Z	x	y	z	x	y	z	x	y	Z
M	0.0000	0.0000	0.0000	0.0000	0.0000	0.0000	0.0000	0.0000	0.0000	0.0000	0.0000	0.0000	0.0000	0.0000	0.0000
1	0.8217	0.0001	0.5699	0.8245	-0.0042	0.5659	0.8247	0.0091	0.5655	0.8674	-0.0033	0.4976	0.8237	0.0063	0.5670
2	0.1603	-0.8597	0.4851	0.1635	-0.8570	0.4887	0.1607	-0.8565	0.4906	0.2891	-0.8673	-0.4052	0.1563	-0.8661	0.4749
3	-0.2126	-0.8598	-0.4643	-0.2226	-0.8527	-0.4726	-0.2004	-0.8592	-0.4708	-0.0603	-0.0055	-0.9982	-0.2085	-0.8603	-0.4652
4	0.2140	0.0000	-0.9768	0.2013	-0.0008	-0.9795	0.2214	0.0024	-0.9752	0.3016	0.8582	-0.4155	0.2138	0.0009	-0.9769
5	-0.2128	0.8597	-0.4644	-0.2141	0.8550	-0.4724	-0.2193	0.8545	-0.4708	-0.2516	-0.8542	0.4551	-0.2036	0.851	-0.4841
6	-0.9307	-0.0001	0.3657	-0.9246	0.0045	0.3809	-0.9289	-0.0102	0.3702	-0.2394	0.8631	0.4446	-0.9234	0.0142	0.3835
7	0.1602	0.8598	0.4849	0.1720	0.8551	0.4890	0.1417	0.8598	0.4905	-0.9068	0.0090	0.4215	0.1418	0.8539	0.5007

Positions	20-Λ			21 (DPAC-7)			22			23 (TSPPY-7)			24-Δ		
	x	y	z	x	y	Z	x	y	z	x	y	z	x	y	z
M	0.0000	0.0000	0.0000	0.0000	0.0000	0.0000	0.0000	0.0000	0.0000	0.0000	0.0000	0.0000	0.0000	0.0000	0.0000
1	0.8237	0.0063	-0.5670	0.9413	0.3053	-0.1443	0.2306	-0.6065	-0.7609	0.9214	0.2689	0.2807	0.9163	0.2765	0.2898
2	0.1563	-0.8661	-0.4749	0.1074	0.9811	0.1608	-0.5804	0.2055	-0.7880	-0.0019	0.9373	0.3485	0.0055	0.9347	0.3554
3	-0.2085	-0.8603	0.4652	0.111	0.0374	-0.9931	-0.3279	0.9439	-0.0401	-0.8766	0.2851	0.3876	-0.8702	0.2962	0.3938
4	0.2138	0.0009	0.9769	0.6627	-0.732	0.1583	0.9441	-0.3297	0.0024	-0.5531	-0.7570	0.3480	-0.5646	-0.7533	0.3374
5	-0.2036	0.851	0.4841	-0.3094	-0.7076	0.6353	-0.7032	-0.6978	0.1360	0.5867	-0.7597	0.2803	0.5896	-0.7614	0.2695
6	-0.9234	0.0142	-0.3835	-0.8478	-0.2742	-0.4538	-0.1770	-0.1443	0.9736	0.1373	0.5521	-0.8224	0.1211	0.5475	-0.8280
7	0.1418	0.8539	-0.5007	-0.6652	0.3899	0.6368	0.6138	0.6290	0.4770	-0.2137	-0.5267	-0.8227	-0.1977	-0.5403	-0.8179

Positions	24-Λ			25 (HEOB-7)			26 (CPPY-7)			27-Δ			27-Λ		
	x	y	z	x	y	z	x	y	z	x	y	z	x	y	z
M	0.0000	0.0000	0.0000	0.0000	0.0000	0.0000	0.0000	0.0000	0.0000	0.0000	0.0000	0.0000	0.0000	0.0000	0.0000
1	0.9163	0.2765	-0.2898	0.9503	0.3086	-0.0414	0.9508	0.3082	-0.0304	0.9513	0.3077	-0.0177	0.9513	0.3077	0.0177
2	0.0055	0.9347	-0.3554	0.1777	0.9700	0.1659	0.1808	0.9705	0.1592	0.1975	0.9641	0.1775	0.1975	0.9641	-0.1775
3	-0.8702	0.2962	-0.3938	0.0340	0.0115	-0.9994	0.0444	0.0158	-0.9989	-0.0042	-0.0076	-1.0000	-0.0042	-0.0076	1.0000
4	-0.5646	-0.7533	-0.3374	0.7134	-0.6818	0.1622	0.7159	-0.6803	0.1568	0.7367	-0.6601	0.1466	0.7367	-0.6601	-0.1466
5	0.5896	-0.7614	-0.2695	-0.2832	-0.8013	0.5269	-0.2824	-0.8124	0.5102	-0.3014	-0.7922	0.5306	-0.3014	-0.7922	-0.5306
6	0.1211	0.5475	0.8280	-0.8936	-0.2893	-0.3430	-0.9048	-0.2929	-0.3091	-0.8826	-0.2977	-0.3639	-0.8826	-0.2977	0.3639
7	-0.1977	-0.5403	0.8179	-0.6985	0.4823	0.5287	-0.7048	0.4909	0.5121	-0.6974	0.4859	0.5268	-0.6974	0.4859	-0.5268

Table S13 (continuation).

Positions	28-Δ			28-Λ			29-Δ			29-Λ			30-Δ		
	x	y	z	x	y	Z	x	y	z	x	y	Z	x	y	z
M	0.0000	0.0000	0.0000	0.0000	0.0000	0.0000	0.0000	0.0000	0.0000	0.0000	0.0000	0.0000	0.0000	0.0000	0.0000
1	0.9482	0.3159	-0.0331	0.8245	-0.0042	0.5659	0.9491	0.3133	-0.0324	0.9491	0.3133	0.0324	0.9530	0.3014	0.0310
2	0.1715	0.9674	0.1864	0.1635	-0.8570	0.4887	0.1648	0.9663	0.1978	0.1648	0.9663	-0.1978	0.1734	0.9722	-0.1576
3	0.0631	-0.0199	-0.9978	-0.2226	-0.8527	-0.4726	0.049	-0.0253	-0.9985	0.049	-0.0253	0.9985	0.0380	0.0171	0.9991
4	0.7359	-0.6679	0.1109	0.2013	-0.0008	-0.9795	0.7191	-0.6836	0.1248	0.7191	-0.6836	-0.1248	0.7121	-0.6846	-0.1553
5	-0.3249	-0.7856	0.5265	-0.2141	0.8550	-0.4724	-0.2681	-0.8011	0.5351	-0.2681	-0.8011	-0.5351	-0.2730	-0.8035	-0.5290
6	-0.899	-0.3005	-0.3188	-0.9246	0.0045	0.3809	-0.906	-0.2664	-0.3288	-0.906	-0.2664	0.3288	-0.8991	-0.2844	0.3328
7	-0.6949	0.4905	0.5259	0.1720	0.8551	0.4890	-0.7078	0.4969	0.5020	-0.7078	0.4969	-0.5020	-0.7044	0.4819	-0.5211

Positions	30-Λ			31 (Δ-THTB-7)			31 (Λ-THTB-7)			32-Δ			32- Λ		
	x	y	Z	x	y	z	x	y	z	x	y	z	x	y	z
M	0.0000	0.0000	0.0000	0.0000	0.0000	0.0000	0.0000	0.0000	0.0000	0.0000	0.0000	0.0000	0.0000	0.0000	0.0000
1	0.8237	0.0063	0.5670	0.7870	0.4200	-0.4519	0.7870	0.4200	0.4519	0.8249	0.2518	-0.5062	0.8249	0.2518	0.5062
2	0.1563	-0.8661	0.4749	0.5118	-0.6608	-0.5490	0.5118	-0.6608	0.5490	-0.1092	0.8496	-0.5161	-0.1092	0.8496	0.5161
3	-0.2085	-0.8603	-0.4652	0.1096	-0.6734	0.7311	0.1096	-0.6734	-0.7311	-0.9130	0.1523	-0.3785	-0.9130	0.1523	0.3785
4	0.2138	0.0009	-0.9769	0.3848	0.4074	0.8282	0.3848	0.4074	-0.8282	-0.5195	-0.8039	-0.2894	-0.5195	-0.8039	0.2894
5	-0.2036	0.851	-0.4841	-0.1558	0.9445	-0.2891	-0.1558	0.9445	0.2891	-0.3215	0.5272	0.7866	-0.3215	0.5272	-0.7866
6	-0.9234	0.0142	0.3835	-0.9218	0.2605	-0.2870	-0.9218	0.2605	0.2870	0.6047	-0.0827	0.7921	0.6047	-0.0827	-0.7921
7	0.1418	0.8539	0.5007	-0.7156	-0.6982	0.0178	-0.7156	-0.6982	-0.0178	0.4335	-0.8942	0.1114	0.4335	-0.8942	-0.1114

Positions	33 (Δ-SHEAPR-7)			33 (Λ -SHEAPR-7)			34-Δ			34-Λ			35-Δ		
	x	y	Z	x	y	z	x	y	z	x	y	z	x	y	z
M	0.0000	0.0000	0.0000	0.0000	0.0000	0.0000	0.0000	0.0000	0.0000	0.0000	0.0000	0.0000	0.0000	0.0000	0.0000
1	0.9242	-0.2796	-0.2602	0.9242	-0.2796	0.2602	0.9935	-0.0283	0.1102	0.9935	-0.0283	-0.1102	0.0290	0.9669	-0.2536
2	-0.3546	-0.6436	0.6782	-0.3546	-0.6436	-0.6782	-0.3315	0.2371	0.9132	-0.3315	0.2371	-0.9132	0.8342	0.4523	0.3155
3	-0.2395	0.4463	0.8623	-0.2395	0.4463	-0.8623	0.4278	-0.8725	-0.2362	0.4278	-0.8725	0.2362	0.1989	-0.7720	-0.6037
4	0.6896	0.7019	0.1782	0.6896	0.7019	-0.1782	-0.0536	0.6048	-0.7945	-0.0536	0.6048	0.7945	-0.4325	0.1097	-0.8949
5	-0.2447	0.8249	-0.5095	-0.2447	0.8249	0.5095	-0.8374	-0.0834	-0.5402	-0.8374	-0.0834	0.5402	-0.7469	0.4851	0.4547
6	-0.8149	-0.1273	-0.5654	-0.8149	-0.1273	0.5654	0.3921	0.8881	0.2400	0.3921	0.8881	-0.2400	-0.5461	-0.6280	0.5545
7	0.0399	-0.9226	-0.3836	0.0399	-0.9226	0.3836	-0.5908	-0.7459	0.3075	-0.5908	-0.7459	-0.3075	0.6635	-0.6140	0.4275

Table S13 (continuation).

Positions	35- Λ		
	x	y	Z
M	0.0000	0.0000	0.0000
1	0.0290	0.9669	0.2536
2	0.8342	0.4523	-0.3155
3	0.1989	-0.7720	0.6037
4	-0.4325	0.1097	0.8949
5	-0.7469	0.4851	-0.4547
6	-0.5461	-0.6280	-0.5545
7	0.6635	-0.6140	-0.4275

Algebraic Counting of the Number of Stereoisomers for the Selected Coordination Polyhedra

The enumeration of possible stereoisomers for a generic formula composition with monodentate ligands within a specific coordination polyhedron can be accomplished using George Pólya's enumeration theorem, as specifically adapted by Krivoshei and Vvedenskii for applications in coordination chemistry. The application of Pólya's formalism to stereoisomer enumeration relies on fundamental concepts from symmetry group theory. A comprehensive background of the underlying algebraic equations and their theoretical foundations can be found in the Supporting Information of our previous work (DOI: 10.1021/acs.inorgchem.8b01133).

In the following, we present the corresponding cycle indices for all 6- and 7-vertex polyhedral shapes that were not considered in our previous work, including the new ones first introduced here. For polyhedra with both first-kind (proper rotations, C_n) and second-kind symmetry operations (improper rotations, Sn, symmetry planes, σ, and center of inversion, i), as defined by IUPAC, we provide two distinct cycle indices: one encompassing all isometries of the polyhedron, and another polynomial considering exclusively first-type isometries. This separation of handedness-reversing symmetry operations from other isometries, resulting in a partial cycle index devoid of handedness-preserving operations, enables the algebraic analysis of coordination chirality and facilitates determination of the total number of achiral (**a**) and chiral (**c**) stereoisomers.

Chiral polyhedra represent a special case in which the complete cycle index incorporating all symmetry operations is identical to the index containing only first-kind operations. Consequently, all enumerated stereoisomers for any given generic formula within these polyhedra are exclusively chiral.

Coordination number 6:

Digonal anticupola DAC-6

$$\mathrm{Z}(\mathrm{C_{2v}}) = \frac{1}{4}(\mathrm{x}_1^6 + \mathrm{x}_1^4\mathrm{t}_2 + \mathrm{x}_1^2\mathrm{t}_2^2 + \mathrm{x}_2^3)$$

$$\mathrm{Z}(\mathrm{C_2}) = \frac{1}{2}(\mathrm{x}_1^6 + \mathrm{x}_2^3)$$

Coordination number 7:

Elongated trigonal pyramid (ETPY-7):

$$P(\mathrm{C_3}) = \frac{x_1^7 + 2x_1x_3^2}{3}$$

$$P(\mathrm{C_{3v}}) = \frac{x_1^7 + 2x_1x_3^2 + 3x_1^3x_2^2}{6}$$

Diminished trigonal trapezohedron (DTT-7):

$$P(\mathrm{C_3}) = \frac{x_1^7 + 2x_1x_3^2}{3}$$

$$P(\mathrm{C_{3v}}) = \frac{x_1^7 + 2x_1x_3^2 + 3x_1^3x_2^2}{6}$$

Trans-bicapped square pyramid (*Tr*BCSPY-7):

$$P(\mathrm{C_2}) = \frac{x_1^7 + x_1x_2^3}{2}$$

$$P(\mathrm{C_{2v}}) = \frac{x_1^7 + 2x_1x_2^3 + x_1^3x_2^2}{4}$$

Five-pointed scallop seashell (FPS-7):

$$P(\mathrm{C_2}) = \frac{x_1^7 + x_1x_2^3}{2}$$

$$P(\mathrm{C_{2v}}) = \frac{x_1^7 + x_1x_2^3 + x_1^3x_2^2 + x_1^5x_2}{4}$$

Hemicube (HECU-7):

$$P(\mathrm{C_2}) = \frac{x_1^7 + x_1x_2^3}{2}$$

$$P(\mathrm{C_{2v}}) = \frac{x_1^7 + 2x_1x_2^3 + x_1^3x_2^2}{4}$$

Digonak pseudo-anticupola (DPAD-7):

$$P(\mathrm{C_1}) = x_1^7$$

$$P(\mathrm{C_s}) = \frac{x_1^7 + x_1^3x_2^2}{2}$$

Tetragon-substitued pentagonal pyramid (TSPPY-7):

$$P(\mathrm{C_1}) = x_1^7$$

$$P(\mathrm{C_s}) = \frac{x_1^7 + x_1 x_2^3}{2}$$

Hemiobelisk (HEOB-7):

$$P(\mathrm{C_1}) = x_1^7$$

$$P(\mathrm{C_s}) = \frac{x_1^7 + x_1^3 x_2^2}{2}$$

Tetragonal helicoid with tetragonal base(THTB)-7):

$$P(\mathrm{C_2}) = \frac{x_1^7 + x_1 x_2^3}{2}$$

Square hemiantiprism (SHEAPR-7):

$$P(\mathrm{C_2}) = \frac{x_1^7 + x_1 x_2^3}{2}$$

Occurrence of the Digonal Anticupola (DAC-6) Coordination Geometry in Reported Crystallographic Structures of Metal Complexes

In coordination chemistry, three polyhedral shapes are traditionally considered as primary geometries for six-coordinate metal complexes: the octahedron (OC-6), the trigonal prism (TPR-6), and the pentagonal pyramid (PPY-6). The hexagonal planar geometry (HP-6) has also been discussed in literature. Our research demonstrates that another polyhedral architecture must also be considered for 6-coordinate compounds: the digonal anticupola (DAC-6). The maximum symmetry minimum repulsion (MSMR) representation of this shape exhibits distinct geometric characteristics that cannot be adequately approximated by any of the aforementioned six-vertex geometries, even when accounting for typical crystallographic smearing effects.

Despite its frequent omission from discussions of molecular geometry, our analysis of the Cambridge Structural Database (CSD) revealed a significant number of six-coordinate complexes exhibiting the DAC-6 shape. Notably, the frequency of occurrence of this geometry surpasses that of the already recognized TPR-6, PPY-6, and HP-6 shapes in crystallographic structures, highlighting its prevalence in 6-coordinate coordination compounds.

In a previous study by our group (DOI: https://doi.org/10.1021/acs.inorgchem.8b01133), a comprehensive methodology for mapping the stereoisomers of metal complexes containing mono- and/or bidentate ligands within specific coordination polyhedra (CP) was developed. This protocol assigns unique codes to each possible stereoisomer, incorporating crucial structural information including the symmetry point group, chirality characteristics, symmetry number, and vertex occupation of each ligand in the given CP through permutation vectors. While this methodology was initially applied to well-established coordination polyhedra, we have now extended its application to the new polyhedral shapes introduced in this work. This extension enables precise assignment of stereoisomer codes for all empirical cases of these polyhedra, specifically when the metal complexes are mononuclear and exclusively contain mono- and/or bidentate ligands.

The digonal anticupola DAC-6 shape demonstrates frequent occurrence among coordination complexes with these ligand types. We systematically identified and cataloged the correct stereoisomer codes for all such crystallographic structures. These data are presented in Table S14. The table includes essential information such as CSD refcodes of the crystallographic structures, metal center atomic symbols, crystal space groups, root mean square deviation (RMSD) and Continuous Shape Measures (CShM) values comparing the empirical geometries to the reference DAC-6 shape.

Table S14. List of all crystallographic structures analyzed in this study that feature 6-coordinate metal complexes with mono- and/or bidentate ligands, for which the coordination environment is best described by a digonal anticupola (DAC-6) shape. The table includes the following details for each entry: the CSD reference code (refcode) of the structure, the identity of the metal center, the crystallographic space group, the root-mean-square deviation (RMSD) of the actual coordination environment from the ideal DAC-6 geometry, the CShM values for the DAC-6 shape, and a stereoisomer code describing the compound's stereochemistry. This code encodes the generic formula of the complex, its idealized point group symmetry, the presence or absence of metal-centered chirality, and a permutation vector, among other stereochemical features.

Refcode	Metal	Spatial group	RMSD	CShM	Stereoisomer code
EYODAY	Sn	P $\bar{1}$	0.0950	0.609	{[M(AA)(AB)2] DAC-6 C2 c 2 B [3 6 1 2 5 4]}<br>{[M(AA)(AB)2] DAC-6 C2 c 2 B [3 4 1 2 5 6]}
SUZWUF		P 2_1/c	0.1163	1.135	
GIZWUJ	Lu	P $\bar{1}$	0.1418	1.973	
AHAPEF	Cd	I b c a	0.1459	1.757	
MIWGOR	Y	P b c a	0.1510	2.231	
KAXMUR	Nb	C c	0.0777	0.587	{[M(AA)3] DAC-6 C2 c 2 B [1 2 3 4 5 6]}<br>{[M(AA)3] DAC-6 C2 c 2 B [1 3 5 6 4 2]}
PABZTA	Ta	P 1	0.0993	0.982	
SAWWIZ	V	P c a 2_1	0.1404	1.959	
HAMKEM	Cd	C 2/c	0.1461	2.123	
MARSEE	Mo	P $\bar{1}$	0.1518	2.287	
GIHLAL	Gd	P 2/n	0.1532	2.329	
JUWTAY	Eu	P b c a	0.1539	2.214	{[M(AA)2(BB)] DAC-6 C1 c 1 A [1 5 3 4 6 2]}<br>{[M(AA)2(BB)] DAC-6 C1 c 1 A [1 2 3 4 5 6]}
QIGYEK	Sn	P 2_1/c	0.1427	2.012	{[M(AB)2(CD)] DAC-6 C1 c 1 A [1 4 5 6 3 2]}
			0.1482	2.175	{[M(AB)2(CD)] DAC-6 C1 c 1 A [1 2 5 6 3 4]}
YIQLUG	Cd	P 2_1/n	0.1144	1.107	{[M(AB)3] DAC-6 C1 c 1 A [1 2 3 4 5 6]}<br>{[M(AB)3] DAC-6 C1 c 1 A [1 6 3 4 5 2]}
PUNFAI	Mn	P $\bar{1}$	0.1514	2.199	
EJUVIP	Dy	P 2_1/n	0.1529	2.196	
XUMPED	Mn	P 2_1/c	0.1525	2.288	{[Ma2(AA)(AB)] DAC-6 C1 c 1 A [1 5 3 4 6 2]}<br>{[Ma2(AA)(AB)] DAC-6 C1 c 1 A [1 2 3 4 6 5]}
TAQVOW	Sn	P 2_1/c	0.0656	0.398	{[Ma2(AA)2] DAC-6 C2v a 2 C [3 1 4 5 6 2]}
KUTZUU		P $\bar{1}$	0.0771	0.592	
BAKNIK01		P m m n	0.0840	0.650	
BAKNIK02		P m n 2_1	0.0855	0.688	
JUDSEG		P 2_1/n	0.0864	0.532	
AXAKAL		P c c n	0.0926	0.764	
ORONIU		P $\bar{1}$	0.0928	0.836	
UGEFAP		C 2/c	0.0943	0.863	

UZAHUZ			0.0943	0.802	{[Ma2(AA)2] DAC-6 C2v a 2 C [3 1 4 5 6 2]}
LUYSED		P c c n	0.0943	0.718	
REWCIG		P $\bar{1}$	0.0960	0.812	
LUYSED01		P c c n	0.0968	0.746	
POSYEB		P 2_1/n	0.0972	0.804	
AXAKEP		C 2/c	0.0976	0.796	
FOFDEJ01			0.0984	0.800	
ETCMSN02		P b c a	0.0985	0.835	
QEQPEH		A b a 2	0.0989	0.966	
			0.1098	1.004	
AZIPOO		C 2/c	0.0999	0.860	
KURKIS			0.1010	0.987	
HOCQAR		P 2_1/n	0.1024	0.838	
KEJGUD		C 2/c	0.1040	0.984	
VARJUU		P 2_1/c	0.1047	0.906	
CIHCIF		P n ma	0.1051	1.059	
SEKBOA		P 2_1/c	0.1067	1.071	
LUKBIC		P c a 2_1	0.1075	1.007	
LELWAD		P $\bar{1}$	0.1082	0.966	
RAQQEH		C 2/c	0.1095	1.009	
FILZUW		P 2_1/c	0.1108	0.991	
WUCQAM		P c c n	0.1107	0.745	
KUMDAX		P 2/n	0.1119	0.995	
IDOLOD01		P 2_1/n	0.1119	1.085	
XUXHIH			0.1140	1.021	
JUMWAS		P $\bar{1}$	0.1157	0.987	
NALBEH	Pb	C 2/c	0.1130	1.091	
HARLOE	Sn	P 2_1/n	0.1141	1.098	
HOCPOE		P 2_1/c	0.1164	1.069	
ABOXEV		P $\bar{1}$	0.1464	1.394	
OGURAJ			0.1314	1.555	
SIHTEI		P2/c	0.1346	1.469	
MURGUB	Ti	P $\bar{1}$	0.1353	1.466	
FIMBOT	Sn	P 2_1/n	0.1454	2.089	
JIVJED	U	P 2_1/c	0.1508	2.237	{[Ma2(AA)2] DAC-6 C1 c 1 A [1 2 3 4 5 6]}<br>{[Ma2(AA)2] DAC-6 C1 c 1 A [1 3 5 6 4 2]}
WEBYOR	Sn	C 2/c	0.0949	0.845	{[Ma2(AB)2] DAC-6 C2v a 2 C'' [3 1 4 6 5 2]}

CAYYOQ01		$P\bar{1}$	0.0308	0.074	
FOTBOF		$P 2_1/c$	0.0444	0.160	
PAHZAZ	Pb		0.0495	0.242	
IMECAE	Sn	$P 2_1/n$	0.0511	0.223	
GALTUM			0.0582	0.287	
GALSAR		$P 2_1/c$	0.0587	0.256	
TUMTEC	Pb	$P\bar{1}$	0.0676	0.450	
REXKOX	Sn		0.0718	0.471	
CUCTUP	Mo	F d d 2	0.0732	0.533	
MEMWEH	Sn	$P 2_1/c$	0.0733	0.400	
			0.0773	0.430	
			0.0976	0.746	
IXENEE		$P\bar{1}$	0.0762	0.380	
LAJSOG	Pb	$P 2_1/n$	0.0817	0.654	
JEDJIL	Sn		0.0825	0.505	
			0.0919	0.613	
AWOGID			0.0834	0.484	
IXENII		$P 2_1/a$	0.0857	0.447	
CIPCEK		$P 2_1/c$	0.0858	0.419	{[Ma2(AB)2] DAC-6 C2v a 2 C'' [4 1 3 5 6 2]}
WEYNAP01		$P 2_1/n$	0.0862	0.400	
YIBWUB			0.0872	0.658	
WEYNAP			0.0885	0.400	
FUNRUB		$P 2_1/c$	0.0891	0.684	
ELUDOE		P b c a	0.0891	0.625	
IJIXUV		$P\bar{1}$	0.0923	0.564	
			0.0979	0.651	
JAYPAB		C 2/c	0.0930	0.594	
CUCTOJ	Mo	$P 2_1/c$	0.0934	0.869	
CIPBUZ	Sn	$P\bar{1}$	0.0942	0.501	
WEVYIH		$P 2_1/n$	0.0962	0.905	
EDUPEA			0.0967	0.837	
SIYLUH		$P\bar{1}$	0.0981	0.870	
VOJKIQ		C 2/c	0.0983	0.741	
KUHPOS		$P 2_1/a$	0.0984	0.868	
XECDEO	Pb	$P 2_1/n$	0.0986	0.726	
MASOMO10	Mo	$P 2_1/c$	0.1039	1.066	
MASOMO			0.1039	1.066	

TARPOS	Sn		0.1043	0.725	
GUQPUD	W	$P\ 2_1/n$	0.1084	1.106	
			0.1405	1.931	
JEJGIP	Sn	$P\ \bar{1}$	0.1095	1.105	
QEWPOZ		$P\ 2_1/c$	0.1113	1.214	
YORLIA		C c	0.1122	0.789	
YEPSOB		$P\ \bar{1}$	0.1142	1.160	
QEWPUF			0.1147	1.264	
JAWQEE		P b c a	0.1148	1.297	
GAQNUH01		$P\ 2_1/c$	0.1164	1.342	
CIPCAG		$P\ \bar{1}$	0.1166	0.708	
ARAXIA		$P\ 2_1/c$	0.1170	0.943	
NALCUY		$P\ \bar{1}$	0.1178	1.369	
YORLIA01		C c	0.1188	0.859	
QEWPIT		C 2/c	0.1192	1.415	
XIJVIW			0.1192	0.744	
KAMGEL		$C\ m\ c\ 2_1$	0.1200	1.403	
LAYRAE		$P\ \bar{1}$	0.1202	1.399	
UZOLAX		C 2/c	0.1216	1.434	{[Ma2(AB)2] DAC-6 C2v a 2 C'' [4 1 3 5 6 2]}
OLENUO		$P\ \bar{1}$	0.1222	1.431	
METFEW		P b c a	0.1231	1.508	
			0.1395	1.820	
JAWQAA			0.1247	1.526	
YORJUK01		$P\ 2_1/c$	0.1250	1.553	
LATRED			0.1256	1.566	
GAQNOB		P c	0.1264	1.519	
SETDOK		$P\ 2_1/m$	0.1275	1.614	
KUTVOK		F d d 2	0.1276	1.614	
QEWPEP		$P\ 2_1/n$	0.1278	1.574	
QEWQAM		$P\ 2_1/c$	0.1311	1.691	
AVOPUY		$P\ 2_1/n$	0.1317	1.652	
ELUDIY			0.1367	1.318	
REQPAF		C 2/c	0.1397	1.920	
PADFOQ	Zr	P b c a	0.1403	1.941	
DUCQEY	Sn	P n m a	0.1404	1.889	
			0.1408	1.948	
ZISCEL	Pb	P b c n	0.1422	1.839	

AHAMSN	Sn	P 2_1/c	0.1441	1.057	{[Ma2(AB)2] DAC-6 C2v a 2 C'' [4 1 3 5 6 2]}
			0.1452	1.111	
ZENSIX	Pb	P 2_1/n	0.1444	1.520	
BENDIJ	Sn	P 2_1/c	0.1463	1.108	
EXIGEX		P $\bar{1}$	0.1485	2.133	
ELUDUK		P b c a	0.1490	1.563	
			0.1514	1.663	
QEWPAL		P 2_1/c	0.1511	2.244	
WEVYED		P $\bar{1}$	0.1530	2.296	
KAMGIP		P 2_1/c	0.0981	0.783	{[Ma2(AB)2] DAC-6 Cs a 1 C' [3 1 4 5 6 2]}
			0.1062	0.961	
AGESUA		P n a 2_1	0.0978	0.849	
			0.0983	0.877	
FINQUP	Cd	P 2_1/c	0.1140	1.149	
SOWPIE	Zr	P 2_1/n	0.1226	1.151	{[Ma2(AB)2] DAC-6 C2 c 2 B [4 3 1 2 6 5]}<br>{[Ma2(AB)2] DAC-6 C2 c 2 B [6 3 1 2 4 5]}
SIGGUM	Sn	P b c a	0.1494	2.088	{[Ma2(AB)2] DAC-6 C2 c 2 B [3 4 1 2 5 6]}<br>{[Ma2(AB)2] DAC-6 C2 c 2 B [3 6 1 2 5 4]}
NENVAG	Cd	P 2_1/c	0.1332	1.690	{[Ma2(AB)2] DAC-6 C1 c 1 A [1 2 4 3 6 5]}<br>{[Ma2(AB)2] DAC-6 C1 c 1 A [1 3 6 5 4 2]}
REHBAJ	Y	P $\bar{1}$	0.0954	0.783	{[Ma2b2(AA)] DAC-6 C2v a 2 C [1 3 5 6 2 4]}
QUSXIO	Dy	P 2_1	0.1242	1.506	
			0.1400	1.924	
TINYAQ	Sn	P $\bar{1}$	0.1401	1.710	{[Ma2b2(AA)] DAC-6 Cs a 1 B [1 3 2 5 6 4]}
FUDJAR	U		0.1303	1.426	{[Ma2b2(AB)] DAC-6 Cs a 1 B [1 3 5 6 2 4]}
			0.1407	1.644	
FUBNAR	Mo	P 2_1/n	0.1492	1.751	{[Ma2b2c2] DAC-6 C2v a 2 C [5 1 3 4 6 2]}
QICNOF		P 2_1 2_1 2_1	0.1060	0.681	{[Ma2bc(AA)] DAC-6 C1 c 1 A [1 4 2 5 3 6]}
SAZWAT	Nd	P 2_1/c	0.0740	0.325	{[Ma3b3] DAC-6 Cs a 1 A [4 1 2 5 6 3]
			0.0977	0.604	
SIDLEY	Ta	P 2_1/n	0.0732	0.332	{[Ma3b(AB)] DAC-6 Cs a 1 B [4 1 2 6 5 3]}
AWEKEV			0.1098	0.741	
HURLIS			0.1221	0.962	
UJOJIM	Sn	P 2_1/c	0.1223	1.484	
JEZZIA	Yb	P 2_1/n	0.1323	1.333	
ERUJUW	Ce	P 2_1/c	0.1343	1.576	{[Ma3b2c] DAC-6 C1 c 1 A [1 4 2 5 3 6]}<br>{[Ma3b2c] DAC-6 C1 c 1 A [1 6 2 4 3 5]}

UZUCOI	Ba	P 2/c	0.0790	0.608	{[Ma4(AA)] DAC-6 C2v a 2 C [1 2 5 6 3 4]}
YUTHOL	Sm	P 2_1/n	0.0942	0.861	
YUTHIF	Eu		0.0956	0.889	
YUTHUR	Yb	C 2 2 2_1	0.1144	1.303	
ITINAB	Na	P 2_1/n	0.1291	1.077	
HABLII	Th		0.1209	1.304	{[Ma6] DAC-6 C2v a 2 A [1 2 3 4 5 6]}
XUXHEG	K	P 2_1 2_1 2	0.1331	1.748	
XUXHAC			0.1340	1.768	
CUNVIT	Y	P 2_1/c	0.1244	1.520	{[Mab(AA)(AB)] DAC-6 C1 c 1 A [2 1 3 4 5 6]}<br>{[Mab(AA)(AB)] DAC-6 C1 c 1 A [5 1 3 4 2 6]}
VIQNAN	Sn	P $\bar{1}$	0.1108	1.075	{[Mab(AA)(AB)] DAC-6 C1 c 1 A [3 4 1 6 2 5]}<br>{[Mab(AA)(AB)] DAC-6 C1 c 1 A [3 5 1 6 2 4]}
IVOJAE	W	P 2_1/c	0.0724	0.365	{[Mab(AA)2] DAC-6 C1 c 1 A [2 1 3 4 5 6]}<br>{[Mab(AA)2] DAC-6 C1 c 1 A [3 1 5 6 2 4]}
GEFQAJ			0.0802	0.518	
IVOHUW			0.0960	0.698	
EBEMON			0.1045	0.856	
GEFLOS		P b c a	0.1099	0.973	
GEFLOS01			0.1099	0.973	
DEHQAJ		P 2_1/n	0.1517	1.908	
XETMEQ	Yb	P 2_1/c	0.1522	2.181	
OBARIS	Ti	P $\bar{1}$	0.1317	1.696	{[Mab(AA)2] DAC-6 C1 c 1 A [3 1 2 5 6 4]}<br>{[Mab(AA)2] DAC-6 C1 c 1 A [3 1 4 2 5 6]}
JEHTAR	Sn		0.1463	1.836	{[Mab(AB)(CD)] DAC-6 C1 c 1 A [3 2 1 6 5 4]}<br>{[Mab(AB)(CD)] DAC-6 C1 c 1 A [3 4 1 6 5 2]}
TEXDUX		P 2_1/n	0.1148	1.033	{[Mab(AB)2] DAC-6 Cs a 1 B [4 1 3 5 6 2]}
OVIQOA	Nd	P $\bar{1}$	0.1497	1.864	{[Mab(AB)2] DAC-6 C1 c 1 A [4 3 1 2 6 5]}<br>{[Mab(AB)2] DAC-6 C1 c 1 A [6 3 1 2 4 5]}

Occurrence of the New 7-Coordinate Geometries Defined in This Study in Known Crystallographic Structures of Metal Complexes

Three coordination polyhedra (CPs) traditionally dominate discussions on 7-coordinate metal complexes: pentagonal bipyramid (PBPY-7), capped trigonal prism (CTPR-7), and capped octahedron (COC-7). The additional seven-vertex geometries commonly considered for these compounds include the hexagonal pyramid (HPY-7), elongated trigonal pyramid (ETPY-7), and heptagonal planar shape (HP-7).

Our research demonstrates that when all possible 34 seven-vertex polyhedra are constructed and optimized to achieve maximum symmetry and minimum repulsion (MSMR), new polyhedra emerge as valid geometries for hepta-coordinate compounds. These geometries maintain their distinctiveness, even after accounting for crystallographic smearing effects.

Extending our findings on hexacoordination, we have also identified the new seven-coordinate polyhedra introduced in this work within the crystallographic structures reported in structural databases. These occurrences are particularly found in s-block and f-block metal complexes featuring polydentate ligands, such as crown ethers and cryptands. Table S15 compiles data from the Cambridge Structural Database (CSD) for all structures containing seven-coordinate metal complexes, whose molecular geometries deviate from the conventional PBPY-7, CTPR-7, and COC-7 types. For each entry, we report the CSD reference code, atomic symbol of the metal center, crystal space group, abbreviated label of the identified seven-vertex coordination polyhedron, and minimum root-mean-square deviation (RMSD) and Continuous Shape Measures (CShM) values between the empirical structure and its corresponding ideal geometry.

Table S15. A table listing all 7-coordinate complexes considered in this work retrieved from crystallographic structures whose coordination polyhedron is not the capped octahedron (COC-7), the capped trigonal prism (CTPR-7) and the pentagonal bipyramid (PBPY-7). The list informs the reference CSD code (refcode) of the crystallographic structure, the metal center of the coordination complex, the spatial group of the crystal structure, the coordination polyhedron of the metal complex, and RMSD and CShM values.

Refcode	Metal	Spatial group	Coordination polyhedron	RMSD	CShM
ZOZDIE	K	P 2_1	HPY-7	0.0686	0.408
XOZMEH		C c		0.0694	0.569
				0.0731	0.393
				0.0967	0.394
				0.1150	0.842
ZOZDIE01		P 2_1		0.0698	0.426
QANXOU		R 3 m		0.0705	0.352
FUCPEY		P $\bar{1}$		0.0737	0.430
				0.0845	0.447
WAJGET				0.0749	0.439
DOLQIG				0.0758	0.509
RIZLUI		P 2_1/n		0.0766	0.576
PUVYOV		P b c a		0.0777	0.595
VUYZEU		C 2/c		0.0782	0.369
WAJBUH		P 2_1/c		0.0784	0.475
MIFXIJ		P $\bar{1}$		0.0811	0.456
NEBBEC01		P 2_1 2_1 2_1		0.0871	0.653
FIXJII		P $\bar{1}$		0.0936	0.604
TIVLUI		P 2_1/n		0.1012	0.953
NEBBIG		P 2_1 2_1 2_1		0.1025	0.674
RABGUA		I 2/a		0.1030	0.932
				0.1030	0.932
YEHLON		P 2_1/n		0.1111	1.057
				0.1264	1.434
YIJWUK				0.1120	1.033
NAQXUZ		P 2_1/c		0.1129	0.755
YOBMAE				0.1136	0.925
VEJTIP		P $\bar{1}$		0.1138	1.077
				0.1138	1.077
ICOGUF		I a		0.1151	0.853
DKCRKT10		P 2_1/c		0.1159	0.543

HUFRAB	Na	P $\bar{1}$	HPY-7	0.1177	0.856
				0.1177	0.856
NEWZUL	K	P 2_1/n		0.1181	1.201
WUMSOP		F d d 2		0.1184	0.897
UPOFOX		P $\bar{1}$		0.1191	0.766
CETGUF01		P 2_1/c		0.1259	1.345
CAHCIZ		F d d 2		0.1263	0.880
HAMGEJ		C c		0.1266	1.473
HEPDEM		P 2_1/n		0.1278	1.289
POGLOM		C 2		0.1279	1.144
				0.1337	1.267
YIWJEW01		P n a 2_1		0.1292	1.534
YIWJEW				0.1306	1.501
POGLOM		C 2		0.1337	1.267
DOLQIG		P $\bar{1}$		0.1357	1.305
CARDIL		P 2_1/c		0.1358	1.645
WODKIM	Na	P $\bar{1}$		0.1362	1.453
UDIHIA	K	P 2_1/c		0.1374	1.240
LUPYIH				0.1392	1.701
CARDIL01		C c		0.1393	1.735
QEWCOK		P 2_1/c		0.1437	1.103
USUJOJ				0.1440	1.780
				0.1452	1.804
USOZIM		P $\bar{1}$		0.1446	1.623
TITGIP		P 2_1/c		0.1447	1.808
				0.1447	1.808
TADNER	Na		DPAC-7	0.1087	0.912 (TSPPY-7) 1.520 (DPAC-7)
				0.1087	0.912 (TSPPY-7) 1.520 (DPAC-7)
LASVUW				0.1115	1.105 (TSPPY-7) 1.245 (DPAC-7)
SIGLEZ		C 2/m		0.1119	1.210 (TSPPY-7) 1.975 (DPAC-7)
TADLUF		P 2_1 2_1 2_1		0.1150	0.965 (TSPPY-7) 1.259 (DPAC-7)
KEPGET		C 2/c		0.1240	1.069 (TSPPY-7) 1.095 (DPAC-7)
				0.1240	1.069 (TSPPY-7) 1.095 (DPAC-7)
SEHHAO		P 2_1/c		0.1241	1.371 (TSPPY-7) 1.846 (DPAC-7)
FIQKOF		C 2/m		0.1249	1.553 (TSPPY-7) 2.344 (DPAC-7)
				0.1250	1.552 (TSPPY-7) 2.344 (DPAC-7)
SAMRIH		P 2_1/n		0.1265	1.484 (TSPPY-7) 2.059 (DPAC-7)

TAYVOF		$P\ 2_1/m$		0.1274	1.576 (TSPPY-7) 2.282 (DPAC-7)
CARJAJ		$P\ 2_1\ 2_1\ 2_1$		0.1277	1.589 (TSPPY-7) 2.293 (DPAC-7)
AMPICC02		$P\ 2_1/n$		0.1287	1.419 (TSPPY-7) 1.864 (DPAC-7)
JAZKAW		$P\ 2_1/c$		0.1329	1.632 (TSPPY-7) 2.354 (DPAC-7)
GARRAT		$P\ \bar{1}$	DPAC-7	0.1358	1.365 (TSPPY-7) 1.828 (DPAC-7)
OBUKEB		$P\ 2_1/c$		0.1369	1.650 (TSPPY-7) 2.090 (DPAC-7)
				0.1369	1.650 (TSPPY-7) 2.090 (DPAC-6)
PASLEA		$P\ 2_1/a$		0.1385	1.661 (TSPPY-7) 2.062 (DPAC-7)
VATLUX		$P\ \bar{1}$		0.1443	1.701 (TSPPY-7) 2.138 (DPAC-6)
				0.1443	1.701 (TSPPY-7) 2.139 (DPAC-7)
REGCEL		$P\ 2_1/c$		0.0781	0.488
BIZTOT		P b c a		0.0818	0.535
POZTNA		$P\ 2_1/c$		0.0907	0.644
RAHMOE		$P\ \bar{1}$		0.1089	1.113
MUGHON		$P\ 2_1/n$	Δ/Λ-SHEAPR-7	0.1221	1.122
GUSQUG	Y	$P\ \bar{1}$		0.1334	1.719
UMITOZ	Na	$P\ 2_1$		0.1380	1.532
MAWYEO	Cd	$P\ 2_1/n$		0.1385	1.460
POZGOA	Na	I a		0.1393	1.774
HOYGEL	Eu	$P\ 2_1/n$		0.0543	0.278
BENGAC		$P\ 2_1/c$		0.0959	0.593
YIHFUS	Na	$P\ n\ a\ 2_1$		0.1048	1.037
ICABIB		$P\ 6_3$		0.1076	1.003
CUQKOP	Ag		HECU-7	0.1092	1.124
				0.1092	1.124
YIHFOM	Na	$P\ 2_1/c$		0.1232	1.295
YEMWIW	Y			0.1355	1.685
PUVDOZ		$P\ 2_1/n$		0.1368	1.715
AVIMUP		$P\ 2_1\ 2_1\ 2_1$		0.1415	1.857
HEXWUC		$R\ \bar{3}\ c$		0.0372	0.125
	Na			0.0372	0.125
HEXXEN		$C\ 2/c$	ETPY-7	0.0453	0.164
				0.0453	0.164
WITNIZ		$P\ 2_1\ 2_1\ 2_1$		0.1353	0.973
CUQZIA	Ce	$P\ 2_1\ 3$		0.1361	1.838
VAXWUN	Mn	$P\ \bar{1}$	DTT-7	0.0760	0.564
VAHROO	U			0.0855	0.700

BIVNEZ	Na	$P\ n\ a\ 2_1$	DTT-7	0.1270	0.754
BIVNEZ10				0.1270	0.754
WUDFEJ	W	$P\ \bar{1}$		0.1366	1.725
SOTSAW	Hf	$I\ \bar{4}\ 3\ d$		0.1449	1.608
YAPPEK	Na	$P\ 2_1/c$	TSPPY-7	0.0996	0.857 (DPAC-7) 1.463 (TSPPY-7)
OBUKIF		$P\ c\ c\ n$		0.1403	1.933 (DPAC-7) 2.506 (TSPPY-7)
				0.1403	1.933 (DPAC-7) 2.506 (TSPPY-7)
JAZKIE		$P\ 2_1/c$		0.1439	1.732 (DPAC-7) 2.216 (TSPPY-7)
NOSPIU		$C\ c$	Δ/Λ-THTB-7	0.1308	1.285
NOSPIU01				0.1308	1.283
IHEQOC	Eu	$P\ 2_1/n$		0.1418	1.300
TASCEV	Na	$P\ 2_1\ 2_1\ 2_1$	HEOB-7	0.1343	0.728
IZUZIO		$P\ \bar{1}$	FPSS-7	0.1080	1.101
RILGUO	Cs		HP-7	0.0899	0.497

Continuous Symmetry Operations Measurements (CSOM) analyses

Table S16. List of crystallographic structures analyzed in this study that contain hexacoordinate metal complexes with mono- and/or bidentate ligands, whose coordination environments are best described by the digonal anticupola (DAC-6) geometry. For each entry, the table reports the Cambridge Structural Database (CSD) reference code (refcode), the identity of the metal center, the CSOM deviation value (σ_{sym}), and the corresponding CSOM-assigned point group. In a small number of cases, CSOM assigned a D_{3h} point group; these assignments likely reflect borderline cases, as indicated by their relatively larger σ_{sym} values, consistent with geometries positioned near a symmetry boundary.

Refcode	Metal	CSOM (PG)
EYODAY	Sn	0.527 (C_{2v})
SUZWUF		9.345 (D_{3h})
GIZWUJ	Lu	2.353 (C_{2v})
AHAPEF	Cd	0.786 (C_{2v})
MIWGOR	Y	2.068 (C_{2v})
KAXMUR	Nb	0.094 (C_{2v})
PABZTA	Ta	0.300 (C_{2v})
SAWWIZ	V	0.011 (C_{2v})
HAMKEM	Cd	2.059 (C_{2v})
MARSEE	Mo	4.642 (C_{2v})
GIHLAL	Gd	0.491 (C_{2v})
JUWTAY	Eu	4.002 (C_{2v})
QIGYEK	Sn	5.823 (C_{2v})
		5.218 (C_{2v})
YIQLUG	Cd	6.605 (D_{3h})
PUNFAI	Mn	4.184 (D_{3h})
EJUVIP	Dy	4.295 (C_{2v})
XUMPED	Mn	1.034 (C_{2v})
TAQVOW		0.066 (C_{2v})
KUTZUU		0.104 (C_{2v})
BAKNIK01		0.000 (C_{2v})
BAKNIK02		0.031 (C_{2v})
JUDSEG	Sn	0.371 (C_{2v})
AXAKAL		0.216 (C_{2v})
ORONIU		0.036 (C_{2v})
UGEFAP		0.091 (C_{2v})
UZAHUZ		0.159 (C_{2v})

Refcode	Metal	CSOM (PG)
LUYSED		0.031 (C_{2v})
REWCIG		0.126 (C_{2v})
LUYSED01		0.035 (C_{2v})
POSYEB		0.122 (C_{2v})
AXAKEP		0.070 (C_{2v})
FOFDEJ01		0.056 (C_{2v})
ETCMSN02		0.109 (C_{2v})
QEQPEH		12.171 (D_{3h})
		12.779 (D_{3h})
AZIPOO		0.287 (C_{2v})
KURKIS		0.005 (C_{2v})
HOCQAR		0.207 (C_{2v})
KEJGUD		0.301 (C_{2v})
VARJUU	Sn	14.408 (D_{3h})
CIHCIF		0.005 (C_{2v})
SEKBOA		0.306 (C_{2v})
LUKBIC		0.629 (C_{2v})
LELWAD		0.086 (C_{2v})
RAQQEH		12.075 (D_{3h})
FILZUW		0.076 (C_{2v})
WUCQAM		0.136 (C_{2v})
KUMDAX		0.072 (C_{2v})
IDOLOD01		0.067 (C_{2v})
XUXHIH		0.389 (C_{2v})
JUMWAS		0.248 (C_{2v})
NALBEH		0.198 (C_{2v})
HARLOE		0.110 (C_{2v})

Refcode	Metal	CSOM (PG)
HOCPOE		0.978 (C_{2v})
ABOXEV	Sn	0.575 (C_{2v})
OGURAJ		0.887 (C_{2v})
SIHTEI		0.779 (C_{2v})
MURGUB	Ti	0.486 (C_{2v})
FIMBOT	Sn	0.304 (C_{2v})
JIVJED	U	4.262 (D_{3h})
WEBYOR		0.199 (C_{2v})
CAYYOQ01	Sn	0.046 (C_{2v})
FOTBOF		0.042 (C_{2v})
PAHZAZ	Pb	10.288 (D_{3h})
IMECAE		0.132 (C_{2v})
GALTUM	Sn	0.145 (C_{2v})
GALSAR		0.064 (C_{2v})
TUMTEC	Pb	12.738 (C_{2v})
REXKOX	Sn	0.100 (C_{2v})
CUCTUP	Mo	0.155 (C_{2v})
MEMWEH		0.138 (C_{2v})
	Sn	0.200 (C_{2v})
		10.790 (C_{2v})
IXENEE		0.138 (C_{2v})
LAJSOG	Pb	11.435 (D_{3h})
JEDJIL		0.092 (C_{2v})
		10.650 (D_{3h})
AWOGID	Sn	0.702 (C_{2v})
IXENII		0.381 (C_{2v})
CIPCEK		0.107 (C_{2v})

Refcode	Metal	CSOM (PG)
WEYNAP01		0.194 (C_{2v})
YIBWUB		0.089 (C_{2v})
WEYNAP		0.194 (C_{2v})
FUNRUB	Sn	0.056 (C_{2v})
ELUDOE		0.146 (C_{2v})
IJIXUV		11.270 (D_{3h})
		11.771 (D_{3h})
JAYPAB		0.003 (C_{2v})
CUCTOJ	Mo	0.329 (C_{2v})
CIPBUZ		0.269 (C_{2v})
WEVYIH		9.215 (C_{2v})
EDUPEA	Sn	0.140 (C_{2v})
SIYLUH		0.457 (C_{2v})
VOJKIQ		7.780 (D_{3h})
KUHPOS		0.052 (C_{2v})
XECDEO	Pb	0.320 (C_{2v})
MASOMO10		0.854 (C_{2v})
MASOMO	Mo	0.854 (C_{2v})
TARPOS		0.089 (C_{2v})
GUQPUD	W	0.182 (C_{2v})
		8.805 (D_{3h})
JEJGIP		0.091 (C_{2v})
QEWPOZ		0.352 (C_{2v})
YORLIA	Sn	0.007 (C_{2v})
YEPSOB		7.048 (C_{2v})
QEWPUF		0.590 (C_{2v})
JAWQEE		0.115 (C_{2v})

Refcode	Metal	CSOM (PG)
GAQNUH01		14.466 (D_{3h})
CIPCAG		9.435 (C_{2v})
ARAXIA		0.194 (C_{2v})
NALCUY		0.015 (C_{2v})
YORLIA01		0.015 (C_{2v})
QEWPIT		0.019 (C_{2v})
XIJVIW		0.002 (C_{2v})
KAMGEL		0.040 (C_{2v})
LAYRAE		0.239 (C_{2v})
UZOLAX		15.396 (D_{3h})
OLENUO		0.329 (C_{2v})
METFEW	Sn	0.119 (C_{2v})
		0.419 (C_{2v})
JAWQAA		14.353 (D_{3h})
YORJUK01		0.052 (C_{2v})
LATRED		0.048 (C_{2v})
GAQNOB		0.387 (C_{2v})
SETDOK		0.011 (C_{2v})
KUTVOK		0.296 (C_{2v})
QEWPEP		0.321 (C_{2v})
QEWQAM		0.245 (C_{2v})
AVOPUY		0.271 (C_{2v})
ELUDIY		0.270 (C_{2v})
REQPAF		0.287 (C_{2v})
PADFOQ	Zr	0.054 (C_{2v})
DUCQEY	Sn	0.024 (C_{2v})
		0.022 (C_{2v})
ZISCEL	Pb	0.044 (C_{2v})
AHAMSN	Sn	0.351 (C_{2v})
		0.412 (C_{2v})
ZENSIX	Pb	0.198 (C_{2v})
BENDIJ	Sn	0.324 (C_{2v})
EXIGEX		0.506 (C_{2v})

Refcode	Metal	CSOM (PG)
ELUDUK		9.793 (C_{2v})
		0.013 (C_{2v})
QEWPAL		0.149 (C_{2v})
WEVYED	Sn	0.121 (C_{2v})
KAMGIP		0.373 (C_{2v})
		12.894 (C_{2v})
AGESUA		0.022 (C_{2v})
		10.113 (C_{2v})
FINQUP	Cd	0.475 (C_{2v})
SOWPIE	Zr	1.163 (C_{2v})
SIGGUM	Sn	1.132 (C_{2v})
NENVAG	Cd	0.906 (C_{2v})
REHBAJ	Y	0.434 (C_{2v})
QUSXIO	Dy	0.517 (C_{2v})
		0.168 (C_{2v})
TINYAQ	Sn	0.483 (C_{2v})
FUDJAR	U	8.158 (C_{2v})
		7.186 (C_{2v})
FUBNAR	Mo	0.396 (C_{2v})
QICNOF		0.571 (C_{2v})
SAZWAT	Nd	0.975 (C_{2v})
		1.104 (C_{2v})
SIDLEY		1.087 (C_{2v})
AWEKEV	Ta	2.433 (C_{2v})
HURLIS		3.088 (C_{2v})
UJOJIM	Sn	0.126 (C_{2v})
JEZZIA	Yb	0.814 (C_{2v})
ERUJUW	Ce	3.537 (C_{2v})
UZUCOI	Ba	8.441 (C_{2v})
YUTHOL	Sm	6.973 (C_{2v})
YUTHIF	Eu	0.346 (C_{2v})
YUTHUR	Yb	0.778 (C_{2v})
ITINAB	Na	6.412 (C_{2v})

Refcode	Metal	CSOM (PG)
HABLII	Th	1.496 (C_{2v})
XUXHEG	K	0.108 (C_{2v})
XUXHAC		0.118 (C_{2v})
CUNVIT	Y	1.159 (C_{2v})
VIQNAN	Sn	0.661 (C_{2v})
IVOJAE		0.856 (C_{2v})
GEFQAJ		12.377 (D_{3h})
IVOHUW		1.290 (C_{2v})
EBEMON	W	1.158 (C_{2v})
GEFLOS		1.462 (C_{2v})
GEFLOS01		1.462 (C_{2v})
DEHQAJ		1.601 (C_{2v})
XETMEQ	Yb	3.741 (C_{2v})
OBARIS	Ti	3.577 (C_{2v})
JEHTAR	Sn	2.727 (C_{2v})
TEXDUX		10.547 (C_{2v})
OVIQOA	Nd	11.402 (C_{2v})

Table S17. List of crystallographic structures analyzed in this study that contain heptacoordinate metal complexes whose coordination environments are best described by the new thermally distinguishable polyhedral shapes (TDPSs) introduced in this work. For each entry, the table reports the Cambridge Structural Database (CSD) reference code (refcode), the identity of the metal center, the CSOM deviation value (σ_{sym}), and the corresponding CSOM-assigned point group. In a few cases, CSOM identified point groups of slightly lower symmetry, which may reflect structural variations encompassed by thermal smearing, yet still broadly consistent with our TDPS classifications.

Refcode	TDPS (PG)	Metal	CSOM (PG)
VAXWUN	DTT-7 (C_{3v})	Mn	0.241 (C_{3v})
VAHROO		U	1.077 (C_{3v})
BIVNEZ		Na	0.108 (C_s)
BIVNEZ10			0.108 (C_s)
WUDFEJ		W	3.995 (C_{3v})
SOTSAW		Hf	2.068 (C_{3v})
IZUZIO	FPSS-7 (C_{2v})	Na	0.759 (C_s)
HOYGEL	HECU-7 (C_{2v})	Eu	0.667 (C_{2v})
BENGAC		Na	0.545 (C_2)
YIHFUS			0.092 (C_s)
ICABIB			1.421 (C_s)
CUQKOP		Ag	0.804 (C_2)
			0.804 (C_2)
YIHFOM		Na	1.747 (C_s)
YEMWIW		Y	1.536 (C_2)
PUVDOZ		Na	0.200 (C_s)
AVIMUP			1.779 (C_2)

Refcode	TDPS (PG)	Metal	CSOM (PG)
TADNER	DPAC-7 (C_s)	Na	0.051 (C_s)
			0.051 (C_s)
LASVUW			0.604 (C_s)
SIGLEZ			0.000 (C_s)
TADLUF			1.367 (C_s)
KEPGET			3.022 (C_s)
SEHHAO			0.526 (C_s)
FIQKOF			0.000 (C_s)
RAHMOE			0.000 (C_s)
SAMRIH			0.160 (C_s)
TAYVOF		Y	0.000 (C_s)
CARJAJ		Na	0.892 (C_s)
AMPICC02		Cd	0.819 (C_s)
JAZKAW		Na	0.235 (C_s)
GARRAT			1.370 (C_s)
OBUKEB			0.642 (C_s)
			0.642 (C_s)
PASLEA			2.130 (C_s)
VATLUX			3.353 (C_s)
			3.353 (C_s)

Refcode	TDPS (PG)	Metal	CSOM (PG)
YAPPEK	TSPPY-7 (C_s)	Na	1.580 (C_s)
OBUKIF			0.548 (C_s)
			0.548 (C_s)
JAZKIE			0.946 (C_s)
TASCEV	HEOB-7 (C_s)	Na	0.239 (C_s)
NOSPIU	Δ/Λ-THTB-7 (C_2)	Na	3.797 (C_2)
NOSPIU01			3.791 (C_2)
IHEQOC		Eu	1.227 (C_2)
REGCEL	Δ/Λ-SHEAPR-7 (C_2)	Na	0.198 (C_2)
BIZTOT			0.258 (C_2)
POZTNA			0.241 (C_2)
RAHMOE			0.854 (C_2)
MUGHON			0.434 (C_2)
GUSQUG		Y	0.376 (C_2)
UMITOZ		Na	3.253 (C_s)
MAWYEO		Cd	3.476 (C_2)
POZGOA		Na	2.887 (C_s)

RMSD Alignment and Displacement Data for Thermally Indistinguishable Polyhedral Shape Classification of 6- and 7-Vertex Polyhedra

As discussed in the main text, we introduce the concept of polyhedral shapes that are thermally indistinguishable to describe geometries whose maximum symmetry minimum repulsion (MSMR) representations can be interconverted through vertex displacements of magnitudes comparable to the typical thermal motion in crystal phases. To establish a quantitative criterion for this classification, we determined a threshold value of 0.1541for the maximum atomic displacement by taking isotropic atomic displacement parameter ($\mathbf{U_{iso}}$) data from a set of over 42,000 good crystallographic structures retrieved from the Cambridge Structure Database (CSD). Statistical analyses of these parameters provided a robust empirical basis for determining the threshold displacement value.

For each pair of optimized polyhedra$\{P_1, P_2\}$ with 6 or 7 vertices, we determined their best alignment by minimizing the root mean square deviation (RMSD) function. Following the alignment, we reordered the indices of their Cartesian coordinate matrices based on the spatial proximity of the aligned vertices, and calculated the Euclidean separation distances for each corresponding vertex pair $\{\boldsymbol{v}_1^i, \boldsymbol{v}_2^i\}$, where $\boldsymbol{v}_1^i$ is the ith vertex of P_1 and $\boldsymbol{v}_2^i$ the ith vertex of P_2, and is the closest to $\boldsymbol{v}_1^i$. Two polyhedra are classified as thermally indistinguishable when all the corresponding vertex pair separation distances fall below the empirically determined cut-off value. Tables S16 and S17 present the separation distances of every vertex pair for all possible pairs of 6- and 7-vertex shapes after the RMSD-based alignment.

Table S18. Minimum values of root mean-square deviation (RMSD) and Euclidean distances of the aligned vertices $d(v_i^1, v_i^2)$ calculated for all possible combination of pair of 6-vertex geometries, P_1 and P_2. These include coordination polyhedra and the hexagonal planar geometry.

Pair of geometries		min RMSD	Vertex pair euclidean distance $d(v_i^1, v_i^2)$					
			v_1	v_2	v_3	v_4	v_5	v_6
OC-6	TPR-6	0.4176	0.4176	0.4176	0.4176	0.4176	0.4177	0.4177
OC-6	PPY-6	0.5625	0.1861	0.1861	0.3407	0.6114	0.6114	0.9825
OC-6	CSPY-6	0.4179	0.4006	0.4014	0.4172	0.4179	0.4338	0.4354
OC-6	Δ/Λ TAW-6	0.416	0.4138	0.4138	0.4139	0.4139	0.4204	0.4204
OC-6	STBPY-6	0.3336	0.1477	0.1477	0.3741	0.3741	0.4148	0.4148
OC-6	DAC-6	0.3262	0.1437	0.1437	0.3668	0.3668	0.4049	0.405
OC-6	HP-6	0.6058	0.6058	0.6058	0.6058	0.6058	0.6058	0.6058
TPR-6	PPY-6	0.3999	0.0932	0.2419	0.2419	0.5177	0.5318	0.5318
TPR-6	CSPY-6	0.0166	0.0115	0.0133	0.0174	0.0175	0.0192	0.0192
TPR-6	Δ/Λ TAW-6	0.0061	0.0043	0.0043	0.0055	0.0055	0.0079	0.008
TPR-6	STBPY-6	0.2492	0.0839	0.084	0.1628	0.1628	0.3909	0.3909
TPR-6	DAC-6	0.2495	0.0835	0.0835	0.1636	0.1636	0.3912	0.3912
TPR-6	HP-6	0.6228	0.4510	0.4510	0.4510	0.4510	0.8699	0.8699
PPY-6	CSPY-6	0.3856	0.0829	0.2266	0.2267	0.5060	0.5130	0.5130
PPY-6	Δ/Λ TAW-6	0.3975	0.0969	0.2387	0.2398	0.5125	0.5282	0.5317
PPY-6	STBPY-6	0.3125	0.0128	0.1504	0.1504	0.3710	0.3710	0.5151
PPY-6	DAC-6	0.3169	0.0214	0.1497	0.1498	0.3744	0.3745	0.5262
PPY-6	HP-6	0.5748	0.1542	0.1542	0.3215	0.6453	0.6453	0.9994
CSPY-6	Δ/Λ-TAW-6	0.0145	0.0075	0.0129	0.0137	0.0142	0.0168	0.019
CSPY-6	STBPY-6	0.2391	0.0735	0.0822	0.1511	0.1671	0.3703	0.3779
CSPY-6	DAC-6	0.2396	0.0735	0.0839	0.1521	0.1686	0.3707	0.3781
CSPY-6	HP-6	0.6222	0.4345	0.4346	0.4660	0.4660	0.8540	0.8841
Δ-TAW-6	Λ-TAW-6	0.0049	0.0025	0.0025	0.0027	0.0027	0.0075	0.0077
Δ/Λ-TAW-6	STBPY-6	0.2474	0.0863	0.0897	0.1554	0.1608	0.3872	0.3897
Δ/Λ-TAW-6	DAC-6	0.2477	0.0855	0.0894	0.1562	0.1619	0.3875	0.39
Δ/Λ-TAW-6	HP-6	0.6212	0.4486	0.4486	0.4549	0.4549	0.8657	0.8657
STBPY-6	DAC-6	0.0076	0.0040	0.0040	0.0074	0.0074	0.0100	0.0101
STBPY-6	HP-6	0.6305	0.1634	0.4386	0.4386	0.7264	0.7264	0.9582
DAC-6	HP-6	0.6279	0.1731	0.4404	0.4404	0.7236	0.7236	0.9490

Table S19. Minimum values of root mean-square deviation (RMSD) and euclidean distances of the aligned vertices $d(v_i^1, v_i^2)$ calculated for all possible combination of pair of 7-vertex geometries, P_1 and P_2. Geometries without known or given symbols are represented by their index numbers. These include coordination polyhedra and the heptagonal planar geometry.

Pair of geometries	min RMSD	Vertex pair euclidean distance $d(\mathbf{v}_i^1, \mathbf{v}_i^2)$						
		v_1	v_2	v_3	v_4	v_5	v_6	v_7
HP-7 PBPY-7	0.62465	4.62E-09	0.420993	0.420993	0.742002	0.742002	0.798672	0.798672
HP-7 HPY-7	0.54136	0.212632	0.212632	0.216989	0.216989	0.585135	0.585135	1.087261
HP-7 ETPY-7	0.63932	0.265644	0.376006	0.402432	0.425138	0.702618	0.891214	1.009238
HP-7 COC-7	0.63778	0.413745	0.477801	0.479493	0.548437	0.741658	0.742438	0.903272
HP-7 6	0.63661	0.371968	0.502775	0.507145	0.633103	0.684521	0.761585	0.859728
HP-7 DTT-7	0.61759	0.102924	0.355697	0.430763	0.49531	0.526211	0.825751	1.069198
HP-7 TT-7	0.61413	0.096335	0.348412	0.433151	0.48682	0.518359	0.819109	1.070114
HP-7 9	0.61551	0.110367	0.364231	0.4297	0.479535	0.518977	0.82592	1.068216
HP-7 10	0.61401	0.111316	0.360537	0.438512	0.476668	0.529252	0.808963	1.068963
HP-7 CTPR-7	0.62086	0.425028	0.425028	0.45197	0.638524	0.638524	0.81157	0.811571
HP-7 *Tr*BCSPY-7	0.60752	0.294518	0.294737	0.349097	0.555108	0.555216	0.914204	0.914289
HP-7 SPBPY-7	0.56167	3.86E-08	0.236815	0.236815	0.379438	0.379438	0.950848	0.950848
HP-7 SPBPY-7	0.55944	1.24E-07	0.240253	0.240253	0.370259	0.370259	0.948989	0.948989
HP-7 HECU-7	0.59346	0.426628	0.427709	0.452659	0.452734	0.523403	0.524692	1.068301
HP-7 16	0.59606	0.420756	0.420756	0.466207	0.466207	0.523918	0.523918	1.07204
HP-7 17	0.59355	0.421128	0.422473	0.457938	0.462744	0.519627	0.520273	1.0704
HP-7 18	0.59709	0.420273	0.42936	0.461428	0.462711	0.524268	0.534478	1.071013
HP-7 *Cis*BCSPY-7	0.59837	0.197807	0.223894	0.278002	0.430692	0.477838	0.896361	1.05949
HP-7 20-Δ/Λ	0.59034	0.417555	0.420546	0.452585	0.455278	0.516036	0.536937	1.059034
HP-7 DPAC-7	0.60787	0.155866	0.325906	0.457531	0.513347	0.529137	0.89049	0.954054
HP-7 22	0.61103	0.159308	0.330271	0.454665	0.516256	0.531949	0.900743	0.954709
HP-7 TSPPY-7	0.59762	0.15	0.38443	0.38443	0.519116	0.519116	0.90637	0.90637
HP-7 24-Δ/Λ	0.59753	0.155293	0.368039	0.393532	0.511602	0.530391	0.89097	0.920873
HP-7 HEOB-7	0.59159	0.189558	0.197846	0.434106	0.481883	0.566186	0.748337	1.036101
HP-7 CPPY-7	0.58147	0.171469	0.198644	0.417048	0.488209	0.562729	0.712908	1.029907
HP-7 27-Δ/Λ	0.58615	0.184371	0.189184	0.427921	0.481478	0.561145	0.738587	1.029499
HP-7 28-Δ/Λ	0.57654	0.170625	0.184961	0.445414	0.471602	0.567111	0.723128	0.99908

HP-7 29-Δ/Λ	0.58121	0.160415	0.197507	0.453296	0.475533	0.5475	0.728752	1.018567
HP-7 30-Δ/Λ	0.60664	0.181856	0.207155	0.450777	0.48421	0.588922	0.762468	1.065019
HP-7 Δ/Λ-THTB-7	0.58874	1.48E-07	0.194837	0.194837	0.547653	0.547653	0.93555	0.93555
HP-7 32-Δ/Λ	0.59315	1.45E-07	0.205925	0.205925	0.54255	0.54255	0.945851	0.945851
HP-7 Δ/Λ-SHEAPR-7	0.5998	1.11E-07	0.401271	0.401271	0.644179	0.644179	0.826552	0.826552
HP-7 34-Δ/Λ	0.63239	0.19427	0.237901	0.402502	0.48908	0.633135	0.876441	1.065316
HP-7 35-Δ/Λ	0.58975	0.03631	0.360301	0.425094	0.617229	0.641941	0.808321	0.822418
PBPY-7 HPY-7	0.52666	0.126597	0.233002	0.233002	0.29307	0.557585	0.557585	1.053223
PBPY-7 ETPY-7	0.48282	0.054886	0.353844	0.422532	0.438831	0.442358	0.622273	0.741345
PBPY-7 COC-7	0.28783	0.143348	0.147559	0.14756	0.23498	0.368263	0.368264	0.435182
PBPY-7 6	0.24898	0.094889	0.136866	0.14589	0.14589	0.320677	0.320677	0.397462
PBPY-7 DTT-7	0.33256	0.095503	0.25846	0.320231	0.320231	0.401548	0.401548	0.413123
PBPY-7 TT-7	0.33643	0.088164	0.264739	0.329601	0.329601	0.404751	0.404751	0.411732
PBPY-7 9	0.33295	0.078411	0.24912	0.316486	0.316487	0.408182	0.408182	0.41743
PBPY-7 10	0.33483	0.082175	0.257582	0.307072	0.336834	0.406155	0.409052	0.414293
PBPY-7 CTPR-7	0.25846	6.13E-08	0.160192	0.160192	0.2416	0.2416	0.387003	0.387003
PBPY-7 TrBCSPY-7	0.46858	9.76E-05	0.451691	0.451799	0.511993	0.512174	0.549652	0.54978
PBPY-7 FPSS-7	0.37613	3.56E-08	0.228681	0.228681	0.406191	0.406191	0.527139	0.527139
PBPY-7 SPBPY-7	0.37943	1.10E-07	0.221136	0.221136	0.416808	0.416808	0.530342	0.530342
PBPY-7 HECU-7	0.39183	0.001192	0.078787	0.079528	0.471717	0.472389	0.554818	0.555592
PBPY-7 16	0.39748	0.208067	0.280527	0.280527	0.379274	0.454433	0.454433	0.590259
PBPY-7 17	0.39584	0.205155	0.285903	0.285903	0.370904	0.446927	0.446927	0.595131
PBPY-7 18	0.39469	0.009686	0.090493	0.096243	0.479758	0.484845	0.548175	0.554244
PBPY-7 CisBCSPY-7	0.39591	0.217269	0.282537	0.28257	0.36152	0.457498	0.45768	0.583816
PBPY-7 20-Δ	0.39416	0.024312	0.075384	0.107177	0.466463	0.491115	0.547671	0.557693
PBPY-7 DPAC-7	0.38456	0.094073	0.244361	0.244361	0.258806	0.515852	0.535644	0.535644
PBPY-7 22	0.38755	0.104565	0.242501	0.242501	0.260241	0.523088	0.539197	0.539197
PBPY-7 TSPPY-7	0.39712	0.181595	0.226786	0.262273	0.316372	0.523956	0.534053	0.539328
PBPY-7 24-Δ/Λ	0.39903	0.160617	0.237215	0.265047	0.317707	0.516721	0.541763	0.548445
PBPY-7 HEOB-7	0.40918	0.027042	0.324535	0.324872	0.339637	0.41552	0.57931	0.580333
PBPY-7 CPPY-7	0.40964	0.062851	0.328473	0.34127	0.341272	0.404651	0.577106	0.577108
PBPY-7 27-Δ/Λ	0.4044	0.038238	0.318133	0.334156	0.334847	0.405393	0.570338	0.573335
PBPY-7 28-Δ/Λ	0.40714	0.059532	0.320437	0.329952	0.339011	0.413404	0.557301	0.590598

PBPY-7 29-Δ/Λ	0.40813	0.045938	0.310795	0.336948	0.354041	0.404709	0.556365	0.59587
PBPY-7 30-Δ/Λ	0.41758	0.015668	0.320369	0.324413	0.387713	0.398734	0.585555	0.600233
PBPY-7 Δ/Λ-THTB-7	0.41551	1.27E-07	0.330344	0.330345	0.480426	0.480427	0.514128	0.514128
PBPY-7 32-Δ/Λ	0.41461	1.56E-07	0.315598	0.315598	0.492429	0.492429	0.509484	0.509485
PBPY-7 Δ/Λ-SHEAPR-7	0.36244	1.12E-07	0.359609	0.359609	0.374204	0.374204	0.436361	0.436361
PBPY-7 34-Δ/Λ	0.35661	8.74E-08	0.26672	0.26672	0.375676	0.375676	0.48251	0.48251
PBPY-7 35-Δ/Λ	0.36249	0.043408	0.333968	0.359768	0.385743	0.408629	0.410589	0.438863
HPY-7 ETPY-7	0.39932	0.061804	0.169369	0.169369	0.459277	0.459277	0.47682	0.637025
HPY-7 COC-7	0.4448	5.62E-07	0.458553	0.458553	0.458553	0.501361	0.501362	0.501362
HPY-7 6	0.42248	0.019902	0.387094	0.409542	0.409542	0.487587	0.512825	0.512825
HPY-7 DTT-7	0.30819	2.38E-08	0.322962	0.322962	0.322962	0.342513	0.342513	0.342513
HPY-7 TT-7	0.30063	4.62E-08	0.315305	0.315305	0.315305	0.33387	0.33387	0.33387
HPY-7 9	0.30585	0.002877	0.313764	0.313764	0.327151	0.333755	0.34604	0.34604
HPY-7 10	0.30353	0.00046	0.304223	0.324875	0.324875	0.329698	0.329698	0.351963
HPY-7 CTPR-7	0.4516	0.096903	0.385385	0.406524	0.406524	0.497375	0.497375	0.666654
HPY-7 TrBCSPY-7	0.41084	8.52E-05	0.341637	0.341836	0.341936	0.342034	0.597463	0.597551
HPY-7 FPSS-7	0.28242	0.033719	0.033719	0.142761	0.222649	0.361047	0.361047	0.474772
HPY-7 SPBPY-7	0.28094	0.036158	0.036158	0.144176	0.224539	0.3592	0.3592	0.469696
HPY-7 HECU-7	0.27017	0.00097	0.197399	0.197453	0.198867	0.198921	0.420393	0.420868
HPY-7 16	0.2686	7.69E-08	0.195685	0.195685	0.195685	0.195685	0.419444	0.419444
HPY-7 17	0.2665	0.005556	0.193351	0.193351	0.196287	0.196287	0.413679	0.417356
HPY-7 18	0.27271	0.00789	0.192604	0.192604	0.204677	0.204677	0.423936	0.427573
HPY-7 CisBCSPY-7	0.27367	0.017877	0.187688	0.187892	0.210078	0.210188	0.426218	0.428302
HPY-7 20-Δ/Λ	0.27021	0.022044	0.182769	0.189436	0.194438	0.22122	0.420323	0.421766
HPY-7 DPAC-7	0.39988	0.100905	0.100905	0.12448	0.303921	0.542447	0.542447	0.634494
HPY-7 22	0.40457	0.102245	0.102245	0.122423	0.311932	0.546806	0.546806	0.643867
HPY-7 TSPPY-7	0.41605	0.100392	0.127513	0.164121	0.306061	0.492166	0.604516	0.676062
HPY-7 24-Δ/Λ	0.40493	0.085787	0.113564	0.149674	0.297809	0.485958	0.586479	0.660539
HPY-7 HEOB-7	0.30108	0.029217	0.029403	0.040689	0.203693	0.401622	0.402048	0.516457
HPY-7 CPPY-7	0.28434	0.027139	0.027139	0.051658	0.19298	0.382615	0.382617	0.481443
HPY-7 27-Δ/Λ	0.29632	0.033852	0.034202	0.045971	0.193979	0.395747	0.400389	0.505608
HPY-7 28-Δ/Λ	0.29788	0.041134	0.0486	0.074717	0.193719	0.392083	0.420228	0.493584
HPY-7 29-Δ/Λ	0.29322	0.051226	0.054069	0.055821	0.195932	0.374635	0.403701	0.501472

HPY-7 30-Δ/Λ	0.30514	0.010256	0.013481	0.014329	0.178099	0.395241	0.414432	0.54001
HPY-7 Δ/Λ-THTB-7	0.34957	0.138454	0.145236	0.226255	0.260148	0.331111	0.49871	0.581307
HPY-7 32-Δ/Λ	0.34863	0.126817	0.136486	0.228304	0.254919	0.327886	0.497882	0.586181
HPY-7 Δ/Λ-SHEAPR-7	0.40486	7.53E-08	0.183028	0.183028	0.464933	0.464933	0.569242	0.569242
HPY-7 34-Δ/Λ	0.3468	6.60E-08	0.08277	0.08277	0.439755	0.439755	0.469795	0.469796
HPY-7 35-Δ/Λ	0.41071	0.03453	0.152179	0.21786	0.469478	0.484448	0.558469	0.584791
ETPY-7 COC-7	0.40502	7.14E-07	0.375999	0.375999	0.376	0.491318	0.491318	0.491318
ETPY-7 6	0.41823	0.033755	0.343708	0.366375	0.458962	0.463683	0.497761	0.54541
ETPY-7 DTT-7	0.43001	0.016312	0.189374	0.189374	0.277537	0.465394	0.465394	0.84388
ETPY-7 TT-7	0.42687	0.01752	0.194796	0.194796	0.286412	0.458471	0.458471	0.834824
ETPY-7 9	0.42095	0.021593	0.179748	0.179748	0.269079	0.457219	0.457219	0.827539
ETPY-7 10	0.42333	0.018746	0.184642	0.185728	0.277949	0.447782	0.468103	0.829849
ETPY-7 CTPR-7	0.42346	0.118913	0.236298	0.416284	0.429496	0.481519	0.491763	0.594793
ETPY-7 TrBCSPY-7	0.12957	0.034209	0.054492	0.117911	0.12274	0.122959	0.186018	0.186274
ETPY-7 FPSS-7	0.35072	0.072516	0.170196	0.170824	0.263832	0.437193	0.508398	0.527633
ETPY-7 SPBPY-7	0.35016	0.073083	0.170047	0.172819	0.267793	0.436841	0.505287	0.525649
ETPY-7 HECU-7	0.36481	0.054502	0.167673	0.24799	0.297567	0.34525	0.438736	0.662405
ETPY-7 16	0.36277	0.055274	0.168144	0.245375	0.294911	0.341353	0.439777	0.657859
ETPY-7 17	0.3631	0.05063	0.16825	0.240621	0.295428	0.340102	0.443615	0.659079
ETPY-7 18	0.36313	0.060634	0.167999	0.250598	0.299513	0.35011	0.430424	0.656346
ETPY-7 CisBCSPY-7	0.36238	0.069255	0.167808	0.252369	0.303905	0.354445	0.423956	0.651827
ETPY-7 20-Δ/Λ	0.35723	0.073881	0.160977	0.251145	0.303025	0.342416	0.414075	0.646771
ETPY-7 DPAC-7	0.14269	0.003012	0.062394	0.095563	0.095563	0.175783	0.211489	0.211489
ETPY-7 22	0.14063	0.006652	0.060106	0.100118	0.100118	0.167479	0.208196	0.208196
ETPY-7 TSPPY-7	0.16827	0.0903	0.123882	0.152378	0.165647	0.182046	0.210438	0.215925
ETPY-7 24-Δ/Λ	0.15861	0.077952	0.108105	0.13488	0.147804	0.188152	0.200123	0.207005
ETPY-7 HEOB-7	0.15386	0.015992	0.073579	0.074401	0.120706	0.17344	0.174714	0.281635
ETPY-7 CPPY-7	0.1648	0.004727	0.089198	0.0892	0.156518	0.179022	0.179023	0.292545
ETPY-7 27-Δ/Λ	0.16117	0.011001	0.076647	0.081243	0.131686	0.182429	0.182706	0.291934
ETPY-7 28-Δ/Λ	0.16412	0.038523	0.045588	0.082388	0.146021	0.154825	0.220203	0.290545
ETPY-7 29-Δ/Λ	0.16385	0.043136	0.081574	0.099737	0.138169	0.148795	0.209761	0.290239
ETPY-7 30-Δ/Λ	0.15747	0.055852	0.05682	0.077833	0.097318	0.155098	0.183021	0.306828
ETPY-7 Δ/Λ-THTB-7	0.17896	0.056509	0.105149	0.130349	0.154334	0.171784	0.239567	0.286733

ETPY-7 32-Δ/Λ	0.1786	0.056368	0.113645	0.142197	0.162061	0.166115	0.23743	0.277033
ETPY-7 Δ/Λ-SHEAPR-7	0.16913	0.027308	0.053464	0.06035	0.094981	0.108263	0.282832	0.30372
ETPY-7 34-Δ/Λ	0.19786	0.051853	0.0525	0.082113	0.090711	0.173692	0.272613	0.38617
ETPY-7 35-Δ/Λ	0.16201	0.039038	0.060327	0.067773	0.071823	0.110864	0.257361	0.300499
COC-7 6	0.06321	0.014788	0.014788	0.029925	0.039904	0.082359	0.082359	0.107138
COC-7 DTT-7	0.13958	6.19E-07	0.138176	0.138176	0.138177	0.162377	0.162378	0.162378
COC-7 TT-7	0.14722	5.85E-07	0.145913	0.145913	0.145913	0.171116	0.171117	0.171117
COC-7 9	0.14246	0.005305	0.129682	0.146325	0.146325	0.15794	0.157941	0.180322
COC-7 10	0.1451	0.004346	0.138384	0.138384	0.148938	0.153224	0.178047	0.178047
COC-7 CTPR-7	0.12147	0.095018	0.105509	0.10551	0.11707	0.132303	0.132304	0.15255
COC-7 TrBCSPY-7	0.42347	0.112429	0.202467	0.372191	0.37564	0.487767	0.562519	0.60636
COC-7 FPSS-7	0.33961	0.29987	0.299871	0.305655	0.305656	0.314509	0.369926	0.452638
COC-7 SPBPY-7	0.34307	0.305823	0.305823	0.310417	0.310418	0.316453	0.374518	0.45132
COC-7 HECU-7	0.26019	0.090209	0.090259	0.143654	0.226987	0.300853	0.300889	0.452112
COC-7 16	0.26681	0.098808	0.098808	0.145289	0.229097	0.316638	0.316638	0.452407
COC-7 17	0.26464	0.096066	0.096067	0.138417	0.233629	0.308468	0.308468	0.455784
COC-7 18	0.26223	0.090339	0.09034	0.136373	0.219793	0.313512	0.313512	0.448949
COC-7 CisBCSPY-7	0.26269	0.091178	0.091245	0.125136	0.217784	0.318263	0.318433	0.447916
COC-7 20-Δ/Λ	0.26388	0.075667	0.105493	0.12535	0.22402	0.311209	0.318498	0.454297
COC-7 DPAC-7	0.3589	0.092798	0.177435	0.186256	0.198239	0.331359	0.561561	0.602017
COC-7 22	0.36012	0.093213	0.181119	0.187779	0.199532	0.338088	0.56375	0.599271
COC-7 TSPPY-7	0.35361	0.051324	0.169687	0.184433	0.213767	0.316497	0.55072	0.600585
COC-7 24-Δ/Λ	0.35357	0.066243	0.167661	0.175053	0.21565	0.32479	0.552277	0.595757
COC-7 HEOB-7	0.36162	0.079672	0.2042	0.238378	0.240215	0.427284	0.516322	0.551049
COC-7 CPPY-7	0.36065	0.064209	0.199725	0.236555	0.240576	0.446411	0.506578	0.544712
COC-7 27-Δ/Λ	0.35617	0.069395	0.200352	0.233835	0.244102	0.423293	0.504444	0.543274
COC-7 28-Δ/Λ	0.35713	0.078204	0.159942	0.190596	0.248425	0.430852	0.505281	0.567527
COC-7 29-Δ/Λ	0.35986	0.056075	0.242369	0.263038	0.274904	0.414867	0.51107	0.516281
COC-7 30-Δ/Λ	0.35874	0.084022	0.229434	0.231549	0.249325	0.423668	0.499039	0.544845
COC-7 Δ/Λ-THTB-7	0.38976	0.019697	0.294037	0.332432	0.362396	0.43326	0.445248	0.590554
COC-7 32-Δ/Λ	0.39048	0.032297	0.286582	0.343802	0.359549	0.443204	0.446456	0.583886
COC-7 Δ/Λ-SHEAPR-7	0.33808	0.084485	0.115245	0.236035	0.378732	0.420499	0.422574	0.474465
COC-7 34-Δ/Λ	0.32871	0.062818	0.121535	0.170477	0.333819	0.373469	0.456894	0.498905

COC-7 35-Δ/Λ	0.33214	0.059696	0.116384	0.205111	0.337961	0.408955	0.452836	0.475919
6 DTT-7	0.1267	0.027593	0.053028	0.099251	0.099251	0.170663	0.170663	0.175634
6 TT-7	0.13356	0.027446	0.061647	0.105693	0.105693	0.179447	0.179447	0.183213
6 9	0.12665	0.032584	0.070716	0.10821	0.10821	0.165767	0.165767	0.166839
6 10	0.13034	0.029734	0.069266	0.10347	0.111876	0.157482	0.175545	0.185463
6 CTPR-7	0.10792	0.055638	0.066414	0.105217	0.105532	0.116719	0.1291	0.146709
6 TrBCSPY-7	0.42353	0.068461	0.284994	0.286752	0.295194	0.459264	0.506778	0.729791
6 FPSS-7	0.3363	0.243519	0.27806	0.300465	0.306145	0.31775	0.391678	0.465488
6 SPBPY-7	0.33937	0.252725	0.279005	0.30787	0.311227	0.322504	0.39187	0.463959
6 HECU-7	0.24073	0.07267	0.137294	0.171822	0.19944	0.242488	0.308195	0.398035
6 16	0.24754	0.086623	0.138468	0.178916	0.216621	0.244128	0.32361	0.398751
6 17	0.24536	0.081738	0.131913	0.17679	0.207088	0.248935	0.31583	0.401842
6 18	0.24247	0.079622	0.129647	0.170119	0.212601	0.234934	0.320665	0.395265
6 CisBCSPY-7	0.24275	0.084662	0.118304	0.168916	0.217106	0.232784	0.325504	0.394376
6 20-Δ/Λ	0.24334	0.096764	0.120847	0.155113	0.207416	0.239263	0.3258	0.400087
6 DPAC-7	0.3476	0.113376	0.135574	0.168826	0.262236	0.330337	0.511478	0.588663
6 22	0.35116	0.113669	0.139704	0.174506	0.267239	0.326957	0.516202	0.596251
6 TSPPY-7	0.34137	0.039424	0.116991	0.125458	0.231841	0.326382	0.531197	0.585068
6 24-Δ/Λ	0.3406	0.032242	0.102293	0.115122	0.234826	0.334159	0.533536	0.579522
6 HEOB-7	0.3219	0.003297	0.005838	0.147587	0.148142	0.394275	0.395835	0.607819
6 CPPY-7	0.31927	0.008563	0.029531	0.162746	0.162748	0.389521	0.389523	0.596801
6 27-Δ/Λ	0.31668	0.003908	0.007151	0.140706	0.158356	0.38664	0.388825	0.596964
6 28-Δ/Λ	0.31796	0.023526	0.040328	0.143131	0.176037	0.344221	0.415989	0.602093
6 29-Δ/Λ	0.32034	0.027019	0.039264	0.134446	0.171648	0.357211	0.428799	0.597533
6 30-Δ/Λ	0.31748	0.026937	0.049586	0.144577	0.154602	0.380661	0.40763	0.588631
6 Δ/Λ-THTB-7	0.36807	0.099638	0.226207	0.291403	0.295841	0.422583	0.464694	0.565946
6 32-Δ/Λ	0.36897	0.108509	0.23711	0.287616	0.290426	0.436032	0.462425	0.560316
6 Δ/Λ-SHEAPR-7	0.33384	0.103541	0.171677	0.26787	0.335419	0.403139	0.434169	0.452404
6 34-Δ/Λ	0.2918	0.112664	0.143639	0.244181	0.264714	0.326542	0.381635	0.425148
6 35-Δ/Λ	0.3275	0.115781	0.148496	0.237549	0.294499	0.420398	0.434819	0.454264
DTT-7 TT-7	0.00766	6.89E-08	0.007756	0.007756	0.007756	0.008769	0.008769	0.008769
DTT-7 9	0.01052	0.004614	0.009206	0.009318	0.009318	0.009888	0.009888	0.017319
DTT-7 10	0.01379	0.003227	0.006712	0.006712	0.012362	0.016207	0.020186	0.020186

DTT-7 CTPR-7	0.17842	0.047533	0.087653	0.087653	0.112773	0.213602	0.213602	0.318201
DTT-7 TrBCSPY-7	0.41702	0.154251	0.222531	0.243219	0.27473	0.347232	0.529736	0.779869
DTT-7 FPSS-7	0.27523	0.045638	0.045638	0.132805	0.140531	0.311476	0.311476	0.542832
DTT-7 SPBPY-7	0.27634	0.048704	0.048704	0.130219	0.144573	0.314688	0.314688	0.542129
DTT-7 HECU-7	0.23253	0.160851	0.160867	0.206577	0.206621	0.218662	0.296857	0.3247
DTT-7 16	0.23622	0.179287	0.179288	0.204455	0.204455	0.218808	0.29652	0.326951
DTT-7 17	0.2345	0.1685	0.1685	0.205921	0.205921	0.224376	0.290736	0.329345
DTT-7 18	0.22972	0.1743	0.1743	0.196368	0.196368	0.210248	0.288427	0.322704
DTT-7 CisBCSPY-7	0.22649	0.178148	0.178253	0.189695	0.189806	0.207827	0.276869	0.322066
DTT-7 20-Δ/Λ	0.22858	0.166543	0.179784	0.185905	0.201741	0.214668	0.277149	0.327924
DTT-7 DPAC-7	0.35141	0.106019	0.244447	0.244447	0.301622	0.326384	0.326384	0.655465
DTT-7 22	0.35675	0.103574	0.248425	0.248426	0.311014	0.331518	0.331518	0.663484
DTT-7 TSPPY-7	0.36654	0.164379	0.199987	0.286285	0.303806	0.361403	0.368546	0.657823
DTT-7 24-Δ/Λ	0.35822	0.147742	0.195728	0.288004	0.28844	0.34139	0.362023	0.651429
DTT-7 HEOB-7	0.28574	0.022566	0.117301	0.118074	0.179624	0.314446	0.315996	0.558845
DTT-7 CPPY-7	0.27681	0.034335	0.107547	0.107547	0.144384	0.309183	0.309184	0.547729
DTT-7 27-Δ/Λ	0.27936	0.029105	0.11056	0.117074	0.169874	0.308067	0.308853	0.548059
DTT-7 28-Δ/Λ	0.28027	0.056246	0.108042	0.153622	0.159561	0.261347	0.33675	0.551581
DTT-7 29-Δ/Λ	0.28129	0.046201	0.103156	0.108363	0.16793	0.275163	0.352035	0.54911
DTT-7 30-Δ/Λ	0.28169	0.02344	0.112227	0.138621	0.199854	0.296908	0.324426	0.538278
DTT-7 Δ/Λ-THTB-7	0.34329	0.135576	0.139545	0.172786	0.174935	0.256345	0.53687	0.610486
DTT-7 32-Δ/Λ	0.34427	0.12939	0.152071	0.172957	0.184125	0.263583	0.535508	0.608056
DTT-7 Δ/Λ-SHEAPR-7	0.3697	0.174084	0.241333	0.293397	0.350162	0.388765	0.463791	0.541535
DTT-7 34-Δ/Λ	0.3135	0.085466	0.22257	0.294905	0.300744	0.333357	0.369951	0.453554
DTT-7 35-Δ/Λ	0.36242	0.180507	0.231426	0.261263	0.350041	0.372353	0.496277	0.507528
TT-7 9	0.01143	0.003962	0.003962	0.004575	0.008511	0.015825	0.015825	0.017
TT-7 10	0.01314	0.003164	0.008514	0.010656	0.010656	0.015075	0.015075	0.021068
TT-7 CTPR-7	0.18408	0.055567	0.094625	0.094625	0.112493	0.219701	0.219701	0.327133
TT-7 TrBCSPY-7	0.41469	0.154595	0.230044	0.248084	0.267693	0.355179	0.522673	0.770991
TT-7 FPSS-7	0.27132	0.052383	0.052383	0.140602	0.141624	0.302854	0.302854	0.535295
TT-7 SPBPY-7	0.27238	0.054545	0.054545	0.138979	0.144583	0.30605	0.30605	0.53465
TT-7 HECU-7	0.23328	0.154664	0.154678	0.21399	0.214034	0.218175	0.305042	0.317529
TT-7 16	0.23674	0.17324	0.17324	0.211641	0.211641	0.218209	0.304594	0.319892

TT-7 17	0.23507	0.162265	0.162265	0.213191	0.213191	0.223835	0.298871	0.32223
TT-7 18	0.2302	0.168095	0.168095	0.203605	0.203605	0.209693	0.29655	0.315599
TT-7 CisBCSPY-7	0.22677	0.171828	0.171926	0.196797	0.196908	0.207249	0.284976	0.314983
TT-7 20-Δ/Λ	0.22889	0.159814	0.1736	0.193336	0.208647	0.214124	0.285277	0.320811
TT-7 DPAC-7	0.34964	0.106554	0.251286	0.251286	0.309816	0.319333	0.319333	0.646667
TT-7 22	0.35502	0.104119	0.255312	0.255313	0.319211	0.324472	0.324472	0.65469
TT-7 TSPPY-7	0.36495	0.164426	0.206805	0.278936	0.31071	0.361823	0.368833	0.648999
TT-7 24-Δ/Λ	0.35647	0.147828	0.202371	0.281035	0.294784	0.348944	0.355207	0.642568
TT-7 HEOB-7	0.28223	0.023055	0.121738	0.12249	0.187834	0.307051	0.308597	0.549949
TT-7 CPPY-7	0.27296	0.034809	0.110838	0.110838	0.15259	0.301771	0.301772	0.538834
TT-7 27-Δ/Λ	0.2758	0.029564	0.11534	0.120872	0.178051	0.300699	0.301409	0.539181
TT-7 28-Δ/Λ	0.27674	0.056718	0.11252	0.161897	0.163149	0.254024	0.329316	0.542612
TT-7 29-Δ/Λ	0.27763	0.046433	0.105778	0.113543	0.175992	0.267701	0.344671	0.540255
TT-7 30-Δ/Λ	0.27835	0.022921	0.116703	0.142931	0.208127	0.289569	0.317007	0.529273
TT-7 Δ/Λ-THTB-7	0.34028	0.135646	0.138265	0.167719	0.181002	0.263949	0.529563	0.601678
TT-7 32-Δ/Λ	0.34122	0.129319	0.150279	0.176772	0.181247	0.271076	0.528281	0.599209
TT-7 Δ/Λ-SHEAPR-7	0.36996	0.170253	0.240495	0.301104	0.344265	0.394099	0.454915	0.547545
TT-7 34-Δ/Λ	0.31384	0.084451	0.221824	0.302779	0.304712	0.326803	0.36099	0.459883
TT-7 35-Δ/Λ	0.36229	0.175058	0.230667	0.26901	0.344178	0.377893	0.487402	0.513539
9 10	0.01213	0.004078	0.008688	0.009429	0.01097	0.012008	0.014074	0.019637
9 CTPR-7	0.17882	0.055757	0.079371	0.102198	0.115563	0.207022	0.22228	0.313641
9 TrBCSPY-7	0.4083	0.156797	0.215344	0.232703	0.268317	0.339381	0.521027	0.763697
9 FPSS-7	0.27041	0.037354	0.051426	0.129536	0.139001	0.294994	0.31553	0.533993
9 SPBPY-7	0.27148	0.041898	0.052843	0.12695	0.143035	0.298217	0.318537	0.533295
9 HECU-7	0.22816	0.148625	0.158333	0.198111	0.213246	0.216231	0.292532	0.316544
9 16	0.23171	0.167295	0.176529	0.196302	0.210519	0.216364	0.292184	0.318809
9 17	0.23006	0.15618	0.166004	0.197644	0.212185	0.221952	0.28642	0.321184
9 18	0.2252	0.162024	0.171754	0.188124	0.202567	0.207824	0.284111	0.31454
9 CisBCSPY-7	0.22189	0.165756	0.175742	0.181723	0.195603	0.205422	0.272573	0.313884
9 20-Δ/Λ	0.22398	0.164779	0.167647	0.177475	0.207401	0.212075	0.27224	0.319853
9 DPAC-7	0.34261	0.110813	0.235641	0.235641	0.292704	0.319734	0.319734	0.6389
9 22	0.34795	0.108382	0.239666	0.239666	0.302115	0.324871	0.324871	0.646931
9 TSPPY-7	0.35796	0.167419	0.191979	0.279994	0.294313	0.353566	0.361483	0.641363

9 24-Δ/Λ	0.34949	0.150729	0.18745	0.27849	0.281798	0.333414	0.354803	0.634929
9 HEOB-7	0.27667	0.027088	0.107423	0.108199	0.170364	0.306432	0.307981	0.542281
9 CPPY-7	0.26788	0.03881	0.097711	0.097712	0.135063	0.301107	0.301108	0.531185
9 27-Δ/Λ	0.27035	0.033586	0.100703	0.10721	0.16059	0.300119	0.300883	0.531496
9 28-Δ/Λ	0.27118	0.059543	0.098164	0.1444	0.149734	0.252939	0.328849	0.534967
9 29-Δ/Λ	0.27241	0.049007	0.093404	0.098681	0.158735	0.267233	0.344032	0.532577
9 30-Δ/Λ	0.2723	0.019191	0.102388	0.128722	0.190662	0.288426	0.316178	0.521622
9 Δ/Λ-THTB-7	0.33494	0.13176	0.136143	0.166687	0.168152	0.247874	0.528036	0.59419
9 32-Δ/Λ	0.33589	0.129545	0.144737	0.167428	0.177064	0.255217	0.526666	0.591719
9 Δ/Λ-SHEAPR-7	0.36283	0.174235	0.243248	0.2909	0.3438	0.378179	0.447371	0.534805
9 34-Δ/Λ	0.30644	0.088381	0.223052	0.290416	0.291947	0.325933	0.353122	0.446399
9 35-Δ/Λ	0.35524	0.178609	0.233607	0.258062	0.343843	0.361852	0.479821	0.500881
10 CTPR-7	0.17847	0.062553	0.093944	0.093944	0.117174	0.217655	0.217655	0.304823
10 TrBCSPY-7	0.40986	0.154771	0.214306	0.238314	0.279244	0.345314	0.512458	0.767731
10 FPSS-7	0.2648	0.041095	0.041095	0.122432	0.139275	0.297572	0.297572	0.525317
10 SPBPY-7	0.26575	0.043001	0.043001	0.119841	0.143313	0.300463	0.300463	0.524617
10 HECU-7	0.22211	0.140694	0.14071	0.203938	0.203982	0.214978	0.284152	0.309171
10 16	0.22536	0.159165	0.159165	0.201282	0.201282	0.215118	0.283809	0.311431
10 17	0.22389	0.148347	0.148347	0.202929	0.202929	0.220724	0.278056	0.313791
10 18	0.21886	0.154156	0.154156	0.193291	0.193291	0.206607	0.275759	0.307128
10 CisBCSPY-7	0.21544	0.158005	0.15811	0.186339	0.186451	0.204244	0.26425	0.30643
10 20-Δ/Λ	0.21772	0.146398	0.159695	0.183075	0.198187	0.211108	0.264567	0.312278
10 DPAC-7	0.34557	0.108007	0.236293	0.246785	0.301553	0.310122	0.330298	0.641325
10 22	0.35093	0.105577	0.240192	0.250945	0.310958	0.315254	0.335441	0.649349
10 TSPPY-7	0.36057	0.16415	0.191683	0.290214	0.305415	0.352407	0.359023	0.64483
10 24-Δ/Λ	0.35211	0.147536	0.187801	0.28922	0.292056	0.339107	0.345694	0.638286
10 HEOB-7	0.279	0.024327	0.112893	0.113375	0.17932	0.297151	0.318542	0.544777
10 CPPY-7	0.26998	0.035995	0.099548	0.105347	0.144063	0.291807	0.311699	0.533704
10 27-Δ/Λ	0.27244	0.030904	0.106079	0.110272	0.169322	0.290814	0.311436	0.533815
10 28-Δ/Λ	0.27325	0.056516	0.103256	0.153629	0.156726	0.263273	0.319485	0.537809
10 29-Δ/Λ	0.27387	0.046206	0.09364	0.10337	0.166555	0.277791	0.334727	0.535254
10 30-Δ/Λ	0.27475	0.021414	0.107393	0.134748	0.199906	0.298644	0.307198	0.524175
10 Δ/Λ-THTB-7	0.33466	0.122961	0.134927	0.163336	0.178999	0.253658	0.519463	0.59813

10 32-Δ/Λ	0.33538	0.12855	0.134882	0.163129	0.18783	0.260657	0.518122	0.595278
10 Δ/Λ-SHEAPR-7	0.36301	0.18131	0.238906	0.28057	0.336183	0.385252	0.450077	0.538286
10 34-Δ/Λ	0.30549	0.09296	0.219428	0.282127	0.294245	0.318409	0.355242	0.450238
10 35-Δ/Λ	0.35567	0.186939	0.229437	0.248713	0.335932	0.369242	0.482443	0.504128
CTPR-7 TrBCSPY-7	0.41932	0.074439	0.305055	0.365741	0.395039	0.413611	0.552152	0.605349
CTPR-7 FPSS-7	0.34168	0.175118	0.203556	0.203556	0.264021	0.38753	0.38753	0.577605
CTPR-7 SPBPY-7	0.34456	0.17683	0.213799	0.213799	0.268902	0.390002	0.390002	0.57607
CTPR-7 HECU-7	0.24122	0.001131	0.200271	0.200311	0.200858	0.200897	0.350583	0.351377
CTPR-7 16	0.24895	3.50E-08	0.215515	0.215515	0.215515	0.215515	0.352175	0.352175
CTPR-7 17	0.24749	0.006404	0.208286	0.208286	0.210111	0.210111	0.355112	0.357141
CTPR-7 18	0.24407	0.009195	0.208711	0.208711	0.212815	0.212815	0.342698	0.348936
CTPR-7 CisBCSPY-7	0.24599	0.020783	0.213547	0.213585	0.217622	0.217802	0.340386	0.348213
CTPR-7 20-Δ/Λ	0.24731	0.025844	0.195519	0.211883	0.218066	0.225639	0.347042	0.354175
CTPR-7 DPAC-7	0.343	0.168097	0.183039	0.187117	0.18757	0.450149	0.45608	0.530023
CTPR-7 22	0.34564	0.171599	0.184181	0.191242	0.192356	0.446974	0.463255	0.533788
CTPR-7 TSPPY-7	0.32992	0.097458	0.097458	0.170486	0.170486	0.445994	0.445994	0.535707
CTPR-7 24-Δ/Λ	0.33225	0.090265	0.109419	0.159723	0.177711	0.440732	0.454597	0.542767
CTPR-7 HEOB-7	0.35353	0.051024	0.108915	0.13232	0.280499	0.355776	0.497723	0.624441
CTPR-7 CPPY-7	0.3512	0.060386	0.105859	0.133887	0.298693	0.35154	0.491044	0.613769
CTPR-7 27-Δ/Λ	0.348	0.059398	0.105717	0.137611	0.275575	0.349156	0.490385	0.613004
CTPR-7 28-Δ/Λ	0.3486	0.067193	0.092658	0.141091	0.280569	0.303567	0.516805	0.61617
CTPR-7 29-Δ/Λ	0.352	0.067332	0.146642	0.157132	0.268665	0.39167	0.459766	0.616149
CTPR-7 30-Δ/Λ	0.34896	0.043857	0.12473	0.137051	0.280406	0.365296	0.485941	0.606575
CTPR-7 Δ/Λ-THTB-7	0.37521	0.112761	0.251131	0.384099	0.398189	0.405304	0.449053	0.487534
CTPR-7 32-Δ/Λ	0.37571	0.114333	0.261761	0.384084	0.396499	0.396795	0.440474	0.500325
CTPR-7 Δ/Λ-SHEAPR-7	0.33664	0.007113	0.192442	0.213104	0.27237	0.396072	0.432455	0.54102
CTPR-7 34-Δ/Λ	0.31197	0.096782	0.153012	0.229325	0.245894	0.345292	0.439579	0.472238
CTPR-7 35-Δ/Λ	0.32746	0.031567	0.165003	0.184582	0.303964	0.353392	0.436696	0.529472
TrBCSPY-7 FPSS-7	0.31468	0.000116	0.138221	0.138229	0.327398	0.327536	0.469277	0.469327
TrBCSPY-7 SPBPY-7	0.31441	0.000116	0.139486	0.139497	0.32695	0.327085	0.468584	0.468636
TrBCSPY-7 HECU-7	0.35718	0.149674	0.154672	0.157005	0.189329	0.238423	0.501736	0.691115
TrBCSPY-7 16	0.35536	0.152571	0.153977	0.15653	0.185646	0.242787	0.498466	0.685998
TrBCSPY-7 17	0.35568	0.150601	0.15137	0.16035	0.184722	0.242753	0.499726	0.686657

TrBCSPY-7 18	0.35396	0.150141	0.152298	0.160163	0.190894	0.244767	0.490507	0.684585
TrBCSPY-7 CisBCSPY-7	0.35187	0.14474	0.151139	0.168174	0.192135	0.249915	0.482304	0.680163
TrBCSPY-7 20-Δ	0.35017	0.15287	0.15973	0.160089	0.191468	0.250587	0.477565	0.675498
TrBCSPY-7 DPAC-7	0.13882	0.031229	0.052537	0.105801	0.123322	0.155488	0.188764	0.212029
TrBCSPY-7 22	0.13695	0.02862	0.05417	0.109891	0.120528	0.149377	0.188809	0.207299
TrBCSPY-7 TSPPY-7	0.10474	0.033132	0.033458	0.035272	0.035384	0.122511	0.122636	0.205027
TrBCSPY-7 24-Δ/Λ	0.10271	0.022559	0.028528	0.042628	0.045839	0.107372	0.132754	0.19864
TrBCSPY-7 HEOB-7	0.17214	0.026289	0.108108	0.155745	0.177747	0.200213	0.201449	0.241941
TrBCSPY-7 CPPY-7	0.18374	0.027049	0.121767	0.174401	0.174935	0.209391	0.229043	0.251869
TrBCSPY-7 27-Δ/Λ	0.17723	0.021121	0.109275	0.155595	0.179818	0.20488	0.210521	0.254235
TrBCSPY-7 28-Δ/Λ	0.17055	0.061146	0.118601	0.136234	0.151234	0.181732	0.226219	0.24531
TrBCSPY-7 29-Δ/Λ	0.16453	0.03439	0.130825	0.136435	0.148152	0.172762	0.19918	0.247205
TrBCSPY-7 30-Δ/Λ	0.17483	0.055588	0.11114	0.148579	0.184906	0.189389	0.192015	0.263646
TrBCSPY-7 Δ/Λ-THTB-7	0.11262	9.31E-05	0.067133	0.067192	0.105089	0.105204	0.169723	0.169851
TrBCSPY-7 32-Δ/Λ	0.11545	9.24E-05	0.079475	0.079544	0.09829	0.098391	0.175022	0.175146
TrBCSPY-7 Δ/Λ-SHEAPR-7	0.11665	9.79E-05	0.077509	0.077564	0.127649	0.127828	0.159	0.159117
TrBCSPY-7 34-Δ/Λ	0.17208	9.83E-05	0.125696	0.125837	0.144166	0.144207	0.258837	0.258958
TrBCSPY-7 35-Δ/Λ	0.12273	0.039435	0.041971	0.096332	0.125472	0.134837	0.153339	0.188178
FPSS-7 SPBPY-7	0.0073	9.66E-08	0.003322	0.003322	0.007593	0.007593	0.01085	0.01085
FPSS-7 HECU-7	0.18093	0.05879	0.145273	0.171825	0.171868	0.203576	0.228103	0.228159
FPSS-7 16	0.17907	0.057111	0.143112	0.169945	0.169945	0.19949	0.227126	0.227126
FPSS-7 17	0.17818	0.064182	0.138747	0.170888	0.170888	0.198539	0.224777	0.224777
FPSS-7 18	0.1754	0.049444	0.135888	0.161501	0.161501	0.204707	0.224028	0.224028
FPSS-7 CisBCSPY-7	0.17087	0.047216	0.124443	0.154476	0.154584	0.205555	0.219721	0.219931
FPSS-7 20-Δ/Λ	0.17093	0.055141	0.12591	0.149863	0.167073	0.200434	0.213571	0.222403
FPSS-7 DPAC-7	0.32379	0.156524	0.22973	0.232044	0.267198	0.349428	0.356736	0.531051
FPSS-7 22	0.32758	0.158017	0.227694	0.228032	0.276683	0.357708	0.361691	0.535743
FPSS-7 TSPPY-7	0.32794	0.12225	0.16027	0.207616	0.319594	0.326684	0.354213	0.578583
FPSS-7 24-Δ/Λ	0.31922	0.126357	0.153224	0.203453	0.307057	0.321713	0.346079	0.561188
FPSS-7 HEOB-7	0.25255	0.140338	0.148796	0.165063	0.221476	0.246346	0.3505	0.380516
FPSS-7 CPPY-7	0.24566	0.11395	0.152075	0.172579	0.2283	0.234711	0.345742	0.360279
FPSS-7 27-Δ/Λ	0.24844	0.132053	0.138104	0.172158	0.229769	0.235213	0.344395	0.373082
FPSS-7 28-Δ/Λ	0.23743	0.122046	0.147724	0.173939	0.202411	0.241121	0.295087	0.376091

FPSS-7 29-Δ/Λ	0.22837	0.124524	0.13309	0.138938	0.189215	0.237635	0.311856	0.350735
FPSS-7 30-Δ/Λ	0.24423	0.135442	0.150687	0.170913	0.211378	0.224521	0.331938	0.376833
FPSS-7 Δ/Λ-THTB-7	0.20468	1.50E-07	0.073616	0.073616	0.226159	0.226159	0.300111	0.300112
FPSS-7 32-Δ/Λ	0.20385	1.95E-07	0.062376	0.062376	0.23361	0.23361	0.294913	0.294913
FPSS-7 Δ/Λ-SHEAPR-7	0.29914	1.01E-07	0.223272	0.223272	0.310218	0.310218	0.408796	0.408796
FPSS-7 34-Δ/Λ	0.2769	0.052285	0.094441	0.118139	0.138452	0.227655	0.422857	0.511168
FPSS-7 35-Δ/Λ	0.30451	0.048351	0.224073	0.248462	0.30018	0.312894	0.403361	0.429059
SPBPY-7 HECU-7	0.18086	0.053925	0.147045	0.172712	0.172753	0.20204	0.227958	0.228014
SPBPY-7 16	0.17846	0.052265	0.144902	0.16965	0.16965	0.197971	0.226337	0.226337
SPBPY-7 17	0.1778	0.059334	0.140535	0.17104	0.17104	0.197018	0.224393	0.224394
SPBPY-7 18	0.17495	0.044644	0.137725	0.161489	0.16149	0.203235	0.223421	0.223421
SPBPY-7 CisBCSPY-7	0.17023	0.042466	0.126331	0.153902	0.154009	0.204132	0.218996	0.219208
SPBPY-7 20-Δ/Λ	0.17043	0.050406	0.127774	0.150653	0.165909	0.198998	0.213402	0.221583
SPBPY-7 DPAC-7	0.32451	0.157565	0.231917	0.232869	0.271239	0.347143	0.357377	0.531509
SPBPY-7 22	0.3283	0.159134	0.227564	0.231181	0.280696	0.355427	0.362306	0.536216
SPBPY-7 TSPPY-7	0.3278	0.122774	0.162145	0.207207	0.319991	0.325457	0.352942	0.578786
SPBPY-7 24-Δ/Λ	0.31906	0.126667	0.155334	0.2031	0.30616	0.321649	0.344717	0.561367
SPBPY-7 HEOB-7	0.25264	0.13814	0.153606	0.168087	0.221717	0.244423	0.348867	0.381127
SPBPY-7 CPPY-7	0.24565	0.119165	0.149541	0.175496	0.228625	0.232801	0.344114	0.360785
SPBPY-7 27-Δ/Λ	0.24848	0.129741	0.142567	0.17518	0.230001	0.23307	0.342888	0.373571
SPBPY-7 28-Δ/Λ	0.23752	0.127883	0.150651	0.172407	0.202603	0.239253	0.292999	0.376819
SPBPY-7 29-Δ/Λ	0.2283	0.122267	0.136144	0.141878	0.189413	0.235819	0.310398	0.351303
SPBPY-7 30-Δ/Λ	0.24428	0.138607	0.148888	0.176286	0.211516	0.222552	0.329285	0.377583
SPBPY-7 Δ/Λ-THTB-7	0.2045	1.19E-07	0.074786	0.074786	0.225919	0.22592	0.299564	0.299564
SPBPY-7 32-Δ/Λ	0.20337	2.09E-07	0.063858	0.063858	0.232885	0.232885	0.294024	0.294025
SPBPY-7 Δ/Λ-SHEAPR-7	0.30023	7.37E-08	0.226259	0.226259	0.313924	0.313924	0.407102	0.407102
SPBPY-7 34-Δ/Λ	0.27635	0.051037	0.092514	0.126558	0.140285	0.226292	0.419645	0.510291
SPBPY-7 35-Δ/Λ	0.30572	0.048344	0.226872	0.251494	0.304905	0.316417	0.40133	0.427857
HECU-7 16	0.01414	0.000935	0.001006	0.001493	0.018494	0.018511	0.018839	0.018847
HECU-7 17	0.00822	0.003564	0.005043	0.006893	0.00789	0.007918	0.011448	0.011467
HECU-7 18	0.01258	0.003268	0.007217	0.007276	0.013583	0.013604	0.017625	0.017635
HECU-7 CisBCSPY-7	0.01963	0.004872	0.008695	0.017995	0.018006	0.01813	0.028449	0.028505
HECU-7 20-Δ/Λ	0.01979	0.001545	0.006497	0.015542	0.020167	0.023172	0.023283	0.031153

HECU-7 DPAC-7	0.34292	0.084063	0.126339	0.18868	0.281652	0.305686	0.534844	0.552897
HECU-7 22	0.34657	0.084528	0.13464	0.186416	0.279785	0.310349	0.539888	0.560941
HECU-7 TSPPY-7	0.33826	0.101197	0.164988	0.181788	0.194569	0.346055	0.502824	0.565678
HECU-7 24-Δ/Λ	0.33248	0.087473	0.142707	0.189616	0.189971	0.329388	0.503585	0.558235
HECU-7 HEOB-7	0.29975	0.028546	0.074895	0.163877	0.218863	0.255599	0.459247	0.521092
HECU-7 CPPY-7	0.29812	0.032775	0.0885	0.143937	0.225978	0.272261	0.448205	0.51617
HECU-7 27-Δ/Λ	0.29512	0.037984	0.071236	0.153958	0.22556	0.250274	0.446721	0.51612
HECU-7 28-Δ/Λ	0.28638	0.021392	0.090514	0.1652	0.200274	0.273978	0.455903	0.463827
HECU-7 29-Δ/Λ	0.28186	0.032414	0.055066	0.134228	0.191251	0.243021	0.451491	0.484269
HECU-7 30-Δ/Λ	0.28733	0.011789	0.086418	0.164003	0.190022	0.256404	0.440225	0.497753
HECU-7 Δ/Λ-THTB-7	0.26952	0.051405	0.071211	0.102077	0.134003	0.196734	0.399049	0.523893
HECU-7 32-Δ/Λ	0.26825	0.059191	0.062603	0.094326	0.123286	0.194504	0.407175	0.51822
HECU-7 Δ/Λ-SHEAPR-7	0.29481	0.000974	0.156953	0.157108	0.371286	0.372531	0.374996	0.376606
HECU-7 34-Δ/Λ	0.21366	0.000988	0.094531	0.094648	0.273525	0.274268	0.274827	0.275851
HECU-7 35-Δ/Λ	0.29744	0.03466	0.159402	0.169824	0.338686	0.365406	0.392724	0.401728
16 17	0.00808	0.002611	0.005419	0.006031	0.007098	0.007099	0.011904	0.011905
16 18	0.00766	0.004295	0.006538	0.006539	0.00819	0.008618	0.009085	0.009085
16 CisBCSPY-7	0.01267	0.005981	0.007881	0.008045	0.010014	0.015857	0.015967	0.018826
16 20-Δ/Λ	0.01678	0.000964	0.004365	0.00702	0.012682	0.023406	0.023848	0.024993
16 DPAC-7	0.34269	0.084483	0.14251	0.188154	0.280538	0.314259	0.532824	0.54575
16 22	0.34634	0.08473	0.150499	0.185927	0.278671	0.318913	0.537838	0.553751
16 TSPPY-7	0.33731	0.100444	0.164707	0.18136	0.194242	0.353137	0.500322	0.559965
16 24-Δ/Λ	0.3314	0.086738	0.143395	0.189176	0.189451	0.33655	0.50061	0.552411
16 HEOB-7	0.29853	0.031714	0.089732	0.175994	0.21703	0.253651	0.45342	0.516517
16 CPPY-7	0.2967	0.036592	0.098208	0.156532	0.224015	0.270133	0.442513	0.511627
16 27-Δ/Λ	0.29374	0.041219	0.08452	0.165791	0.223755	0.248299	0.440663	0.511626
16 28-Δ/Λ	0.28519	0.021589	0.101515	0.178066	0.19864	0.272411	0.45008	0.458951
16 29-Δ/Λ	0.28036	0.030813	0.066104	0.147309	0.189506	0.24104	0.445908	0.479957
16 30-Δ/Λ	0.28619	0.015863	0.103184	0.176496	0.188393	0.254759	0.434477	0.492067
16 Δ/Λ-THTB-7	0.26763	0.055353	0.079975	0.101106	0.132394	0.192728	0.396197	0.519667
16 32-Δ/Λ	0.26587	0.060282	0.068347	0.093214	0.121583	0.190525	0.403772	0.513523
16 Δ/Λ-SHEAPR-7	0.29384	1.13E-07	0.157059	0.15706	0.366821	0.366821	0.378103	0.378103
16 34-Δ/Λ	0.21274	3.37E-08	0.09464	0.09464	0.269742	0.269742	0.276913	0.276913

16 35-Δ/Λ	0.29609	0.035232	0.159402	0.170002	0.332258	0.367607	0.394214	0.396486
17 18	0.00863	0.002195	0.005884	0.005885	0.006871	0.010045	0.010045	0.014072
17 CisBCSPY-7	0.01463	0.008516	0.010113	0.010273	0.012871	0.015509	0.020094	0.020188
17 20-Δ/Λ	0.01595	0.002638	0.010761	0.012436	0.013885	0.019398	0.019795	0.023314
17 DPAC-7	0.34319	0.085137	0.135963	0.185383	0.286824	0.311551	0.533711	0.54788
17 22	0.34684	0.085318	0.144037	0.183239	0.284974	0.316224	0.538726	0.55592
17 TSPPY-7	0.33756	0.100575	0.163736	0.177713	0.198303	0.351512	0.501431	0.561084
17 24-Δ/Λ	0.33172	0.086905	0.142027	0.185772	0.193676	0.334845	0.501904	0.553602
17 HEOB-7	0.29886	0.032709	0.084419	0.170568	0.223955	0.249553	0.454651	0.518466
17 CPPY-7	0.29709	0.037566	0.094856	0.150699	0.230953	0.266043	0.443639	0.513583
17 27-Δ/Λ	0.29419	0.042219	0.079794	0.160544	0.230655	0.244149	0.442004	0.513533
17 28-Δ/Λ	0.28535	0.022119	0.097714	0.171951	0.205265	0.268568	0.45118	0.46104
17 29-Δ/Λ	0.28063	0.03078	0.062067	0.14115	0.196263	0.236899	0.446938	0.481814
17 30-Δ/Λ	0.28638	0.016957	0.097169	0.170776	0.195259	0.250676	0.435622	0.494456
17 Δ/Λ-THTB-7	0.2676	0.052529	0.076739	0.106233	0.128579	0.191792	0.397447	0.519668
17 32-Δ/Λ	0.26611	0.058583	0.067067	0.098832	0.117628	0.189588	0.405218	0.513846
17 Δ/Λ-SHEAPR-7	0.29443	0.00598	0.157632	0.157812	0.366888	0.368095	0.377214	0.380303
17 34-Δ/Λ	0.21324	0.006005	0.095138	0.095366	0.269235	0.270754	0.276232	0.279291
17 35-Δ/Λ	0.29679	0.030349	0.160004	0.171346	0.333829	0.369338	0.393919	0.397108
18 CisBCSPY-7	0.00769	0.001451	0.001631	0.005252	0.005458	0.010691	0.010853	0.010927
18 20-Δ/Λ	0.01365	0.004519	0.006562	0.008105	0.016001	0.017044	0.017455	0.018016
18 DPAC-7	0.33965	0.083976	0.143632	0.184101	0.273153	0.314331	0.524736	0.545165
18 22	0.34338	0.08452	0.151914	0.18203	0.271275	0.31899	0.529765	0.553182
18 TSPPY-7	0.33529	0.101764	0.172959	0.176082	0.18925	0.354102	0.492057	0.55885
18 24-Δ/Λ	0.32923	0.087985	0.151485	0.183734	0.184119	0.337455	0.492511	0.551308
18 HEOB-7	0.29442	0.027768	0.082263	0.174523	0.209527	0.247212	0.452217	0.50919
18 CPPY-7	0.29237	0.031958	0.089171	0.154821	0.21661	0.263625	0.441265	0.504272
18 27-Δ/Λ	0.28956	0.037133	0.076413	0.164443	0.216268	0.24176	0.439527	0.504231
18 28-Δ/Λ	0.28111	0.021481	0.092824	0.176102	0.191343	0.266458	0.448807	0.451718
18 29-Δ/Λ	0.27635	0.0329	0.057486	0.145389	0.182194	0.234485	0.444616	0.472456
18 30-Δ/Λ	0.28222	0.010612	0.096899	0.174804	0.18078	0.24856	0.433146	0.485179
18 Δ/Λ-THTB-7	0.26549	0.062351	0.079811	0.095635	0.12629	0.198078	0.388044	0.517912
18 32-Δ/Λ	0.26388	0.068184	0.069302	0.087268	0.115296	0.195936	0.395764	0.511897

18 Δ/Λ-SHEAPR-7	0.29388	0.007932	0.155831	0.156908	0.365197	0.368651	0.375354	0.381358
18 34-Δ/Λ	0.21284	0.008048	0.093804	0.094484	0.267643	0.267757	0.278441	0.280094
18 35-Δ/Λ	0.29573	0.02869	0.159164	0.167963	0.340869	0.370635	0.384902	0.395122
CisBCSPY-7 20-Δ/Λ	0.01323	0.004597	0.006112	0.009069	0.014415	0.015288	0.017016	0.018823
CisBCSPY-7 DPAC-7	0.33762	0.084166	0.15422	0.178495	0.271002	0.319323	0.517294	0.540551
CisBCSPY-7 22	0.34141	0.084763	0.162516	0.176671	0.269129	0.32399	0.522306	0.548569
CisBCSPY-7 TSPPY-7	0.33321	0.102323	0.168708	0.17922	0.187941	0.359297	0.484221	0.554327
CisBCSPY-7 24-Δ/Λ	0.32697	0.088515	0.158269	0.176564	0.182308	0.34262	0.484509	0.546754
CisBCSPY-7 HEOB-7	0.29052	0.02737	0.087299	0.179493	0.207148	0.236875	0.44765	0.5008
CisBCSPY-7 CPPY-7	0.28814	0.031479	0.09015	0.159747	0.214194	0.253054	0.436708	0.495896
CisBCSPY-7 27-Δ/Λ	0.28565	0.036739	0.080248	0.169638	0.213902	0.231301	0.43516	0.495986
CisBCSPY-7 28-Δ/Λ	0.27728	0.021829	0.094733	0.18103	0.189083	0.257191	0.443405	0.444354
CisBCSPY-7 29-Δ/Λ	0.27227	0.03335	0.06037	0.150335	0.179906	0.223979	0.440046	0.464115
CisBCSPY-7 30-Δ/Λ	0.27841	0.009849	0.103541	0.178468	0.179702	0.238744	0.42849	0.476461
CisBCSPY-7 Δ/Λ-THTB-7	0.26254	0.071045	0.084922	0.094144	0.116937	0.199106	0.380099	0.51339
CisBCSPY-7 32-Δ/Λ	0.26082	0.074364	0.075974	0.08566	0.105551	0.197034	0.387573	0.507305
CisBCSPY-7 Δ/Λ-SHEAPR-7	0.29369	0.018377	0.155299	0.156974	0.360661	0.363686	0.378211	0.386384
CisBCSPY-7 34-Δ/Λ	0.2128	0.018583	0.09345	0.094549	0.262722	0.263186	0.281958	0.284903
CisBCSPY-7 35-Δ/Λ	0.29497	0.021318	0.159205	0.167319	0.343419	0.375738	0.378984	0.390502
20-Δ 20-Λ	0.02379	0.001975	0.012257	0.021467	0.021467	0.028643	0.032134	0.032134
20-Δ/Λ DPAC-7	0.33781	0.077159	0.121097	0.186995	0.279999	0.289352	0.537592	0.540472
20-Δ/Λ 22	0.34132	0.077469	0.128741	0.184266	0.278105	0.293957	0.542645	0.548423
20-Δ/Λ TSPPY-7	0.3317	0.102823	0.169261	0.183264	0.190889	0.35979	0.478618	0.549924
20-Δ 24-Δ	0.32584	0.089054	0.148869	0.186314	0.190976	0.343095	0.479507	0.542492
20-Δ 24-Λ	0.33028	0.086666	0.16128	0.162715	0.188541	0.342368	0.497307	0.550941
20-Δ/Λ HEOB-7	0.29216	0.029482	0.099071	0.180293	0.217512	0.24771	0.443867	0.498636
20-Δ/Λ CPPY-7	0.28994	0.033764	0.103388	0.160496	0.224107	0.263272	0.432816	0.493966
20-Δ 27-Δ	0.28782	0.042889	0.062623	0.165674	0.218961	0.2226	0.438685	0.506678
20-Δ 27-Λ	0.2872	0.03889	0.092676	0.170141	0.224058	0.241643	0.431376	0.4936
20-Δ 28-Δ	0.27847	0.021907	0.078763	0.175856	0.195008	0.246276	0.447922	0.453759
20-Δ 28-Λ	0.27922	0.022792	0.107847	0.18182	0.197804	0.269246	0.440295	0.441637
20-Δ 29-Δ	0.27342	0.033588	0.073141	0.15128	0.189013	0.234095	0.436176	0.461718
20-Δ 29-Λ	0.27451	0.029782	0.043291	0.145401	0.185699	0.215634	0.443748	0.475059

20-Δ 30-Δ	0.2805	0.011709	0.114216	0.180587	0.189708	0.250203	0.424639	0.475442
20-Δ 30-Λ	0.27967	0.018378	0.084998	0.175157	0.182412	0.228594	0.432349	0.486613
20-Δ Δ-THTB-7	0.2608	0.064287	0.085676	0.097724	0.128814	0.196897	0.375572	0.508599
20-Δ Λ-THTB-7	0.26505	0.071747	0.084045	0.101655	0.106962	0.19105	0.392704	0.516776
20-Δ 32-Δ	0.25914	0.06761	0.074621	0.090545	0.116803	0.194844	0.383579	0.502991
20-Δ 32-Λ	0.26358	0.076131	0.078901	0.093037	0.096462	0.188932	0.399909	0.510586
20-Δ Δ-SHEAPR-7	0.28774	0.020384	0.150471	0.158227	0.351835	0.355348	0.364873	0.385058
20-Δ Λ-SHEAPR-7	0.30083	0.026646	0.15728	0.161683	0.364786	0.379856	0.388768	0.391678
20-Δ 34-Δ	0.20671	0.021381	0.088017	0.095812	0.251381	0.257603	0.268303	0.283138
20-Δ 34-Λ	0.21995	0.025944	0.094476	0.099794	0.267097	0.278641	0.290999	0.292263
20-Δ 35-Δ	0.28985	0.035628	0.160437	0.163813	0.330187	0.368761	0.374612	0.385874
20-Δ 35-Λ	0.30176	0.008589	0.159761	0.173843	0.354279	0.380399	0.394554	0.394601
20-Λ 24-Δ	0.33028	0.086666	0.16128	0.162715	0.188541	0.342368	0.497307	0.550941
20-Λ 24-Λ	0.32584	0.089054	0.148869	0.186314	0.190976	0.343095	0.479507	0.542492
20-Λ 27-Δ	0.2872	0.03889	0.092676	0.170141	0.224058	0.241643	0.431376	0.4936
20-Λ 27-Λ	0.28782	0.042889	0.062623	0.165674	0.218961	0.2226	0.438685	0.506678
20-Λ 28-Δ	0.27922	0.022792	0.107847	0.18182	0.197804	0.269246	0.440295	0.441637
20-Λ 28-Λ	0.27847	0.021907	0.078763	0.175856	0.195008	0.246276	0.447922	0.453759
20-Λ 29-Δ	0.27451	0.029782	0.043291	0.145401	0.185699	0.215634	0.443748	0.475059
20-Λ 29-Λ	0.27342	0.033588	0.073141	0.15128	0.189013	0.234095	0.436176	0.461718
20-Λ 30-Δ	0.27967	0.018378	0.084998	0.175157	0.182412	0.228594	0.432349	0.486613
20-Λ 30-Λ	0.2805	0.011709	0.114216	0.180587	0.189708	0.250203	0.424639	0.475442
20-Λ Δ-THTB-7	0.26505	0.071747	0.084044	0.101655	0.106962	0.191051	0.392704	0.516776
20-Λ Λ-THTB-7	0.2608	0.064287	0.085676	0.097724	0.128814	0.196898	0.375572	0.508599
20-Λ 32-Δ	0.26358	0.076131	0.0789	0.093037	0.096462	0.188932	0.399909	0.510586
20-Λ 32-Λ	0.25914	0.06761	0.074621	0.090545	0.116803	0.194844	0.383579	0.502991
20-Λ Δ-SHEAPR-7	0.30083	0.026646	0.15728	0.161683	0.364786	0.379857	0.388768	0.391678
20-Λ Λ-SHEAPR-7	0.28774	0.020384	0.150471	0.158227	0.351835	0.355348	0.364873	0.385058
20-Λ 34-Δ	0.21995	0.025944	0.094476	0.099794	0.267097	0.278641	0.290999	0.292263
20-Λ 34-Λ	0.20671	0.021381	0.088017	0.095812	0.251381	0.257603	0.268303	0.283138
20-Λ 35-Δ	0.30176	0.008589	0.159761	0.173843	0.354279	0.380399	0.394554	0.394601
20-Λ 35-Λ	0.28985	0.035628	0.160437	0.163813	0.330187	0.368761	0.374612	0.385874
DPAC-7 22	0.00618	0.001977	0.00453	0.00453	0.005416	0.005416	0.008023	0.009975

DPAC-7 TSPPY-7	0.08988	0.028099	0.02909	0.03026	0.043583	0.057527	0.151253	0.16096
DPAC-7 24-Δ/Λ	0.08074	0.020667	0.032334	0.033866	0.036444	0.05051	0.133586	0.145898
DPAC-7 HEOB-7	0.10999	0.071323	0.071404	0.081519	0.103354	0.120859	0.145654	0.146099
DPAC-7 CPPY-7	0.12436	0.070641	0.074253	0.074253	0.114001	0.15703	0.165212	0.165214
DPAC-7 27-Δ/Λ	0.1137	0.066507	0.068702	0.07617	0.113789	0.13191	0.147496	0.153097
DPAC-7 28-Δ/Λ	0.1182	0.070648	0.075652	0.108528	0.110719	0.134261	0.147937	0.152114
DPAC-7 29-Δ/Λ	0.12129	0.068268	0.081506	0.089116	0.112544	0.137764	0.141852	0.178814
DPAC-7 30-Δ/Λ	0.12033	0.093557	0.099566	0.103689	0.124274	0.126663	0.134993	0.149083
DPAC-7 Δ/Λ-THTB-7	0.16568	0.083797	0.087352	0.102683	0.130179	0.200513	0.219959	0.247839
DPAC-7 32-Δ/Λ	0.16822	0.07693	0.096976	0.110691	0.126668	0.199696	0.21981	0.257434
DPAC-7 Δ/Λ-SHEAPR-7	0.13659	0.032331	0.032646	0.08268	0.091223	0.184006	0.188	0.210086
DPAC-7 34-Δ/Λ	0.17767	0.062075	0.070063	0.088629	0.14495	0.203117	0.236726	0.293334
DPAC-7 35-Δ/Λ	0.1136	0.012754	0.042005	0.069203	0.088179	0.146388	0.162216	0.167636
22 TSPPY-7	0.08945	0.024627	0.032349	0.032662	0.039907	0.05754	0.149719	0.16115
22 24-Δ/Λ	0.08082	0.01499	0.029888	0.037304	0.039777	0.051785	0.132915	0.145905
22 HEOB-7	0.11439	0.072523	0.072704	0.079365	0.111545	0.130635	0.150177	0.150618
22 CPPY-7	0.12906	0.068489	0.075939	0.075939	0.122186	0.166797	0.169747	0.169749
22 27-Δ/Λ	0.11833	0.068358	0.070505	0.074025	0.121953	0.141682	0.151937	0.157652
22 28-Δ/Λ	0.12292	0.070322	0.073814	0.112139	0.118871	0.138869	0.156549	0.157701
22 29-Δ/Λ	0.1257	0.066919	0.084879	0.087267	0.120717	0.146165	0.147393	0.183424
22 30-Δ/Λ	0.12431	0.094131	0.10578	0.109238	0.122071	0.134899	0.139651	0.153572
22 Δ/Λ-THTB-7	0.16725	0.08448	0.092446	0.108449	0.134883	0.199704	0.215352	0.252796
22 32-Δ/Λ	0.16972	0.082899	0.097467	0.115764	0.131226	0.198745	0.215202	0.26237
22 Δ/Λ-SHEAPR-7	0.13509	0.023643	0.032144	0.075719	0.094721	0.184101	0.187967	0.205481
22 34-Δ/Λ	0.17492	0.062034	0.069816	0.078722	0.137189	0.198514	0.239292	0.289437
22 35-Δ/Λ	0.11216	0.014551	0.038265	0.061662	0.091817	0.150274	0.158049	0.163072
TSPPY-7 24-Δ/Λ	0.01533	0.010376	0.011394	0.012889	0.014324	0.01604	0.017758	0.021554
TSPPY-7 HEOB-7	0.14599	0.059078	0.093199	0.118779	0.120753	0.164582	0.189425	0.212979
TSPPY-7 CPPY-7	0.15852	0.059552	0.098668	0.128629	0.139122	0.160197	0.209376	0.239177
TSPPY-7 27-Δ/Λ	0.14913	0.053743	0.091297	0.120362	0.131347	0.165693	0.196014	0.216411
TSPPY-7 28-Δ/Λ	0.14785	0.080838	0.086434	0.117637	0.121504	0.122481	0.19749	0.237509
TSPPY-7 29-Δ/Λ	0.14425	0.05597	0.079032	0.113379	0.124655	0.124913	0.207277	0.222063
TSPPY-7 30-Δ/Λ	0.15204	0.084904	0.123923	0.126986	0.138885	0.175246	0.191067	0.191373

TSPPY-7 Δ/Λ-THTB-7	0.15126	0.056443	0.058332	0.091059	0.101403	0.109825	0.207313	0.282764
TSPPY-7 32-Δ/Λ	0.15389	0.056411	0.072494	0.097874	0.100761	0.101219	0.206823	0.29085
TSPPY-7 Δ/Λ-SHEAPR-7	0.15794	0.072142	0.092167	0.130002	0.159644	0.194036	0.198768	0.203395
TSPPY-7 34-Δ/Λ	0.18649	0.053606	0.104658	0.136597	0.155099	0.210278	0.252755	0.280717
TSPPY-7 35-Δ/Λ	0.1396	0.095	0.101855	0.111046	0.146616	0.150246	0.170265	0.17783
24-Δ 24-Λ	0.02965	0.023114	0.023114	0.025217	0.026414	0.026414	0.039078	0.039078
24-Δ/Λ HEOB-7	0.13152	0.046311	0.079286	0.108732	0.110961	0.147076	0.173678	0.191636
24-Δ/Λ CPPY-7	0.14426	0.046631	0.08497	0.118698	0.129471	0.142955	0.193397	0.218173
24-Δ 27-Δ	0.13538	0.038586	0.079629	0.115596	0.118737	0.14288	0.176765	0.203326
24-Δ 27-Λ	0.13487	0.04083	0.077829	0.111228	0.121282	0.148313	0.180087	0.195176
24-Δ 28-Δ	0.14427	0.057223	0.11547	0.11896	0.127157	0.155603	0.179833	0.205491
24-Δ 28-Λ	0.13361	0.071074	0.072025	0.104079	0.106575	0.112788	0.181761	0.216259
24-Δ 29-Δ	0.13045	0.045023	0.064948	0.104793	0.10761	0.11484	0.18633	0.205942
24-Δ 29-Λ	0.15174	0.061962	0.098948	0.119639	0.144043	0.171984	0.186479	0.219408
24-Δ 30-Δ	0.13873	0.073443	0.096531	0.103261	0.130108	0.1627	0.176275	0.186747
24-Δ 30-Λ	0.13745	0.072012	0.113561	0.114397	0.129285	0.159309	0.16946	0.173956
24-Δ Δ-THTB-7	0.14337	0.051369	0.065134	0.087214	0.088729	0.100258	0.201581	0.266164
24-Δ Λ-THTB-7	0.15547	0.060432	0.066533	0.095145	0.096899	0.127713	0.200252	0.293721
24-Δ 32-Δ	0.14589	0.04896	0.079202	0.080108	0.095204	0.095597	0.201137	0.274306
24-Δ 32-Λ	0.15847	0.069204	0.074307	0.09481	0.106999	0.118645	0.199712	0.301787
24-Δ Δ-SHEAPR-7	0.15404	0.078621	0.104065	0.115005	0.144401	0.184632	0.196652	0.205566
24-Δ Λ-SHEAPR-7	0.15947	0.064318	0.081088	0.150783	0.170596	0.181899	0.197392	0.208379
24-Δ 34-Δ	0.18874	0.053775	0.125747	0.127085	0.158705	0.219154	0.25772	0.273625
24-Δ 34-Λ	0.18592	0.067391	0.09007	0.144175	0.147834	0.205016	0.242602	0.292882
24-Δ 35-Δ	0.14349	0.076165	0.101305	0.143067	0.14804	0.154176	0.16874	0.182861
24-Δ 35-Λ	0.13524	0.095673	0.100791	0.10161	0.138485	0.155307	0.158854	0.172787
24-Λ 27-Δ	0.13487	0.04083	0.077829	0.111228	0.121282	0.148313	0.180087	0.195176
24-Λ 27-Λ	0.13538	0.038586	0.079629	0.115596	0.118737	0.14288	0.176765	0.203326
24-Λ 28-Δ	0.13361	0.071074	0.072025	0.104079	0.106575	0.112788	0.181761	0.216259
24-Λ 28-Λ	0.14427	0.057223	0.11547	0.11896	0.127157	0.155603	0.179833	0.205491
24-Λ 29-Δ	0.15174	0.061962	0.098948	0.119639	0.144043	0.171984	0.186479	0.219408
24-Λ 29-Λ	0.13045	0.045023	0.064948	0.104793	0.10761	0.11484	0.18633	0.205942
24-Λ 30-Δ	0.13745	0.072012	0.113561	0.114397	0.129285	0.159309	0.16946	0.173956

24-Λ 30-Λ	0.13873	0.073443	0.096531	0.103261	0.130108	0.1627	0.176275	0.186747
24-Λ Δ-THTB-7	0.15547	0.060432	0.066533	0.095145	0.096899	0.127713	0.200252	0.293721
24-Λ Λ-THTB-7	0.14337	0.051369	0.065134	0.087214	0.088729	0.100258	0.201581	0.266164
24-Λ 32-Δ	0.15847	0.069204	0.074307	0.09481	0.106999	0.118645	0.199712	0.301787
24-Λ 32-Λ	0.14589	0.04896	0.079202	0.080108	0.095203	0.095597	0.201137	0.274305
24-Λ Δ-SHEAPR-7	0.15947	0.064318	0.081088	0.150783	0.170596	0.181899	0.197392	0.208379
24-Λ Λ-SHEAPR-7	0.15404	0.078621	0.104065	0.115005	0.144401	0.184632	0.196652	0.205566
24-Λ 34-Δ	0.18592	0.067391	0.09007	0.144175	0.147834	0.205016	0.242602	0.292882
24-Λ 34-Λ	0.18874	0.053775	0.125747	0.127085	0.158705	0.219154	0.25772	0.273625
24-Λ 35-Δ	0.13524	0.095673	0.100791	0.10161	0.138485	0.155307	0.158854	0.172787
24-Λ 35-Λ	0.14349	0.076165	0.101305	0.143067	0.14804	0.154176	0.16874	0.182861
HEOB-7 CPPY-7	0.0186	0.006128	0.007486	0.011019	0.011256	0.019754	0.020019	0.035916
HEOB-7 27-Δ/Λ	0.00981	0.005994	0.008477	0.008859	0.009375	0.009677	0.0123	0.012425
HEOB-7 28-Δ/Λ	0.03599	0.010288	0.011951	0.022945	0.027318	0.044425	0.045579	0.05911
HEOB-7 29-Δ/Λ	0.03049	0.010139	0.017097	0.02968	0.031816	0.034609	0.037916	0.039817
HEOB-7 30-Δ/Λ	0.02748	0.004225	0.020733	0.023718	0.023751	0.025071	0.035447	0.042737
HEOB-7 Δ/Λ-THTB-7	0.1371	0.083639	0.105856	0.109496	0.121727	0.131937	0.160332	0.208444
HEOB-7 32-Δ/Λ	0.13783	0.070633	0.112591	0.118791	0.121087	0.139131	0.153999	0.208511
HEOB-7 Δ/Λ-SHEAPR-7	0.18366	0.075907	0.125536	0.150065	0.151813	0.180687	0.196705	0.31254
HEOB-7 34-Δ/Λ	0.23786	0.107796	0.147732	0.189784	0.206915	0.223484	0.248777	0.414628
HEOB-7 35-Δ/Λ	0.17012	0.091468	0.107675	0.144026	0.146502	0.156524	0.170708	0.294573
CPPY-7 27-Δ/Λ	0.01483	0.006015	0.006545	0.007208	0.007743	0.012839	0.02267	0.025888
CPPY-7 28-Δ/Λ	0.03478	0.008694	0.013845	0.016766	0.028876	0.040511	0.051509	0.052839
CPPY-7 29-Δ/Λ	0.03104	0.005839	0.01183	0.03063	0.032655	0.032935	0.041138	0.042313
CPPY-7 30-Δ/Λ	0.03718	0.012723	0.017136	0.025473	0.0307	0.034486	0.053994	0.059374
CPPY-7 Δ/Λ-THTB-7	0.1428	0.087093	0.091475	0.117592	0.132502	0.149389	0.162432	0.216094
CPPY-7 32-Δ/Λ	0.14357	0.079316	0.100613	0.108401	0.143008	0.155828	0.156882	0.216263
CPPY-7 Δ/Λ-SHEAPR-7	0.19483	0.065711	0.12546	0.151664	0.18605	0.191273	0.203959	0.331438
CPPY-7 34-Δ/Λ	0.23795	0.045239	0.046728	0.062505	0.105346	0.275497	0.305892	0.455683
CPPY-7 35-Δ/Λ	0.18171	0.08056	0.112869	0.14622	0.163765	0.179363	0.181353	0.314057
27-Δ 27-Λ	0.01201	0.001845	0.001845	0.006344	0.012551	0.014329	0.017316	0.017316
27-Δ 28-Δ	0.03698	0.013725	0.015441	0.02146	0.029119	0.044745	0.053068	0.054948
27-Δ 28-Λ	0.03248	0.002339	0.0059	0.015189	0.029262	0.038735	0.04283	0.054062

27-Δ 29-Δ	0.03154	0.002294	0.025083	0.031744	0.032034	0.033661	0.037396	0.042003
27-Δ 29-Λ	0.02889	0.008202	0.012621	0.019617	0.024524	0.031342	0.041563	0.043793
27-Δ 30-Δ	0.03063	0.007439	0.015442	0.022097	0.030737	0.033514	0.037586	0.048019
27-Δ 30-Λ	0.03248	0.010013	0.01474	0.030577	0.032324	0.03576	0.037518	0.048995
27-Δ Δ-THTB-7	0.14125	0.085497	0.110451	0.114947	0.120799	0.140907	0.1588	0.21741
27-Δ Λ-THTB-7	0.13875	0.096087	0.096208	0.110795	0.115074	0.127416	0.16501	0.217474
27-Δ 32-Δ	0.14253	0.07398	0.106121	0.123907	0.132204	0.148134	0.152938	0.217491
27-Δ 32-Λ	0.13912	0.08348	0.106123	0.108932	0.121925	0.133778	0.159034	0.217562
27-Δ Δ-SHEAPR-7	0.18938	0.070178	0.135348	0.143873	0.161066	0.189418	0.205337	0.321146
27-Δ Λ-SHEAPR-7	0.1861	0.072009	0.119706	0.146054	0.161092	0.192315	0.205829	0.310282
27-Δ 34-Δ	0.24107	0.07964	0.169349	0.182637	0.244186	0.28551	0.296809	0.330409
27-Δ 34-Λ	0.23653	0.07699	0.170619	0.174565	0.24851	0.28046	0.287348	0.321134
27-Δ 35-Δ	0.1754	0.086168	0.117081	0.138512	0.153688	0.164719	0.180144	0.303047
27-Δ 35-Λ	0.17269	0.086127	0.104246	0.14073	0.155891	0.165	0.181872	0.293376
27-Λ 28-Δ	0.03248	0.002339	0.0059	0.015189	0.029262	0.038735	0.04283	0.054062
27-Λ 28-Λ	0.03698	0.013725	0.015441	0.02146	0.029119	0.044745	0.053068	0.054948
27-Λ 29-Δ	0.02889	0.008202	0.012621	0.019617	0.024524	0.031342	0.041563	0.043793
27-Λ 29-Λ	0.03154	0.002294	0.025083	0.031744	0.032034	0.033661	0.037396	0.042003
27-Λ 30-Δ	0.03248	0.010013	0.01474	0.030577	0.032324	0.03576	0.037518	0.048995
27-Λ 30-Λ	0.03063	0.007439	0.015442	0.022097	0.030737	0.033514	0.037586	0.048019
27-Λ Δ-THTB-7	0.13875	0.096087	0.096208	0.110795	0.115074	0.127417	0.16501	0.217474
27-Λ Λ-THTB-7	0.14125	0.085497	0.110451	0.114947	0.120799	0.140907	0.1588	0.21741
27-Λ 32-Δ	0.13912	0.08348	0.106123	0.108932	0.121925	0.133778	0.159034	0.217562
27-Λ 32-Λ	0.14253	0.07398	0.106121	0.123907	0.132204	0.148134	0.152938	0.217491
27-Λ Δ-SHEAPR-7	0.1861	0.072009	0.119706	0.146054	0.161092	0.192315	0.205829	0.310282
27-Λ Λ-SHEAPR-7	0.18938	0.070178	0.135348	0.143873	0.161066	0.189418	0.205337	0.321146
27-Λ 34-Δ	0.23653	0.07699	0.170619	0.174565	0.24851	0.28046	0.287348	0.321134
27-Λ 34-Λ	0.24107	0.07964	0.169349	0.182637	0.244186	0.28551	0.296809	0.330409
27-Λ 35-Δ	0.17269	0.086127	0.104246	0.14073	0.155891	0.165	0.181872	0.293376
27-Λ 35-Λ	0.1754	0.086168	0.117081	0.138512	0.153688	0.164719	0.180144	0.303047
28-Δ 28-Λ	0.06064	0.016511	0.018399	0.054264	0.054264	0.079474	0.080391	0.080391
28-Δ 29-Δ	0.05803	0.013765	0.014057	0.01921	0.060736	0.060762	0.081743	0.093555
28-Δ 29-Λ	0.03109	0.002737	0.013716	0.014898	0.026733	0.02786	0.036128	0.059611

28-Δ 30-Δ	0.05258	0.016898	0.030998	0.049425	0.051995	0.05342	0.065265	0.076456
28-Δ 30-Λ	0.03793	0.005315	0.017557	0.025008	0.027594	0.036932	0.04801	0.068403
28-Δ Δ-THTB-7	0.1615	0.097692	0.104149	0.116924	0.134284	0.14563	0.203229	0.260733
28-Δ Λ-THTB-7	0.12818	0.074896	0.083118	0.106335	0.127225	0.132318	0.14733	0.189184
28-Δ 32-Δ	0.16115	0.084815	0.112951	0.127487	0.136895	0.140747	0.19758	0.260749
28-Δ 32-Λ	0.13091	0.063861	0.071344	0.119944	0.123559	0.143144	0.157662	0.189199
28-Δ Δ-SHEAPR-7	0.19628	0.081592	0.082521	0.161564	0.174049	0.192806	0.249197	0.317075
28-Δ Λ-SHEAPR-7	0.18521	0.059184	0.136237	0.152371	0.177428	0.179447	0.183931	0.311984
28-Δ 34-Δ	0.24247	0.068954	0.147626	0.177685	0.230229	0.242231	0.257672	0.418749
28-Δ 34-Λ	0.23538	0.088079	0.175192	0.187347	0.210864	0.248243	0.302892	0.341238
28-Δ 35-Δ	0.18186	0.062034	0.083412	0.155759	0.16973	0.181567	0.211694	0.299762
28-Δ 35-Λ	0.17168	0.084819	0.1184	0.136548	0.148672	0.171269	0.174853	0.290587
28-Λ 29-Δ	0.03109	0.002737	0.013716	0.014898	0.026733	0.02786	0.036128	0.059611
28-Λ 29-Λ	0.05803	0.013765	0.014057	0.01921	0.060736	0.060762	0.081743	0.093555
28-Λ 30-Δ	0.03793	0.005315	0.017557	0.025008	0.027594	0.036932	0.04801	0.068403
28-Λ 30-Λ	0.05258	0.016898	0.030998	0.049425	0.051995	0.05342	0.065265	0.076456
28-Λ Δ-THTB-7	0.12818	0.074896	0.083118	0.106335	0.127225	0.132318	0.14733	0.189184
28-Λ Λ-THTB-7	0.1615	0.097692	0.104149	0.116924	0.134284	0.14563	0.203229	0.260733
28-Λ 32-Δ	0.13091	0.063861	0.071344	0.119944	0.123559	0.143145	0.157662	0.1892
28-Λ 32-Λ	0.16115	0.084815	0.112951	0.127487	0.136895	0.140747	0.197581	0.26075
28-Λ Δ-SHEAPR-7	0.18521	0.059184	0.136237	0.152371	0.177428	0.179447	0.183931	0.311984
28-Λ Λ-SHEAPR-7	0.19628	0.081592	0.082521	0.161564	0.174049	0.192806	0.249197	0.317075
28-Λ 34-Δ	0.23538	0.088079	0.175192	0.187347	0.210864	0.248243	0.302892	0.341238
28-Λ 34-Λ	0.24247	0.068954	0.147625	0.177685	0.230229	0.242231	0.257672	0.418749
28-Λ 35-Δ	0.17168	0.084819	0.1184	0.136548	0.148672	0.171269	0.174853	0.290587
28-Λ 35-Λ	0.18186	0.062034	0.083412	0.155759	0.16973	0.181567	0.211694	0.299762
29-Δ 29-Λ	0.05784	0.010629	0.041156	0.041156	0.051062	0.073391	0.073391	0.080877
29-Δ 30-Δ	0.03974	0.015747	0.02903	0.034489	0.036924	0.038533	0.052691	0.056124
29-Δ 30-Λ	0.04743	0.01522	0.015515	0.039993	0.045358	0.054174	0.065752	0.066046
29-Δ Δ-THTB-7	0.1143	0.077893	0.084642	0.085443	0.100564	0.118897	0.121404	0.178713
29-Δ Λ-THTB-7	0.1665	0.09377	0.1178	0.139049	0.156283	0.161174	0.201141	0.247351
29-Δ 32-Δ	0.11523	0.073609	0.075279	0.091971	0.110776	0.116278	0.12502	0.178845
29-Δ 32-Λ	0.16705	0.081056	0.131214	0.147791	0.150817	0.167111	0.194351	0.247508

29-Δ Δ-SHEAPR-7	0.17857	0.072553	0.128272	0.138157	0.168651	0.168663	0.1868	0.301046
29-Δ Λ-SHEAPR-7	0.20482	0.095117	0.139836	0.164218	0.164549	0.19225	0.233472	0.345765
29-Δ 34-Δ	0.22434	0.085448	0.171827	0.194515	0.204145	0.259531	0.271078	0.308429
29-Δ 34-Λ	0.25074	0.033182	0.050942	0.070529	0.092171	0.272953	0.330569	0.489035
29-Δ 35-Δ	0.16815	0.098061	0.119025	0.126515	0.133579	0.165996	0.177769	0.284825
29-Δ 35-Λ	0.18939	0.098159	0.1203	0.154178	0.158059	0.181674	0.193462	0.328324
29-Λ 30-Δ	0.04743	0.01522	0.015515	0.039993	0.045358	0.054174	0.065752	0.066046
29-Λ 30-Λ	0.03974	0.015747	0.02903	0.034489	0.036924	0.038533	0.052691	0.056124
29-Λ Δ-THTB-7	0.1665	0.09377	0.1178	0.13905	0.156283	0.161174	0.201141	0.247351
29-Λ Λ-THTB-7	0.1143	0.077893	0.084642	0.085443	0.100563	0.118897	0.121404	0.178713
29-Λ 32-Δ	0.16705	0.081056	0.131214	0.147791	0.150817	0.167111	0.194351	0.247508
29-Λ 32-Λ	0.11523	0.073609	0.075279	0.091971	0.110776	0.116278	0.12502	0.178845
29-Λ Δ-SHEAPR-7	0.20482	0.095117	0.139836	0.164218	0.164549	0.19225	0.233472	0.345765
29-Λ Λ-SHEAPR-7	0.17857	0.072553	0.128272	0.138157	0.168651	0.168663	0.1868	0.301046
29-Λ 34-Δ	0.25074	0.033182	0.050942	0.070529	0.092171	0.272954	0.330569	0.489035
29-Λ 34-Λ	0.22434	0.085448	0.171827	0.194515	0.204145	0.259531	0.271078	0.308429
29-Λ 35-Δ	0.18939	0.098159	0.1203	0.154178	0.158059	0.181674	0.193462	0.328324
29-Λ 35-Λ	0.16815	0.098061	0.119025	0.126515	0.133579	0.165996	0.177769	0.284825
30-Δ 30-Λ	0.02388	0.001434	0.014301	0.022955	0.025575	0.025575	0.031232	0.031232
30-Δ Δ-THTB-7	0.13359	0.099621	0.104432	0.110714	0.112286	0.132806	0.151074	0.196883
30-Δ Λ-THTB-7	0.14359	0.088932	0.099943	0.106508	0.133077	0.138581	0.164847	0.225808
30-Δ 32-Δ	0.13412	0.088599	0.101292	0.11772	0.122697	0.14174	0.141822	0.19669
30-Δ 32-Λ	0.14303	0.089173	0.100819	0.117795	0.128323	0.140058	0.155653	0.225601
30-Δ Δ-SHEAPR-7	0.18762	0.116409	0.128893	0.129536	0.170907	0.18559	0.203275	0.307415
30-Δ Λ-SHEAPR-7	0.19263	0.105935	0.116726	0.127057	0.176925	0.205431	0.21494	0.314738
30-Δ 34-Δ	0.23586	0.105172	0.159232	0.176963	0.232963	0.284562	0.28843	0.321301
30-Δ 34-Λ	0.23601	0.110431	0.15083	0.157575	0.246756	0.286299	0.30149	0.310425
30-Δ 35-Δ	0.17532	0.112356	0.121017	0.134666	0.148408	0.166011	0.193444	0.287673
30-Δ 35-Λ	0.18062	0.086718	0.124748	0.132778	0.171392	0.178787	0.195357	0.296901
30-Λ Δ-THTB-7	0.14359	0.088932	0.099943	0.106508	0.133077	0.13858	0.164847	0.225807
30-Λ Λ-THTB-7	0.13359	0.099621	0.104432	0.110714	0.112286	0.132806	0.151074	0.196883
30-Λ 32-Δ	0.14303	0.089173	0.100819	0.117795	0.128323	0.140058	0.155653	0.225601
30-Λ 32-Λ	0.13412	0.088599	0.101292	0.11772	0.122697	0.14174	0.141822	0.196691

30-Λ Δ-SHEAPR-7	0.19263	0.105935	0.116726	0.127057	0.176925	0.205431	0.21494	0.314738
30-Λ Λ-SHEAPR-7	0.18762	0.116409	0.128893	0.129535	0.170907	0.18559	0.203275	0.307415
30-Λ 34-Δ	0.23601	0.110431	0.15083	0.157575	0.246756	0.286299	0.30149	0.310425
30-Λ 34-Λ	0.23586	0.105172	0.159232	0.176963	0.232963	0.284562	0.28843	0.321301
30-Λ 35-Δ	0.18062	0.086718	0.124748	0.132778	0.171392	0.178787	0.195357	0.296901
30-Λ 35-Λ	0.17532	0.112356	0.121017	0.134666	0.148408	0.166011	0.193444	0.287673
Δ-THTB-7 Λ-THTB-7	0.20892	2.28E-07	0.159132	0.159132	0.159132	0.159132	0.319554	0.319554
Δ-THTB-7 32-Δ	0.01277	1.03E-07	0.011847	0.011847	0.013915	0.013915	0.01538	0.01538
Δ-THTB-7 32-Λ	0.21539	1.84E-07	0.15516	0.15516	0.173129	0.173129	0.329132	0.329132
Δ-THTB-7 Δ-SHEAPR-7	0.13502	5.39E-08	0.12585	0.12585	0.127324	0.127324	0.178204	0.178204
Δ-THTB-7 Λ-SHEAPR-7	0.19456	1.44E-07	0.126674	0.126674	0.235242	0.235243	0.247182	0.247182
Δ-THTB-7 34-Δ	0.19628	0.083639	0.155797	0.174979	0.21264	0.212713	0.238326	0.246039
Δ-THTB-7 34-Λ	0.23972	8.14E-08	0.093673	0.093673	0.285249	0.285249	0.333153	0.333153
Δ-THTB-7 35-Δ	0.14337	0.044437	0.125572	0.12574	0.140318	0.140428	0.166687	0.20771
Δ-THTB-7 35-Λ	0.19396	0.040553	0.125893	0.151577	0.203183	0.20857	0.25919	0.266298
Λ-THTB-7 32-Δ	0.21539	2.61E-07	0.15516	0.15516	0.173129	0.173129	0.329132	0.329132
Λ-THTB-7 32-Λ	0.01277	3.06E-07	0.011847	0.011847	0.013915	0.013915	0.01538	0.01538
Λ-THTB-7 Δ-SHEAPR-7	0.19456	1.94E-07	0.126674	0.126674	0.235242	0.235243	0.247182	0.247182
Λ-THTB-7 Λ-SHEAPR-7	0.13502	2.29E-07	0.12585	0.12585	0.127324	0.127324	0.178204	0.178204
Λ-THTB-7 34-Δ	0.23972	8.14E-08	0.093673	0.093673	0.285249	0.285249	0.333153	0.333153
Λ-THTB-7 34-Λ	0.19628	0.083639	0.155797	0.174979	0.21264	0.212712	0.238326	0.246039
Λ-THTB-7 35-Δ	0.19396	0.040553	0.125892	0.151577	0.203183	0.20857	0.25919	0.266298
Λ-THTB-7 35-Λ	0.14337	0.044437	0.125572	0.12574	0.140318	0.140428	0.166687	0.20771
32-Δ 32-Λ	0.2214	1.07E-07	0.169001	0.169001	0.169002	0.169002	0.338298	0.338298
32-Δ Δ-SHEAPR-7	0.1362	2.41E-07	0.124901	0.124901	0.132975	0.132976	0.177882	0.177882
32-Δ Λ-SHEAPR-7	0.19458	1.82E-07	0.137371	0.137371	0.224504	0.224504	0.251471	0.251471
32-Δ 34-Δ	0.19478	0.088234	0.145204	0.187402	0.20272	0.214479	0.234294	0.244115
32-Δ 34-Λ	0.23567	2.44E-07	0.099491	0.099491	0.28407	0.28407	0.322188	0.322188
32-Δ 35-Δ	0.14541	0.044454	0.122463	0.130823	0.143289	0.147993	0.16867	0.207434
32-Δ 35-Λ	0.19403	0.040333	0.136791	0.16177	0.192108	0.213098	0.248335	0.270268
32-Λ Δ-SHEAPR-7	0.19458	1.82E-07	0.137371	0.137371	0.224504	0.224504	0.251471	0.251471
32-Λ Λ-SHEAPR-7	0.1362	1.30E-07	0.124901	0.124901	0.132975	0.132976	0.177882	0.177882
32-Λ 34-Δ	0.23567	2.44E-07	0.099491	0.099491	0.28407	0.28407	0.322188	0.322188

32-Λ 34-Λ	0.19478	0.088234	0.145204	0.187402	0.20272	0.214478	0.234294	0.244115
32-Λ 35-Δ	0.19403	0.040334	0.136791	0.16177	0.192108	0.213098	0.248335	0.270268
32-Λ 35-Λ	0.14541	0.044454	0.122463	0.130823	0.143289	0.147993	0.16867	0.207434
Δ-SHEAPR-7 Λ-SHEAPR-7	0.22791	1.43E-07	0.138204	0.138204	0.285221	0.285221	0.285221	0.285221
Δ-SHEAPR-7 34-Δ	0.08343	1.33E-07	0.062653	0.062653	0.099497	0.099497	0.102656	0.102656
Δ-SHEAPR-7 34-Λ	0.26406	1.41E-07	0.1553	0.1553	0.268897	0.268897	0.384212	0.384212
Δ-SHEAPR-7 35-Δ	0.03024	0.001969	0.01901	0.026848	0.031437	0.032662	0.038755	0.041954
Δ-SHEAPR-7 35-Λ	0.23338	0.041447	0.106081	0.154411	0.261359	0.283156	0.310724	0.3153
Λ-SHEAPR-7 34-Δ	0.26406	2.70E-08	0.1553	0.1553	0.268897	0.268897	0.384212	0.384212
Λ-SHEAPR-7 34-Λ	0.08343	9.12E-08	0.062653	0.062653	0.099497	0.099497	0.102656	0.102656
Λ-SHEAPR-7 35-Δ	0.23338	0.041447	0.106081	0.154411	0.261359	0.283156	0.310724	0.3153
Λ-SHEAPR-7 35-Λ	0.03024	0.001969	0.01901	0.026848	0.031437	0.032662	0.038755	0.041954
34-Δ 34-Λ	0.27969	9.29E-08	0.09626	0.09626	0.363677	0.363677	0.363677	0.363677
34-Δ 35-Δ	0.09053	0.040437	0.06277	0.071587	0.080253	0.09437	0.121199	0.128957
34-Δ 35-Λ	0.26047	0.050674	0.078592	0.080156	0.206122	0.280929	0.376053	0.44376
34-Λ 35-Δ	0.26047	0.050674	0.078592	0.080156	0.206122	0.280929	0.376053	0.44376
34-Λ 35-Λ	0.09053	0.040437	0.06277	0.071587	0.080253	0.09437	0.121199	0.128957

Mathematical Essentials

A convex polyhedron is a solid figure with flat polygonal faces, straight edges, and vertices. In a convex polyhedron, any line segment joining two points within or on the polyhedron lies entirely inside or on the polyhedron.

According to Euler's formula (1750), any 3-dimensional (3D) convex polyhedron P must obey:

$$V - E + F = 2$$

where V, E, and F represent the numbers of vertices, edges, and faces, respectively. Therefore, specifying only V and E suffices, since the number of faces (F) is already determined uniquely by the formula. Because each face of a polyhedron must have at least three edges, and each edge is shared by exactly two faces, these conditions, when combined with Euler's formula, naturally lead to the following inequalities (Steinitz, 1906):

$$F \leq 2V - 4$$
$$V \leq 2F - 4$$

As a result, plotting the number of faces (F) against the number of vertices (V) for all convex polyhedra, as shown in Figure S7, yields a characteristic cone-shaped region (Ziegler, 2004). The bounding inequalities appear graphically as two straight lines intersecting at the point V = 4, F = 4, forming a clearly defined, conical area. Therefore, all possible topologically distinct convex polyhedra must lie within this bounded region constrained by the upper and lower limits. It is important to note that each point in the plot might not necessarily correspond to a single polyhedron, that is, nonisomorphic polyhedra can have equal numbers of *V*, *E*, and *F*.

A graph **G** is an abstract mathematical structure defined as an ordered pair **G** = (**V**,**E**), where **V** is a non-empty set of vertices, and **E** is a set of edges that represent adjacency relations between pairs of vertices.

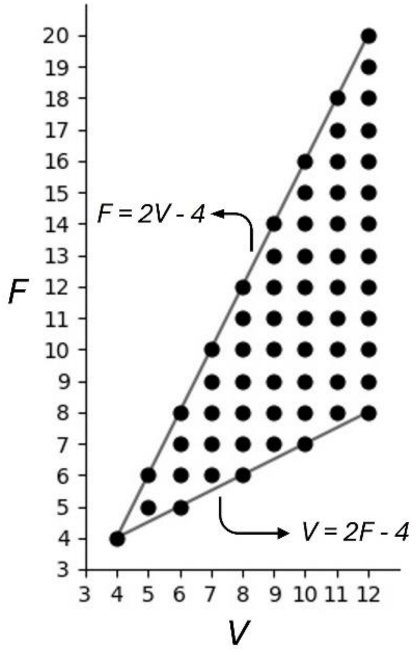

Figure S7. A bidimensional F vs. V plot of all convex polyhedra, with their upper and lower bound limits indicated. All quantities recalculated and redrawn by the authors; layout inspired by Ziegler, G. M. (2004), but all data and rendering are original.

The linkage between graphs and convex polyhedra is summarized in Steinitz's theorem (1922), which states that a finite graph **G** = (**V**,**E**) is the graph of a 3D convex polyhedron P if and only if it is simple, planar, and 3-connected(Steinitz, 1922). Such a graph captures the wireframe skeleton of the polyhedron by focusing on the fact that its edges are linkages that connect vertices. (Grünbaum, 2007; Matoušek, 2002) In this theorem, when it is said the graph **G** is to be "simple", it means its structure is loopless and absent of parallel edges. The planarity property ensures that **G** can be drawn on a plane in a crossing-free straight-line embedding (Tutte, 1963). The resulting figure is called a Schlegel diagram (Schlegel, 1883), which is a projection of the polyhedron onto a plane with one face expanded to form the outer boundary. A graph is 3-connected if it remains connected even after removing any two vertices, and any two vertices are joined by at least three independent paths (Balinski, 1961). According to Steinitz's theorem, you can always "draw" graph **G** as a polyhedron P in 3D. This drawing is executed in a smooth, reversible manner that preserves the connections between the edges and vertices along the boundaries.(Rote, 2012)

References

Balinski, M. (1961). On the graph structure of convex polyhedra in *n* -space. *Pacific Journal of Mathematics*, *11*(2), 431–434. https://doi.org/10.2140/pjm.1961.11.431

Grünbaum, B. (2007). Graphs of polyhedra; polyhedra as graphs. *Discrete Mathematics*, *307*(3–5), 445–463. https://doi.org/10.1016/j.disc.2005.09.037

Matoušek, J. (2002). Convex Polytopes. In J. Matoušek (Ed.), *Lectures on Discrete Geometry. Graduate Texts in Mathematics* (Vol. 212, pp. 77–124). Springer. https://doi.org/10.1007/978-1-4613-0039-7_5

Rote, G. (2012). *Realizing Planar Graphs as Convex Polytopes* (pp. 238–241). https://doi.org/10.1007/978-3-642-25878-7_23

Schlegel, V. (1883). *Theorie der homogen zusammengesetzten Raumgebilde*. Nova Acta, Ksl. Leop.-Carol. Deutsche Akademie der Naturforscher, Band XLIV, Nr. 4, Druck von E. Blochmann & Sohn in Dresden.

Silva, F. T., Lins, S. L. S., & Simas, A. M. (2018). Stereoisomerism in Lanthanide Complexes: Enumeration, Chirality, Identification, Random Coordination Ratios. *Inorganic Chemistry*, *57*(17), 10557–10567. https://doi.org/10.1021/acs.inorgchem.8b01133

Steinitz, E. (1906). Über die Eulerschen Polyederrelationen. *Archiv Für Mathematik Und Physik*, *11*, 86–88.

Steinitz, E. (1922). Polyeder und Raumeinteilungen. *Encyclopädie Der Mathematischen Wissenschaften*, *3*, 1–33.

Tutte, W. T. (1963). How to Draw a Graph. *Proceedings of the London Mathematical Society*, *s3-13*(1), 743–767. https://doi.org/10.1112/plms/s3-13.1.743

Ziegler, G. M. (2004). *Convex Polytopes: Extremal Constructions and f-Vector Shapes*.
