# Supplementary material for: Thermally Distinguishable Polyhedral Shapes in Chemistry: 6- and 7‑Coordination
Source: ACS Omega. 2025 Sep 29;10(40):47189–209. doi: 10.1021/acsomega.5c05878 (PMC12529146; doi:10.1021/acsomega.5c05878)
Supplement: Supplementary file 1 [file ao5c05878_si_001.zip › i - Read_Me_First.pdf]

# Including the new thermally distinguishable shapes in the SHAPE 2.1 software

## SHAPE 2.1 Software

Shape is a program that calculates Continuous Shape Measures (CShMs) by comparing a set of points (e.g., coordinating atom positions) to ideal reference polyhedra, either centered or non-centered. It evaluates structural distortions, interconversion coordinates, and does not distinguish between chiral enantiomers. The method is based on algorithms developed by Pinsky, Avnir, and others.

## Getting Started with the SHAPE Software

To start, please, go to the download page of the Shape Software:
https://www.ee.ub.edu/downloads/

Go to the end of the page, where you will find large blue buttons. Hover over the "Descargar" buttons, choose the appropriate version for your operating system and, please, download it. *Warning*: if you are using Windows, please download the 32 bit version even if your computer is a 64 bit one. If you are using Linux, choose the appropriate version, either 32 or 64-bits one.

## On Windows:

You should have downloaded the file *SHAPE_2.1_win32.zip*. Then, unzip it "here" so that a new folder *SHAPE_2.1_win32* is created. From now on we will call this folder, in this tutorial, the "*SHAPE_2.1_win32*" folder.

Go inside this *"SHAPE_2.1_win32"* folder, two files will appear: "*shape_2.1.bat*" and "*shape_2.1_win32.exe*" files. Ignore the *.bat* file. We will work with the *.exe* file alone.

In our supporting information *.zip* file, we provided a folder named "*ii - TDPSs_SHAPE_ref_files*". Copy all files from the *"ii - TDPSs_SHAPE_ref_files"* folder into the *"SHAPE_2.1_win32"* directory.

For the purposes of this demonstration, we employ the file *HABLII_DAC-6_OLD.dat*, which is contained within the folder "*ii - TDPSs_SHAPE_ref_files*". Its contents are presented below to illustrate the structure of the data.

The numbers "1 2 3 4 5" in the 5$^{th}$ line indicate that the coordination polyhedron below is to be compared to all 5 predefined shapes that come with the Shape software.

```
$ CN-6 structures

! Coordinate atoms  Metal
       6          1
1 2 3 4 5

HABLII_0
 Th  13.00590     4.873200     0.496400
  C  14.98400     4.381500     -1.17600
  C  12.87000     6.930600     -1.12090
  C  13.84450     6.285800     2.456000
  C  10.78720     5.982300     1.291700
  C  11.27040     3.114200     -0.14670
  C  14.07870     3.000100     2.030500
```

Click the address bar at the top of File Explorer where the folder path is displayed. Clicking anywhere in the empty space of the address bar will highlight the entire path. Now, type the following letters: ***c,*** then ***m,*** and finally ***d*** in sequence, and press the ***enter*** key.

A ***Command Prompt*** window will open directly in this folder.

On the command prompt, then type: ***shape_2.1_win32.exe***

Then, when asked for the ***"Input file name ?"*** enter the input file name *HABLII_DAC-6_OLD.dat* containing data for the complex of CSD refcode HABLII.

The screen will look like:

```
S H A P E   v2.1         Continuous Shape Measures calculation
(c) 2013  Electronic Structure Group, Universitat de Barcelona
                   Contact:  llunell@ub.edu

Shape command-line usage:
   Standard Calculation:  shape_2.1_win32.exe filename[.dat]
   Ideal Structures List: shape_2.1_win32.exe +[n]
   Keyword's Information: shape_2.1_win32.exe -h

Input file name ? HABLII_DAC-6_OLD.dat
Reading data from file HABLII_DAC-6_OLD.dat
CShM table in HABLII_DAC-6_OLD.tab

Arrivederci.
```

Please open the file *HABLII_DAC-6_OLD.tab* with a text editor such as Notepad. Its contents should look like the example shown below:":

```
-------------------------------------------------------------------------------
S H A P E   v2.1         Continuous Shape Measures calculation
(c) 2013  Electronic Structure Group, Universitat de Barcelona
                   Contact:  llunell@ub.edu
-------------------------------------------------------------------------------

CN-6 structures

HP-6               1 D6h    Hexagon
PPY-6              2 C5v    Pentagonal pyramid
OC-6               3 Oh     Octahedron
TPR-6              4 D3h    Trigonal prism
JPPY-6             5 C5v    Johnson pentagonal pyramid J2

Structure [ML6 ]         HP-6        PPY-6         OC-6        TPR-6       JPPY-6
 HABLII_0       ,      34.135,      14.967,       6.173,       4.834,      18.513
```

Please note that this HABLII structure does not match any of the five predefined shapes, as the CShM values below each of them are above 3 units. According to the authors of the Shape software, this indicates a significant distortion, meaning the reference polyhedron can only be considered a "rather crude description of the real structure".

Now, since HABLII is a six-coordinate polyhedron, we will now include our new thermally distinguishable shape DAC-6 to be considered along the previous one. For that purpose, the reference *.dat* file for DAC-6, named "*DAC-6.ref*" is already present in the "*SHAPE_2.1_win32*". To include this new reference file in the Shape calculation, we added the line" *%reference DAC-6*" as the second line of the file. This will designate it as reference 0. Then, we updated the fifth line so that it includes the number 0, resulting in the sequence: 0 1 2 3 4 5, as shown below:

*HABLII_DAC-6.dat*

```
$ CN-6 structures
%reference DAC-6
! Coordinate atoms  Metal
       6           1
0 1 2 3 4 5

HABLII_0
 Th  13.00590    4.873200    0.496400
  C  14.98400    4.381500   -1.17600
  C  12.87000    6.930600   -1.12090
  C  13.84450    6.285800    2.456000
  C  10.78720    5.982300    1.291700
  C  11.27040    3.114200   -0.14670
  C  14.07870    3.000100    2.030500
```

Now, as before, run ***shape_2.1_win32.exe***, and when prompted for the *.dat* file, write "*HABLII_DAC-6.dat*" as shown below.

```
S H A P E   v2.1         Continuous Shape Measures calculation
(c) 2013  Electronic Structure Group, Universitat de Barcelona
                   Contact:  llunell@ub.edu

Shape command-line usage:
   Standard Calculation:  shape_2.1_win32.exe filename[.dat]
   Ideal Structures List: shape_2.1_win32.exe +[n]
   Keyword's Information: shape_2.1_win32.exe -h

Input file name ? HABLII_DAC-6.dat
Reading data from file HABLII_DAC-6.dat
CShM table in HABLII_DAC-6.tab
Reading reference structures from file DAC-6.ref

Arrivederci.
```

Now, open the "*HABLII_DAC-6.tab*" file with the new results:

```
-------------------------------------------------------------------------------
S H A P E   v2.1         Continuous Shape Measures calculation
(c) 2013  Electronic Structure Group, Universitat de Barcelona
                   Contact:  llunell@ub.edu
-------------------------------------------------------------------------------

CN-6 structures

DAC-6          0 C2v   Digonal anticupola
HP-6           1 D6h   Hexagon
PPY-6          2 C5v   Pentagonal pyramid
OC-6           3 Oh    Octahedron
TPR-6          4 D3h   Trigonal prism
JPPY-6         5 C5v   Johnson pentagonal pyramid J2

Structure [ML6 ]       DAC-6       HP-6       PPY-6       OC-6       TPR-6      JPPY-6
 HABLII_0      ,       1.351,     34.135,     14.967,     6.173,     4.834,     18.513
```

Note that now, this same coordination polyhedron matches the DAC-6 shape, as its CShM value is 1.351, below 3, indicating that DAC-6 is its correct shape.

Now, let us turn our attention to 7 coordinate complexes. As an example, we provided file "*HOYGEL_HECU-7_OLD.dat*" whose contents are shown below:

```
$ CN-7 structures

! Coordinate atoms  Metal
       7          1
1 2 3 4 5 6 7

HABLII_0
 Eu    1.96030     4.810200     11.47920
  I    1.84900     3.604300     14.51880
  N    4.47830     3.726700     11.05320
  N    1.95730     2.189600     10.65450
  N    0.15470     4.276200     9.466600
  N    1.74300     7.409900     12.42830
  N    3.95120     6.609000     10.64540
  N   -0.63620     5.631900     11.99900
```

When you run *shape_2.1_win32.exe*, it will generate a *.tab* file named "*HOYGEL_HECU-7_OLD.tab*", whose contents are shown below:

```
-------------------------------------------------------------------------------
S H A P E   v2.1         Continuous Shape Measures calculation
(c) 2013  Electronic Structure Group, Universitat de Barcelona
                   Contact:  llunell@ub.edu
-------------------------------------------------------------------------------

CN-6 structures

HP-7            1 D7h    Heptagon
HPY-7           2 C6v    Hexagonal pyramid
PBPY-7          3 D5h    Pentagonal bipyramid
COC-7           4 C3v    Capped octahedron
CTPR-7          5 C2v    Capped trigonal prism
JPBPY-7         6 D5h    Johnson pentagonal bipyramid J13
JETPY-7         7 C3v    Johnson elongated triangular pyramid J7

Structure [ML7 ]        HP-7        HPY-7       PBPY-7        COC-7        CTPR-7       JPBPY-7       JETPY-7
 HABLII_0       ,     33.732,       8.993,      12.459,       6.444,        8.055,       15.741,       18.222
```

Please, note that, once again, none of the predefined shapes displayed CShM values below 3 units.

We have 11 new thermally distinguishable shapes for coordination number 7. Unfortunately, we would have to test each of them separately, one by one.

Since we already know that the shape of this HOYGEL complex is the hemicube HECU-7, we wrote in the second line "%reference HECU-7", and added the zero in the fifth line as before.

The contents of "*HOYGEL_HECU-7.dat*" now become:

```
$ CN-7 structures
%reference HECU-7
! Coordinate atoms  Metal
      7          1
0 1 2 3 4 5 6 7

HABLII_0
 Eu    1.96030     4.810200     11.47920
  I    1.84900     3.604300     14.51880
  N    4.47830     3.726700     11.05320
  N    1.95730     2.189600     10.65450
  N    0.15470     4.276200     9.466600
  N    1.74300     7.409900     12.42830
  N    3.95120     6.609000     10.64540
  N   -0.63620     5.631900     11.99900
```

We now run “*shape_2.1_win32.exe*” again and get as output file “*HOYGEL_HECU-7.tab*”, whose contents are shown below:

```
--------------------------------------------------------------------------------
S H A P E   v2.1        Continuous Shape Measures calculation
(c) 2013  Electronic Structure Group, Universitat de Barcelona
                  Contact:  llunell@ub.edu
--------------------------------------------------------------------------------

CN-7 structures

HECU-7         0 C2v   Hemicube
HP-7           1 D7h   Heptagon
HPY-7          2 C6v   Hexagonal pyramid
PBPY-7         3 D5h   Pentagonal bipyramid
COC-7          4 C3v   Capped octahedron
CTPR-7         5 C2v   Capped trigonal prism
JPBPY-7        6 D5h   Johnson pentagonal bipyramid J13
JETPY-7        7 C3v   Johnson elongated triangular pyramid J7

Structure [ML7 ]      HECU-7        HP-7        HPY-7       PBPY-7       COC-7       CTPR-7      JPBPY-7      JETPY-7
 HABLII_0       ,      0.607,      33.732,      8.993,      12.459,      6.444,      8.055,      15.741,      18.222
```

Now, the CShM value for the shape HECU-7 is 0.607, indicating that the Hemicube is the correct shape for this coordination polyhedron.

## On Linux:

The following instructions for Linux assume the 32-bit version of the Shape software, although the procedure is similar for the 64-bit version. The primary difference is that folders and associated files for the 64-bit version explicitly specify '64-bit' instead of '32-bit'.

You should have downloaded the file *SHAPE_2.1_linux_32.zip*. Next, click on it, then right-click and select "Extract". This action will create a directory named *SHAPE_2.1_linux_32*. Inside this directory, you will find another subdirectory with the same name. For the remainder of this tutorial, we will refer to this inner subdirectory as the *SHAPE_2.1_linux_32* directory.

Navigate into the *SHAPE_2.1_linux_32* directory. Inside, you will see a Linux executable file named “*shape_2.1_linux32*”, accompanied by a PDF file *(*.pdf*) containing the Shape User Manual, a macOS metadata file *(.DS_Store*), and the *SHAPE_2.1_example_files* directory. You can safely disregard all these additional files and directories. For the purpose of this tutorial, we will focus exclusively on the Linux executable *shape_2.1_linux32*.

In the supporting materials, you will find a subdirectory named “*ii - TDPSs_SHAPE_ref_files*”. Move this folder into the *SHAPE_2.1_linux_32* directory.

Next, open the *ii - TDPSs_SHAPE_ref_files* subdirectory. Select all its contents (you can press Ctrl+A to highlight everything), then right-click and choose "Copy". Navigate back to the parent directory, *SHAPE_2.1_linux_32*, right-click on an empty area, and select "Paste". This will copy all files from *ii - TDPSs_SHAPE_ref_files* into the *SHAPE_2.1_linux_32* directory.

For this demonstration, we will use the file *HABLII_DAC-6_OLD.dat*. Its contents are shown below to illustrate the structure of the data.

The numbers “1 2 3 4 5” in the 5$^{th}$ line indicate that the coordination polyhedron below is to be compared to all 5 predefined shapes that come with the Shape software.

```
$ CN-6 structures

! Coordinate atoms  Metal
       6           1
1 2 3 4 5

HABLII_0
 Th  13.00590     4.873200     0.496400
  C  14.98400     4.381500    -1.17600
  C  12.87000     6.930600    -1.12090
  C  13.84450     6.285800     2.456000
  C  10.78720     5.982300     1.291700
  C  11.27040     3.114200    -0.14670
  C  14.07870     3.000100     2.030500
```

Please, right-click on the *“SHAPE_2.1_linux_32”* directory and select "*Open in Terminal*" from the context menu

A *Linux Terminal* window will open in the specific folder.

On the command prompt, then type:

./***shape_2.1_linux32***

Then, when asked for the **“Input file name ?”** enter the input file name *HABLII_DAC-6_OLD.dat* containing data for the complex of CSD refcode HABLII.

The screen will look like:

```
S H A P E   v2.1         Continuous Shape Measures calculation
(c) 2013  Electronic Structure Group, Universitat de Barcelona
                 Contact:  llunell@ub.edu


Input file name ?  "HABLII_DAC-6_OLD.dat"
Reading data from file HABLII_DAC-6_OLD.dat
CShM table in HABLII_DAC-6_OLD.tab

Arrivederci.
```

After running the program, navigate back to the *SHAPE_2.1_linux_32* directory. Then, right-click on the file “*HABLII_DAC-6_OLD.tab*” and choose "Open With Text Editor" from the context menu. The contents should resemble the example shown below:

```
-------------------------------------------------------------------------------
S H A P E   v2.1         Continuous Shape Measures calculation
(c) 2013  Electronic Structure Group, Universitat de Barcelona
                 Contact:  llunell@ub.edu
-------------------------------------------------------------------------------

CN-6 structures

HP-6               1 D6h    Hexagon
PPY-6              2 C5v    Pentagonal pyramid
OC-6               3 Oh     Octahedron
TPR-6              4 D3h    Trigonal prism
JPPY-6             5 C5v    Johnson pentagonal pyramid J2

Structure [ML6 ]        HP-6        PPY-6        OC-6        TPR-6       JPPY-6
 HABLII_0       ,      34.135,      14.967,      6.173,      4.834,      18.513
```

Please note that this HABLII structure does not match the predefined shapes, as the CShM values below each of them are above 3 units. According to the authors of the Shape software, this indicates a significant distortion, meaning the reference polyhedron can only be considered a “rather crude description of the real structure”.

Now, since HABLII is a six-coordinate polyhedron, we will now include our new thermally distinguishable shape DAC-6 to be considered along the previous one. For that purpose, the reference *.dat* file for DAC-6, named “*DAC-6.ref*” is already present in the “*SHAPE_2.1_linux_32*” folder. To include this new reference file in the Shape calculation, we added the line” *%reference DAC-6*” as the second line of the file. This will designate it as reference 0. Then, we updated the fifth line so that it includes the number 0, resulting in the sequence: 0 1 2 3 4 5, as shown below:

*HABLII_DAC-6.dat*

```
$ CN-6 structures
%reference DAC-6
! Coordinate atoms  Metal
       6          1
0 1 2 3 4 5

HABLII_0
 Th  13.00590     4.873200     0.496400
  C  14.98400     4.381500     -1.17600
  C  12.87000     6.930600     -1.12090
  C  13.84450     6.285800     2.456000
  C  10.78720     5.982300     1.291700
  C  11.27040     3.114200     -0.14670
  C  14.07870     3.000100     2.030500
```

Now, as before, run **./shape_2.1_linux32** in the terminal, and when prompted for the *.dat* file, write “*HABLII_DAC-6.dat*” as shown below.

```
S H A P E   v2.1         Continuous Shape Measures calculation
(c) 2013  Electronic Structure Group, Universitat de Barcelona
                  Contact:  llunell@ub.edu


Input file name ? HABLII_DAC-6.dat
Reading data from file HABLII_DAC-6.dat
CShM table in HABLII_DAC-6.tab
Reading reference structures from file DAC-6.ref

Arrivederci.
```

Now, open the “*HABLII_DAC-6.tab*” file with the new results:

```
-------------------------------------------------------------------------------
S H A P E   v2.1         Continuous Shape Measures calculation
(c) 2013  Electronic Structure Group, Universitat de Barcelona
                  Contact:  llunell@ub.edu
-------------------------------------------------------------------------------

CN-6 structures

DAC-6           0 C2v   Digonal anticupola
HP-6            1 D6h   Hexagon
PPY-6           2 C5v   Pentagonal pyramid
OC-6            3 Oh    Octahedron
TPR-6           4 D3h   Trigonal prism
JPPY-6          5 C5v   Johnson pentagonal pyramid J2

Structure [ML6 ]        DAC-6       HP-6       PPY-6        OC-6       TPR-6       JPPY-6
 HABLII_0       ,       1.351,     34.135,     14.967,      6.173,      4.834,      18.513
```

Note that now, this same coordination polyhedron matches the DAC-6 shape, as its CShM value is 1.351, below 3, indicating that DAC-6 is its correct shape.

Now, let us turn our attention to 7 coordinate complexes. As an example, we provided file "*HOYGEL_HECU-7_OLD.dat*" whose contents are shown below:

```
$ CN-7 structures

! Coordinate atoms  Metal
       7          1
1 2 3 4 5 6 7

HABLII_0
 Eu   1.96030     4.810200     11.47920
  I   1.84900     3.604300     14.51880
  N   4.47830     3.726700     11.05320
  N   1.95730     2.189600     10.65450
  N   0.15470     4.276200     9.466600
  N   1.74300     7.409900     12.42830
  N   3.95120     6.609000     10.64540
  N  -0.63620     5.631900     11.99900
```

Upon running **./shape_2.1_linux32** the following *.tab* file named "*HOYGEL_HECU-7_OLD.tab*" will be output whose contents are shown below:

```
-------------------------------------------------------------------------------
S H A P E   v2.1         Continuous Shape Measures calculation
(c) 2013  Electronic Structure Group, Universitat de Barcelona
                  Contact:  llunell@ub.edu
-------------------------------------------------------------------------------

CN-7 structures

HP-7               1 D7h    Heptagon
HPY-7              2 C6v    Hexagonal pyramid
PBPY-7             3 D5h    Pentagonal bipyramid
COC-7              4 C3v    Capped octahedron
CTPR-7             5 C2v    Capped trigonal prism
JPBPY-7            6 D5h    Johnson pentagonal bipyramid J13
JETPY-7            7 C3v    Johnson elongated triangular pyramid J7

Structure [ML7 ]        HP-7        HPY-7       PBPY-7        COC-7        CTPR-7       JPBPY-7       JETPY-7
 HABLII_0       ,      33.732,      8.993,      12.459,      6.444,       8.055,       15.741,       18.222
```

Please, note that, once again, none of the predefined shapes displayed CShM values below 3 units.
We have 11 new thermally distinguishable shapes for coordination number 7. Unfortunately, we would have to test each of them separately, one by one. Since we already know that the shape of this HOYGEL complex is the hemicube HECU-7, we wrote in the second line "%reference HECU-7", and added the zero in the fifth line as before.

The contents of "*HOYGEL_HECU-7.dat*" now become:

```
$ CN-7 structures
%reference HECU-7
! Coordinate atoms  Metal
       7          1
0 1 2 3 4 5 6 7

HABLII_0
 Eu   1.96030     4.810200     11.47920
  I   1.84900     3.604300     14.51880
  N   4.47830     3.726700     11.05320
  N   1.95730     2.189600     10.65450
  N   0.15470     4.276200     9.466600
  N   1.74300     7.409900     12.42830
  N   3.95120     6.609000     10.64540
  N  -0.63620     5.631900     11.99900
```

We now run **./shape_2.1_linux32** in the terminal again and get as output file "*HOYGEL_HECU-7.tab*", whose contents are shown below:

```
-------------------------------------------------------------------------------
S H A P E   v2.1        Continuous Shape Measures calculation
(c) 2013  Electronic Structure Group, Universitat de Barcelona
                  Contact:  llunell@ub.edu
-------------------------------------------------------------------------------

CN-7 structures

HECU-7          0 C2v   Hemicube
HP-7            1 D7h   Heptagon
HPY-7           2 C6v   Hexagonal pyramid
PBPY-7          3 D5h   Pentagonal bipyramid
COC-7           4 C3v   Capped octahedron
CTPR-7          5 C2v   Capped trigonal prism
JPBPY-7         6 D5h   Johnson pentagonal bipyramid J13
JETPY-7         7 C3v   Johnson elongated triangular pyramid J7

Structure [ML7 ]      HECU-7        HP-7       HPY-7       PBPY-7       COC-7       CTPR-7      JPBPY-7      JETPY-7
 HABLII_0       ,      0.607,      33.732,      8.993,      12.459,      6.444,       8.055,      15.741,      18.222
```

Now, the CShM value for the shape HECU-7 is 0.607, indicating that the Hemicube is the correct shape for this coordination polyhedron.
